# Apolipoprotein *E-C1-C4-C2* gene cluster region and inter-individual variation in plasma lipoprotein levels: a comprehensive genetic association study in two ethnic groups

**DOI:** 10.1371/journal.pone.0214060

**Published:** 2019-03-26

**Authors:** Dilek Pirim, Zaheda H. Radwan, Xingbin Wang, Vipavee Niemsiri, John E. Hokanson, Richard F. Hamman, Eleanor Feingold, Clareann H. Bunker, F. Yesim Demirci, M. Ilyas Kamboh

**Affiliations:** 1 Department of Human Genetics, Graduate School of Public Health, University of Pittsburgh, Pittsburgh, Pennsylvania, United States of America; 2 Department of Molecular Biology and Genetics, Faculty of Arts&Science, Uludag University, Gorukle, Bursa, Turkey; 3 Department of Epidemiology, Colorado School of Public Health, University of Colorado Denver, Aurora, Colorado, United States of America; 4 Department of Epidemiology, Graduate School of Public Health, University Pittsburgh, Pittsburgh, Pennsylvania, United States of America; Duke University, UNITED STATES

## Abstract

The apolipoprotein *E-C1-C4-C2* gene cluster at 19q13.32 encodes four amphipathic apolipoproteins. The influence of *APOE* common polymorphisms on plasma lipid/lipoprotein profile, especially on LDL-related traits, is well recognized; however, little is known about the role of other genes/variants in this gene cluster. In this study, we evaluated the role of common and uncommon/rare genetic variation in this gene region on inter-individual variation in plasma lipoprotein levels in non-Hispanic Whites (NHWs) and African blacks (ABs). In the variant discovery step, the *APOE*, *APOC1*, *APOC4*, *APOC2* genes were sequenced along with their flanking and hepatic control regions (*HCR1* and *HCR2*) in 190 subjects with extreme HDL-C/TG levels. The next step involved the genotyping of 623 NHWs and 788 ABs for the identified uncommon/rare variants and common tagSNPs along with additional relevant SNPs selected from public resources, followed by association analyses with lipid traits. A total of 230 sequence variants, including 15 indels were identified, of which 65 were novel. A total of 70 QC-passed variants in NHWs and 108 QC-passed variants in ABs were included in the final association analyses. Single-site association analysis of SNPs with MAF>1% revealed 20 variants in NHWs and 24 variants in ABs showing evidence of association with at least one lipid trait, including several variants exhibiting independent associations from the established *APOE* polymorphism even after multiple-testing correction. Overall, our study has confirmed known associations and also identified novel associations in this genomic region with various lipid traits. Our data also support the contribution of both common and uncommon/rare variation in this gene region in affecting plasma lipid profile in the general population.

## Introduction

Dyslipidemia with elevated low-density lipoprotein cholesterol (LDL-C) and reduced high-density lipoprotein cholesterol (HDL-C) is a major risk factor for cardiovascular disease, the leading cause of death worldwide [1]. Plasma lipoprotein-lipid variation is under genetic control and its estimated heritability ranges between 40–80% [2,3]. Although more than 100 lipid-associated loci have been identified, common variants in these loci explain only a small proportion of estimated heritability for lipid traits [4–9]. This indicates that there could be additional uncommon/rare variants that might contribute to the remaining unexplained heritability. Therefore, sequencing of candidate genes in subjects with extreme lipid traits would be an appropriate approach to identify all potentially causal common and uncommon/rare variants affecting plasma lipid trait variation.

The *APOE-C1-C4-C2* gene cluster, encoding four amphipathic apolipoproteins and encompassing ~45 kb, is located on chromosome 19q13.32. This cluster also includes two hepatic control regions (*HCR1* and *HCR2*) that regulate the hepatic expression of these genes [10–17]. ApoE participates in reverse cholesterol transport mechanism and mediates hepatic uptake of triglycerides-rich lipoprotein [18]. ApoC1 is involved in lecithin cholesterol acyl transferase activation [19] and cholesterol ester transfer protein inhibition [20,21]. ApoC4 is involved in triglyceride (TG) metabolism [22] and apoC2 is a cofactor for lipoprotein lipase enzyme [23]. Although the major contribution of the *APOE-C1-C4-C2* gene cluster is in the regulation of LDL-related traits, recent genome-wide association studies (GWAS) also reported their significant associations with TG and HDL-C levels [4,5,8]

Our group has previously reported the sequencing-based analysis of *APOE* genetic variation and its association with plasma lipoprotein traits in non-Hispanic Whites (NHWs) and African blacks (ABs) [24]. The objective of this study was to extend our work to include the entire *APOE-C1-C4-C2* gene region in order to comprehensively evaluate the association of *APOE-C1-C4-C2* common [minor allele frequency (MAF) ≥0.05] and uncommon/rare genetic variation with plasma lipid traits in NHWs and ABs. To achieve this objective we initially sequenced the four genes along with their 5’ & 3’ flanking regions [*APOE* (5,491 bp), *APOC1* (6,687 bp), *APOC4* (5,086 bp), *APOC2* (6,438 bp)] and their hepatic control regions [*HCR1* (820 bp) and *HCR2* (849 bp)] in selected subjects with extreme lipid traits [falling in the upper 10^th^ percentile (47 NHWs, 48 Blacks) and lower 10^th^ percentile (48 NHWs and 47 Blacks) of HDL-C/TG distribution], followed by genotyping of identified relevant single-nucleotide polymorphisms (SNPs) in NHWs (n = 623) and ABs (n = 788) for genotype-phenotype association analyses with plasma lipid traits.

## Materials & methods

### Study samples

The study was conducted on two well-characterized epidemiological samples, including 623 NHWs and 788 ABs (S1 Table). NHW samples were collected as part of San Luis valley Diabetes Study (SLVDS) and AB samples as part of a previous study on coronary heart disease risk factors in Benin City, Nigeria. The study details, including methods and sample characteristics can ben found elsewhere [25, 26, 27, 28]. All NHW subjects included in this study were non-diabetic. While LDL-C, HDL-C and TG levels were measured in all subjects, apoB and apoA1 measurements were available only in a subset of individuals [29, 30]. This study was approved by the University of Pittsburgh Institutional Review Board, and all study participants provided written informed consent. DNAs obtained from the study subjects (extracted from blood clots in ABs and buffy coats in NHWs following standard procedures) were used for the sequencing and genotyping experiments (described below) following whole genome amplification.

### DNA sequencing

Subjects with extreme HDL-C/TG levels falling in the upper 10^th^ percentile (47 NHWs and 48 ABs) and lower 10^th^ percentile (48 NHWs and 47 ABs) of HDL-C/TG distribution were selected for the initial sequencing-based variant discovery (S2 Table).

All four genes (*APOE*, *APOC1*, *APOC4*, *APOC2*) along with their 5’ & 3’ flanking regions and their two hepatic control regions (*HCR1*, and *HCR2*), which represent more than 50% (excluding intergenic regions and *APOCIPI* pseudogene) of the entire *APOE/C1/C1P1/C4/C2* gene cluster region (~45 kb), were targeted for sequencing in 190 individuals with extreme HDL-C/TG levels from two ethnic groups (NHWs and Blacks) using Sanger sequencing method. The targeted region sizes were 5,491 bp for *APOE*, 6,687 for *APOC1*, 5,086 bp for *APOC4*, 6,438 bp for *APOC2*, 820 bp for *HCR1*, and 849 bp for *HCR2*. We used SeattleSNPs reference sequences for *APOE*, *APOC1*, *APOC4*, and *APOC2* and NCBI database (build 137) to locate *HCR1* and *HCR2* reference sequences according to Allan et al. (1995) [12] and Dang et al. (1995) [17]. For the genes with insufficient 5’ and/or 3’ flanking region coverage at SeattleSNPs database, additional sequences were adopted from Chip Bioinformatics database. PCR amplification of targeted genomic regions was performed using either M13 tagged primers listed at SeattleSNPs database or the primers that we designed using the Primer3 software (see S3 Table for primer sequences), in order to generate overlapping PCR amplicons covering each targeted region (PCR conditions are available upon request). Automated Sanger sequencing of generated PCR amplicons was performed (in both directions) in a commercial lab (Beckman Coulter Genomics, Danvers, MA). We used Variant Reporter (Applied Biosystems, Foster City, CA) and Sequencher (Gene Codes Corporation, Ann Arbor, MI) software to analyze the resulting sequencing data for variant detection.

### Variant selection for follow-up genotyping

We analyzed the sequencing data separately in each ethnic group to identify common tagSNPs (MAF≥0.05, *r*^*2*^ = 0.9) and uncommon/rare variants (MAF<0.05) to be included in follow-up genotyping of NHW and AB samples. Suspicious sequence variants with borderline quality (that warrant validation) and additional common variants reported in SeattleSNPs and/or Chip Bioinformatics databases within the sequenced regions (but not successfully captured by our sequencing) were also targeted for follow-up genotyping. Moreover, additional common tagSNPs were selected from the HapMap database for genotyping in order to achieve a full coverage of the entire region of interest at 19q13.32 (including intergenic regions) for common variation in each ethnic group.

### Genotyping

The iPLEX Gold (Sequenom, San Diego, CA, USA) or TaqMan (Applied Biosystem, foster City, CA, USA) methods following manufacturer’s protocol were employed to genotype selected variants in the entire sample sets of 623 NHWs and 788 ABs. Whole genome amplified DNAs in 384-well plates were used both genotyping methods. The ABI Prism 7900HT Sequence Detection Systems was used for end-point fluorescence reading for TaqMan genotyping and genotype calls were analyzed by using the SDSv2.4.1 and TaqMan Genotyper software. The MassARRAY iPlex Gold (Sequenom, San Diego, CA) genotyping technique was applied in Genomics and Proteomics Core laboratories of the University of Pittsburgh. In addition to random replicates included in the genotyping process for quality control (QC) assessment, the subsets of samples used in both sequencing and genotyping steps allowed us to also evaluate the concordance between the sequencing and genotyping results. QC filters used for genotyped variants included extensive missing data (>15%) and/or deviation from Hardy-Weinberg Equilibrium (HWE) (*P*<0.01).

### Statistical analyses

Analyses were performed separately in NHWs and ABs. The haploview software (www.broadinstitute.org/haploview) was used to analyze the sequencing data to determine SNP allele/genotype frequencies, SNPs concordance with HWE, and their linkage disequilibrium (LD) patterns.

The Box-Cox transformation was used to normalize the distribution of apoB, HDL-C and TG levels in NHWs and that of all lipid traits in ABs. Significant covariates for each trait were identified using stepwise regression to select the most parsimonious set of covariates for each trait in each ethnic group [gender, age, BMI, and smoking in NHWs; gender, age, BMI, waist measurement, smoking, exercise (minutes walking or bicycling to work each day), and staff level (junior or senior, an indicator of lower or higher socio-economic status) in ABs]. A total of 70 QC-passed variants in NHWs and 108 QC-passed variants in Blacks were included in final association analyses with lipid traits. In addition to single-site, haplotype-based and uncommon/rare variant association analyses (conducted using R program) were also performed.

In single-site association analysis, additive linear regression model was used to test the associations between SNPs and plasma lipid levels (HDL-C, LDL-C, TC, and TG) and apoB and apoA1 levels. A P-value of less than 0.05 was considered as suggestive evidence of association for initial observations. P-values were also adjusted for the *APOE2/3/4* polymorphism given its established effect on cholesterol levels. After applying the Meff (effective number of independent tests) method for multiple-testing correction [31], 8 and 14 independent tests were identified for 70 QC-passed variants in NHWs and 108 QC-passed variants in ABs, respectively. Thus, after correcting for the number of independent tests performed, we considered P<6.25E-03 (0.05/8) and P<3.57E-03 (0.05/14) as statistically significant in NHWs and Blacks, respectively.

For haplotype association analysis, the generalized linear model (GLM) [32] was used. Because including too many haplotypes can make this analysis inefficient and impractical, we used a sliding window approach (4-SNP per window, sliding one SNP at a time) and assessed evidence for association within each window. A global P-value for overall effect of all haplotypes (with frequency greater than 0.01) in each window was used to assess their association with lipid traits. The sliding-window haplotype analysis was performed using the haplo.glm function in the Haplo.Stats R package.

The cumulative effects of uncommon/rare variants (MAF<0.05) were analyzed by using the SKAT-O method [33], which has been proposed to be the optimal test for rare variant analysis that exceeds the SKAT and burden tests. The analyses were performed using three different MAF bin thresholds (≤1%, ≤2% and <5%) by employing the SKAT R package.

## Results

### DNA sequencing

Sequencing of four genes (*APOE*, *APOC1*, *APOC4*, *APOC2*) along with their 5’ and 3’ flanking regions and their two hepatic control regions (*HCR1* and *HCR2*) in selected 190 subjects with extreme HDL-C/TG levels revealed a total of 230 variants (215 substitutions and 15 indels), of which 160 were previously reported and 65 were novel (not reported in public databases). While 63 of 230 variants were present in both ethnic groups, 52 were specific to NHWs and 115 were specific to ABs.

In NHWs, a total of 115 variants were identified (S4 Table), of which 27 were novel. Of 115 variants, 19 were mapped to *APOE* (1 novel), 29 to *APOC1* (9 novel), 5 to *HCR1* (2 novel), 3 to *HCR2* (1 novel), 21 to *APOC4* (6 novel), 2 each to *APOC4* and *APOC2*, and 36 to *APOC2* (8 novel).

In ABs, a total of 178 variants were identified (S5 Table), of which 42 were novel. Of 178 variants, 31 were mapped to *APOE* (9 novel), 23 to *APOC1* (3 novel), 3 to *HCR1*, 4 to *HCR2* (2 novel), 46 to *APOC4* (10 novel), 5 each to *APOC4* and *APOC2*, and 66 to *APOC2* (18 novel).

All novel SNPs and short indels identified in this study [excluding a large indel (114 bp) in *APOC4* 5' flanking region] were submitted to dbSNP database (http://www.ncbi.nlm.nih.gov/SNP/snp_viewTable.cgi?handle=KAMBOH) and assigned refSNP IDs can be found in S4 and S5 Tables.

### Genotyping

Tagger analysis results for the identified common sequence variants (MAF≥0.05, *r*^*2*^ = 0.9) are presented in S6 Table for NHWs and S7 Table for ABs. S8 and S9 Tables summarize the HapMap tagSNPs in CEU (Utah residents with Northern and Western European ancestry from the CEPH collection) and YRI (Yoruba in Ibadan, Nigeria) populations, respectively.

Initially, a total of 103 variants were selected for follow-up genotyping in NHWs, consisting of 90 variants identified by sequencing (33 common SNPs based on Tagger results, 53 uncommon/rare variants, and 4 suspicious variants), 6 additional common SNPs reported in SeattleSNPs and/or Chip Bioinformatics databases within the sequenced regions, and 7 additional tagSNPs from the HapMap database. Probably because of the high degree of sequence homology among the members of this gene cluster, we observed a relatively high failure rate; a total of 22 variants (6 common SNPs, 15 uncommon/rare variants and one HapMap SNP) failed genotyping. Eleven out of 81 genotyped variants were excluded from final statistical analyses, including 4 suspicious variants that turned out to be sequencing artifacts, 4 database SNPs (according to Chip Bioinformatics and SeattleSNPs; rs12721047, rs12709888, rs76186107, rs5164) and one HapMap SNP (rs5127) that turned out to be monomorphic in our NHW sample, and 2 variants that failed post-genotyping QC (*APOE*/rs769446 had low call rate and *APOC1*/rs12721052 was out of HWE). Therefore, a total of 70 QC-passed variants (65 variants identified by sequencing and 5 additional tagSNPs selected from HapMap) were included in final association analyses in NHWs, comprising 29 common SNPs (MAF≥0.05) and 41 uncommon/rare variants (MAF<0.05) (see S10 Table).

Initially, a total of 160 variants were selected for follow-up genotyping in ABs, consisting of 152 variants identified by sequencing (58 common SNPs based on Tagger results, 90 uncommon/rare variants, and 4 suspicious variants), one additional common SNP reported in SeattleSNPs and/or Chip Bioinformatics databases within the sequenced regions, and 7 additional tagSNPs from the HapMap database. Since genotyping of variants in this gene cluster region is challenging, we ended up with a total of 42 failures: 12 common SNPs, 27 uncommon/rare variants, and 3 suspicious variants. Ten out of 118 genotyped variants were excluded from final statistical analyses because they either turned out to be monomorphic, were out of HWE, or had low call rate. Thus, a total of 108 QC-passed variants (103 variants identified by sequencing and 5 additional tagSNPs selected from HapMap) were advanced into final association analyses in ABs, comprising 48 common SNPs (MAF≥0.05) and 60 uncommon/rare variants (MAF<0.05) (see S11 Table).

### Association analyses

#### Single-site association analysis

Single-site association analysis revealed 20 variants (MAF>1%) in NHWs and 24 (MAF>1%) in ABs with suggestive evidence of association (*P* <0.05) with at least one lipid trait, including the two SNPs (rs7412 and rs429358) that define the *APOE* 2/3/4 polymorphism (Tables 1 & 2). After adjusting the observed associations for the effects of APOE*2/rs7412 and APOE*4/rs429358, 11 of 18 variants in NHWs and 15 of 22 variants in ABs exhibited independent associations, including one variant (*APOE*/rs440446) showing association with LDL-related traits in both populations (LDL-C, TC in NHWs; apoB in ABs).

**Table 1:** Significant single-site association analysis results for lipid traits in NHWs.

**Variant**						**LDL-C**				**HDL-C**^a^				**TC**			
**Gene**	**RefSNP ID**	**Locations**	**RegulomeDB scores**	**Associated Allele**	**MAF**	***B***	**P**	**Adj. *B***^b^	**Adj. P**^b^	***B***	**P**	**Adj. *B***^b^	**Adj. P**^b^	***B***	**P**	**Adj. *B***^**1**^	**Adj. P**^b^
***APOE***	*rs449647	5'flanking	5	T	0.161	-7.12	**0.025**	-0.784	0.815	-0.01	0.353	-0.01	0.528	-6.71	0.043	-1.705	0.626
***APOE***	*rs405509	5'flanking	1f	T	0.478	0.42	0.854	-5.048	**0.039**	0.01	0.403	0.012	0.292	-0.68	0.776	-6.093	**0.017**
***APOE***	*rs440446	Intron 1	4	C	0.36	-2.59	0.281	-5.901	**0.024**	0.01	0.287	0.007	0.598	-4.26	0.089	-7.47	**0.006**
***APOE***	rs769448	Intron 1	4	T	0.021	0.75	0.923	-0.722	0.924	0.08	0.02	0.082	**0.025**	1.88	0.817	0.389	0.961
***APOE***	rs769449	Intron 2	4	A	0.116	6.95	0.055	0.088	0.99	-0.01	0.58	0.036	0.282	8.31	**0.029**	4.09	0.574
***APOE***	rs769450	Intron 2	5	A	0.401	4.89	0.039	5.29	**0.037**	0.003	0.773	-0.005	0.688	6.88	0.005	6.917	**0.009**
***APOE***	*rs429358 *(E4)*	Exon 4	5	C	0.152	8.1	**0.01**	-	-	-0.02	0.223	-	-	6.82	**0.038**	-	-
***APOE***	*rs7412 (*E2*)	Exon 4	5	T	0.081	-21.84	**1.84E-07**	-	-	-0.01	0.452	-	-	-19.46	**9.51E-06**	-	-
***Intergenic***	*rs439401	Intergenic	1b	T	0.36	-1.76	0.452	-5.527	**0.031**	0.01	0.545	0.001	0.929	-2.82	0.249	-6.488	**0.016**
***Intergenic***	rs445925	Intergenic	No Data	A	0.11	-12.58	5.00E-04	2.932	0.683	-0.02	0.272	-0.015	0.664	-13.53	3.20E-04	0.046	0.995
***APOC1***	rs3826688	Intron 2	5	A	0.342	-2.38	0.325	-5.796	**0.028**	0.01	0.399	0.004	0.776	-3.6	0.154	-6.812	**0.013**
***APOC1***	rs12721046	Intron 3	6	A	0.152	6.93	0.031	2.15	0.645	-0.0001	0.997	0.027	0.234	5.95	0.077	3.266	0.506
***APOC1***	rs1064725	3'UTR	No Data	G	0.039	11.99	0.042	10.922	0.058	0.03	0.325	0.024	0.384	13.65	0.026	12.813	**0.033**
***APOC1***	*rs56131196	3'flanking	No Data	A	0.189	7.24	0.014	1.441	0.8	-0.01	0.615	0.025	0.362	6.69	0.03	5.863	0.325
***APOC1***	rs4420638	3'flanking	No Data	G	0.157	6.97	0.026	4.378	0.469	-0.01	0.57	0.024	0.435	6.79	0.04	10.72	0.093
***Intergenic***	rs4803770	Intergenic	5	G	0.378	5.52	0.0197	6.159	**0.016**	0.003	0.765	-0.003	0.828	6.08	0.014	6.434	**0.016**
***APOC1P1***	rs5112	*APOC1P1*	4	C	0.463	-4.61	0.0515	-6.893	**0.004**	-0.01	0.653	-0.008	0.49	-4.95	0.045	-7.129	**0.004**
***APOC1P1***	rs7259004	*APOC1P1*	6	G	0.118	-9.78	0.005	-3.218	0.419	0.01	0.712	0.02	0.3	-9.06	0.013	-1.574	0.707
***HCR2***	rs35136575	HCR2	2a	G	0.227	-3.15	0.2324	-4.769	0.068	0.03	0.033	0.027	**0.034**	-2.25	0.414	-3.814	0.163
***APOC4***	rs12721109	Intron 1	2b	A	0.024	-19.14	0.0092	-3.627	0.64	0.03	0.386	0.056	0.135	-12.75	0.098	-7.586	0.349
**Variant**						**TG**^a^				**ApoB**^a^				**ApoA1**			
**Gene**	**RefSNP ID**	**Locations**	**RegulomeDB scores**	**Associated Allele**	**MAF**	***B***	**P**	**Adj. *B***^b^	**Adj. P**^b^	***B***	**P**	**Adj. *B***^b^	**Adj. P**^b^	***B***	**P**	**Adj. *B***^b^	**Adj. P**^b^
***APOE***	*rs449647	5'flanking	5	T	0.161	-0.02	0.523	-0.027	0.434	-0.95	0.129	0.524	0.409	-1.57	0.618	-4.095	0.232
***APOE***	*rs405509	5'flanking	1f	T	0.4775	-0.07	0.003	-0.077	**0.002**	1.46	9.00E-04	-0.051	0.914	-1.8	0.417	-0.142	0.956
***APOE***	*rs440446	Intron 1	4	C	0.3604	-0.08	0.002	-0.087	**0.001**	0.56	0.232	-0.231	0.642	-0.19	0.935	-0.086	0.975
***APOE***	rs769448	Intron 1	4	T	0.021	-0.09	0.232	-0.094	0.229	0.94	0.52	0.407	0.767	3.44	0.639	3.977	0.594
***APOE***	rs769449	Intron 2	4	A	0.1165	0.01	0.687	0.049	0.494	2.03	0.003	-0.19	0.886	-4.38	0.205	-3.037	0.672
***APOE***	rs769450	Intron 2	5	A	0.4015	0.06	0.008	0.083	**0.002**	0.15	0.742	0.196	0.685	0.7	0.766	0.337	0.897
***APOE***	*rs429358 *(E4)*	Exon 4	5	C	0.1525	0.01	0.707	-	-	2.14	**5.00E-04**	-	-	-3.66	0.24	-	-
***APOE***	*rs7412 (*E2*)	Exon 4	5	T	0.0806	0.01	0.744	-	-	-5.6	**9.65E-13**	-	-	5.12	0.208	-	-
***Intergenic***	*rs439401	Intergenic	1b	T	0.3596	-0.06	0.019	-0.072	**0.006**	0.39	0.398	-0.433	0.374	0.91	0.692	0.978	0.71
***Intergenic***	rs445925	Intergenic	No Data	A	0.1094	0.01	0.825	-0.071	0.338	-3.78	5.17E-08	-0.287	0.829	3.53	0.323	1.237	0.864
***APOC1***	rs3826688	Intron 2	5	A	0.3424	-0.08	0.001	-0.094	**0.001**	0.3	0.517	-0.485	0.322	0.24	0.918	0.389	0.883
***APOC1***	rs12721046	Intron 3	6	A	0.1522	0.02	0.479	0.033	0.494	1.59	0.01	0.242	0.778	-2.11	0.496	0.919	0.843
***APOC1***	rs1064725	3'UTR	No Data	G	0.0388	-0.05	0.406	-0.049	0.417	2	0.093	1.452	0.194	-4.32	0.473	-3.909	0.521
***APOC1***	*rs56131196	3'flanking	No Data	A	0.1885	0.01	0.768	0.009	0.877	1.75	0.002	0.42	0.685	-2.59	0.37	0.337	0.952
***APOC1***	rs4420638	3'flanking	No Data	G	0.1556	0.03	0.392	0.048	0.456	1.43	0.018	0.408	0.712	-1.1	0.716	0.153	0.98
***Intergenic***	rs4803770	Intergenic	5	G	0.3779	0.04	0.08	0.061	**0.023**	0.56	0.219	0.603	0.2	0.02	0.994	0.253	0.923
***APOC1P1***	rs5112	*APOC1P1*	4	C	0.4633	-0.02	0.322	-0.019	0.444	-0.15	0.755	-0.699	0.118	-0.67	0.777	-0.146	0.953
***APOC1P1***	rs7259004	*APOC1P1*	6	G	0.1176	0.05	0.21	0.051	0.224	-1.33	0.052	0.889	0.236	6.89	0.046	7.775	0.057
***HCR2***	rs35136575	HCR2	2a	G	0.2274	-0.03	0.283	-0.029	0.283	-0.48	0.366	-0.878	0.078	5.16	0.05	5.879	**0.028**
***APOC4***	rs12721109	Intron 1	2b	A	0.0237	-0.16	0.033	-0.207	**0.01**	-4.99	0.001	-1.863	0.204	-9.31	0.196	-12.623	0.104

MAF: minor allele frequency. LDL-C: low-density lipoprotein cholesterol; HDL-C: high-density lipoprotein cholesterol; TC: total cholesterol; TG: triglyceride; ApoB: Apolipoprotein B; ApoA1: apolipoprotein A1. Age, gender, smoking, and BMI were significant covariates that were included in all association analyses.

^a^Box-Cox transformed variables.

^b^APOE*2/E*4 adjusted results.**Bold** values represent significant P-values for the SNPs showing independent associations after adjusting for the effects of *APOE* epsilon polymorphism (E*2/E*4).**Underlined** values represent significant P-values after multiple-testing correction (P<6.25E-03).

*Variants showing significant associations in NHWs and ABs.

**Table 2:** Significant single-site association analysis results for lipid traits in ABs.

**Variant**						**LDL-C**^a^				**HDL-C**^a^				**TC**^a^			
**Gene**	**RefSNP ID**	**Locations**	**RegulomeDB scores**	**Associated Allele**	**MAF**	***B***	**P**	**Adj. *B***^b^	**Adj. P**^b^	***B***	**P**	**Adj. *B***^b^	**Adj. P**^b^	***B***	**P**	**Adj. *B***^b^	**Adj. P**^b^
***APOE***	rs1081101	5'flanking	4	T	0.061	-0.62	0.111	-0.683	0.083	0.3	0.427	0.321	0.415	-0.2	0.328	-0.221	0.292
***APOE***	*rs449647	5'flanking	5	T	0.366	0.58	0.003	0.414	0.051	-0.18	0.356	-0.178	0.411	0.25	0.018	0.171	0.134
***APOE***	*rs405509	5'flanking	1f	T	0.256	0.6	0.004	0.451	0.053	-0.19	0.361	-0.094	0.691	0.22	0.053	0.152	0.224
***APOE***	*rs440446	Intron 1	4	C	0.1	0.44	0.179	0.488	0.147	-0.54	0.095	-0.603	0.074	0.12	0.475	0.105	0.558
***APOE***	rs61357706	Intron 2	5	A	0.017	-2.05	0.006	-2.27	**0.003**	0.77	0.291	0.929	0.217	-0.69	0.077	-0.728	0.068
***APOE***	*rs429358 *(E4)*	Exon 4	5	C	0.266	0.46	**0.032**	-	-	-0.14	0.507	-	-	0.17	0.132	-	-
***APOE***	rs769455	Exon 4	5	T	0.02	-2.23	0.001	-2.439	**3.49E-04**	-0.46	0.483	-0.381	0.575	-0.87	0.013	-0.933	**0.01**
***APOE***	*rs7412 *(E2)*	Exon 4	5	T	0.061	-2.05	**5.35E-07**	-	-	0.75	0.066	-	-	-0.82	**1.00E-04**	-	-
***Intergenic***	*rs439401	Intergenic	1b	T	0.109	0.39	0.193	0.365	0.235	-0.6	0.044	-0.58	0.065	0.07	0.662	0.036	0.828
***APOC1***	rs11568822	5'flanking	4	InsCGTT	0.274	-0.57	0.008	-0.275	0.235	0.27	0.209	0.144	0.543	-0.2	0.074	-0.083	0.508
***APOC1***	rs10424339	Intron 3	No Data	G	0.14	-0.02	0.937	-0.018	0.951	0.55	0.046	0.575	**0.048**	0.07	0.637	0.07	0.65
***APOC1***	rs12721054	3'UTR	6	G	0.145	-0.77	0.006	-0.63	**0.027**	0.23	0.422	0.188	0.527	-0.3	0.047	-0.262	0.094
***APOC1***	*rs56131196	3'flanking	No Data	A	0.175	-0.22	0.393	-0.269	0.306	0.31	0.225	0.311	0.243	-0.05	0.73	-0.086	0.542
***APOC4***	rs12721105	5’flanking	5	T	0.038	0.14	0.778	0.062	0.901	-0.19	0.694	-0.344	0.489	0.13	0.619	0.059	0.824
***APOC4***	rs5157	Intron 1	4	C	0.172	0.28	0.27	0.36	0.166	-0.14	0.596	-0.135	0.608	0.13	0.329	0.149	0.284
***APOC4***	rs5158	Intron 1	2b	T	0.021	0.55	0.414	0.568	0.406	-0.29	0.662	-0.39	0.565	0.01	0.971	-0.004	0.991
***APOC2-C4***	rs12709885	C4-3'/C2-5'	5	T	0.018	-1.25	0.086	-1.152	0.123	1.1	0.135	1.345	0.08	-0.55	0.155	-0.374	0.353
***APOC2-C4***	rs2288912	C4-3'/C2-5'	1a	C	0.258	0.51	0.02	0.473	**0.033**	0.05	0.809	0.121	0.587	0.28	0.015	0.262	**0.027**
***APOC2-C4***	rs75463753	C2-Intron1	4	A	0.108	0.8	0.011	0.713	**0.026**	0.09	0.771	0.14	0.666	0.42	0.011	0.394	**0.02**
***APOC2***	rs9304645	Intron 1	4	A	0.366	-0.54	0.008	-0.389	0.064	0.09	0.653	0.043	0.841	-0.2	0.074	-0.122	0.276
***APOC2***	rs11879392	Intron 1	2b	G	0.014	0.87	0.317	0.82	0.338	0.57	0.499	0.569	0.5	0.27	0.54	0.244	0.586
***APOC2***	rs5120	Intron 1	4	T	0.185	0.6	0.016	0.493	0.051	0.08	0.741	0.164	0.518	0.33	0.013	0.281	**0.037**
***APOC2***	rs10423208	3'flanking	5	G	0.316	0.42	0.047	0.311	0.144	0.03	0.876	0.084	0.695	0.18	0.117	0.127	0.264
***APOC2***	rs10422888	3'flanking	5	A	0.078	0.98	0.008	0.914	**0.015**	-0.01	0.986	0.077	0.838	0.48	0.013	0.456	**0.021**
**Variant**						**TG**^a^				**ApoB**^a^				**ApoA1**^a^			
**Gene**	**RefSNP ID**	**Location**	**RegulomeDB score**	**Associated Allele**	**MAF**	***B***	**P**	**Adj. *B***^b^	**Adj.P**^b^	***B***	**P**	**Adj. *B***^b^	**Adj. P**^b^	***B***	**P**	**Adj. *B***^b^	**Adj. P**^b^
***APOE***	rs1081101	5'flanking	4	T	0.061	0.038	0.009	0.037	**0.013**	0.65	0.549	0.464	0.674	-0.003	0.997	0.077	0.937
***APOE***	*rs449647	5'flanking	5	T	0.366	-0.001	0.848	0.001	0.934	0.92	0.095	1.056	0.077	-0.49	0.319	-0.207	0.695
***APOE***	*rs405509	5'flanking	1f	T	0.256	0.003	0.746	0.005	0.553	0.66	0.261	0.679	0.298	-0.67	0.199	-0.213	0.713
***APOE***	*rs440446	Intron 1	4	C	0.1	0.023	0.054	0.02	0.1	2.3	0.011	1.94	**0.037**	-0.44	0.58	-0.759	0.356
***APOE***	rs61357706	Intron 2	5	A	0.017	0.04	0.149	0.044	0.121	-2.56	0.22	-2.822	0.186	1.09	0.55	1.536	0.409
***APOE***	*rs429358 *(E4)*	Exon 4	5	C	0.266	-0.008	0.308	-	-	0.05	0.937	-	-	-1	0.059	-	-
***APOE***	rs769455	Exon 4	5	T	0.02	0.056	0.037	0.059	**0.035**	-3.45	0.066	-3.717	0.052	-0.71	0.664	-0.408	0.805
***APOE***	*rs7412 *(E2)*	Exon 4	5	T	0.061	-0.018	0.238	-	-	-2.35	**0.036**	-	-	3.85	**8.00E-05**	-	-
***intergenic***	*rs439401	Intergenic	1b	T	0.109	0.011	0.34	0.011	0.357	1.42	0.086	0.995	0.253	-0.5	0.502	-0.535	0.486
***APOC1***	rs11568822	5'flanking	4	InsCGTT	0.274	-0.013	0.114	-0.012	0.183	-1.1	0.064	-0.798	0.216	1.03	0.048	0.493	0.383
***APOC1***	rs10424339	Intron3	No Data	G	0.14	0.001	0.91	-0.003	0.76	-0.71	0.365	-0.827	0.315	0.4	0.559	0.302	0.674
***APOC1***	rs12721054	3'UTR	6	G	0.145	-0.028	0.007	-0.029	**0.006**	-1.01	0.188	-0.976	0.214	1.01	0.141	0.894	0.201
***APOC1***	*rs56131196	3'flanking	No Data	A	0.175	-0.019	0.044	-0.021	**0.036**	-0.7	0.338	-0.682	0.358	0.87	0.173	1.047	0.105
***APOC4***	rs12721105	5’ flanking	5	T	0.038	0.063	0.001	0.057	**0.002**	0.45	0.744	0.262	0.85	-1.4	0.239	-1.529	0.204
***APOC4***	rs5157	Intron 1	4	C	0.172	0.001	0.948	0.004	0.701	1.43	0.044	1.781	**0.014**	0.78	0.215	0.728	0.25
***APOC4***	rs5158	Intron 1	2b	T	0.021	-0.052	0.035	-0.049	0.05	1.44	0.446	1.505	0.433	-1.12	0.5	-1.157	0.487
***APOC2-C4***	rs12709885	C4-3/C2-5	5	T	0.018	-0.035	0.187	-0.036	0.194	-4.39	0.024	-5.312	**0.009**	5.95	0.001	5.48	**0.003**
***APOC2-C4***	rs2288912	C4-3'/C2-5	1a	C	0.258	-0.007	0.361	-0.006	0.467	1.37	0.023	1.422	**0.02**	0.8	0.133	1.034	0.054
***APOC2-C4***	rs75463753	C2-Intron1	4	A	0.108	-0.008	0.5	-0.008	0.491	1.08	0.218	0.967	0.278	0.48	0.538	0.937	0.236
***APOC2***	rs9304645	Intron 1	4	A	0.366	0.011	0.141	0.011	0.149	-0.21	0.718	-0.01	0.987	0.75	0.136	0.401	0.437
***APOC2***	rs11879392	Intron 1	2b	G	0.014	-0.069	0.029	-0.071	**0.024**	0.44	0.855	0.221	0.927	0.18	0.93	0.335	0.871
***APOC2***	rs5120	Intron 1	4	T	0.185	-1.00E-04	0.994	0.002	0.806	1.11	0.108	1.007	0.152	0.79	0.2	1.064	0.085
***APOC2***	rs10423208	3'flanking	5	G	0.316	-0.01	0.203	-0.008	0.314	0.34	0.559	0.225	0.706	-0.43	0.41	-0.231	0.658
***APOC2***	rs10422888	3'flanking	5	A	0.078	0.015	0.258	0.012	0.398	1.47	0.148	0.961	0.358	0.05	0.954	0.293	0.749

MAF: minor allele frequency. LDL-C: low-density lipoprotein cholesterol; HDL-C: high-density lipoprotein cholesterol; TC: total cholesterol; TG: triglyceride; ApoB: Apolipoprotein B; ApoA1: apolipoprotein A1. Gender, age, BMI, waist measurement, smoking, exercise, and staff level were significant covariates that were included in all association analyses.

^a^Box-Cox transformed variables.

^b^APOE*2/E*4 adjusted results. **Bold** values represent significant P-values for the SNPs showing independent associations after adjusting for the effects of *APOE* epsilon polymorphism (E*2/E*4). **Underlined** values represent significant p-values after multiple-testing correction (P<3.57E-03).

*Variants showing significant associations in NHWs and ABs.

The established associations of APOE*2/E*4 alleles with LDL-C are replicated in both ethnic groups included in this study (Tables 1 & 2); APOE*2/rs7412 was associated with lower LDL-C levels (P = 1.84E-07 in NHWs and P = 5.35E-07 in ABs) and APOE*4/rs429358 was associated with higher LDL-C levels (P = 0.01 in NHWs and P = 0.032 in ABs). In addition to their established association with LDL-C, we observed APOE*2/rs7412 to be associated with lower TC levels in both ethnic groups (P = 9.51E-06 in NHWs and P = 1.0E-04 in ABs), with lower apoB levels in both ethnic groups (P = 9.65E-13 in NHWs and P = 0.0356 in ABs) and with higher apoA1 levels in ABs (P = 8.0E-05); while APOE*4/rs429358 showed association with higher TC levels (P = 0.0383) and higher apoB levels (P = 5.0E-04) in NHWs and a non-significant but similar trend of association with TC levels in ABs.

**Fig 1:**
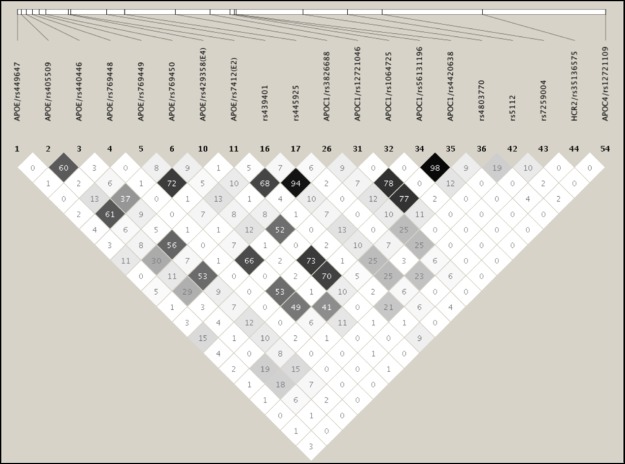
LD plot of the 20 common/uncommon variants (MAF>1%) showing suggestive evidence of association with lipid traits in NHWs. The values in the cells are the pairwise degree of LD indicated by *r*^2^ × 100. *r*^2^ = 0 is shown as white, 0 < *r*^*2*^ <1 is shown in gray and *r*^2^ = 1 is shown in black.

In NHWs, 8 additional SNPs (MAF>1%) showed independent associations with LDL-C and/or TC levels after adjusting for the effects of APOE*2/E*4 alleles (Table 1): *APOE*/rs405509 with LDL-C (Adj. P = 0.039) and TC (Adj. P = 0.017), *APOE*/rs440446 with LDL-C (Adj. P = 0.024) and TC (Adj. P = 0.006), *APOE*/rs769450 with LDL-C (Adj. P = 0.037) and TC (Adj. P = 0.009), rs439401 with LDL-C (Adj. P = 0.031) and TC (Adj. P = 0.016), *APOC1*/rs3826688 with LDL-C (Adj. P = 0.028) and TC (Adj. P = 0.013), *APOC1*/rs1064725 with only TC (Adj. P = 0.033), rs4803770 with LDL-C (Adj. P = 0.016) and TC (Adj. P = 0.016), and *APOC1P1/*rs5112 with LDL-C (Adj. P = 0.004) and TC (Adj. P = 0.004). Of these 8 SNPs, four (*APOE*/rs405509, *APOE*/rs440446, rs439401, and *APOC1*/rs3826688) exhibited significant association with LDL-C and/or TC only after adjusting for the *APOE* 2/3/4 polymorphism. Two of these 8 SNPs (*APOE*/rs440446 and *APOC1P1*/rs5112) maintained their independent significant association with LDL-C and/or TC even after multiple-testing correction (P<6.25E-03). None of these 8 SNPs were in significant LD (*r*^*2*^≤0.15) with the APOE*2/E*4 SNPs (Fig 1).

In addition to the association with LDL-related traits in NHWs, we observed one SNP (*APOE*/rs769448) showing association with only HDL-C levels (Adj. P = 0.025) and one SNP (*HCR2*/rs35136575) with both HDL-C (Adj. P = 0.034) and apoA1 (Adj. P = 0.028) levels (Table 1). Moreover, seven SNPs were associated with TG levels (Table 1); *APOE*/rs405509 (Adj. P = 0.002), *APOE*/rs440446 (Adj. P = 0.001), *APOE*/rs769450 (Adj. P = 0.002), rs439401 (Adj. P = 0.006), *APOC1*/rs3826688 (Adj. P = 0.001), rs4803770 (Adj. P = 0.023), and *APOC4*/rs12721109 (Adj. P = 0.010). Most of these SNPs were also nominally significant before the adjustment for APOE*2/E*4 alleles. Five SNPs maintained their significant association with TG even after multiple-testing correction (P<6.25E-03). While the apoA1 and/or HDL-C associated SNPs were not in LD with each other, a modest or high LD was observed within the subsets of TG associated SNPs (Fig 1).

**Fig 2:**
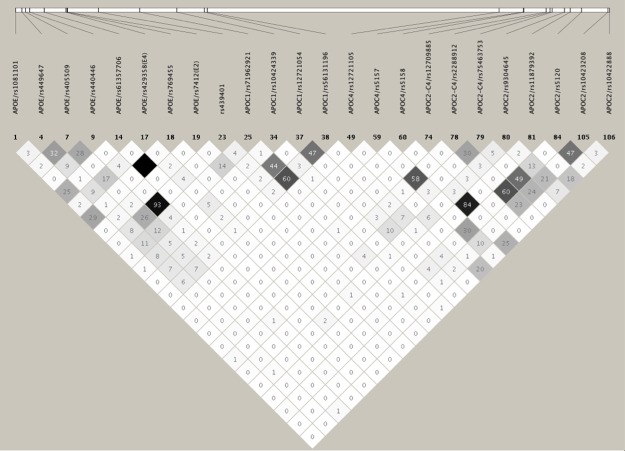
LD plot of the 24 common/uncommon variants (MAF>1%) showing suggestive evidence of association with lipid traits in African blacks. The values in the cells are the pairwise degree of LD indicated by *r*^*2*^ × 100. *r*^2^ = 0 is shown as white, 0 < *r*^2^ <1 is shown in gray and *r*^2^ = 1 is shown in black.

In ABs, we observed 10 SNPs (MAF>1%), in addition to APOE*2/rs7412 and APOE*4/rs429358 SNPs, to be associated with LDL-related traits after adjusting for the effects of APOE*2/E*4 alleles (Table 2). *APOC2-C4*/rs2288912 was associated with all LDL-related traits: LDL-C (Adj. P = 0.033), TC (Adj. β = 0.262; Adj. P = 0.027), and apoB (Adj. P = 0.020). Three variants were independently associated with two LDL-related traits: *APOE*/rs769455 with LDL-C (Adj. P = 3.49E-04) and TC (Adj. P = 0.012), *APOC2-C4*/rs75463753 with LDL-C (Adj. P = 0.026) and TC (Adj. P = 0.020), and *APOC2*/rs10422888 with LDL-C (Adj. P = 0.015) and TC (Adj. P = 0.021). Six variants were independently associated with only one LDL-related trait: *APOC2*/rs5120 with TC (Adj. P = 0.037), *APOE*/rs61357706 with LDL-C (Adj. P = 0.003), *APOC1*/rs12721054 with LDL-C (Adj. P = 0.027), *APOE*/rs440446 with apoB (Adj. P = 0.037), *APOC4*/rs5157 with apoB (Adj. P = 0.014), and *APOC2-C4*/rs12709885 with apoB (Adj. P = 0.009). Two of these 10 SNPs (*APOE*/rs769455 and *APOE*/rs61357706) maintained their independent significant association with LDL-C even after multiple-testing correction (P<3.57E-03). While these 2 SNPs were in strong LD with each other, they were not in LD with APOE*2/E*4 SNPs (Fig 2).

In addition to the association with LDL-related traits in ABs, we observed one SNP (*APOC1*/rs10424339) showing association with HDL-C levels (Adj. P = 0.048), one SNP (*APOC2-C4*/rs12709885) with apoA1 levels (Adj. P = 0.003), and six SNPs with TG levels: *APOE*/rs1081101 (Adj. P = 0.013), *APOE*/rs769455 (Adj. P = 0.035), *APOC1*/rs12721054 (Adj. P = 0.006), *APOC1*/rs56131196 (Adj. P = 0.036), *APOC4*/rs12721105 (Adj. P = 0.002), *APOC2*/rs11879392 (Adj. P = 0.024) (Table 2). TG-associated *APOC4*/rs12721105 SNP and apoA1-associated *APOC2-C4*/rs12709885 SNP remained significant (P<3.57E-03) even after multiple-testing correction. While the HDL-C or apoA1 associated SNPs were not in LD with each other, a low or modest LD was observed among some of TG associated SNPs (Fig 2). See Supplementary Data for the single-site association results for all tested variants (S12–S23 Tables).

#### Rare/Uncommon variants association analysis

In NHWs, while significant associations were observed with TC for all tested MAF thresholds (≤1%, ≤2% and <5%), the most significant result was detected for variants with MAF≤0.01 (P = 0.0088), indicating the major impact of rare variants on TC. In ABs, significant association was detected between variants with MAF≤0.01 and TG (P = 0.0302). On the other hand, variants with MAF≤2% and MAF<5% showed association with apoA1 (P = 0.025 to 0.021), indicating a modest effect of variants with MAF<5% on TG (see Table 3).

**Table 3:** Significant results for rare/uncommon variants (MAF<5%) association analysis* with lipid traits in NHWs and ABs.

MAF threshold		MAF ≤0.01		MAF≤0.02		MAF<0.05	
	Lipid trait	N.RV	P	N.RV	P	N.RV	P
**NHWs**	**TC**	31	**0.0088**	32	**0.0165**	41	**0.0498**
**Blacks**	^a^**TG**	29	**0.0302**	45	0.6291	^b^61	0.2645
	^a^**ApoA1**	29	0.0756	45	**0.0248**	^b^61	**0.0213**

= *Analysis was performed using SKAT-O (optimal sequencing Kernel association test); N.RV: number of variants with the defined MAF. Significant covariates that were included in all association analyses were age, gender, smoking, and BMI in NHWs and gender, age, BMI, waist measurement, smoking, exercise, and staff level in Blacks.

^a^Box-Cox transformed variables.

^**b**^ Includes 60 variants with MAF<0.05 and one variant with borderline MAF of 0.0499.

#### Haplotype-based association analysis

S24–S35 Tables and Figs 3–14 show the results for haplotype-based association analysis with lipid traits using the sliding window approach in NHWs and ABs, respectively. For each window, the most common haplotype was used as the reference haplotype to compare with other haplotypes to calculate the p-values.

**Fig 3:**
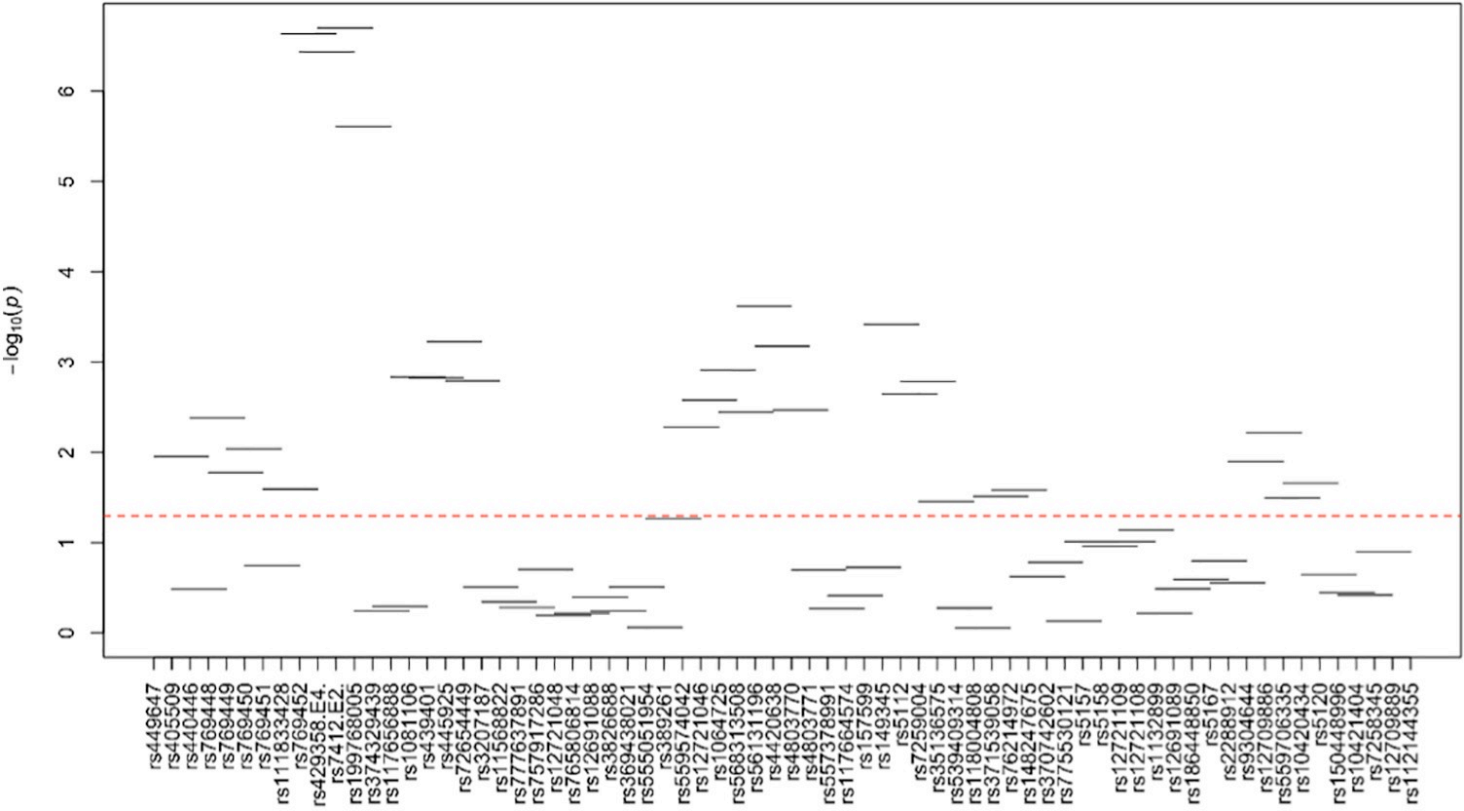
Plots of haplotype-based association analysis results for LDL-C in NHWs. Horizontal lines represent the 4-SNP windows. X-axis direction shows the SNPs genotyped in the regions based on the order of *APOE*, *APOC1*, *HCR1*, *HCR2*, *APOC4* and *APOC2* while the Y-axis shows the–log (global p-value) with red line representing the p-value threshold (P = 0.05).

**Fig 4:**
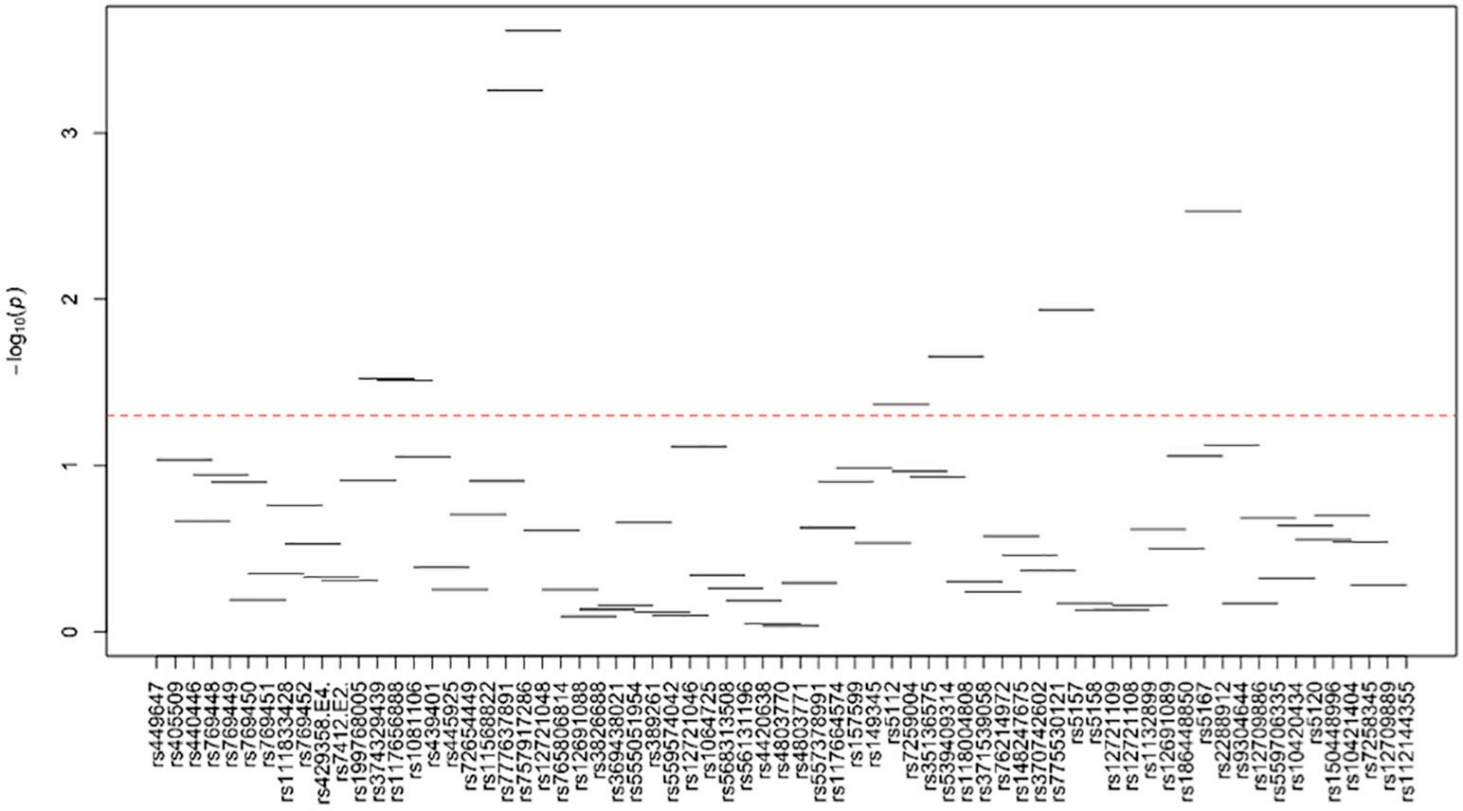
Plots of haplotype-based association analysis results for HDL-C in NHWs. Horizontal lines represent the 4-SNP windows. X-axis direction shows the SNPs genotyped in the regions based on the order of *APOE*, *APOC1*, *HCR1*, *HCR2*, *APOC4* and *APOC2* while the Y-axis shows the–log (global p-value) with red line representing the p-value threshold (P = 0.05).

**Fig 5:**
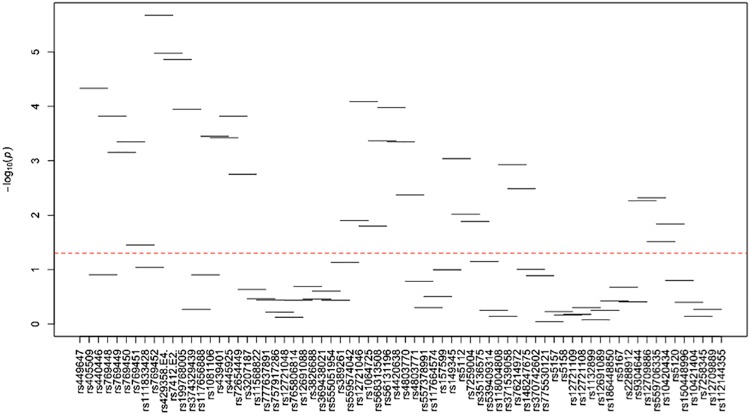
Plots of haplotype-based association analysis results for TC in NHWs. Horizontal lines represent the 4-SNP windows. X-axis direction shows the SNPs genotyped in the regions based on the order of *APOE*, *APOC1*, *HCR1*, *HCR2*, *APOC4* and *APOC2* while the Y-axis shows the–log (global p-value) with red line representing the p-value threshold (P = 0.05).

**Fig 6:**
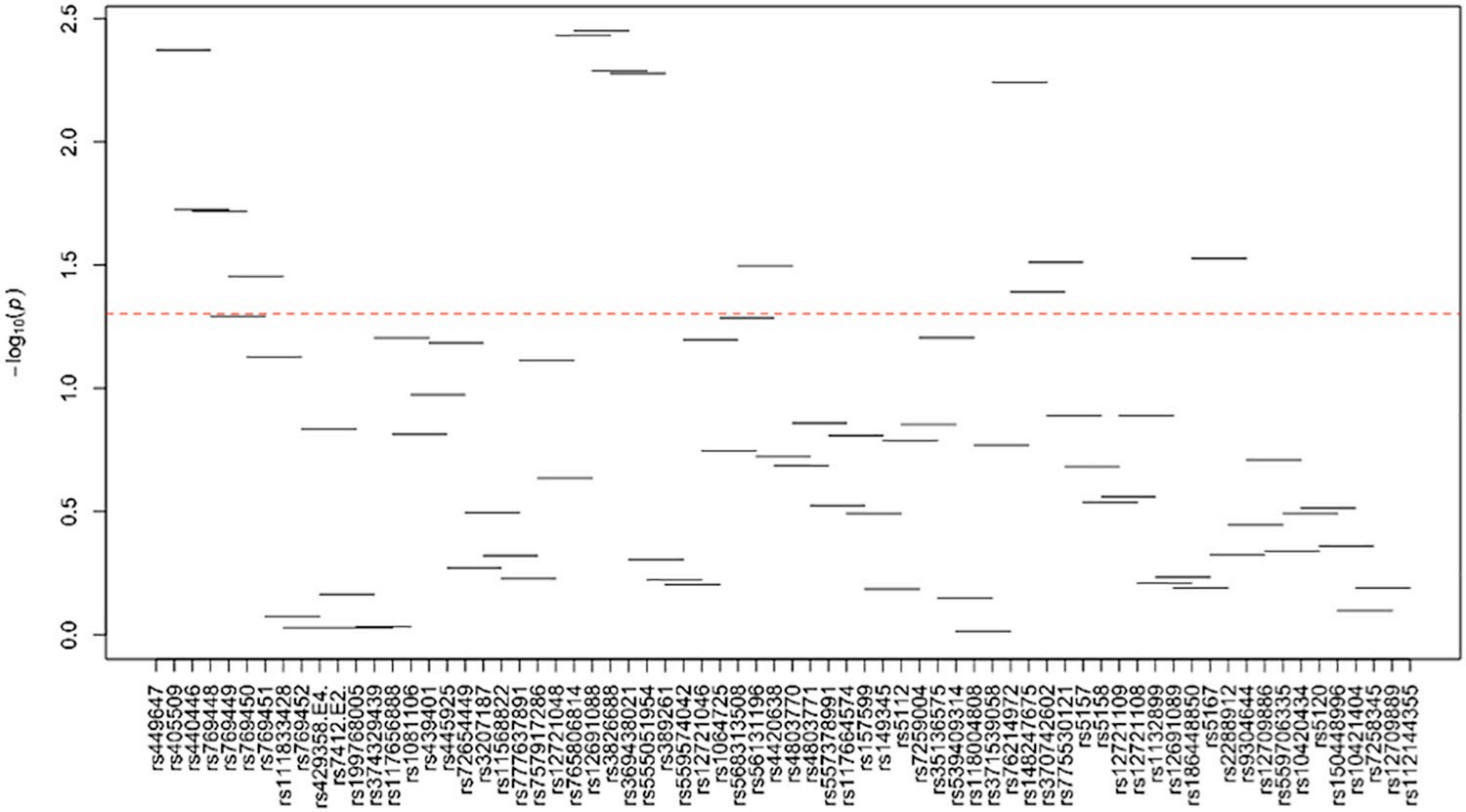
Plots of haplotype-based association analysis results for TG in NHWs. Horizontal lines represent the 4-SNP windows. X-axis direction shows the SNPs genotyped in the regions based on the order of *APOE*, *APOC1*, *HCR1*, *HCR2*, *APOC4* and *APOC2* while the Y-axis shows the–log (global p-value) with red line representing the p-value threshold (P = 0.05).

**Fig 7:**
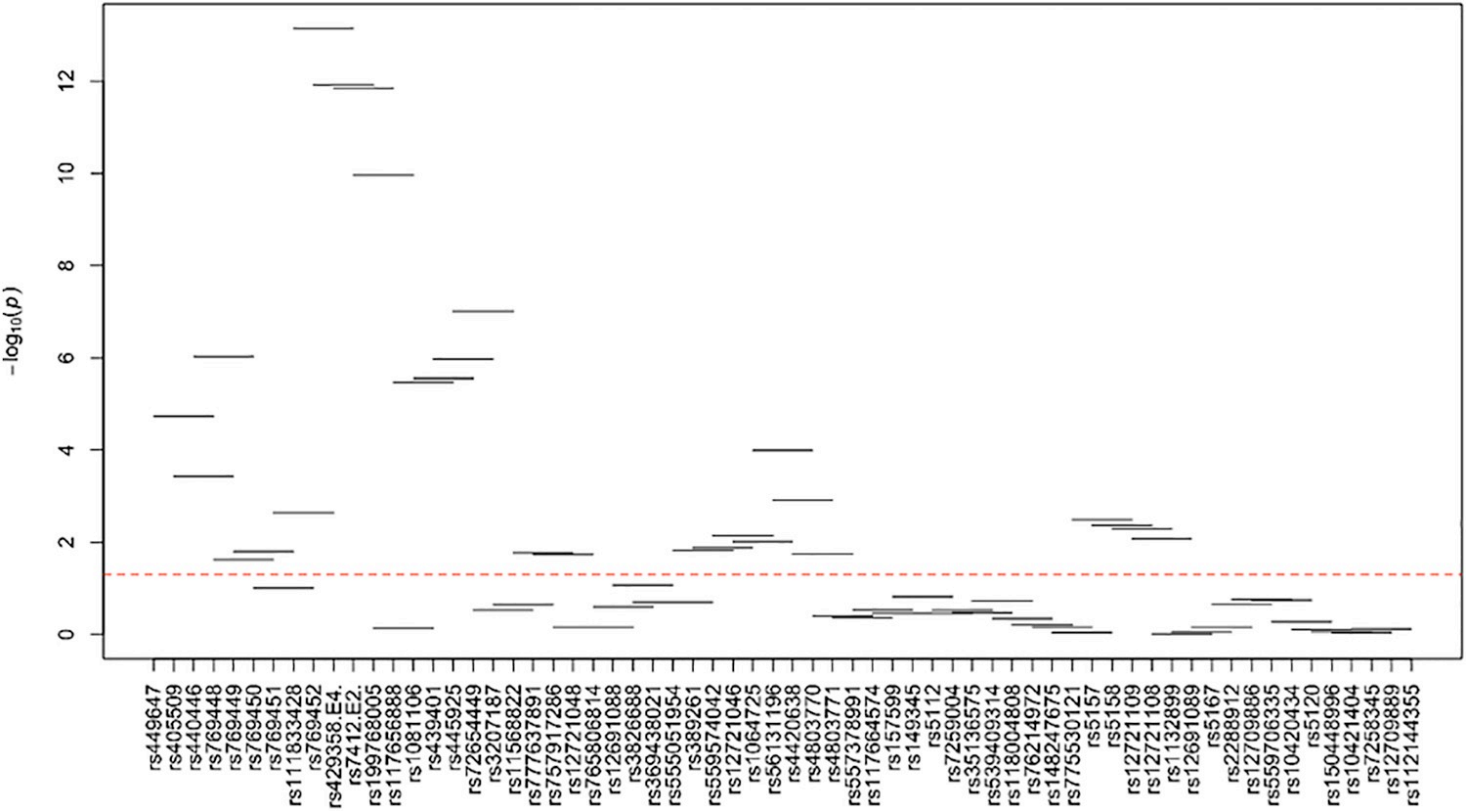
Plots of haplotype-based association analysis results for ApoB in NHWs. Horizontal lines represent the 4-SNP windows. X-axis direction shows the SNPs genotyped in the regions based on the order of *APOE*, *APOC1*, *HCR1*, *HCR2*, *APOC4* and *APOC2* while the Y-axis shows the–log (global p-value) with red line representing the p-value threshold (P = 0.05).

**Fig 8:**
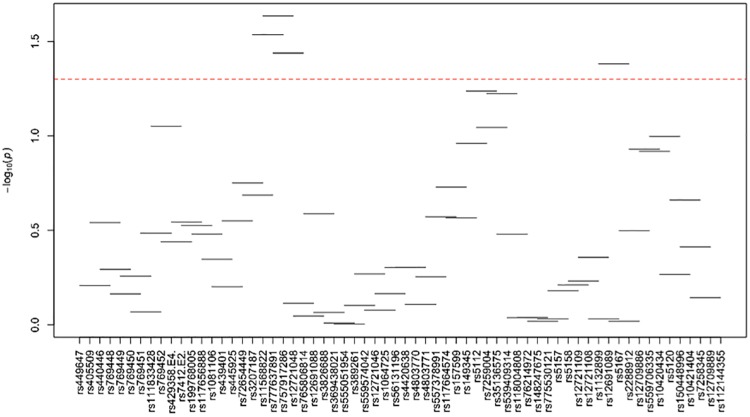
Plots of haplotype-based association analysis results for ApoA1 in NHWs. Horizontal lines represent the 4-SNP windows. X-axis direction shows the SNPs genotyped in the regions based on the order of *APOE*, *APOC1*, *HCR1*, *HCR2*, *APOC4* and *APOC2* while the Y-axis shows the–log (global p-value) with red line representing the p-value threshold (P = 0.05).

**Fig 9:**
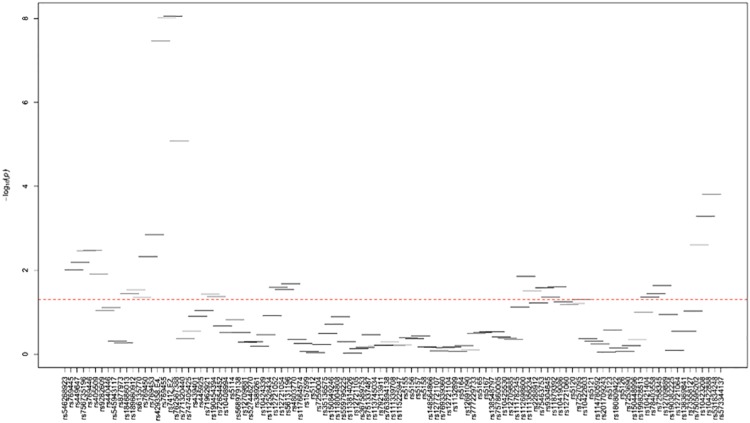
Plots of haplotype-based association analysis results for LDL-C in African blacks. Horizontal lines represent the 4-SNP windows. X-axis shows the SNPs genotyped in the regions based on the order of *APOE*, *APOC1*, *HCR1*, *HCR2*, *APOC4* and *APOC2* while the Y-axis shows the–log (global p-value) with red line representing the p-value threshold (P = 0.05).

**Fig 10:**
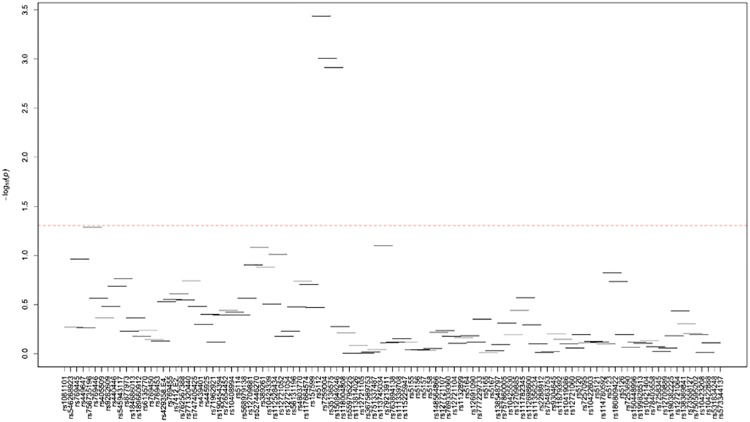
Plots of haplotype-based association analysis results for HDL-C in African blacks. Horizontal lines represent the 4-SNP windows. X-axis shows the SNPs genotyped in the regions based on the order of *APOE*, *APOC1*, *HCR1*, *HCR2*, *APOC4* and *APOC2* while the Y-axis shows the–log (global p-value) with red line representing the p-value threshold (P = 0.05).

**Fig 11:**
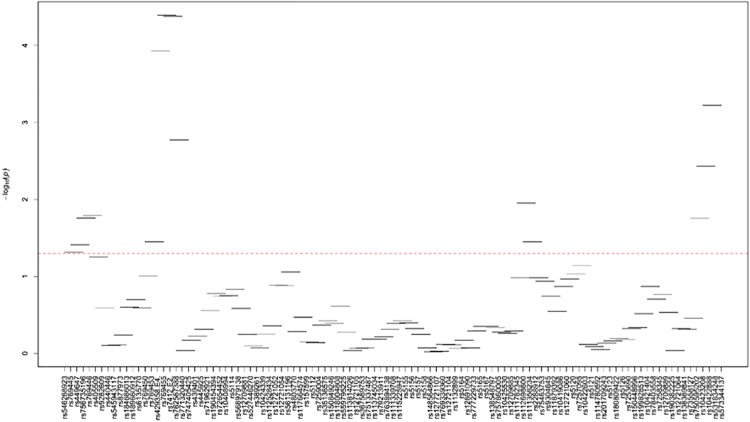
Plots of haplotype-based association analysis results for TC in African blacks. Horizontal lines represent the 4-SNP windows. X-axis shows the SNPs genotyped in the regions based on the order of *APOE*, *APOC1*, *HCR1*, *HCR2*, *APOC4* and *APOC2* while the Y-axis shows the–log (global p-value) with red line representing the p-value threshold (P = 0.05).

**Fig 12:**
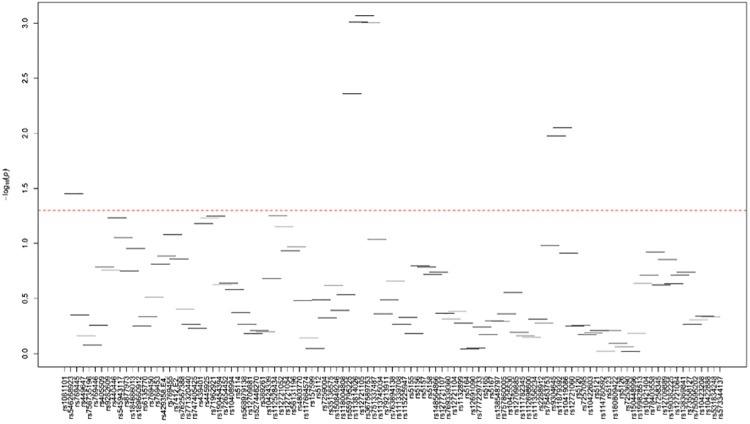
Plots of haplotype-based association analysis results for TG in African blacks. Horizontal lines represent the 4-SNP windows. X-axis shows the SNPs genotyped in the regions based on the order of *APOE*, *APOC1*, *HCR1*, *HCR2*, *APOC4* and *APOC2* while the Y-axis shows the–log (global p-value) with red line representing the p-value threshold (P = 0.05).

**Fig 13:**
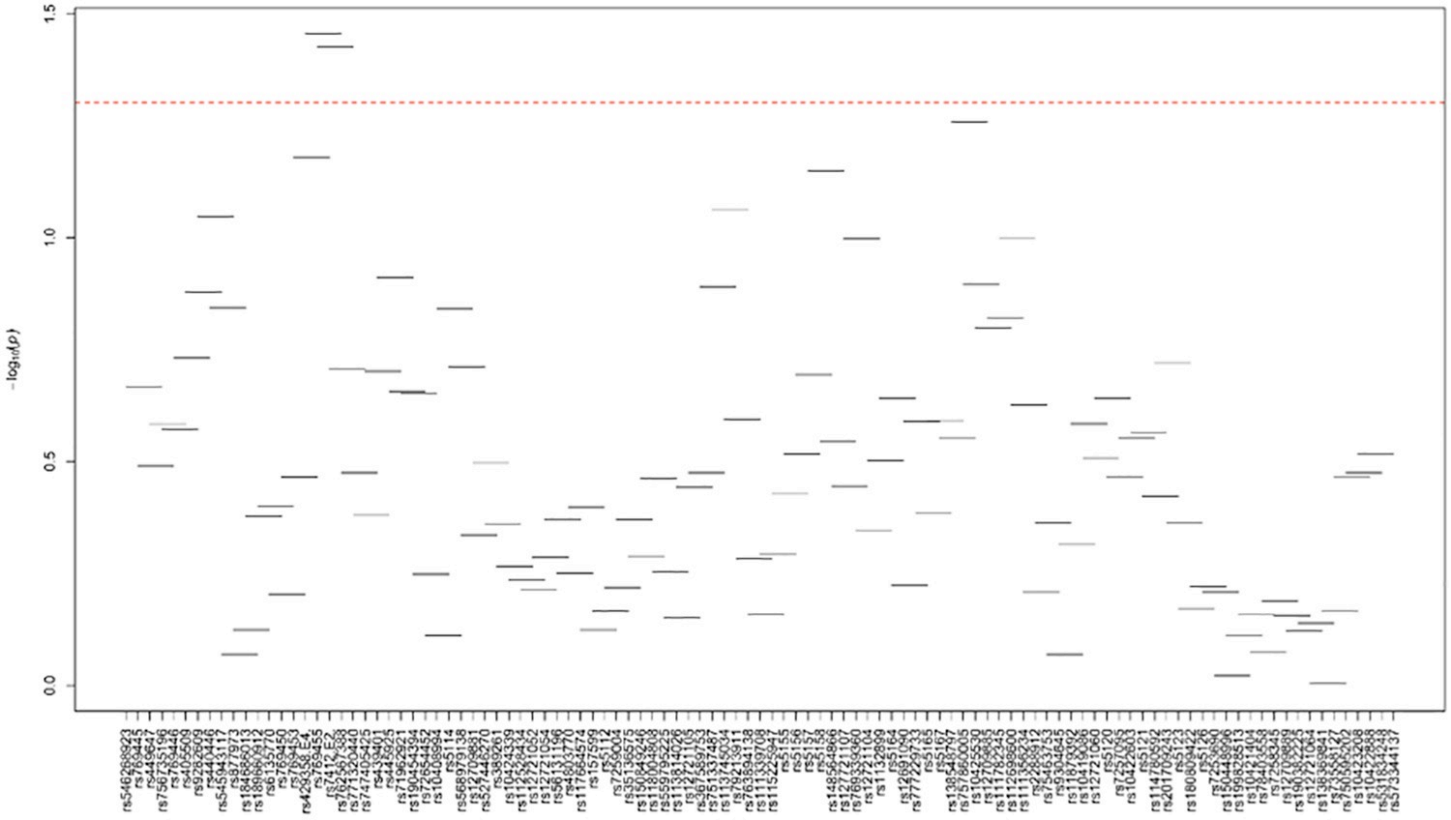
Plots of haplotype-based association analysis results for ApoB in African blacks. Horizontal lines represent the 4-SNP windows. X-axis shows the SNPs genotyped in the regions based on the order of *APOE*, *APOC1*, *HCR1*, *HCR2*, *APOC4* and *APOC2* while the Y-axis shows the–log (global p-value) with red line representing the p-value threshold (P = 0.05).

**Fig 14:**
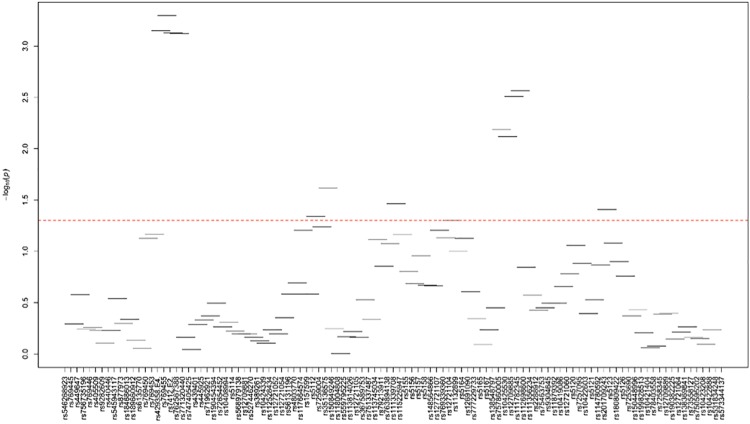
Plots of haplotype-based association analysis results for ApoA1 in African blacks. Horizontal lines represent the 4-SNP windows. X-axis shows the SNPs genotyped in the regions based on the order of *APOE*, *APOC1*, *HCR1*, *HCR2*, *APOC4* and *APOC2* while the Y-axis shows the–log (global p-value) with red line representing the p-value threshold (P = 0.05).

In NHWs, multiple haplotype windows showed significant global P-values for association with LDL-C, TC, and/or apoB levels confirming the single-site association results. The top significant global P-values (1.12E-07≤P≤1.02E-06) for LDL-C included windows 8 through 11 that contained the APOE*2/E*4 alleles. Whereas the significance of a number of relevant windows appeared to be driven by significant variants with MAF>0.01 (see above for single-site analysis results), we also found six significant windows (windows 46–47 and 60–63) that did not include any significant variants with MAF>0.01 and thus suggesting the cumulative effects of other variants. Similarly, multiple haplotype windows showed significant global P-values for association with TC and apoB levels, of which the top ones included windows 8 through 10 (2E-06≤P≤8E-06 and 4.37E-14≤P≤9.72E-13 for TC and apoB, respectively). Six windows (windows 46–47, 60–63) associated with TC and two windows (windows 19–20) associated with apoB were significant without containing any significant variants with MAF>0.01. Moreover, a total of thirteen haplotype windows showed significant global P-values for association with TG levels, of which the top ones included windows 1 and 23–24 (0.00374≤P≤0.00429) that harbored significant variants with MAF>0.01. However, there were five significant TG-associated windows (windows 33, 47–49 and 58) that did not contain any significant variants with MAF>0.01. Additionally, a total of eight and four haplotype windows showed significant global P-values for association with HDL-C (windows 12, 13, 19, 20, 40, 43, 49, 57) and apoA1 (windows 18–20, 52) levels, respectively (2.4E-04≤P≤03.7E-02 and 0.023≤P≤0.043, respectively). Five of HDL-associated windows (windows 13, 19–20, 49, and 57) and three of apoA1-associated windows (windows 18–20) did not harbor any significant variants with MAF>0.01.

In ABs, multiple haplotype windows showed significant global P-values for association with LDL-C and TC levels (8.86E-09≤P≤0.049 and 4E-05≤P≤0.048, respectively) as seen in NHWs, but the effect on apoB levels (0.035≤P≤0.037) was smaller than that observed in NHWs. The top significant LDL-C and TC-associated windows (window 17 for LDL-C and window 18 for TC) harbored the APOE*2/E*4 and APOE*4/rs429358, respectively. Whereas the significance of a number of LDL-C and TC-associated windows appeared to be driven by significant variants with MAF>0.01, we also observed one significant region for LDL-C (windows 95–97) and one significant window for TC (window 5) that did not include any significant variants identified in single-site association analysis (see above). Moreover, we observed three HDL-C-associated (windows 41–43), seven TG-associated (windows 1, 46–49, 79–80) and twelve apoA1-associated (windows 16–19, 41, 43, 54, 63, and 71–74) significant haplotype windows (3.7E-04≤P≤1.2E-03, 8.6E-04≤P≤0.01 and 5E-04≤P≤0.049, respectively). Although all of TG-associated significant windows contained variants that yielded significant associations in single-site analysis, all three HDL-C-associated windows (windows 41–43) and four of apoA1-associated windows (windows 41, 43, 54 and 63) were significant without containing any significant variants identified in single-site association analysis.

## Discussion

In this study we resequenced four genes in the *APOE-C1-C4-C2* cluster at 19q13.32 along with their 5’ & 3’ flanking regions and hepatic control regions (*HCR-1* and *HCR-2*) in selected NHW and AB subjects with extreme HDL-C/TG distribution in order to examine the role of identified common tagSNPs and uncommon/rare variants with plasma lipid levels in two epidemiologically well-characterized samples. Additional common tagSNPs selected from the HapMap database were also included in order to achieve a full coverage of the *APOE-C1-C4-C2* gene region (including intergenic segments) for common variation. Although the established contribution of the *APOE* region is on LDL-related traits, recent GWAS meta-analyses reported multiple SNPs in this gene region to be associated with TG and HDL-C [4,5,8,34,35,36]. Therefore, we considered four major lipid traits (plasma LDL-C, TC, HDL-C, and TG levels) and two correlated apolipoprotiens (apoB and apoA1 levels) for our genotype-phenotype association analyses.

To our knowledge, this is the first study that has considered both common and uncommon/rare variants for genotype-phenotype association analysis of this gene cluster. We compared our sequencing data with a previously sequenced data in the four genes in this cluster in 48 African Americans and 48 white Americans (SeattleSNPs database). We detected all previously reported common variants (MAF≥5%) in SeattleSNPs and NCBI’s dbSNP build 138 for these two ethnic groups (n = 160) and identified 65 new variants in our NHW and AB subjects.

Single-site association analysis of common/uncommon variants (MAF>1%) revealed evidence of association (P<0.05) with at least one lipid trait including the well-known APOE*2/rs7412and APOE*4/rs429358 polymorphisms. The established associations of the APOE*2/E*4 alleles with LDL-C and related traits were replicated in this study such that APOE*2/rs7412 (T) was associated with lower LDL-C, TC, and apoB levels in both ethnic groups, and APOE*4/rs429358(C) was associated with higher LDL-C, TC, and apoB levels in NHWs and with higher LDL-C levels in ABs.

After adjusting for the effects of APOE*2/E*4 alleles, we observed 11 variants in NHWs and 15 variants in ABs that showed independent associations with at least one lipid trait. Eight variants in NHWs and 10 variants in ABs exhibited independent associations with LDL-related traits, including *APOE*/rs440446 that remained significantly associated with LDL-related traits in both populations. Four of the variants that showed independent associations with LDL-related traits in our study (*APOE*/rs440446 and *APOC1P1*/rs5112 in NHWs and *APOE*/rs769455 and *APOE*/rs61357706 in ABs) maintained their significance even after multiple-testing correction. The novel association of other 8 LDL-related traits-associated non-*APOE* variants (*APOC1*/rs3826688, *APOC1*/rs1064725, *APOC2-C4/*rs2288912, *APOC2/*rs5120, *APOC2/*rs10422888, *APOC2-C4*/rs12709885, *APOC4*/rs5157, *APOC2-C4/*rs75463753) should be considered provisional until replicated in independent larger samples. The intronic variant, *APOE*/rs440446, was previously reported to be associated with TG levels and CHD risk in a large Finnish cohort [37] and our current finding of its association with TG, LDL-C and TC levels in NHWs (Table 1) and with apoB levels in Blacks (Table 2) reaffirm the importance of this SNP. In our study, the *APOE*/rs769455 non-synonymous variant (Arg163Cys) detected only in ABs showed association with higher TG and lower LDL-C and TC levels. Previously the same variant was found to be associated with type III hyperlipoproteinemia in five Latin-American family members [38,39] and, in accordance with our finding, Coram et al. (2013) [40] reported its association with TG. *APOE*/rs769455 was in strong LD (r^2^ = 1) with an intronic variant (*APOE*/rs61357706), which also showed population-specific association with lower LDL-C levels in ABs in our study. To the best of our knowledge, this SNP-trait association was not previously reported in any population. Previously, *APOE*/rs405509 was found to be associated with LDL-related traits [41–45], and in agreement, we observed this variant to be associated with LDL-C, TC and TG levels in NHWs (Table 1). Ken-Dror et al. (2010) [45] have reported the association of rs4803770 with LDL-C and apoB, while we found this variant to be associated with LDL-C, TC and TG levels in NHWs.

One intergenic variant, rs7259004, which was previously found to be associated with LDL-C and apoB in US Whites [45], initially showed association with LDL-related traits in NHWs that disappeared after adjusting for the effects of APOE*2/E*4 in our study. Previous studies have also shown the association of *APOC*1/rs11568822 with elevated *APOC1* expression, dysbetalipoproteinemia, and higher risk of CHD and Alzheimer’s disease [46–48]. Moreover, this variant was found to be associated with TG, apoB and HDL-C among APOE*3 carriers [49]. In our study, *APOC1*/rs11568822 was associated with LDL-C and apoA1 levels in AB dependent of APOE*2/E*4. The previously reported association of *APOC4*/rs12721109 with LDL-C [50,51] and apoB [52] was also replicated in our NHW sample, but it disappeared following APOE*2/E*4 adjustment. On the other hand, we observed a novel and independent association of *APOC4*/rs12721109 with TG in NHWs. Previously, *APOE*/rs449647 was found to be associated with lower LDL-C in US Whites but higher LDL-C in African [53]. Although we also observed similar opposite associations of this SNP in our NWH and AB samples, they did not survive after adjusting for APOE*2/E*4.

The association of *APOE* polymorphisms with HDL-C and TG has been inconsistent in individual studies but subsequent GWAS meta-analyses have shown genome-wide significant association of some *APOE* gene cluster SNPs with HDL-C and TG [4,30,35,37]. We also observed multiple APOE*2/E*4-independent associations with HDL-C, TG as well as with apoA1 levels in our study. Independent associations with HDL-C/apoA1 included two variants in NHWs (*APOE*/rs769448 with HDL-C; and *HCR2*/rs35136575 with HDL-C and apoA1), and two variants in ABs (*APOC1*/rs10424339 with HDL-C; and *APOC2-C4*/rs12709885 with apoA1). Although only the latter association (*APOC2-C4*/rs12709885 with apoA1) survived multiple testing correction, our results for *HCR2*/rs35136575 in NHWs was consistent with a previously reported association in Whites [54]. Independent associations with TG included variants that have previously been reported, including *APOE*/rs440446 [37], *APOE/*rs769455 [38,39], *APOC1*/rs12721054 [40], *APOE*/rs405509 [45], rs439401 [45, 50], *APOC4*/rs12721109 [50], *APOE*/rs769450 [55]. One of these variants, rs439401, has shown genome-wide significant associations with both TG and HDL-C [4,34,36,45,50]. In our study, rs439401was associated with TG in NHWs and HDL-C in ABs.

In addition to significant association of variants with MAF>1% with lipid traits, our rare/uncommon variants association analysis has revealed significant association of variants with MAF≤1% with TC in NHWs and TG in ABs, indicating an additional contribution of rare variants to inter-individual variation in plasma lipid levels, as it has previously been shown for some lipid-related genes/loci [56–62]. Moreover, our haplotype-based association analysis helped us to identify a number of significant haplotype windows not harboring individually significant variants (in addition to confirming our single-site analysis results), thus suggesting the cumulative effects of variants with weak effects captured by this approach.

Our study has some limitations. The sample size of the sequencing sample was relatively small and thus we may have missed the identification of some relevant/functional variants. Also, we primarily targeted the relevant genes and their flanking (or known regulatory) regions for sequencing, but not the intergenic regions; the latter were evaluated by tagSNP genotyping only. Moreover, in addition to some initially observed associations that were attenuated by APOE*2/E*4 adjustment, a number of the identified independent associations lost their significance after multiple-testing correction. Nevertheless, we were able to confirm several known associations and also identified some novel associations, awaiting replication in larger independent samples.

In summary, the association of *APOE-C1-C4-C2* gene cluster variation with the evaluated lipid traits confirms the importance of this genomic region in affecting plasma lipid profile in the general population. Our study also supports the involvement of both common/uncommon and rare variants in regulating plasma lipid variation.

## Supporting information

S1 Table Characteristics of sample subsets used in sequencing-based variant discovery step. P-values were calculated based on the original values by using t-test. No covariates were included.
(DOC)

S2 Table Demographic and characteristics of NHWs (n = 623) and ABs (788). TC: Total cholesterol; LDL-C: Low-density lipoprotein cholesterol; HDL-C; High-density lipoprotein cholesterol; TG: Triglycerides; ApoB: Apolipoprotein B; ApoA1: Apolipoprotein A1 *Data available for only 435 NHWs and 766 Blacks.
(DOCX)

S3 Table PCR primers sequences. (**) marked amplicons represent the PCR designed primers using Primer3 software, while the other remaining primers are M13-tag primers based on SeattleSNPs database.
(DOCX)

S4 Table Sequencing results for the *APOE/C1/C4/C2* gene cluster in NHWs (n = 95). Nucleotide position is according to the reference sequence NC_000019.9; Grey-shaded variants represent variants observed in both populations, (****) represents insufficient data. HWE-P: Hardy Weinberg Equillibirium p-value. *Novel variants. **Bold** rs# numbers represent novel refSNP IDs assigned as a result of our dbSNP submission (http://www.ncbi.nlm.nih.gov/SNP/snp_viewTable.cgi?handle5KAMBOH)).
(DOCX)

S5 Table Sequencing results for the *APOE/C1/C4/C2* gene cluster in ABs (n = 95). Nucleotide position is according to the reference sequence NC_000019.9; Grey-shaded variants represent variants observed in both populations; (****) represent insufficient data. HWE-P: Hardy Weinberg Equilibirium p-value; *Novel variants. **Bold** rs numbers represent novel refSNP IDs assigned as a result of our dbSNP submission (http://www.ncbi.nlm.nih.gov/SNP/snp_viewTable.cgi?handle5KAMBOH).
(DOCX)

S6 Table Tagger results for the *APOE/C1/C4/C2* gene cluster variants (MAF≥5%, r^2^ = 0.9) identified by sequencing in NHWs. **Bold** variants represent those genotyped successfully. *Italics* variants represent those failed genotyping or post-genotyping QC.
(DOCX)

S7 Table Tagger results for the *APOE/C1/C4/C2* gene cluster variants (MAF≥5%, r^2^ = 0.9) identified by sequencing in ABs. **Bold** variants represent those genotyped successfully. *Italics* variants represent those failed genotyping or post-genotyping QC.
(DOCX)

S8 Table Tagger results for HapMap SNPs (MAF≥0.048, r^2^ = 0.9) covering the region of interest at 19q13.32 in CEU population. Underlined variants represent those located within the sequenced regions. Bold variants represent those genotyped successfully. *Italics* variants represent those failed genotyping or post-genotyping QC.
(DOCX)

S9 Table Tagger results for HapMap SNPs (MAF≥0.048, r^2^ = 0.9) covering the region of interest at 19q13.32 in YRI population. Underlined variants represent those located within the sequenced regions. **Bold** variants represent those genotyped successfully. *Italics* variants represent those failed genotyping or post-genotyping QC.
(DOCX)

S10 Table Features of 70 QC-passed genotyped variants in NHWs (n = 623). HWE-P: Hardy Weinberg equilibrium, MAF: minor allele frequency, Position: chromosomal position corresponding to Chip bioinformatics database (NC_000019.9). RegulomeDB scores were generated by using http://regulome.stanford.edu/. Scores represent; “1a- eQTL + TF binding + matched TF motif + matched DNase Footprint + DNase peak; 1b- eQTL + TF binding + any motif + DNase Footprint + DNase peak; 1c- eQTL + TF binding + matched TF motif + DNase peak; 1d- eQTL + TF binding + any motif + DNase peak; 1e- eQTL + TF binding + matched TF motif; 1f- eQTL + TF binding / DNase peak; 2a- TF binding + matched TF motif + matched DNase Footprint + DNase peak; 2b- TF binding + any motif + DNase Footprint + DNase peak; 2c- TF binding + matched TF motif + DNase peak; 3a- TF binding + any motif + DNase peak; 3b- TF binding + matched TF motif; 4- TF binding + DNase peak; 5-TF binding or DNase peak; 6-other.” Selection criteria: 1) Common tagSNPs identified by Tagger analyses of sequencing data (MAF≥0.05, r^2^ = 0.9); 2) Rare/uncommon variants identified by sequencing (MAF<5%); 3) Additional common SNPs selected from public resources.
(DOCX)

S11 Table Features of 108 QC-passed genotyped variants in ABs (n = 788). HWE-P: Hardy Weinberg equilibrium, MAF: minor allele frequency, Position: chromosomal position corresponding to Chip bioinformatics database (NC_000019.9). RegulomeDB scores were generated by using http://regulome.stanford.edu/. Scores represents; 1a- eQTL + TF binding + matched TF motif + matched DNase Footprint + DNase peak; 1b- eQTL + TF binding + any motif + DNase Footprint + DNase peak; 1c- eQTL + TF binding + matched TF motif + DNase peak; 1d- eQTL + TF binding + any motif + DNase peak; 1e- eQTL + TF binding + matched TF motif; 1f- eQTL + TF binding / DNase peak; 2a- TF binding + matched TF motif + matched DNase Footprint + DNase peak; 2b- TF binding + any motif + DNase Footprint + DNase peak; 2c- TF binding + matched TF motif + DNase peak; 3a- TF binding + any motif + DNase peak; 3b- TF binding + matched TF motif; 4- TF binding + DNase peak; 5-TF binding or DNase peak; 6-other. Selection criteria: 1) Common tagSNPs identified by Tagger analyses of sequencing data (MAF≥0.05, r^2^ = 0.9); 2) Rare/uncommon variants identified by sequencing (MAF<5%); 3) Additional common SNPs selected from public resources.
(DOCX)

S12 Table Single-site association analysis results for LDL-C levels in NHWs (n = 623). MAF is the minor allele frequency; GT is genotype; GT count is the number of individuals in each genotype group; GT_SD is standard deviation of the lipid trait in each genotype group;. *Adjusted for relevant covariates, **Adjusted for APOE*2/E*4 SNPs in addition to the covariates.
(DOCX)

S13 Table Single-site association analysis results for TC in NHWs. MAF is the minor allele frequency; GT is genotype; GT count is the number of individuals in each genotype group; GT_SD is standard deviation of lipid traits mean in each genotype group; *Adjusted for relevant covariates, **Adjusted for APOE*2/E*4 SNPs in addition to the covariates.
(DOCX)

S14 Table Single-site association analysis results for ApoB in NHWs. MAF is the minor allele frequency; GT is genotype; GT count is the number of individuals in each genotype group; GT_SD is standard deviation of lipid traits mean in each genotype group; *Adjusted for relevant covariates, **Adjusted for APOE*2/E*4 SNPs in addition to the covariates. Four rare variants were excluded due to missing data.
(DOCX)

S15 Table Single-site association analysis results for HDL-C in NHWs. MAF is the minor allele frequency; GT is genotype; GT count is the number of individuals in each genotype group; GT_SD is standard deviation of lipid traits mean in each genotype group; *Adjusted for relevant covariates, **Adjusted for APOE*2/E*4 SNPs in addition to the covariates. APOC1p703/rs3207187 was excluded due to missing data.
(DOCX)

S16 Table Single-site association analysis results for ApoA1 in NHWs. MAF is the minor allele frequency; GT is genotype; GT count is the number of individuals in each genotype group; GT_SD is standard deviation of lipid traits mean in each genotype group; *Adjusted for relevant covariates, **Adjusted for APOE*2/E*4 SNPs in addition to the covariates. Four rare variants were excluded due to missing data.
(DOCX)

S17 Table Single-site association analysis results for TG in NHWs. MAF is the minor allele frequency; GT is genotype; GT count is the number of individuals in each genotype group; GT_SD is standard deviation of lipid traits mean in each genotype group; *Adjusted for relevant covariates, **Adjusted for APOE*2/E*4 SNPs in addition to the covariates.
(DOCX)

S18 Table Single-site association analysis results for LDL-C in ABs. MAF is the minor allele frequency; GT is genotype; GT count is the number of individuals in each genotype group; GT_SD is standard deviation of lipid traits mean in each genotype group; *Adjusted for relevant covariates, **Adjusted for APOE*2/E*4 SNPs in addition to the covariates. APOC2p5771 is excluded due to missing data.
(DOCX)

S19 Table Single-site association analysis results for TC in ABs. MAF is the minor allele frequency; GT is genotype; GT count is the number of individuals in each genotype group; GT_SD is standard deviation of lipid traits mean in each genotype group; *Adjusted for relevant covariates, **Adjusted for APOE*2/E*4 SNPs in addition to the covariates. APOC2p5771 is excluded due to missing data.
(DOCX)

S20 Table Single-site association analysis results for ApoB in ABs. MAF is the minor allele frequency; GT is genotype; GT count is the number of individuals in each genotype group; GT_SD is standard deviation of lipid traits mean in each genotype group; *Adjusted for relevant covariates, **Adjusted for APOE*2/E*4 SNPs in addition to the covariates. APOC2p5771 is excluded due to missing data.
(DOCX)

S21 Table Single-site association analysis results for HDL-C in ABs. MAF is the minor allele frequency; GT is genotype; GT count is the number of individuals in each genotype group; GT_SD is standard deviation of lipid traits mean in each genotype group; *Adjusted for relevant covariates, **Adjusted for APOE*2/E*4 SNPs in addition to the covariates. APOC2p4118/rs201709243 is excluded due to missing data.
(DOCX)

S22 Table Single-site association analysis results for ApoA1 in ABs. MAF is the minor allele frequency; GT is genotype; GT count is the number of individuals in each genotype group; GT_SD is standard deviation of lipid traits mean in each genotype group; *Adjusted for relevant covariates, **Adjusted for APOE*2/E*4 SNPs in addition to the covariates. APOC2p4118/rs201709243 is excluded due to missing data.
(DOCX)

S23 Table Single-site association analysis results for TG in ABs. MAF is the minor allele frequency; GT is genotype; GT count is the number of individuals in each genotype group; GT_SD is standard deviation of lipid traits mean in each genotype group; *Adjusted for relevant covariates, **Adjusted for APOE*2/E*4 SNPs in addition to the covariates. APOC2p4118/rs201709243 is excluded due to missing data.
(DOCX)

S24 Table Haplotype summary of significant windows with LDL-C in NHWs. hap.freq: haplotype frequency; coef: coefficient; se: standard error; t.stat: test statistic; p-val: haplotype p-value.
(DOCX)

S25 Table Haplotype summary of significant windows with TC in NHWs. hap.freq: haplotype frequency; coef: coefficient; se: standard error; t.stat: test statistic; p-val: haplotype p-value.
(DOCX)

S26 Table Haplotype summary of significant windows with ApoB in NHWs. hap.freq: haplotype frequency; coef: coefficient; se: standard error; t.stat: test statistic; p-val: haplotype p-value.
(DOCX)

S27 Table Haplotype summary of significant windows with HDL-C in NHWs. hap.freq: haplotype frequency; coef: coefficient; se: standard error; t.stat: test statistic; p-val: haplotype p-value.
(DOCX)

S28 Table Haplotype summary of significant windows with ApoA1 in NHWs. hap.freq: haplotype frequency; coef: coefficient; se: standard error; t.stat: test statistic; p-val: haplotype p-value.
(DOCX)

S29 Table Haplotype summary of significant windows with TG in NHWs. hap.freq: haplotype frequency; coef: coefficient; se: standard error; t.stat: test statistic; p-val: haplotype p-value.
(DOCX)

S30 Table Haplotype summary of significant windows with LDL-C in ABs. hap.freq: haplotype frequency; coef: coefficient; se: standard error; t.stat: test statistic; p-val: haplotype p-value; ^a^Box-Cox transformed data.
(DOCX)

S31 Table Haplotype summary of significant windows with TC in ABs. hap.freq: haplotype frequency; coef: coefficient; se: standard error; t.stat: test statistic; p-val: haplotype p-value; ^a^Box-Cox transformed data.
(DOCX)

S32 Table Haplotype summary of significant windows with ApoB in ABs. hap.freq: haplotype frequency; coef: coefficient; se: standard error; t.stat: test statistic; p-val: haplotype p-value; ^a^Box-Cox transformed data.
(DOCX)

S33 Table Haplotype summary of significant windows with HDL-C in ABs. hap.freq: haplotype frequency; coef: coefficient; se: standard error; t.stat: test statistic; p-val: haplotype p-value; ^a^Box-Cox transformed data.
(DOCX)

S34 Table Haplotype summary of significant windows with ApoA1 in ABs. hap.freq: haplotype frequency; coef: coefficient; se: standard error; t.stat: test statistic; p-val: haplotype p-value; ^a^Box-Cox transformed data.
(DOCX)

S35 Table Haplotype summary of significant windows with TG in ABs. hap.freq: haplotype frequency; coef: coefficient; se: standard error; t.stat: test statistic; p-val: haplotype p-value; ^a^Box-Cox transformed data.
(DOCX)
