# Supplementary material for: Apolipoprotein E-C1-C4-C2 gene cluster region and inter-individual variation in plasma lipoprotein levels: a comprehensive genetic association study in two ethnic groups
Source: PLoS One. 2019 Mar 26;14(3):e0214060. doi: 10.1371/journal.pone.0214060 (PMC6435132; doi:10.1371/journal.pone.0214060)
Supplement: S1 Table — P-values were calculated based on the original values by using t-test. No covariates were included. (DOC) [file pone.0214060.s001.doc]

S1 Table. Characteristics of sample subsets used in sequencing-based variant discovery step.

|  | **NHWs (n=95)** | | | **African blacks (n=95)** | | |
| --- | --- | --- | --- | --- | --- | --- |
|  | **High HDL-C/low TG (n=47)** | **Low HDL–C/high TG (n=48)** | **P** | **High HDL-C/low TG (n=48)** | **Low HDL-C/high TG (n=47)** | **P** |
| **Sex (M/F)** | 24/23 | 24/24 | 1 | 24/24 | 23/24 | 1 |
| **Age (years)** | 55.45 ± 9.80 | 53.03 ± 10.54 | 0.25 | 41.29 ± 8.72 | 40.87 ± 7.12 | 0.80 |
| **BMI (kg/m2)** | 23.17 ± 3.17 | 27.35 ± 3.90 | 1.2E-07 | 22.06 ± 4.70 | 23.91 ± 5.51 | 0.08 |
| **TC (mg/dl)** | 227.34 ± 51.76 | 208.81± 44.65 | 0.07 | 201 ± 39.68 | 141.68 ± 31.03 | 2.4E-12 |
| **LDL-C(mg/dl)** | 126.84 ± 46.95 | 125.54 ± 54.97 | 0.90 | 112.55 ± 39.75 | 95.04 ± 28.28 | 0.02 |
| **HDL-C (mg/dl)** | 77.68 ± 13.32 | 31.81 ± 4.37 | 2.2E-16 | 76.05 ± 7.53 | 25.51 ± 5.66 | 2.2E-16 |
| **TG (mg/dl)** | 114.09 ± 60.88 | 240.21 ± 153.22 | 1.7E-06 | 61.98 ± 19.85 | 95.79 ± 73.21 | 0.004 |
| **ApoB(mg/dl)** | 87.88 ± 25.49 | 89.61± 25.18 | 0.80 | 66.00 ± 20.22 | 69.64 ± 21.46 | 0.40 |
| **ApoA1(mg/dl)** | 174.08 ± 3.57 | 130.20 ± 2.78 | 1.4E-06 | 166.04 ± 28.19 | 103.84 ± 27.23 | 2.2E-16 |
| P-values were calculated based on the original values by using t-test. No covariates were included. | | | | | | |
