# Supplementary material for: Apolipoprotein E-C1-C4-C2 gene cluster region and inter-individual variation in plasma lipoprotein levels: a comprehensive genetic association study in two ethnic groups
Source: PLoS One. 2019 Mar 26;14(3):e0214060. doi: 10.1371/journal.pone.0214060 (PMC6435132; doi:10.1371/journal.pone.0214060)
Supplement: S2 Table — TC: Total cholesterol; LDL-C: Low-density lipoprotein cholesterol; HDL-C; High-density lipoprotein cholesterol; TG: Triglycerides; ApoB: Apolipoprotein B; ApoA1: Apolipoprotein A1 *Data available for only 435 NHWs and 766 Blacks. (DOCX) [file pone.0214060.s002.docx]

**Table S2. Demographic and characteristics of NHWs (n=623) and ABs (788).**

| **Variable** | **NHWs (n=623)**  **Mean±sd or percentage** | **African Blacks (n=788)**  **Mean±sd or percentage** |
| --- | --- | --- |
| **Males/Females (%)** | 47.35/52.64 | 62.81/37.18 |
| **Age (Yrs)** | 52.83 ± 11.41 | 40.95 ± 8.39 |
| **BMI (kg/m^2^)** | 25.5 1± 4.06 | 22.87 ± 4.04 |
| **LDL-C (mg/dl)** | 136.9 ± 40.80 | 109.25 ± 34.40 |
| **HDL-C (mg/dl)** | 50.76 ± 14.35 | 47.88 ± 12.87 |
| **TG (mg/dl)** | 142.72 ± 93.49 | 72.96 ± 39.32 |
| **TC (mg/dl)** | 217.0 ± 43.5 | 172.01 ± 38.47 |
| ***ApoB (mg/dl)** | 87.72 ± 24.27 | 66.98 ± 22.19 |
| ***ApoA1 (mg/dl)** | 149.62 ± 33.33 | 137.03 ± 28.46 |
| TC: Total cholesterol; LDL-C: Low-density lipoprotein cholesterol; HDL-C; High-density lipoprotein cholesterol; TG: Triglycerides; ApoB: Apolipoprotein B; ApoA1: Apolipoprotein A1  *Data available for only 435 NHWs and 766 Blacks. | | |
