# Supplementary material for: Apolipoprotein E-C1-C4-C2 gene cluster region and inter-individual variation in plasma lipoprotein levels: a comprehensive genetic association study in two ethnic groups
Source: PLoS One. 2019 Mar 26;14(3):e0214060. doi: 10.1371/journal.pone.0214060 (PMC6435132; doi:10.1371/journal.pone.0214060)
Supplement: S3 Table — (**) marked amplicons represent the PCR designed primers using Primer3 software, while the other remaining primers are M13-tag primers based on SeattleSNPs database. (DOCX) [file pone.0214060.s003.docx]

S3 Table. PCR primers used for sequencing

| ***APOE* Gene** | | | |
| --- | --- | --- | --- |
| **Amplicon** | **Amplified Region** | **Forward Primer Sequence (5’-3’)** | **Reverse Primer Sequence (3’-5’)** |
| #1 | 34-855 | CTTGATGCTCAGAGAGGACAAG | GGCATAGAGGTCTTTTGACCA |
| #2 | 790-1515 | GGTCAGGAAAGGAGGACTCT | GTCCCAGTCTCGCATTCCTC |
| #3 | 1445-2053 | GGCAGCGACACGGTAGCTAG | AACCGAGGCCCAGAGAGCGT |
| #4 | 1930-2880 | GTTGCTGGTCACATTCCTGG | GAGTCGGTTTAATCACTTG |
| #5 | 2560-3194 | AGCCCTGCCTGGGGCACAC | GGACACTCACCTCAGTTCCT |
| #6 | 3100-3795 | GAGTGGCAGAGCGGCCAGCG | CCTTCAACTCCTTCATGGTCTC |
| #7 | 3625-4360 | CTAGCTCCTTCTTCGTCTCTG | GCTCGAACCAGCTCTTGAGG |
| #8 | 4250-4780 | GCCAGCCGCTACAGGAGCG | CCAGCTACTGAGGCAGCAG |
| #9 | 4775-5430 | GTGTGTATCTTTCTCTCTGCC | GGCAGGCCGCTCGGAGCCCAT |
| ***APOC1* Gene** | | | |
| **AMPLICON** | **Amplified Region** | **FORWARD PRIMER SEQUENCE (5’-3’)** | **RESVERSE PRIMER SEQUENCE (3’-5’)** |
| #10* | 380-947 | CCAATTTCTGCCTCCAAAGA | GGGCAGGTTGATGTTGATCT |
| #11 | 845-1610 | TCAGGAAGATTGAGAGGTGAGAG | GGAGGAATACTGGAGTGACCTG |
| #12* | 1480-1950 | AGGTTCTCCCAGGCTCAGTC | ATTGGAACCCCCTTAGGTTG |
| #13 | 2025-2325 | ATCTGCGCAGGAGAGCACTAGCA | GATGGGCAGGGGTTCAAAATTTG |
| #14* | 1960-2400 | GCCATTCCTCCCCATTCTAA | GGCATGATCTCGGCCTAC |
| #15* | 2025-2600 | CTCTATCCATCCCGGTATCC | GCATCTTGGCAGAAAGTTCA |
| #16 | 2545-3150 | ATTTTGAACCCCTGCCCATCTTC | TTACCTCACAAAACCATCTTTTT |
| #17* | 2815-3200 | CAAGTCCTGGAGAGGCTGAC | GAGACAGGATCTCGCCATGT |
| #18* | 2976-3435 | GGCTGATTGCCTGAGGTC | CCTGTAGGCCTGCTCCTTTA |
| #19 | 3915-3944 | AAGATGGTTTTGTGAGGTAATGA | ATCTCTTGCTACCAATGCCTTCC |
| #20* | 3435-3850 | CTGGAGTAGCACCCCTTCCT | GAATGCAGTGGCACAAACAT |
| #21 | 4975-5650 | AGGCATTGGTAGCAAGAGATGGC | CTGGAAGCCCCTACCCTCTCCC |
| #22* | 5600-6260 | GAGGACCTGAAGGGTGACAT | GATTGCTTAAGCCCAGGAGT |
| ***HCR-1*** | | | |
| **Amplicon** | **Amplified Region** | **Forward Primer Sequence (5’-3’)** | **Resverse Primer Sequence (3’-5’)** |
| #23* | 18-838 | GAGGGGCTAGAGACACCAGA | AGGCTGAGGCTGACCAACAT |
| ***HCR-2*** | | | |
| **Amplicon** | **Amplified Region** | **Forward Primer Sequence (5’-3’)** | **Resverse Primer Sequence (3’-5’)** |
| #24* | 22-871 | CCAGAAGATTAGCAGGGAGGA | GGTGGATCACAAGGTCAAGAG |
| ***APOC4*** | | | |
| **Amplicon** | **Amplified Region** | **Forward Primer Sequence (5’-3’)** | **Resverse Primer Sequence (3’-5’)** |
| #25 | 25-655 | GAGACGGAGTCTTGCTCTTTCGC | TCAGTTTCCTCCTCCATAAAGTG |
| #25 | 489-1245 | ATTACAGGCACGCATCACTACTT | CACACAGATGATCCCAGTTTGTA |
| #26 | 1108-1842 | GTGAGAAGAAGTGGGTGGAGG | GTCAGGATATGGAGACCATCCTG |
| #27* | 1869-2147 | CCAGGATGGTCTCCATATCC | GTGCCTGGCCCTGTATTAAA |
| #28 | 2165-2928 | ATTCTAGATCAGCATTATCCAGTA | TCTTCACCTTGTGTCAGTAGTCC |
| #29 | 2819-3755 | GTCCACAGAGGTAGCTCAGACAG | GCTCCTCTCTGTGACCTAGGAGT |
| #30 | 3537-4462 | AAAGCTAAAGATGAGTCGCTGG | GGAGTCAGAGCTTGTAGGAGACA |
| #31* | 4432-5022 | CTTCAAGGCGTGTCAGTTTC | ATTTGTGGAGTGTGGTGGTG |
| ***APOC2*** | | | |
| **Amplicon** | **Amplified Region** | **Forward Primer Sequence (5’-3’)** | **Resverse Primer Sequence (3’-5’)** |
| #32* | 62-585 | TTAGCGGTCAGGGGACACAT | CCCTCAGGTGGTCGTCATAG |
| #33* | 478-997 | CTAGGTCTGGGAGGAGTGGA | CAAGCCCACAATAGGGTGTT |
| #34 | 685-1555 | AAGACAGCCTCTTGAAGAAGACC | GTCATGGTTCCAACACGGGCTT |
| #35 | 1455-2170 | TGGGTCTCAACCACTATAAAGCC | GCTAGAGTACAGTGGCACGATCT |
| #36 | 1840-2734 | GAACTGTAGACTATTTGAGCTTCT | CTAAGTCAGACCTCATGTCCCTG |
| #37* | 2665-3338 | TGCCGTACTTCCTCATCTCC | AGCTGGAATCACAAGCACCT |
| #38 | 3165-3909 | TCACTTGAGGTTAGGAGTTCAAGA | TACCAGGAGGACAAGAAACAGAG |
| #39* | 3810-4535 | GAGCACACACAGAGCAGGAT | GTACAAGTCCCTGGGGAGAA |
| #40* | 4390-4960 | GCCCGCTGTAGATGAGAAAC | GAGAGAAAGCCGGACTTCAA |
| #41* | 4860-5430 | AGGACTCAAGGTGCCAAGAT | CTTGCTTTGTTTGTGCGTTT |
| #42 | 5350-6059 | GCCAAAATCCAGCCTTCTCATTTGTA | CACGGGACCATTATACACTGATT |
| #43* | 5920-6370 | GCTCAAGAGATCCTCCCATC | CTATCTCCGCCTCCAGGGTA |
| (*) marked amplicons represent the PCR designed primers using Primer3 software, while the other remaining primers are M13-tag primers based on SeattleSNPs database. | | | |
