# Supplementary material for: Apolipoprotein E-C1-C4-C2 gene cluster region and inter-individual variation in plasma lipoprotein levels: a comprehensive genetic association study in two ethnic groups
Source: PLoS One. 2019 Mar 26;14(3):e0214060. doi: 10.1371/journal.pone.0214060 (PMC6435132; doi:10.1371/journal.pone.0214060)
Supplement: S4 Table — Nucleotide position is according to the reference sequence NC_000019.9; Grey-shaded variants represent variants observed in both populations, (****) represents insufficient data. HWE-P: Hardy Weinberg Equillibirium p-value. *Novel variants. Bold rs# numbers represent novel refSNP IDs assigned as a result of our dbSNP submission (http://www.ncbi.nlm.nih.gov/SNP/snp_viewTable.cgi?handle5KAMBOH)). (DOCX) [file pone.0214060.s004.docx]

S4 Table. Sequencing results for the *APOE/C1/C4/C2* gene cluster in NHWs (n=95)

| **Variant Name** | **Alleles** | **RefSNP ID** | **Location** | **Amino acid change** | **MAF in the total sample** | **MAF in the High HDL-C/low TG group** | **MAF in the Low HCL-C/high TG group** | **HWE-P** | **Call rate (%)** |
| --- | --- | --- | --- | --- | --- | --- | --- | --- | --- |
| APOE-560 | A>T | rs449647 | 5'flanking |  | 0.126 | 0.085 | 0.167 | 0.379 | 100 |
| APOE-624 | T>C | rs769446 | 5'flanking |  | 0.075 | 0.111 | 0.042 | 1.000 | 97.9 |
| APOE-832 | G>T | rs405509 | 5'flanking |  | 0.484 | 0.479 | 0.490 | 1.000 | 100 |
| APOE-1163 | G>C | rs440446 | Intron 1 |  | 0.358 | 0.340 | 0.375 | 0.108 | 100 |
| APOE-1575 | C>T | rs769448 | Intron 1 |  | 0.021 | 0.043 | 0.000 | 1.000 | 100 |
| APOE-1998 | G>A | rs769449 | Intron 2 |  | 0.126 | 0.128 | 0.125 | 1.000 | 100 |
| ***APOE-2294** | C>T | **rs572713679** | Intron 2 |  | 0.005 | 0.011 | 0.000 | 1.000 | 100 |
| APOE-2440 | G>A | rs769450 | Intron 2 |  | 0.400 | 0.404 | 0.396 | 0.855 | 100 |
| APOE-2907 | T>G | rs769451 | Intron 2 |  | 0.005 | 0.000 | 0.010 | 1.000 | 100 |
| APOE-3038 | G>A | rs111833428 | Exon 3 | Ala23Ala | 0.005 | 0.011 | 0.000 | 1.000 | 100 |
| APOE-3937 | T>C | rs429358 | Exon 4 | Cys130Arg | 0.168 | 0.160 | 0.177 | 1.000 | 100 |
| APOE-4075 | C>T | rs7412 | Exon 4 | Arg176Cys | 0.063 | 0.074 | 0.052 | 1.000 | 100 |
| APOE-4310 | T>A | rs199768005 | Exon 4 | Val254Glu | 0.005 | 0.011 | 0.000 | 1.000 | 100 |
| APOE-4528 | C>T | rs374329439 | 3' UTR |  | 0.005 | 0.011 | 0.000 | 1.000 | 100 |
| APOE-4737 | C>G | rs117656888 | 3'flanking |  | 0.011 | 0.021 | 0.000 | 1.000 | 100 |
| APOE-4951 | A>C | rs1081105 | 3'flanking |  | 0.042 | 0.032 | 0.052 | 1.000 | 100 |
| APOE-5229 | G>T | rs1065853 | 3'flanking |  | 0.064 | 0.076 | 0.052 | 1.000 | 98.9 |
| APOE-5230 | INDEL | rs55729972 | 3'flanking |  | **** | **** | **** | **** | **** |
| APOE-5361 | T>C | rs1081106 | 3'flanking |  | 0.105 | 0.117 | 0.094 | 1.000 | 100 |
| APOC1-607 | G>A | rs72654447 | 5'flanking |  | 0.005 | 0.011 | 0.000 | 1.000 | 100 |
| APOC1-655 | A>T | rs66807996 | 5'flanking |  | 0.016 | 0.021 | 0.011 | 1.000 | 98.9 |
| APOC1-659 | G>C | rs72654448 | 5'flanking |  | 0.005 | 0.000 | 0.010 | 1.000 | 100 |
| APOC1-698 | C>A | rs72654449 | 5'flanking |  | 0.011 | 0.011 | 0.010 | 1.000 | 100 |
| APOC1-703 | C>T | rs320**7187** | 5'flanking |  | 0.005 | 0.011 | 0.000 | 1.000 | 100 |
| APOC1-720_721 | ins4 | rs11568822 | 5'flanking |  | 0.226 | 0.223 | 0.229 | 0.894 | 100 |
| *****APOC1-1170 | G>A | **rs777637891** | Intron 1 |  | 0.005 | 0.011 | 0.000 | 1.000 | 100 |
| APOC1-1276 | C>G | rs72654453 | Exon 2 | Ile3Met | 0.011 | 0.021 | 0.000 | 1.000 | 100 |
| *****APOC1-1294 | T>G | **rs757917286** | Intron 2 |  | 0.005 | 0.011 | 0.000 | 1.000 | 100 |
| APOC1-1317 | G>A | rs12721048 | Intron 2 |  | 0.011 | 0.021 | 0.000 | 1.000 | 100 |
| *****APOC1-1422 | G>A | **rs765806814** | Intron 2 |  | 0.005 | 0.011 | 0.000 | 1.000 | 100 |
| APOC1-1566 | G>A | rs12691088 | Intron 2 |  | 0.026 | 0.011 | 0.042 | 1.000 | 100 |
| *****APOC1-1669 | T>C | **rs749865057** | Intron 2 |  | 0.005 | 0.011 | 0.000 | 1.000 | 100 |
| APOC1-1870 | T>C | rs5117 | Intron 2 |  | 0.229 | 0.228 | 0.229 | 0.8701 | 98.9 |
| APOC1-2041 | C>T | rs3826688 | Intron 2 |  | 0.370 | 0.337 | 0.402 | 0.674 | 96.8 |
| *****APOC1-2629 | G>A | **rs369438021** | Exon 3 |  | 0.005 | 0.011 | 0.000 | 1.000 | 100 |
| *****APOC1-2817 | C>T | **rs555051945** | Intron 3 |  | 0.005 | 0.011 | 0.000 | 1.000 | 100 |
| APOC1-3423 | G>A | rs389261 | Intron 3 |  | 0.005 | 0.000 | 0.010 | 1.000 | 100 |
| *****APOC1-3494 | C>T | **rs559574042** | Intron 3 |  | 0.005 | 0.011 | 0.000 | 1.000 | 100 |
| APOC1-4334 | G>A | rs12721046 | Intron 3 |  | 0.181 | 0.202 | 0.160 | 0.288 | 98.9 |
| APOC1-4957 | G>A | rs484195 | Intron 3 |  | **** | **** | **** | **** | **** |
| APOC1-5053_5054 | del1 | rs12721052 | Intron 3 |  | 0.306 | 0.326 | 0.287 | 1.000 | 97.9 |
| APOC1-5240 | C>G | rs12721051 | Intron 3 |  | 0.209 | 0.226 | 0.193 | 0.935 | 90.5 |
| APOC1-5641 | T>G | rs1064725 | 3'UTR |  | 0.037 | 0.021 | 0.052 | 1.000 | 100 |
| *****APOC1-5773 | G>A | **rs568313508** | 3'flanking |  | 0.005 | 0.011 | 0.000 | 1.000 | 100 |
| APOC1-5926 | G>A | rs56131196 | 3'flanking |  | 0.221 | 0.234 | 0.208 | 0.541 | 100 |
| APOC1-6026 | A>G | rs4420638 | 3'flanking |  | 0.221 | 0.234 | 0.208 | 0.541 | 100 |
| APOC1-6122 | G>C | rs142134314 | 3'flanking |  | 0.005 | 0.000 | 0.010 | 1.000 | 100 |
| *****APOC1-6213 | G>A | **rs756508011** | 3'flanking |  | 0.005 | 0.011 | 0.000 | 1.000 | 100 |
| HCR1-292 | C>G | rs4803771 | HCR1 |  | 0.042 | 0.021 | 0.062 | 0.285 | 100 |
| *****HCR1-362 | C>A | **rs557378991** | HCR1 |  | 0.011 | 0.011 | 0.010 | 1.000 | 100 |
| *HCR1-423 | C>G | **rs117664574** | HCR1 |  | 0.026 | 0.043 | 0.010 | 1.000 | 100 |
| HCR1-575 | A>G | rs157599 | HCR1 |  | 0.005 | 0.000 | 0.010 | 1.000 | 100 |
| HCR1-727 | T>G | rs149345 | HCR1 |  | 0.005 | 0.000 | 0.011 | 1.000 | 98.9 |
| HCR2-188 | C>G | rs35136575 | HCR2 |  | 0.242 | 0.277 | 0.208 | 0.263 | 100 |
| *****HCR2-365 | C>A | **rs539409314** | HCR2 |  | 0.011 | 0.011 | 0.010 | 1.000 | 100 |
| HCR2-523 | C>T | rs118004808 | HCR2 |  | 0.026 | 0.021 | 0.031 | 1.000 | 100 |
| APOC4-92_94 | del3 | rs12721101 | 5’ flanking |  | 0.253 | 0.256 | 0.250 | 0.015 | 87.4 |
| APOC4-108 | G>A | rs112391061 | 5’flanking |  | 0.253 | 0.256 | 0.250 | 0.015 | 87.4 |
| *****APOC4-116 | A>G | **rs774571362** | 5’flanking |  | 0.007 | 0.000 | 0.013 | 1.000 | 78.9 |
| *****APOC4-150_152 | ins114 | Novel | 5’flanking |  | 0.256 | 0.262 | 0.250 | 0.022 | 88.4 |
| APOC4-204 | G>A | rs4803773 | 5’flanking |  | 0.250 | 0.212 | 0.292 | 0.237 | 52.6 |
| *****APOC4-636 | C>T | **rs371539058** | 5’flanking |  | 0.005 | 0.000 | 0.010 | 1.000 | 100 |
| APOC4-968 | A>G | rs76214972 | 5’ UTR |  | 0.037 | 0.011 | 0.062 | 1.000 | 100 |
| APOC4-1150 | A>G | rs148247675 | Intron 1 |  | 0.005 | 0.011 | 0.000 | 1.000 | 100 |
| APOC4-1229 | G>C | rs370742602 | Intron 1 |  | 0.011 | 0.000 | 0.021 | 1.000 | 100 |
| APOC4-1325_1327 | del3 | rs79213911 | Intron 1 |  | 0.147 | 0.138 | 0.156 | 1.000 | 89.5 |
| APOC4-1733 | C>T | rs12721111 | Intron 1 |  | 0.263 | 0.276 | 0.250 | 0.016 | 82.1 |
| *****APOC4-1823 | C>G | **rs754628459** | Intron 1 |  | 0.187 | 0.191 | 0.183 | 0.440 | 78.9 |
| *****APOC4-2063 | C>G | **rs763200677** | Intron 1 |  | 0.005 | 0.000 | 0.011 | 1.000 | 98.9 |
| *****APOC4-2557 | C>A | **rs775530121** | Intron 1 |  | 0.005 | 0.000 | 0.010 | 1.000 | 100 |
| APOC4-2623 | C>T | rs5157 | Intron 1 |  | 0.479 | 0.446 | 0.510 | 0.654 | 98.9 |
| APOC4-2640 | C>T | rs5158 | Intron 1 |  | 0.147 | 0.149 | 0.146 | 1.000 | 100 |
| APOC4-2683 | G>A | rs12721109 | Intron 1 |  | 0.011 | 0.011 | 0.010 | 1.000 | 100 |
| APOC2-242 | G>A | rs12691089 | Exon 2 | Gly52Asp | 0.005 | 0.000 | 0.010 | 1.000 | 100 |
| APOC2-194APOC4-3498 | C>T | rs1132899 | Exon 2 | Pro36Leu | 0.474 | 0.436 | 0.510 | 0.898 | 100 |
| APOC2-543 | T>C | rs186448850 | Intron 2 |  | 0.011 | 0.000 | 0.021 | 1.000 | 100 |
| APOC2-623APOC4-3927 | T>G | rs5167 | Exon 3 | Leu96Arg | 0.358 | 0.394 | 0.323 | 0.515 | 100 |
| APOC2-1324 | G>A | rs12721063 | 5'flanking |  | 0.021 | 0.021 | 0.021 | 1.000 | 100 |
| APOC2-1357APOC4-4661 | G>C | rs2288912 | C4-3'/C2-5' |  | 0.484 | 0.457 | 0.510 | 0.885 | 100 |
| APOC2-1442APOC4-4746 | G>T | rs2288911 | APOC2-splice site |  | 0.495 | 0.479 | 0.510 | 0.878 | 100 |
| *****APOC2-1591 | G>A | **rs752555437** | APOC2-Intron 1 |  | 0.005 | 0.000 | 0.010 | 1.000 | 100 |
| APOC2-1851 | C>T | rs12709886 | Intron 1 |  | 0.037 | 0.011 | 0.062 | 1.000 | 100 |
| APOC2-2191 | C>T | rs9304644 | Intron 1 |  | 0.457 | 0.457 | 0.457 | 0.691 | 98.9 |
| APOC2-2410 | T>C | rs12721076 | Intron 1 |  | 0.154 | 0.160 | 0.149 | 1.000 | 98.9 |
| APOC2-2486 | G>A | rs9304645 | Intron 1 |  | 0.188 | 0.185 | 0.191 | 1.000 | 97.9 |
| APOC2-2566 | T>C | rs9304646 | Intron 1 |  | 0.457 | 0.467 | 0.447 | 0.541 | 96.8 |
| *****APOC2-2870 | G>T | **rs559706335** | Intron 1 |  | 0.005 | 0.011 | 0.000 | 1.000 | 100 |
| APOC2-2935 | C>G | rs11879392 | Intron 1 |  | 0.153 | 0.160 | 0.146 | 1.000 | 100 |
| *****APOC2-2938 | A>G | **rs578173884** | Intron 1 |  | 0.005 | 0.011 | 0.000 | 1.000 | 100 |
| APOC2-3010 | A>G | rs10419086 | Intron 1 |  | 0.063 | 0.085 | 0.042 | 1.000 | 100 |
| APOC2-3030 | A>G | rs4803774 | Intron 1 |  | 0.453 | 0.457 | 0.448 | 0.630 | 100 |
| APOC2-3086 | C>T | rs4803775 | Intron 1 |  | 0.484 | 0.543 | 0.490 | 0.885 | 100 |
| APOC2-3348 | G>A | rs10420434 | Intron 1 |  | 0.063 | 0.085 | 0.042 | 1.000 | 100 |
| *****APOC2-3409 | T>C | **rs753768173** | Intron 1 |  | 0.005 | 0.000 | 0.010 | 1.000 | 100 |
| APOC2-3600 | A>G | rs7256684 | Intron 1 |  | 0.453 | 0.457 | 0.448 | 0.630 | 100 |
| APOC2-3778 | A>T | rs5120 | Intron 1 |  | 0.484 | 0.457 | 0.510 | 0.885 | 100 |
| APOC2-3814 | T>G | rs10422603 | Intron 1 |  | 0.189 | 0.191 | 0.188 | 1.000 | 100 |
| APOC2-4429 | G>C | rs3745152 | Intron 3 |  | 0.489 | 0.489 | 0.489 | 0.018 | 96.8 |
| APOC2-4430_4431 | ins3 | rs35625559 | Intron 3 |  | 0.489 | 0.489 | 0.489 | 0.018 | 96.8 |
| *****APOC2-4477 | G>A | **rs765929871** | Intron 3 |  | 0.005 | 0.011 | 0.000 | 1.000 | 95.8 |
| APOC2-4493 | C>T | rs4803776 | Intron 3 |  | 0.483 | 0.543 | 0.489 | 0.800 | 94.7 |
| APOC2-4532 | C>T | **rs767229989** | Intron 3 |  | 0.075 | 0.098 | 0.054 | 1.000 | 91.6 |
| *****APOC2-4534_4535 | del1 | **rs779524886** | Intron 3 |  | 0.075 | 0.098 | 0.054 | 1.000 | 91.6 |
| *****APOC2-4853_4854 | del1 | rs150448996 | 3'flanking |  | 0.261 | 0.261 | 0.260 | 1.000 | 98.9 |
| APOC2-4971 | C>T | rs1130742 | 3'flanking |  | 0.295 | 0.713 | 0.698 | 0.100 | 100 |
| APOC2-5004 | G>A | rs10421404 | 3'flanking |  | 0.189 | 0.191 | 0.188 | 1.000 | 100 |
| APOC2-5018_5022 | del5 | rs78403558 | 3'flanking |  | 0.153 | 0.160 | 0.146 | 1.000 | 100 |
| APOC2-5303 | C>T | rs7257468 | 3'flanking |  | 0.458 | 0.468 | 0.448 | 0.480 | 100 |
| APOC2-5310 | T>G | rs7258345 | 3'flanking |  | 0.457 | 0.543 | 0.543 | 0.614 | 97.9 |
| APOC2-5324 | C>T | rs7257476 | 3'flanking |  | 0.458 | 0.468 | 0.448 | 0.480 | 100 |
| APOC2-5398 | G>A | rs12709889 | 3'flanking |  | 0.268 | 0.277 | 0.260 | 1.000 | 100 |
| *****APOC2-5644 | G>A | **rs112144355** | 3'flanking |  | 0.011 | 0.011 | 0.010 | 1.000 | 100 |
| APOC2-5815 | G>A | rs10423208 | 3'flanking |  | 0.453 | 0.457 | 0.448 | 0.630 | 100 |
| APOC2-5922 | A>G | rs10422888 | 3'flanking |  | 0.149 | 0.160 | 0.138 | 1.000 | 98.9 |
| APOC2-6037 | G>A | rs10402642 | 3'flanking |  | 0.453 | 0.457 | 0.448 | 0.630 | 100 |

Nucleotide position is according to the reference sequence NC_000019.9; Grey-shaded variants represent variants observed in both populations, (****) represents insufficient data. HWE-P: Hardy Weinberg Equillibirium p-value. *Novel variants. **Bold** rs# numbers represent novel refSNP IDs assigned as a result of our dbSNP submission (http://www.ncbi.nlm.nih.gov/SNP/snp_viewTable.cgi?handle5KAMBOH).
