# Supplementary material for: Apolipoprotein E-C1-C4-C2 gene cluster region and inter-individual variation in plasma lipoprotein levels: a comprehensive genetic association study in two ethnic groups
Source: PLoS One. 2019 Mar 26;14(3):e0214060. doi: 10.1371/journal.pone.0214060 (PMC6435132; doi:10.1371/journal.pone.0214060)
Supplement: S5 Table — Nucleotide position is according to the reference sequence NC_000019.9; Grey-shaded variants represent variants observed in both populations; (****) represent insufficient data. HWE-P: Hardy Weinberg Equilibirium p-value; *Novel variants. Bold rs numbers represent novel refSNP IDs assigned as a result of our dbSNP submission (http://www.ncbi.nlm.nih.gov/SNP/snp_viewTable.cgi?handle5KAMBOH). (DOCX) [file pone.0214060.s005.docx]

S5 Table. Sequencing results for the *APOE/C1/C4/C2* gene cluster in ABs (n=95)

| **Variant Name** | **Alleles** | **RefSNP ID** | **Location** | | **Amino acid change** | **MAF in the total sample** | **MAF in High HDL-C/low TG group** | **MAF in Low HDL-C/high TG group** | **HWE-P** | **Call rate (%)** |
| --- | --- | --- | --- | --- | --- | --- | --- | --- | --- | --- |
| APOE-73 | C>T | rs1081101 | 5'flanking | |  | 0.074 | 0.062 | 0.087 | 1.000 | 98.9 |
| *APOE-173 | A>G | **rs546268923** | 5'flanking | |  | 0.005 | 0.000 | 0.011 | 1.000 | 100 |
| APOE-308 | C>T | rs769445 | 5'flanking | |  | 0.005 | 0.000 | 0.011 | 1.000 | 100 |
| APOE-471 | A>G | rs439382 | 5'flanking | |  | 0.132 | 0.125 | 0.138 | 1.000 | 100 |
| *APOE-494 | C>T | **rs534584472** | 5'flanking | |  | 0.005 | 0.010 | 0.000 | 1.000 | 100 |
| *APOE-526 | T>C | **rs781138752** | 5'flanking | |  | 0.005 | 0.010 | 0.000 | 1.000 | 100 |
| APOE-560 | A>T | rs449647 | 5'flanking | |  | 0.395 | 0.385 | 0.404 | 0.169 | 100 |
| *APOE-618 | G>C | **rs756735196** | 5'flanking | |  | 0.005 | 0.000 | 0.011 | 1.000 | 100 |
| APOE-624 | T>C | rs769446 | 5'flanking | |  | 0.005 | 0.010 | 0.000 | 1.000 | 100 |
| APOE-832 | G>T | rs405509 | 5'flanking | |  | 0.261 | 0.260 | 0.261 | 0.337 | 98.9 |
| APOE-1109 | C>T | rs9282609 | Splice site | |  | 0.053 | 0.052 | 0.054 | 1.000 | 98.9 |
| APOE-1163 | G>C | rs440446 | Intron 1 | |  | 0.124 | 0.115 | 0.133 | 0.822 | 97.9 |
| *APOE-1231 | G>A | **rs545943117** | Intron 1 | |  | 0.011 | 0.010 | 0.011 | 1.000 | 98.9 |
| APOE-1279 | C>A | rs877973 | Intron 1 | |  | 0.048 | 0.062 | 0.033 | 1.000 | 98.9 |
| APOE-1539 | A>G | rs184686013 | Intron 1 | |  | 0.011 | 0.021 | 0.000 | 1.000 | 100 |
| APOE-1591 | G>T | rs147236548 | Intron 1 | |  | 0.016 | 0.021 | 0.011 | 1.000 | 100 |
| APOE-2072 | G>A | rs189660912 | Intron 2 | |  | 0.016 | 0.021 | 0.011 | 1.000 | 100 |
| APOE-2269 | G>A | rs61357706 | Intron 2 | |  | 0.016 | 0.010 | 0.022 | 1.000 | 98.9 |
| APOE-2440 | G>A | rs769450 | Intron 2 | |  | 0.394 | 0.383 | 0.404 | 0.978 | 98.9 |
| APOE-2544 | A>G | rs115299243 | Intron 2 | |  | 0.016 | 0.010 | 0.022 | 1.000 | 98.9 |
| *APOE-2576 | G>A | **rs745575113** | Intron 2 | |  | 0.005 | 0.010 | 0.000 | 1.000 | 100 |
| APOE-3673 | C>G | rs769453 | Intron 3 | |  | 0.005 | 0.000 | 0.011 | 1.000 | 100 |
| APOE-3937 | T>C | rs429358 | Exon 4 | | Cys130Arg | 0.237 | 0.240 | 0.234 | 0.201 | 100 |
| APOE-4036 | C>T | rs769455 | Exon 4 | | Arg163Cys | 0.016 | 0.011 | 0.022 | 1.000 | 96.8 |
| APOE-4075 | G>A | rs7412 | Exon 4 | | Arg176Cys | 0.042 | 0.062 | 0.021 | 1.000 | 100 |
| *APOE-4569 | G>T | **rs762567388** | 3'UTR | |  | 0.005 | 0.010 | 0.000 | 1.000 | 100 |
| APOE-4951 | A>C | rs1081105 | 3'flanking | |  | 0.042 | 0.042 | 0.043 | 1.000 | 100 |
| *APOE-5223 | G>C | **rs771320440** | 3'flanking | |  | 0.005 | 0.000 | 0.011 | 1.000 | 100 |
| APOE-5229 | G>T | rs1065853 | 3'flanking | |  | 0.059 | 0.074 | 0.043 | 1.000 | 98.9 |
| APOE-5230 | INDEL | rs55729972 | 3'flanking | |  | **** | **** | **** | **** | **** |
| *APOE-5231 | T>G | **rs747405425** | 3'flanking | |  | 0.032 | 0.042 | 0.021 | 1.000 | 100 |
| APOC1-720_721 | ins4 | rs11568822 | 5'flanking | |  | 0.237 | 0.260 | 0.213 | 1.000 | 100 |
| APOC1-894 | C>T | rs190454394 | 5'flanking | |  | 0.005 | 0.010 | 0.000 | 1.000 | 100 |
| APOC1-1166 | G>A | rs72654452 | Intron 1 | |  | 0.032 | 0.031 | 0.032 | 1.000 | 100 |
| APOC1-1331 | G>A | rs10408994 | Intron 2 | |  | 0.108 | 0.098 | 0.117 | 0.637 | 97.9 |
| APOC1-1526 | C>T | rs5114 | Intron 2 | |  | 0.047 | 0.062 | 0.032 | 1.000 | 100 |
| *APOC1-1642 | C>T | **rs568979138** | Intron 2 | |  | 0.011 | 0.010 | 0.011 | 1.000 | 98.9 |
| APOC1-1684 | G>A | rs12709881 | Intron 2 | |  | 0.075 | 0.083 | 0.067 | 1.000 | 97.9 |
| APOC1-1870 | T>C | rs5117 | Intron 2 | |  | 0.212 | 0.239 | 0.185 | 0.756 | 96.8 |
| APOC1-2041 | C>T | rs3826688 | Intron 2 | |  | 0.126 | 0.115 | 0.138 | 0.894 | 100 |
| *APOC1-2099 | T>C | **rs777015592** | Intron 2 | |  | 0.005 | 0.000 | 0.011 | 1.000 | 100 |
| APOC1-2296 | A>T | rs185495905 | Intron 2 | |  | 0.005 | 0.010 | 0.000 | 1.000 | 100 |
| *APOC1-3358 | A>G | **rs527446270** | Intron 3 | |  | 0.005 | 0.010 | 0.000 | 1.000 | 100 |
| APOC1-3423 | G>A | rs389261 | Intron 3 | |  | 0.389 | 0.406 | 0.372 | 0.011 | 100 |
| APOC1-3573 | G>A | rs10424339 | Intron 3 | |  | 0.181 | 0.198 | 0.163 | 0.761 | 98.9 |
| APOC1-5006 | G>T | rs112528434 | Intron 3 | |  | 0.081 | 0.083 | 0.078 | 1.000 | 97.9 |
| APOC1-5053_5054 | del1 | rs12721052 | Intron 3 | |  | 0.215 | 0.191 | 0.239 | 1.000 | 97.9 |
| APOC1-5240 | C>G | rs12721051 | Intron 3 | |  | 0.035 | 0.045 | 0.024 | 1.000 | 89.5 |
| APOC1-5667 | A>G | rs12721054 | 3'UTR | |  | 0.137 | 0.135 | 0.138 | 0.914 | 100 |
| APOC1-5716 | G>T | rs12721055 | 3'flanking | |  | 0.026 | 0.031 | 0.021 | 1.000 | 100 |
| APOC1-5717 | G>C | rs72654457 | 3'flanking | |  | 0.021 | 0.021 | 0.021 | 1.000 | 100 |
| APOC1-5926 | G>A | rs56131196 | 3'flanking | |  | 0.153 | 0.156 | 0.149 | 0.160 | 100 |
| APOC1-6026 | A>G | rs4420638 | 3'flanking | |  | 0.158 | 0.156 | 0.160 | 0.131 | 100 |
| APOC1-6158 | G>A | rs370098302 | 3'flanking | |  | 0.005 | 0.000 | 0.011 | 1.000 | 100 |
| HCR1-424 | G>A | rs117664574 | HCR1 | |  | 0.011 | 0.021 | 0.000 | 1.000 | 100 |
| HCR1-575 | A>G | rs157599 | HCR1 | |  | 0.424 | 0.448 | 0.398 | 0.034 | 96.8 |
| HCR1-727 | T>G | rs149345 | HCR1 | |  | 0.392 | 0.404 | 0.380 | 0.012 | 97.9 |
| HCR2-188 | C>G | rs35136575 | HCR2 | |  | 0.181 | 0.181 | 0.181 | 1.000 | 98.9 |
| *HCR2-286 | G>A | **rs150849246** | HCR2 | |  | 0.059 | 0.032 | 0.085 | 1.000 | 98.9 |
| HCR2-523 | C>T | rs118004808 | HCR2 | |  | 0.005 | 0.010 | 0.000 | 1.000 | 100 |
| *HCR2-632 | T>C | **rs773414210** | HCR2 | |  | 0.083 | 0.033 | 0.136 | 0.940 | 94.7 |
| *APOC4-65 | C>T | **rs754162992** | 5’ flanking | |  | 0.007 | 0.000 | 0.015 | 1.000 | 77.9 |
| APOC4-92_94 | del3 | rs12721101 | 5’ flanking | |  | 0.262 | 0.209 | 0.321 | 0.571 | 86.3 |
| APOC4-108 | G>A | rs112391061 | 5’ flanking | |  | 0.262 | 0.209 | 0.321 | 0.571 | 86.3 |
| *APOC4-150_151 | ins114 | Novel | 5’ flanking | |  | 0.283 | 0.228 | 0.341 | 1.000 | 94.7 |
| APOC4-204 | G>A | rs4803773 | 5’ flanking | |  | 0.233 | 0.250 | 0.216 | 1.000 | 94.7 |
| *APOC4-233 | C>T | **rs538318669** | 5’ flanking | |  | 0.006 | 0.000 | 0.011 | 1.000 | 93.7 |
| APOC4-245 | G>T | rs192072159 | 5’ flanking | |  | 0.006 | 0.000 | 0.011 | 1.000 | 94.7 |
| *APOC4-368 | A>G | **rs559795225** | 5’ flanking | |  | 0.011 | 0.010 | 0.011 | 1.000 | 100 |
| APOC4-438 | G>A | rs146656012 | 5’ flanking | |  | 0.006 | 0.000 | 0.011 | 1.000 | 94.7 |
| APOC4-489 | C>T | rs140241604 | 5’ flanking | |  | 0.017 | 0.011 | 0.023 | 1.000 | 93.7 |
| APOC4-637 | G>T | rs113814026 | 5’ flanking | |  | 0.042 | 0.042 | 0.043 | 1.000 | 100 |
| APOC4-757 | C>A | rs12721105 | 5’ flanking | |  | 0.058 | 0.062 | 0.053 | 0.052 | 100 |
| APOC4-1088 | T>G | rs367589753 | Intron 1 | |  | 0.005 | 0.000 | 0.011 | 1.000 | 100 |
| *APOC4-1130 | T>C | **rs751337487** | Intron 1 | |  | 0.005 | 0.010 | 0.000 | 1.000 | 100 |
| APOC4-1192 | G>A | rs113745034 | Intron 1 | |  | 0.011 | 0.010 | 0.011 | 1.000 | 100 |
| APOC4-1325_1327 | del3 | rs79213911 | Intron 1 | |  | 0.043 | 0.064 | 0.022 | 1.000 | 97.9 |
| *APOC4-1430_1431 | insG | **rs763894138** | Intron 1 | |  | 0.037 | 0.042 | 0.032 | 0.216 | 100 |
| APOC4-1702 | G>A | rs12721102 | Intron 1 | |  | 0.006 | 0.011 | 0.000 | 1.000 | 91.6 |
| *APOC4-1719 | C>A | **rs780999048** | Intron 1 | |  | 0.006 | 0.011 | 0.000 | 1.000 | 91.6 |
| APOC4-1733 | C>T | rs1271111 | Intron 1 | |  | 0.244 | 0.228 | 0.262 | 1.000 | 90.5 |
| APOC4-1823 | C>G | **rs754628459** | Intron 1 | |  | 0.203 | 0.238 | 0.156 | 0.286 | 77.9 |
| APOC4-2099 | G>T | rs111339708 | Intron 1 | |  | 0.011 | 0.010 | 0.011 | 1.000 | 100 |
| APOC4-2467 | C>T | rs115225947 | Intron 1 | |  | 0.021 | 0.021 | 0.021 | 1.000 | 100 |
| APOC4-2559 | C>T | rs5155 | Intron 1 | |  | 0.09 | 0.074 | 0.106 | 1.000 | 98.9 |
| APOC4-2607 | G>A | rs5156 | Intron 1 | |  | 0.011 | 0.010 | 0.011 | 1.000 | 100 |
| APOC4-2623 | C>T | rs5157 | Intron 1 | |  | 0.163 | 0.167 | 0.160 | 1.000 | 100 |
| APOC4-2640 | C>T | rs5158 | Intron 1 | |  | 0.032 | 0.042 | 0.021 | 1.000 | 100 |
| *APOC4-2641 | G>A | **rs542078429** | Intron 1 | |  | 0.005 | 0.000 | 0.011 | 1.000 | 97.9 |
| APOC4-2678 | G>C | rs148564866 | Intron 1 | |  | 0.005 | 0.000 | 0.011 | 1.000 | 100 |
| APOC4-2767 | G>T | rs12721107 | Intron 1 | |  | 0.021 | 0.031 | 0.011 | 1.000 | 100 |
| APOC4-2971 | A>G | rs5159 | Intron 1 | |  | 0.15 | 0.152 | 0.148 | 0.613 | 94.7 |
| APOC4-3213 | T>C | rs28616151 | Intron 1 | |  | 0.056 | 0.064 | 0.048 | 0.038 | 93.7 |
| *APOC4-3348 | G>A | **rs769339360** | Intron 1 | |  | 0.005 | 0.011 | 0.000 | 1.000 | 97.9 |
| APOC2-59APOC4-3363 | G>A | rs60170431 | APOC4-Intron 1 | |  | 0.043 | 0.053 | 0.032 | 1.000 | 98.9 |
| APOC2-75APOC4-3380 | G>A | rs12721104 | APOC4-Intron 1 | |  | 0.147 | 0.146 | 0.149 | 0.638 | 100 |
| APOC2-194APOC4-3498 | C>T | rs1132899 | APOC4-Exon 2 | | Leu36Pro | 0.232 | 0.240 | 0.223 | 1.000 | 100 |
| APOC2-198-APOC4-3502 | C>T | rs10423683 | APOC4-Exon 2 | | Ser37Ser | 0.058 | 0.062 | 0.053 | 0.052 | 100 |
| APOC2-228 | G>A | rs5164 | APOC4-Exon 2 | | Trp47Ter | 0.005 | 0.000 | 0.011 | 1.000 | 100 |
| APOC2-288-APOC4-3592 | C>T | rs12691090 | APOC4-Exon 2 | | Asp67Asp | 0.026 | 0.042 | 0.011 | 1.000 | 100 |
| *APOC2-396APOC4-3700 | G>A | **rs777229733** | APOC4-Intron 2 | |  | 0.005 | 0.010 | 0.000 | 1.000 | 100 |
| APOC2-488APOC4-3792 | G>A | rs5165 | APOC4-Intron 2 | |  | 0.016 | 0.021 | 0.011 | 1.000 | 100 |
| APOC2-623APOC4-3927 | T>G | rs5167 | APOC4-Exon 3 | | Leu96Arg | 0.011 | 0.010 | 0.011 | 1.000 | 100 |
| APOC2-665APOC4-3969 | A>C | rs138548797 | APOC4-Exon 3 | | Lys110Thr | 0.468 | 0.448 | 0.489 | 0.747 | 100 |
| *APOC2-708APOC4-4012 | G>A | **rs757860005** | APOC4-Exon 3 | | Lys124Lys | 0.005 | 0.000 | 0.011 | 1.000 | 100 |
| APOC2-850APOC4-4154 | G>A | rs12709884 | APOC4-3' UTR | |  | 0.112 | 0.104 | 0.120 | 0.623 | 98.9 |
| APOC2-853APOC4-4157 | G>A | rs10425530 | APOC4-3' UTR | |  | 0.101 | 0.104 | 0.098 | 1.000 | 98.9 |
| APOC2-1042APOC4-4346 | A>T | rs12709885 | APOC4-3'/APOC2-5' | |  | 0.005 | 0.010 | 0.000 | 1.000 | 98.9 |
| APOC2-1187APOC4-4491 | G>A | rs111782345 | APOC4-3'/APOC2-5' | |  | 0.005 | 0.000 | 0.011 | 1.000 | 97.9 |
| APOC2-1229APOC4-4533 | C>T | rs112698600 | APOC4-3'/APOC2-5' | |  | 0.011 | 0.010 | 0.011 | 1.000 | 98.9 |
| APOC2-1275APOC4-4579 | G>A | rs111356234 | APOC4-3'/APOC2-5' | |  | 0.053 | 0.031 | 0.076 | 1.000 | 98.9 |
| APOC2-1357APOC4-4661 | G>C | rs2288912 | APOC4-3'/APOC2-5' | |  | 0.232 | 0.240 | 0.223 | 1.000 | 100 |
| APOC2-1442APOC4-4746 | G>T | rs2288911 | APOC2-Exon 1/UTR | |  | 0.207 | 0.223 | 0.189 | 0.857 | 96.8 |
| APOC2-1540APOC4-4844 | G>A | rs75463753 | APOC2-Intron 1 | |  | 0.095 | 0.094 | 0.096 | 0.371 | 100 |
| *APOC2-1608APOC4-4912 | G>C | **rs770592644** | APOC2-Intron 1 | |  | 0.005 | 0.000 | 0.011 | 1.000 | 100 |
| APOC2-2003 | G>A | rs187834478 | Intron 1 | |  | 0.016 | 0.021 | 0.011 | 1.000 | 100 |
| APOC2-2090 | G>A | rs12721072 | Intron 1 | |  | 0.058 | 0.052 | 0.064 | 1.000 | 100 |
| APOC2-2191 | T>C | rs9304644 | Intron 1 | |  | 0.358 | 0.365 | 0.351 | 1.000 | 100 |
| APOC2-2258 | A>C | rs116489256 | Intron 1 | |  | 0.058 | 0.052 | 0.064 | 1.000 | 100 |
| APOC2-2339 | A>G | rs115943687 | Intron 1 | |  | 0.058 | 0.052 | 0.064 | 1.000 | 100 |
| APOC2-2371 | A>G | **rs746606616** | Intron 1 | |  | 0.005 | 0.010 | 0.000 | 1.000 | 100 |
| APOC2-2410 | T>C | rs12721076 | Intron 1 | |  | 0.032 | 0.042 | 0.021 | 1.000 | 100 |
| APOC2-2486 | G>A | rs9304645 | Intron 1 | |  | 0.394 | 0.415 | 0.372 | 0.685 | 98.9 |
| APOC2-2566 | C>T | rs9304646 | Intron 1 | |  | 0.288 | 0.293 | 0.283 | 1.000 | 96.8 |
| APOC2-2935 | C>G | rs11879392 | Intron 1 | |  | 0.027 | 0.042 | 0.011 | 1.000 | 98.9 |
| APOC2-3010 | A>G | rs10419086 | Intron 1 | |  | 0.142 | 0.146 | 0.138 | 0.232 | 100 |
| APOC2-3030 | G>A | rs4803774 | Intron 1 | |  | 0.289 | 0.302 | 0.277 | 1.000 | 100 |
| *APOC2-3082_3083 | ins3 | **rs755301852** | Intron 1 | |  | 0.058 | 0.052 | 0.064 | 1.000 | 100 |
| APOC2-3086 | C>T | rs4803775 | Intron 1 | |  | 0.158 | 0.167 | 0.149 | 0.343 | 100 |
| APOC2-3154 | G>A | rs145931717 | Intron 1 | |  | 0.058 | 0.052 | 0.064 | 1.000 | 100 |
| APOC2-3193 | A>G | rs148616221 | Intron 1 | |  | 0.005 | 0.000 | 0.011 | 1.000 | 100 |
| *APOC2-3234 | T>C | **rs772477913** | Intron 1 | |  | 0.005 | 0.010 | 0.000 | 1.000 | 100 |
| APOC2-3259 | C>T | rs12721061 | Intron 1 | |  | 0.058 | 0.052 | 0.064 | 1.000 | 100 |
| APOC2-3260 | G>A | rs151176577 | Intron 1 | |  | 0.011 | 0.000 | 0.021 | 1.000 | 100 |
| APOC2-3348 | G>A | rs10420434 | Intron 1 | |  | 0.126 | 0.135 | 0.117 | 0.379 | 100 |
| APOC2-3600 | G>A | rs7256684 | Intron 1 | |  | 0.287 | 0.298 | 0.277 | 1.000 | 98.9 |
| *APOC2-3617 | C>T | **rs748663557** | Intron 1 | |  | 0.005 | 0.000 | 0.011 | 1.000 | 98.9 |
| APOC2-3692 | T>G | rs12721060 | Intron 1 | |  | 0.011 | 0.011 | 0.011 | 1.000 | 98.9 |
| APOC2-3778 | A>T | rs5120 | Intron 1 | |  | 0.158 | 0.167 | 0.149 | 0.343 | 100 |
| APOC2-3805 | C>G | rs7257095 | Intron 1 | |  | 0.163 | 0.138 | 0.189 | 0.524 | 96.8 |
| APOC2-3806 | C>G | rs12709887 | Intron 1 | |  | 0.043 | 0.053 | 0.032 | 1.000 | 98.9 |
| APOC2-3814 | T>G | rs10422603 | Intron 1 | |  | 0.306 | 0.340 | 0.272 | 0.588 | 97.9 |
| APOC2-3892 | C>T | rs5121 | Exon 2/UTR | |  | 0.027 | 0.021 | 0.033 | 1.000 | 96.8 |
| APOC2-4086 | G>A | rs114780592 | Intron 2 | |  | 0.021 | 0.031 | 0.011 | 1.000 | 100 |
| APOC2-4112 | G>C | rs74500990 | Intron 2 | |  | 0.059 | 0.052 | 0.065 | 1.000 | 98.9 |
| APOC2-4118 | G>A | rs201709243 | Exon 3 | | Val20Ile | 0.005 | 0.010 | 0.000 | 1.000 | 100 |
| APOC2-4319 | G>A | rs5123 | Intron 3 | |  | 0.055 | 0.044 | 0.065 | 1.000 | 95.8 |
| APOC2-4429 | C>G | rs3745152 | Intron 3 | |  | 0.259 | 0.293 | 0.226 | 0.545 | 87.4 |
| APOC2-4430_4431 | ins3 | rs35625559 | Intron 3 | |  | 0.259 | 0.293 | 0.226 | 0.545 | 87.4 |
| *APOC2-4458 | C>T | **rs573032402** | Intron 3 | |  | 0.024 | 0.043 | 0.000 | 1.000 | 44.2 |
| *APOC2-4460_4461 | del1 | **rs773467390** | Intron 3 | |  | 0.157 | 0.161 | 0.154 | 0.449 | 56.8 |
| APOC2-4493 | C>T | rs4803776 | Intron 3 | |  | 0.179 | 0.188 | 0.170 | 0.002 | 96.8 |
| APOC2-4513 | A>C | rs180809422 | Intron 3 | |  | 0.011 | 0.011 | 0.011 | 1.000 | 92.6 |
| *APOC2-4532 | C>T | **rs767229989** | Intron 3 | |  | 0.194 | 0.216 | 0.167 | 0.097 | 70.5 |
| *APOC2-4534_4535 | del1 | **rs779524886** | Intron 3 | |  | 0.165 | 0.171 | 0.158 | 0.170 | 83.2 |
| APOC2-4587 | A>C | rs5126 | Exon 4 | | Lys77Gln | 0.044 | 0.044 | 0.043 | 1.000 | 95.8 |
| APOC2-4754 | C>T | rs7253690 | Exon 4/UTR | |  | 0.065 | 0.062 | 0.067 | 1.000 | 97.9 |
| APOC2-4853_4854 | del1 | rs150448996 | 3'flanking | | | 0.272 | 0.255 | 0.289 | 0.659 | 96.8 |
| APOC2-4971 | C>T | rs1130742 | 3'flanking |  | | 0.279 | 0.740 | 0.702 | 0.529 | 100 |
| APOC2-4973_4974 | ins1 | rs199828513 | 3'flanking | | | 0.005 | 0.000 | 0.011 | 1.000 | 100 |
| APOC2-5004 | G>A | rs10421404 | 3'flanking |  | | 0.305 | 0.344 | 0.266 | 0.551 | 100 |
| APOC2-5018_5022 | del5 | rs78403558 | 3'flanking |  | | 0.058 | 0.083 | 0.032 | 1.000 | 100 |
| APOC2-5303 | T>C | rs7257468 | 3'flanking |  | | 0.284 | 0.292 | 0.277 | 1.000 | 100 |
| APOC2-5310 | G>T | rs7258345 | 3'flanking |  | | 0.285 | 0.302 | 0.267 | 1.000 | 97.9 |
| APOC2-5324 | T>C | rs7257476 | 3'flanking |  | | 0.295 | 0.302 | 0.287 | 1.000 | 100 |
| APOC2-5398 | G>A | rs12709889 | 3'flanking |  | | 0.25 | 0.240 | 0.261 | 1.000 | 98.9 |
| *APOC2-5491 | C>T | **rs190382225** | 3’flanking |  | | 0.005 | 0.010 | 0.000 | 1.000 | 100 |
| APOC2-5512 | G>A | rs12721064 | 3'flanking |  | | 0.005 | 0.010 | 0.000 | 1.000 | 100 |
| *APOC2-5562 | G>C | **rs138369841** | 3'flanking |  | | 0.021 | 0.010 | 0.032 | 1.000 | 100 |
| APOC2-5586 | T>G | rs73558127 | 3'flanking |  | | 0.089 | 0.104 | 0.074 | 1.000 | 100 |
| *APOC2-5612 | A>G | **rs116675446** | 3'flanking |  | | 0.063 | 0.062 | 0.064 | 1.000 | 100 |
| *APOC2-5771_5775 | del5 | **rs750595202** | 3'flanking |  | | 0.005 | 0.011 | 0.000 | 1.000 | 97.9 |
| APOC2-5815 | A>G | rs10423208 | 3'flanking |  | | 0.293 | 0.302 | 0.283 | 1.000 | 98.9 |
| APOC2-5922 | A>G | rs10422888 | 3'flanking |  | | 0.121 | 0.156 | 0.085 | 0.258 | 100 |
| *APOC2-5965 | G>A | **rs531834248** | 3'flanking |  | | 0.005 | 0.010 | 0.000 | 1.000 | 100 |
| APOC2-6037 | A>G | rs10402642 | 3'flanking |  | | 0.295 | 0.302 | 0.287 | 1.000 | 100 |
| *APOC2-6222 | G>C | **rs144490316** | 3'flanking |  | | 0.011 | 0.010 | 0.989 | 1.000 | 100 |
| *APOC2-6334 | G>A | **rs573344137** | 3'flanking |  | | 0.026 | 0.031 | 0.021 | 1.000 | 100 |

Nucleotide position is according to the reference sequence NC_000019.9; Grey-shaded variants represent variants observed in both populations; (****) represent insufficient data. HWE-P: Hardy Weinberg Equilibirium p-value; *Novel variants. **Bold** rs numbers represent novel refSNP IDs assigned as a result of our dbSNP submission (http://www.ncbi.nlm.nih.gov/SNP/snp_viewTable.cgi?handle5KAMBOH).
