# Supplementary material for: Apolipoprotein E-C1-C4-C2 gene cluster region and inter-individual variation in plasma lipoprotein levels: a comprehensive genetic association study in two ethnic groups
Source: PLoS One. 2019 Mar 26;14(3):e0214060. doi: 10.1371/journal.pone.0214060 (PMC6435132; doi:10.1371/journal.pone.0214060)
Supplement: S6 Table — Bold variants represent those genotyped successfully. Italics variants represent those failed genotyping or post-genotyping QC. (DOCX) [file pone.0214060.s006.docx]

S6 Table. Tagger results for the *APOE/C1/C4/C2* gene cluster variants (MAF≥5%, r^2^=0.9) identified by sequencing in NHWs

| **Bin 1-28** | **Alleles Captured** |
| --- | --- |
| APOC2-5303 | APOC2-5324, APOC2-3600, APOC2-6037, APOC2-2191, **APOC2-5310**, APOC2-5815, APOC2-5303, APOC2-3030, APOC2-2566 |
| **APOC2-194APOC4-3498** | APOC2-3086, **APOC2-1357, APOC4-4661**, **APOC4-2623**, APOC2-1442APOC4-4746, APOC2-4493, APOC2-194APOC4-3498, APOC4-204, **APOC2-3778** |
| APOC2-5018 | APOC4-1325del3, APOC2-2935, **APOC4-2640**, APOC2-5922, APOC2-2410, APOC2-5018 |
| *APOC4-108* | APOC4-92del3, *APOC4-108*, APOC4-150ins114 |
| APOC2-3814 | **APOC2-5004**, APOC2-3814, APOC2-2486 |
| **APOC1-6026 (rs4420638)** | **APOC1-5926 (rs56131196)**, APOC1-5240, **APOC1-6026 (rs4420638)** |
| *APOC2-4534* | *APOC2-4534*, APOC2-4532 |
| *APOC2-4429* | *APOC2-4429*, APOC2-4430ins |
| **APOC1-720 (rs11568822)** | APOC1-1870, **APOC1-720 (rs11568822)** |
| **APOC2-4853** | **APOC2-5398**, APOC2-4853 |
| **APOC2-3348** | APOC2-3010, APOC2-3348 |
| APOE-5229 | **APOE-4075 (rs7412)**, APOE-5229 |
| **APOE-1163 (rs440446)** | **APOE-1163 (rs440446)** |
| **APOC1-4334 (rs1064725)** | **APOC1-4334 (rs1064725)** |
| **APOE-560 (rs449647)** | **APOE-560 (rs449647)** |
| **APOE-2440 (rs769450)** | **APOE-2440 (rs769450)** |
| **APOC2-623APOC4-3927** | **APOC2-623APOC4-3927** |
| *APOC4-1733* | APOC4-1733 |
| *APOC4-1823* | APOC4-1823 |
| **APOC1-2041(rs3826688)** | **APOC1-2041 (rs3826688)** |
| **APOE-5361** | **APOE-5361** |
| *APOC1-5053* | *APOC1-5053* |
| **HCR2-188 (rs35136575)** | **HCR2-188 (rs35136575)** |
| **APOE-1998 (rs769449)** | **APOE-1998 (rs769449)** |
| **APOE-624** | **APOE-624** |
| **APOE-3937 (rs429358)** | **APOE-3937** |
| *APOC2-4971* | APOC2-4971 |
| **APOE-832 (rs405509)** | **APOE-832 (rs405509)** |

**Bold** variants represent those genotyped successfully. *Italics* variants represent those failed genotyping or post-genotyping QC.
