# Supplementary material for: Apolipoprotein E-C1-C4-C2 gene cluster region and inter-individual variation in plasma lipoprotein levels: a comprehensive genetic association study in two ethnic groups
Source: PLoS One. 2019 Mar 26;14(3):e0214060. doi: 10.1371/journal.pone.0214060 (PMC6435132; doi:10.1371/journal.pone.0214060)
Supplement: S7 Table — Bold variants represent those genotyped successfully. Italics variants represent those failed genotyping or post-genotyping QC. (DOCX) [file pone.0214060.s007.docx]

S7 Table. Tagger results for the *APOE/C1/C4/C2* gene cluster variants (MAF≥5%, r^2^=0.9) identified by sequencing in ABs

| **Bins 1-55** | **Alleles Captured** |
| --- | --- |
| APOC2-2090 | APOC2-2258, **APOC2-4754**, APOC2-3154, APOC2-3259, **APOC2-4319**, APOC2-2339, APOC2-4112, APOC2-2090, APOC2-5612, APOC2-3082 |
| **APOC2-5310** | APOC2-5303, APOC2-5310, APOC2-6037, APOC2-5324, APOC2-4430, **APOC2-5815 (rs10423208)**, APOC2-3600, APOC2-4429 |
| *APOC4-92* | APOC4-92, APOC4-150, APOC4-108 |
| APOC2-198APOC4-3502 | APOC4-3213, **APOC4-757 (rs12721105)**, APOC2-198APOC4-3502 |
| APOC1-1684 | APOC1-1684, **APOC1-5006** |
| **APOC2-194APOC4-3498** | APOC2-194APOC4-3498, APOC4-204 |
| **APOC2-3778 (rs5120)** | APOC2-3778 (**rs5120)**, APOC2-3086 |
| APOC1-2041 | APOC1-2041, **APOE-1163 (rs440446)** |
| HCR1-727 | HCR1-727, **APOC1-3423** |
| APOC1-6026 | APOC1-6026, **APOC1-5926 (rs56131196)** |
| **APOC2-75APOC4-3380** | APOC2-75APOC4-3380, APOC4-2971 |
| **APOC2-5004** | APOC2-5004, **APOC2-3814** |
| **APOC4-2559** | APOC4-2559 |
| **APOC2-5398** | **APOC2-5398** |
| **APOC2-623APOC4-3927** | **APOC2-623APOC4-3927** |
| **APOC2-1275APOC4-4579** | **APOC2-1275APOC4-4579** |
| **APOC2-5018** | **APOC2-5018** |
| **APOE-73 (rs1081101)** | **APOE-73 (rs1081101)** |
| **APOC2-1357APOC4-4661 (rs2288912)** | **APOC2-1357APOC4-4661 (rs2288912)** |
| **APOE-1109** | **APOE-1109** |
| **APOC2-5586** | **APOC2-5586** |
| APOC2-1442APOC4-4746 | APOC2-1442APOC4-4746 |
| **APOC1-5667 (rs12721054)** | **APOC1-5667 (rs12721054)** |
| **APOE-3937** | **APOE-3937** |
| *APOC2-4971* | *APOC2-4971* |
| **APOC2-853APOC4-4157** | **APOC2-853APOC4-4157** |
| *APOE-471* | *APOE-471* |
| *APOC2-2566* | *APOC2-2566*, APOC2-3030 |
| *APOC1-1870* | *APOC1-1870* |
| APOE-2440 | APOE-2440 |
| APOC2-5922 (rs10422888) | APOC2-5922 (rs10422888) |
| APOC2-1540APOC4-4844 (rs75463753) | APOC2-1540APOC4-4844 (rs75463753) |
| APOC2-3010 | APOC2-3010 |
| *APOC2-2191* | *APOC2-2191* |
| **HCR2-286** | **HCR2-286** |
| **APOC1-720** | **APOC1-720** |
| **HCR2-188** | **HCR2-188** |
| **APOC2-4853** | **APOC2-4853** |
| **APOC1-1331** | **APOC1-1331** |
| *APOC2-4493* | APOC2-4493 |
| *APOE-5229* | APOE-5229 |
| *APOC4-1823* | APOC4-1823 |
| *APOC2-850APOC4-4154* | *APOC2-850APOC4-4154* |
| **APOC2-2486 (rs9304645)** | **APOC2-2486 (rs9304645)** |
| *APOC2-3348* | *APOC2-3348* |
| APOE-832 (rs405509) | APOE-832(rs405509) |
| APOC1-3573 (rs10424339) | APOC1-3573 (rs10424339) |
| APOC4-2623 (rs5157) | APOC4-2623 (rs5157) |
| APOE-560 (rs449647) | APOE-560 (rs449647) |
| *HCR2-632* | *HCR2-632* |
| HCR1-575 | HCR1-575 |
| APOC2-3805 | APOC2-3805 |
| APOC1-5053 | APOC1-5053 |
| *APOC4-1733* | *APOC4-1733* |
| *APOC2-4534* | *APOC2-4534* |

**Bold** variants represent those genotyped successfully. *Italics* variants represent those failed genotyping or post-genotyping QC.
