# Supplementary material for: Apolipoprotein E-C1-C4-C2 gene cluster region and inter-individual variation in plasma lipoprotein levels: a comprehensive genetic association study in two ethnic groups
Source: PLoS One. 2019 Mar 26;14(3):e0214060. doi: 10.1371/journal.pone.0214060 (PMC6435132; doi:10.1371/journal.pone.0214060)
Supplement: S8 Table — Underlined variants represent those located within the sequenced regions. Bold variants represent those genotyped successfully. Italics variants represent those failed genotyping or post-genotyping QC. (DOCX) [file pone.0214060.s008.docx]

S8 Table. Tagger results for HapMap SNPs (MAF≥0.048, r^2^=0.9) covering the region of interest at 19q13.32 in CEU population.

| **Bins 1-15** | **Alleles Captured** |
| --- | --- |
| rs2288912(APOC4-4661) | rs2288912(APOC4-4661),rs7257476(APOC2-5324),rs1132899(APOC4-3498),rs2288911(APOC4-4746),  rs5157(APOC4-2623),rs5120(3778) |
| *rs5127* | rs12709889(APOC2-5398),rs1130742(APOC2-4971),*rs5127* |
| rs584007 | rs439401,rs584007 |
| *rs11083751* | *rs11083751* |
| **rs769450(APOE-2440)** | **rs769450(APOE-2440)** |
| **rs445925** | **rs445925** |
| **rs1064725 (APOC1-5641)** | **rs1064725 (APOC1-5641)** |
| **rs10421404(APOC2-5004)** | **rs10421404 (APOC2-5004)** |
| **rs405509(APOE-832)** | **rs405509 (APOE-832)** |
| **rs4420638(APOC1-6026)** | **rs4420638 (APOC1-6026)** |
| **rs5112** | **rs5112** |
| **rs7259004** | **rs7259004** |
| **rs5167(APOC4-3927)** | **rs5167(APOC4-3927)** |
| **rs5158(APOC4-2640)** | **rs5158(APOC4-2640)** |
| **rs4803770** | **rs4803770** |
| Underlined variants represent those located within the sequenced regions. **Bold** variants represent those genotyped successfully. *Italics* variants represent those failed genotyping or post-genotyping QC | |

.
