# Supplementary material for: Apolipoprotein E-C1-C4-C2 gene cluster region and inter-individual variation in plasma lipoprotein levels: a comprehensive genetic association study in two ethnic groups
Source: PLoS One. 2019 Mar 26;14(3):e0214060. doi: 10.1371/journal.pone.0214060 (PMC6435132; doi:10.1371/journal.pone.0214060)
Supplement: S9 Table — Underlined variants represent those located within the sequenced regions. Bold variants represent those genotyped successfully. Italics variants represent those failed genotyping or post-genotyping QC. (DOCX) [file pone.0214060.s009.docx]

Table S9. Tagger results for HapMap SNPs (MAF≥0.048, r^2^=0.9) covering the region of interest at 19q13.32 in YRI population.

| **Bins 1-30** | **Alleles Captured** |
| --- | --- |
| rs5127 | **rs12709889(APOC2-5398),** rs5127, rs1130742(APOC2-4971) |
| **rs5159(APOC4-2971)** | **rs12721104(APOC2-75/APOC4-3380), rs5159** |
| rs584007 | rs584007, **rs439401** |
| rs2288911(APOC4-4746) | **rs2288912 (APOC4-4661),** rs2288911 |
| **rs7259004** | **rs7259004** |
| **rs5114(APOC1-1526)** | **rs5114** |
| **rs1132899(APOC2-194)** | **rs1132899** |
| **rs10421404(APOC2-5004)** | **rs10421404** |
| **rs4420638(APOC1-6026)** | **rs4420638** |
| **rs5126 (APOC2-4587)** | **rs5126** |
| **rs769450(APOE-2440)** | **rs769450** |
| *rs11878790* | *rs11878790* |
| **rs405509(APOE-832)** | **rs405509** |
| **rs10424339(APOC1-3573)** | **rs10424339** |
| **rs5167(APOC2-623)** | **rs5167** |
| **rs445925** | **rs445925** |
| **rs5157(APOC4-2623)** | **rs5157** |
| **rs10425530(APOC2-853)** | **rs10425530** |
| **rs389261(APOC1-3423)** | **rs389261** |
| **rs5112** | **rs5112** |
| rs12709884(APOC2-850) | rs12709884 |
| **rs157599(HCR1-575)** | **rs157599** |
| **rs5155(APOC4-2559)** | **rs5155** |
| **rs5120(APOC2-3778)** | **rs5120** |
| rs7257476(APOC2-5324) | rs7257476 |
| **rs7412(APOE-4075)** | **rs7412** |
| *rs10424663* | *rs10424663* |
| **rs1081101(APOE-73)** | **rs1081101** |
| **rs12721054(APOC1-5667)** | **rs12721054** |
| **rs4803770** | **rs4803770** |

.

Underlined variants represent those located within the sequenced regions. **Bold** variants represent those genotyped successfully. *Italics* variants represent those failed genotyping or post-genotyping QC.
