# Supplementary material for: Apolipoprotein E-C1-C4-C2 gene cluster region and inter-individual variation in plasma lipoprotein levels: a comprehensive genetic association study in two ethnic groups
Source: PLoS One. 2019 Mar 26;14(3):e0214060. doi: 10.1371/journal.pone.0214060 (PMC6435132; doi:10.1371/journal.pone.0214060)
Supplement: S10 Table — HWE-P: Hardy Weinberg equilibrium, MAF: minor allele frequency, Position: chromosomal position corresponding to Chip bioinformatics database (NC_000019.9). RegulomeDB scores were generated by using http://regulome.stanford.edu/. Scores represent; “1a- eQTL + TF binding + matched TF motif + matched DNase Footprint + DNase peak; 1b- eQTL + TF binding + any motif + DNase Footprint + DNase peak; 1c- eQTL + TF binding + matched TF motif + DNase peak; 1d- eQTL + TF binding + any motif + DNase peak; 1e- eQTL + TF binding + matched TF motif; 1f- eQTL + TF binding / DNase peak; 2a- TF binding + matched TF motif + matched DNase Footprint + DNase peak; 2b- TF binding + any motif + DNase Footprint + DNase peak; 2c- TF binding + matched TF motif + DNase peak; 3a- TF binding + any motif + DNase peak; 3b- TF binding + matched TF motif; 4- TF binding + DNase peak; 5-TF binding or DNase peak; 6-other.” Selection criteria: 1) Common tagSNPs identified by Tagger analyses of sequencing data (MAF≥0.05, r2 = 0.9); 2) Rare/uncommon variants identified by sequencing (MAF<5%); 3) Additional common SNPs selected from public resources. (DOCX) [file pone.0214060.s010.docx]

S10 Table. Features of 70 QC-passed genotyped variants in NHWs (n=623)

| **Common Variants (MAF≥5%)** | | | | | | | | | | | | | | | | | | | |
| --- | --- | --- | --- | --- | --- | --- | --- | --- | --- | --- | --- | --- | --- | --- | --- | --- | --- | --- | --- |
| **#** | **Variant Name** | | | **RefSNP ID** | | **Position** | **Alleles** | | **MAF** | **HW-P** | **Call rate (%)** | **RegulomeDB score** | | | **Location** | **Gene** | | **Selection criteria** | **GT**  **Method** |
| 1 | APOE560 | | | rs449647 | | 45408564 | | A>T | 0.162 | 0.27 | 99.70 | 5 | | 5'flanking | | APOE | | 1 | TQM |
| 2 | APOE832 | | | rs405509 | | 45408836 | | G>T | 0.478 | 1.00 | 99.80 | 1f | | 5'flanking | | APOE | | 1 | IPLEX GOLD |
| 3 | APOE1163 | | | rs440446 | | 45409167 | | G>C | 0.360 | 0.47 | 100.00 | 4 | | Intron 1 | | APOE | | 1 | TQM |
| 4 | APOE1998 | | | rs769449 | | 45410002 | | G>A | 0.117 | 0.46 | 99.20 | 4 | | Intron 2 | | APOE | | 1 | IPLEX GOLD |
| 5 | APOE2440 | | | rs769450 | | 45410444 | | G>A | 0.401 | 0.51 | 99.40 | 5 | | Intron 2 | | APOE | | 1 | TQM |
| 6 | APOE3937 | | | rs429358 | | 45411941 | | T>C | 0.153 | 1.00 | 98.40 | 5 | | Exon 4 | | APOE | | 1 | IPLEX GOLD |
| 7 | APOE4075 | | | rs7412 | | 45412079 | | C>T | 0.081 | 0.81 | 99.50 | 5 | | Exon 4 | | APOE | | 1 | IPLEX GOLD |
| 8 | APOE5361 | | | rs1081106 | | 45413365 | | T>C | 0.085 | 1.00 | 99.80 | 3a | | 3'flanking | | APOE | | 1 | TQM |
| 9 | r**s439401** | | | r**s439401** | | 45414451 | | C>T | 0.359 | 0.38 | 97.80 | 1b | | Intergenic | | APOE (+6447bp) | | 3 | IPLEX GOLD |
| 10 | **APOC1rs445925** | | | **rs445925** | | 45415640 | | G>A | 0.110 | 1.00 | 99.00 | No Data | | Intergenic | | APOC1(-1281bp) | | 3 | TQM |
| 11 | APOC1p720 | | | rs11568822 | | 45417640 | | A>G | 0.231 | 0.88 | 99.80 | 4 | | 5'flanking | | APOC1 | | 1 | TQM |
| 12 | APOC1p2041 | | | rs3826688 | | 45418961 | | G>A | 0.342 | 0.76 | 97.30 | 5 | | Intron 2 | | APOC1 | | 1 | TQM |
| 13 | APOC1p4334 | | | rs12721046 | | 45421254 | | G>A | 0.153 | 0.84 | 98.10 | 6 | | Intron 3 | | APOC1 | | 1 | TQM |
| 14 | APOC1p5926 | | | rs56131196 | | 45422846 | | G>A | 0.189 | 0.51 | 99.20 | No Data | | 3'flanking | | APOC1 | | 1 | TQM |
| 15 | APOC1p6026 | | | rs4420638 | | 45422946 | | A>G | 0.156 | 0.01 | 89.30 | No Data | | 3'flanking | | APOC1 | | 1 | IPLEX GOLD |
| 16 | **rs4803770** | | | **rs4803770** | | 45427353 | | C>G | 0.377 | 0.99 | 95.40 | 5 | | Intergenic | | HCR1(-4bp) | | 3 | IPLEX GOLD |
| 17 | **rs5112** | | | **rs5112** | | 45430280 | | G>C | 0.464 | 0.98 | 91.80 | 4 | | *APOC1P1* | | HCR1(+2931bp) | | 3 | TQM |
| 18 | **rs7259004** | | | **rs7259004** | | 45432557 | | C>G | 0.117 | 1.00 | 98.20 | 6 | | *APOC1P1* | | HCR2 (-6419bp) | | 3 | TQM |
| 19 | HCR2p188 | | | rs35136575 | | 45439163 | | C>G | 0.227 | 0.24 | 97.80 | 2a | | HCR2 | | HCR2 | | 1 | IPLEX GOLD |
| 20 | APOC4p2623 | | | rs5157 | | 45447161 | | C>T | 0.497 | 0.86 | 99.80 | 4 | | Intron 1 | | APOC4 | | 1 | TQM |
| 21 | APOC4p2640 | | | rs5158 | | 45447178 | | C>T | 0.139 | 0.92 | 99.40 | 2b | | Intron 1 | | APOC4 | | 1 | TQM |
| 22 | APOC4p3498APOC2p194 | | | rs1132899 | | 45448036 | | C>T | 0.486 | 0.66 | 99.50 | 5 | | C4-Exon 2 | | APOC4 | | 1 | TQM |
| 23 | APOC4p3927APOC2p623 | | | rs5167 | | 45448465 | | T>G | 0.360 | 0.28 | 100.00 | 5 | | C4-Exon 3 | | APOC4 | | 1 | IPLEX GOLD |
| 24 | APOC4p4661APOC2p1357 | | | rs2288912 | | 45449199 | | C>G | 0.498 | 0.79 | 99.80 | 1a | | C4-3'/C2-5' | | APOC4/APOC2 | | 1 | TQM |
| 25 | APOC2p3778 | | | rs5120 | | 45451620 | | T>A | 0.498 | 0.82 | 98.90 | 4 | | Intron 1 | | APOC2 | | 1 | IPLEX GOLD |
| 26 | APOC2p4853 | | | rs199828513 | | 45452695 | | C>A | 0.279 | 0.27 | 99.20 | No Data | | 3'flanking | | APOC2 | | 1 | IPLEX GOLD |
| 27 | APOC2p5004 | | | rs10421404 | | 45452845 | | C>T | 0.183 | 0.43 | 99.00 | No Data | | 3'flanking | | APOC2 | | 1 | TQM |
| 28 | APOC2p5310 | | | rs7258345 | | 45453151 | | T>G | 0.466 | 0.92 | 98.20 | No Data | | 3'flanking | | APOC2 | | 1 | TQM |
| 29 | APOC2p5398 | | | rs12709889 | | 45453240 | | G>A | 0.276 | 0.34 | 97.10 | 6 | | 3'flanking | | APOC2 | | 1 | IPLEX GOLD |
| **Rare and less common variants (MAF<5%)** | | | | | | | | | | | | | | | | | | | |
| **#** | | **Variant Name** | **RefSNP ID** | | **Position** | | | **Alleles** | **MAF** | **HW-P** | **Call rate (%)** | | **RegulomeDB score** | | **Location** | **Gene** | **Selection criteria** | | **GT**  **Method** |
| 1 | | APOE1575 | rs769448 | | 45409579 | | | C>T | 0.021 | 0.47 | 99.50 | | 4 | | Intron 1 | APOE | 2 | | IPLEX GOLD |
| 2 | | APOE2907 | rs769451 | | 45410911 | | | T>G | 0.011 | 1.00 | 100.00 | | 5 | | Intron 2 | APOE | 2 | | IPLEX GOLD |
| 3 | | APOE3038 | rs111833428 | | 45411042 | | | G>A | 0.002 | 1.00 | 99.20 | | 5 | | Exon 3 | APOE | 2 | | IPLEX GOLD |
| 4 | | **APOE3106** | **rs769452** | | 45411110 | | | T>C | 0.001 | 1.00 | 99.70 | | 5 | | Exon 3 | APOE | 3 | | IPLEX GOLD |
| 5 | | APOE4310 | rs199768005 | | 45412314 | | | T>A | 0.004 | 1.00 | 99.80 | | 5 | | Exon 4 | APOE | 2 | | TQM |
| 6 | | APOE4528 | rs374329439 | | 45412532 | | | C>T | 0.001 | 1.00 | 100.00 | | 5 | | 3' UTR | APOE | 2 | | IPLEX GOLD |
| 7 | | APOE4737 | rs117656888 | | 45412741 | | | C>G | 0.008 | 1.00 | 99.50 | | 5 | | 3'flanking | APOE | 2 | | IPLEX GOLD |
| 8 | | APOC1p698 | rs72654449 | | 45417618 | | | C>A | 0.004 | 1.00 | 99.20 | | 4 | | 5'flanking | APOC1 | 2 | | TQM |
| 9 | | APOC1p703 | rs3207187 | | 45417623 | | | C>T | 0.001 | 1.00 | 99.50 | | 4 | | 5'flanking | APOC1 | 2 | | TQM |
| 10 | | APOC1p1170 | rs777637891 | | 45418090 | | | G>A | 0.001 | 1.00 | 97.90 | | 2b | | Intron 1 | APOC1 | 2 | | TQM |
| 11 | | APOC1p1294 | rs757917286 | | 45418214 | | | A>C | 0.001 | 1.00 | 99.40 | | 4 | | Intron 2 | APOC1 | 2 | | TQM |
| 12 | | APOC1p1317 | rs12721048 | | 45418237 | | | G>A | 0.002 | 1.00 | 97.80 | | 4 | | Intron 2 | APOC1 | 2 | | IPLEX GOLD |
| 13 | | APOC1p1422 | rs765806814 | | 45418342 | | | G>A | 0.002 | 1.00 | 100.00 | | 4 | | Intron 2 | APOC1 | 2 | | TQM |
| 14 | | APOC1p1566 | rs12691088 | | 45418486 | | | G>A | 0.006 | 1.00 | 97.10 | | 2b | | Intron 2 | APOC1 | 2 | | IPLEX GOLD |
| 15 | | APOC1p2629 | rs369438021 | | 45419549 | | | G>A | 0.001 | 1.00 | 99.40 | | 4 | | Exon 3 | APOC1 | 2 | | TQM |
| 16 | | APOC1p2817 | rs555051945 | | 45419737 | | | C>T | 0.003 | 1.00 | 97.40 | | 2b | | Intron 3 | APOC1 | 2 | | IPLEX GOLD |
| 17 | | APOC1p3423 | rs389261 | | 45420343 | | | G>A | 0.002 | 1.00 | 97.60 | | No Data | | Intron 3 | APOC1 | 2 | | IPLEX GOLD |
| 18 | | APOC1p3494 | rs559574042 | | 45420414 | | | C>T | 0.002 | 1.00 | 99.70 | | No Data | | Intron 3 | APOC1 | 2 | | IPLEX GOLD |
| 19 | | APOC1p5641 | rs1064725 | | 45422561 | | | T>G | 0.039 | 1.00 | 99.20 | | No Data | | 3'UTR | APOC1 | 2 | | IPLEX GOLD |
| 20 | | APOC1p5773 | rs568313508 | | 45422693 | | | G>A | 0.001 | 1.00 | 97.10 | | No Data | | 3'flanking | APOC1 | 2 | | IPLEX GOLD |
| 21 | | HCR1p292 | rs4803771 | | 45427648 | | | C>G | 0.024 | 0.61 | 98.20 | | 4 | | HCR1 | HCR1 | 2 | | IPLEX GOLD |
| 22 | | HCR1p362 | rs557378991 | | 45427718 | | | C>A | 0.002 | 1.00 | 97.60 | | 2a | | HCR1 | HCR1 | 2 | | IPLEX GOLD |
| 23 | | HCR1p423 | rs117664574 | | 45427779 | | | C>G | 0.026 | 0.67 | 99.50 | | 4 | | HCR1 | HCR1 | 2 | | IPLEX GOLD |
| 24 | | HCR1p575 | rs157599 | | 45427931 | | | A>G | 0.002 | 1.00 | 99.70 | | 3a | | HCR1 | HCR1 | 2 | | IPLEX GOLD |
| 25 | | HCR1p727 | rs149345 | | 45428083 | | | T>G | 0.002 | 1.00 | 98.60 | | 3a | | HCR1 | HCR1 | 2 | | TQM |
| 26 | | HCR2p365 | rs539409314 | | 45439340 | | | C>A | 0.004 | 1.00 | 98.10 | | 2b | | HCR2 | HCR2 | 2 | | IPLEX GOLD |
| 27 | | HCR2p523 | rs118004808 | | 45439498 | | | C>T | 0.023 | 1.00 | 96.00 | | 2b | | HCR2 | HCR2 | 2 | | IPLEX GOLD |
| 28 | | APOC4p636 | rs371539058 | | 45445174 | | | C>T | 0.001 | 1.00 | 96.60 | | No Data | | 5’ flanking | APOC4 | 2 | | TQM |
| 29 | | APOC4p968 | rs76214972 | | 45445506 | | | A>G | 0.036 | 0.88 | 99.70 | | 4 | | 5’ UTR | APOC4 | 2 | | IPLEX GOLD |
| 30 | | APOC4p1150 | rs148247675 | | 45445688 | | | A>G | 0.002 | 1.00 | 96.80 | | 5 | | Intron 1 | APOC4 | 2 | | IPLEX GOLD |
| 31 | | APOC4p1229 | rs370742602 | | 45445767 | | | G>C | 0.002 | 1.00 | 99.70 | | 2b | | Intron 1 | APOC4 | 2 | | IPLEX GOLD |
| 32 | | APOC4p2557 | rs775530121 | | 45447095 | | | C>A | 0.001 | 1.00 | 99.50 | | 4 | | Intron 1 | APOC4 | 2 | | TQM |
| 33 | | APOC4p2683 | rs12721109 | | 45447221 | | | G>A | 0.024 | 0.61 | 98.20 | | 2b | | Intron 1 | APOC4 | 2 | | IPLEX GOLD |
| 34 | | **APOC4p2703** | **rs12721108** | | 45447241 | | | G>T | 0.008 | 1.00 | 99.40 | | 2a | | Intron 1 | APOC4 | 3 | | IPLEX GOLD |
| 35 | | APOC4p3546APOC2p242 | rs12691089 | | 45448084 | | | G>A | 0.003 | 1.00 | 99.70 | | 5 | | C4-Exon 2 | APOC4 | 2 | | IPLEX GOLD |
| 36 | | APOC4p3847APOC2p543 | rs186448850 | | 45448385 | | | T>C | 0.002 | 1.00 | 98.10 | | 5 | | C4-Intron 2 | APOC4 | 2 | | IPLEX GOLD |
| 37 | | APOC4p4895APOC2p1591 | rs186448850 | | 45449433 | | | G>A | 0.001 | 1.00 | 99.70 | | 4 | | C2-Intron 1 | APOC2 | 2 | | IPLEX GOLD |
| 38 | | APOC2p1851 | rs12709886 | | 45449693 | | | G>A | 0.037 | 0.84 | 99.20 | | 6 | | Intron 1 | APOC2 | 2 | | TQM |
| 39 | | APOC2p2870 | rs559706335 | | 45450712 | | | G>T | 0.004 | 1.00 | 99.70 | | 4 | | Intron 1 | APOC2 | 2 | | IPLEX GOLD |
| 40 | | APOC2p3348 | rs10420434 | | 45451190 | | | G>A | 0.037 | 0.84 | 99.50 | | No Data | | Intron 1 | APOC2 | 2 | | TQM |
| 41 | | APOC2p5644 | rs112144355 | | 45453486 | | | G>A | 0.009 | 1.00 | 95.70 | | 6 | | 3'flanking | APOC2 | 2 | | IPLEX GOLD |
| HWE-P: Hardy Weinberg equilibrium, MAF: minor allele frequency, Position: chromosomal position corresponding to Chip bioinformatics database ([NC_000019.9](https://www.ncbi.nlm.nih.gov/projects/sviewer/?id=NC_000019.9&search=NC_000019.9:g.45411042G%3EA&v=1:100&content=5" \t )), GT method: Methods used for genotyping, TQM: TaqMan, IPLEX GOLD: Sequenom  RegulomeDB scores were generated by using <http://regulome.stanford.edu/>. Scores represent; “1a- eQTL + TF binding + matched TF motif + matched DNase Footprint + DNase peak; 1b- eQTL + TF binding + any motif + DNase Footprint + DNase peak; 1c- eQTL + TF binding + matched TF motif + DNase peak; 1d- eQTL + TF binding + any motif + DNase peak; 1e- eQTL + TF binding + matched TF motif; 1f- eQTL + TF binding / DNase peak; 2a- TF binding + matched TF motif + matched DNase Footprint + DNase peak; 2b- TF binding + any motif + DNase Footprint + DNase peak; 2c- TF binding + matched TF motif + DNase peak; 3a- TF binding + any motif + DNase peak; 3b- TF binding + matched TF motif; 4- TF binding + DNase peak; 5-TF binding or DNase peak; 6-other.”  Selection criteria: 1) Common tagSNPs identified by Tagger analyses of sequencing data (MAF≥0.05, r^2^=0.9); 2) Rare/uncommon variants identified by sequencing (MAF<5%); 3) Additional common SNPs selected from public resources. | | | | | | | | | | | | | | | | | | | |
