# Supplementary material for: Apolipoprotein E-C1-C4-C2 gene cluster region and inter-individual variation in plasma lipoprotein levels: a comprehensive genetic association study in two ethnic groups
Source: PLoS One. 2019 Mar 26;14(3):e0214060. doi: 10.1371/journal.pone.0214060 (PMC6435132; doi:10.1371/journal.pone.0214060)
Supplement: S11 Table — HWE-P: Hardy Weinberg equilibrium, MAF: minor allele frequency, Position: chromosomal position corresponding to Chip bioinformatics database (NC_000019.9). RegulomeDB scores were generated by using http://regulome.stanford.edu/. Scores represents; 1a- eQTL + TF binding + matched TF motif + matched DNase Footprint + DNase peak; 1b- eQTL + TF binding + any motif + DNase Footprint + DNase peak; 1c- eQTL + TF binding + matched TF motif + DNase peak; 1d- eQTL + TF binding + any motif + DNase peak; 1e- eQTL + TF binding + matched TF motif; 1f- eQTL + TF binding / DNase peak; 2a- TF binding + matched TF motif + matched DNase Footprint + DNase peak; 2b- TF binding + any motif + DNase Footprint + DNase peak; 2c- TF binding + matched TF motif + DNase peak; 3a- TF binding + any motif + DNase peak; 3b- TF binding + matched TF motif; 4- TF binding + DNase peak; 5-TF binding or DNase peak; 6-other. Selection criteria: 1) Common tagSNPs identified by Tagger analyses of sequencing data (MAF≥0.05, r2 = 0.9); 2) Rare/uncommon variants identified by sequencing (MAF<5%); 3) Additional common SNPs selected from public resources. (DOCX) [file pone.0214060.s011.docx]

S11 Table. Features of 108 QC-passed genotyped variants in ABs (n=788)

| **Common Variants with (MAF≥5%)** | | | | | | | | | | | | | | | | | | | | |
| --- | --- | --- | --- | --- | --- | --- | --- | --- | --- | --- | --- | --- | --- | --- | --- | --- | --- | --- | --- | --- |
| **#** | **Variant name** | **RefSNP ID** | **Position** | | **Alleles** | | **MAF** | | **HWE-P** | | **Call rate (%)** | | **RegulomeDB score** | | **Location** | | **Gene** | | **Selection criteria** | **GT method** |
| 1 | APOE73 | rs1081101 | 45408077 | | C>T | | 0.061 | | 0.284 | | 99.3 | | 4 | | 5'flanking | | APOE | | 1 | IPLEX GOLD |
| 2 | APOE560 | rs449647 | 45408564 | | A>T | | 0.374 | | 0.498 | | 96.6 | | 5 | | 5'flanking | | APOE | | 1 | IPLEX GOLD |
| 3 | APOE832 | rs405509 | 45408836 | | G>T | | 0.253 | | 0.128 | | 99.0 | | 1f | | 5'flanking | | APOE | | 1 | IPLEX GOLD |
| 4 | APOE1163 | rs440446 | 45409167 | | G>C | | 0.100 | | 0.862 | | 93.6 | | 4 | | Intron 1 | | APOE | | 1 | IPLEX GOLD |
| 5 | APOE1279 | rs877973 | 45409283 | | C>A | | 0.060 | | 0.557 | | 99.5 | | 4 | | Intron 1 | | APOE | | 1 | IPLEX GOLD |
| 6 | APOE2440 | rs769450 | 45410444 | | G>A | | 0.387 | | 0.241 | | 90.1 | | 5 | | Intron 2 | | APOE | | 1 | IPLEX GOLD |
| 7 | APOE3937 | rs429358 | 45411941 | | T>C | | 0.266 | | 0.659 | | 98.0 | | 5 | | Exon 4 | | APOE | | 1 | IPLEX GOLD |
| 8 | APOE407 | rs7412 | 45412079 | | G>A | | 0.061 | | 1.000 | | 99.0 | | 5 | | Exon 4 | | APOE | | 1 | TQM |
| 9 | **rs439401** | **rs439401** | 45414451 | | C>T | | 0.109 | | 0.043 | | 97.4 | | 1b | | Intergenic | | APOE(+6447bp) | | 3 | IPLEX GOLD |
| 10 | **APOC1rs445925** | **rs445925** | 45415640 | | G>A | | 0.297 | | 0.800 | | 97.7 | | No Data | | Intergenic | | APOC1(-1281bp) | | 3 | TQM |
| 11 | APOC1p720ins4 | rs11568822 | 45417640 | | A>G | | 0.274 | | 0.233 | | 97.8 | | 4 | | 5'flanking | | APOC1 | | 1 | IPLEX GOLD |
| 12 | APOC1p1331 | rs10408994 | 45418251 | | G>A | | 0.067 | | 0.059 | | 98.0 | | 4 | | Intron 2 | | APOC1 | | 1 | IPLEX GOLD |
| 13 | APOC1p1526 | rs5114 | 45418446 | | C>T | | 0.058 | | 0.467 | | 98.0 | | 4 | | Intron 2 | | APOC1 | | 1 | IPLEX GOLD |
| 14 | APOC1p1684 | rs12709881 | 45418604 | | G>A | | 0.098 | | 0.564 | | 99.0 | | 4 | | Intron 2 | | APOC1 | | 1 | TQM |
| 15 | APOC1p3423 | rs389261 | 45420343 | | G>A | | 0.331 | | 0.980 | | 95.0 | | No Data | | Intron 3 | | APOC1 | | 1 | IPLEX GOLD |
| 16 | APOC1p3573 | rs10424339 | 45420493 | | G>A | | 0.140 | | 0.732 | | 97.3 | | No Data | | Intron 3 | | APOC1 | | 1 | IPLEX GOLD |
| 17 | APOC1p5006 | rs112528434 | 45421926 | | G>T | | 0.086 | | 0.436 | | 89.3 | | No Data | | Intron 3 | | APOC1 | | 1 | TQM |
| 18 | APOC1p5053 | rs12721052 | 45421973 | | A>C | | 0.223 | | 0.395 | | 98.7 | | No Data | | Intron 3 | | APOC1 | | 1 | TQM |
| 19 | APOC1p5667 | rs12721054 | 45422587 | | A>G | | 0.145 | | 0.238 | | 91.6 | | 6 | | 3'UTR | | APOC1 | | 1 | IPLEX GOLD |
| 20 | APOC1p5926 | rs56131196 | 45422846 | | G>A | | 0.175 | | 0.806 | | 96.9 | | No Data | | 3'flanking | | APOC1 | | 1 | IPLEX GOLD |
| 21 | **rs4803770** | **rs4803770** | 45427353 | | C>G | | 0.270 | | 0.489 | | 96.9 | | 5 | | Intergenic | | HCR1(-4bp) | | 3 | IPLEX GOLD |
| 22 | HCR1p575 | rs157599 | 45427931 | | A>G | | 0.357 | | 0.983 | | 89.6 | | 3a | | HCR1 | | HCR1 | | 1 | TQM |
| 23 | **rs5112** | **rs5112** | 45430280 | | C>G | | 0.477 | | 0.211 | | 91.4 | | 4 | | *APOC1P1* | | HCR1(+2931bp) | | 3 | TQM |
| 24 | **rs7259004** | **rs7259004** | 45432557 | | G>C | | 0.302 | | 0.134 | | 97.3 | | 6 | | *APOC1P1* | | HCR2 (-6419bp) | | 3 | IPLEX GOLD |
| 25 | HCR2p188 | rs35136575 | 45439163 | | C>G | | 0.155 | | 0.067 | | 97.5 | | 2a | | HCR2 | | HCR2 | | 1 | IPLEX GOLD |
| 26 | APOC4p2559 | rs5155 | 45447097 | | C>T | | 0.099 | | 1.000 | | 98.7 | | 4 | | Intron 1 | | APOC4 | | 1 | TQM |
| 27 | APOC4p2623 | rs5157 | 45447161 | | C>T | | 0.173 | | 0.627 | | 97.9 | | 4 | | Intron 1 | | APOC4 | | 1 | TQM |
| 28 | APOC2p75APOC4p3380 | rs12721104 | 45447918 | | G>A | | 0.138 | | 1.000 | | 98.2 | | 5 | | C4-Intron 1 | | APOC4 | | 1 | TQM |
| 29 | APOC2p194APOC4p3498 | rs1132899 | 45448036 | | C>T | | 0.239 | | 0.604 | | 99.0 | | 5 | | C4-Exon 2 | | APOC4 | | 1 | TQM |
| 30 | APOC2p623APOC4p3927 | rs5167 | 45448465 | | T>G | | 0.459 | | 0.597 | | 98.6 | | 5 | | C4-Exon 3 | | APOC4 | | 1 | TQM |
| 31 | APOC2p853APOC4p4157 | rs10425530 | 45448695 | | G>A | | 0.111 | | 0.530 | | 97.8 | | 6 | | C4-3' UTR | | APOC4 | | 1 | TQM |
| 32 | APOC2p1357APOC4p4661 | rs2288912 | 45449199 | | G>C | | 0.257 | | 0.882 | | 97.9 | | 1a | | C4-3'/C2-5' | | APOC2/APOC4 | | 1 | TQM |
| 33 | APOC2p1540APOC4p4844 | rs75463753 | 45449382 | | G>A | | 0.108 | | 0.365 | | 92.6 | | 4 | | C2-Intron 1 | | APOC2 | | 1 | IPLEX GOLD |
| 34 | APOC2p2486 | rs9304645 | 45450328 | | G>A | | 0.368 | | 0.094 | | 97.5 | | 4 | | Intron 1 | | APOC2 | | 1 | TQM |
| 35 | APOC2p3010 | rs10419086 | 45450852 | | A>G | | 0.124 | | 0.539 | | 92.2 | | 6 | | Intron 1 | | APOC2 | | 1 | TQM |
| 36 | APOC2p3778 | rs5120 | 45451620 | | A>T | | 0.182 | | 0.384 | | 98.8 | | 4 | | Intron 1 | | APOC2 | | 1 | TQM |
| 37 | APOC2p3805 | rs7257095 | 45451647 | | C>G | | 0.165 | | 0.303 | | 98.2 | | 2a | | Intron 1 | | APOC2 | | 1 | IPLEX GOLD |
| 38 | APOC2p3814 | rs10422603 | 45451656 | | T>G | | 0.301 | | 0.706 | | 96.3 | | 2b | | Intron 1 | | APOC2 | | 1 | IPLEX GOLD |
| 39 | APOC2p4319 | rs5123 | 45452161 | | G>A | | 0.059 | | 0.074 | | 97.0 | | No Data | | Intron 3 | | APOC2 | | 1 | IPLEX GOLD |
| 40 | APOC2p4587 | rs5126 | 45452429 | | A>C | | 0.050 | | 0.918 | | 94.1 | | 5 | | Exon 4 | | APOC2 | | 1 | IPLEX GOLD |
| 41 | APOC2p4754 | rs7253690 | 45452596 | | G>A | | 0.061 | | 0.101 | | 98.8 | | 5 | | Exon 4 | | APOC2 | | 1 | TQM |
| 42 | APOC2p4853 | rs150448996 | 45452694 | | delT | | 0.274 | | 0.822 | | 96.6 | | No Data | | 3'flanking | | APOC2 | | 1 | IPLEX GOLD |
| 43 | APOC2p5004 | rs10421404 | 45452845 | | C>T | | 0.293 | | 0.170 | | 98.2 | | No Data | | 3'flanking | | APOC2 | | 1 | TQM |
| 44 | APOC2p5310 | rs7258345 | 45453151 | | G>T | | 0.305 | | 0.192 | | 90.9 | | No Data | | 3'flanking | | APOC2 | | 1 | TQM |
| 45 | APOC2p5398 | rs12709889 | 45453239 | | G>A | | 0.259 | | 1.000 | | 97.7 | | 6 | | 3'flanking | | APOC2 | | 1 | IPLEX GOLD |
| 46 | APOC2p5586 | rs73558127 | 45453427 | | T>G | | 0.100 | | 0.376 | | 96.5 | | No Data | | 3'flanking | | APOC2 | | 1 | IPLEX GOLD |
| 47 | APOC2p5815 | rs10423208 | 45453656 | | A>G | | 0.316 | | 0.818 | | 97.8 | | 5 | | 3'flanking | | APOC2 | | 1 | IPLEX GOLD |
| 48 | APOC2p5922 | rs10422888 | 45453763 | | A>G | | 0.078 | | 0.891 | | 93.2 | | 5 | | 3'flanking | | APOC2 | | 1 | IPLEX GOLD |
| **Rare and less common variants (MAF<5%)** | | | | | | | | | | | | | | | | | | | | |
| **#** | **Variant name** | **RefSNP ID** | | **Position** | | **Alleles** | | **MAF** | | **HWE-P** | | **Call rate (%)** | | **RegulomeDB score** | | **Location** | | **Gene** | **Selection criteria** | **GT method** |
| 1 | APOE173 | **rs546268923** | | 45408177 | | A>G | | 0.002 | | 1.000 | | 99.6 | | 3a | | 5'flanking | | APOE | 2 | IPLEX GOLD |
| 2 | APOE308 | rs769445 | | 45408312 | | C>T | | 0.007 | | 1.000 | | 99.6 | | 4 | | 5'flanking | | APOE | 2 | IPLEX GOLD |
| 3 | APOE618 | rs756735196 | | 45408622 | | G>C | | 0.001 | | 1.000 | | 99.6 | | 4 | | 5'flanking | | APOE | 2 | TQM |
| 4 | APOE624 | rs769446 | | 45408628 | | T>C | | 0.008 | | 1.000 | | 91.9 | | 3a | | 5'flanking | | APOE | 2 | TQM |
| 5 | APOE1109 | rs9282609 | | 45409113 | | C>T | | 0.042 | | 0.070 | | 99.1 | | 4 | | Splice site | | APOE | 2 | IPLEX GOLD |
| 6 | APOE1231 | rs545943117 | | 45409235 | | G>A | | 0.012 | | 1.000 | | 99.5 | | 2b | | Intron 1 | | APOE | 2 | IPLEX GOLD |
| 7 | APOE1539 | rs184686013 | | 45409543 | | A>G | | 0.009 | | 0.101 | | 99.1 | | 4 | | Intron 1 | | APOE | 2 | IPLEX GOLD |
| 8 | APOE2072 | rs189660912 | | 45410076 | | G>A | | 0.008 | | 1.000 | | 99.2 | | 4 | | Intron 2 | | APOE | 2 | IPLEX GOLD |
| 9 | APOE2269 | rs61357706 | | 45410273 | | G>A | | 0.017 | | 1.000 | | 98.0 | | 5 | | Intron 2 | | APOE | 2 | TQM |
| 10 | APOE3673 | rs769453 | | 45411677 | | C>G | | 0.007 | | 1.000 | | 99.3 | | 5 | | Intron 3 | | APOE | 2 | IPLEX GOLD |
| 11 | APOE4036 | rs769455 | | 45412040 | | C>T | | 0.020 | | 0.512 | | 97.9 | | 5 | | Exon 4 | | APOE | 2 | IPLEX GOLD |
| 12 | APOE4569 | rs762567388 | | 45412573 | | G>T | | 0.001 | | 1.000 | | 99.3 | | 5 | | 3'UTR | | APOE | 2 | IPLEX GOLD |
| 13 | APOE5223 | rs771320440 | | 45413227 | | C>G | | 0.005 | | 1.000 | | 100.0 | | 2b | | 3'flanking | | APOE | 2 | TQM |
| 14 | APOE5231 | rs747405425 | | 45413235 | | T>G | | 0.027 | | 0.197 | | 99.2 | | 2b | | 3'flanking | | APOE | 2 | IPLEX GOLD |
| 15 | APOC1p894 | rs190454394 | | 45417814 | | C>T | | 0.002 | | 1.000 | | 98.8 | | 4 | | 5'flanking | | APOC1 | 2 | IPLEX GOLD |
| 16 | APOC1p1166 | rs72654452 | | 45418086 | | C>T | | 0.031 | | 0.334 | | 99.5 | | 2b | | Intron 1 | | APOC1 | 2 | TQM |
| 17 | APOC1p1642 | rs568979138 | | 45418562 | | C>T | | 0.011 | | 1.000 | | 99.3 | | 4 | | Intron 2 | | APOC1 | 2 | TQM |
| 18 | APOC1p3358 | rs527446270 | | 45420278 | | A>G | | 0.002 | | 1.000 | | 94.3 | | No Data | | Intron 3 | | APOC1 | 2 | IPLEX GOLD |
| 19 | HCR1p424 | rs117664574 | | 45427779 | | G>A | | 0.007 | | 1.000 | | 99.0 | | 4 | | HCR1 | | HCR1 | 2 | IPLEX GOLD |
| 20 | HCR2p286 | rs150849246 | | 45439261 | | G>A | | 0.046 | | 0.399 | | 98.6 | | 2a | | HCR2 | | HCR2 | 2 | IPLEX GOLD |
| 21 | HCR2p523 | rs118004808 | | 45439498 | | C>T | | 0.003 | | 1.000 | | 99.2 | | 2b | | HCR2 | | HCR2 | 2 | IPLEX GOLD |
| 22 | APOC4p368 | rs559795225 | | 45444906 | | T>C | | 0.002 | | 1.000 | | 98.3 | | 6 | | 5’ flanking | | APOC4 | 2 | TQM |
| 23 | APOC4p637 | rs113814026 | | 45445175 | | G>T | | 0.046 | | 1.000 | | 98.7 | | No Data | | 5’ flanking | | APOC4 | 2 | TQM |
| 24 | APOC4p757 | rs12721105 | | 45445295 | | G>T | | 0.038 | | 0.591 | | 99.7 | | 5 | | 5’ flanking | | APOC4 | 2 | TQM |
| 25 | APOC4p1088 | rs367589753 | | 45445626 | | T>G | | 0.001 | | 1.000 | | 98.2 | | 2b | | Intron 1 | | APOC4 | 2 | IPLEX GOLD |
| 26 | APOC4p1130 | rs751337487 | | 45445668 | | T>C | | 0.001 | | 1.000 | | 99.1 | | 5 | | Intron 1 | | APOC4 | 2 | IPLEX GOLD |
| 27 | APOC4p1192 | rs113745034 | | 45445730 | | G>A | | 0.012 | | 1.000 | | 94.9 | | 4 | | Intron 1 | | APOC4 | 2 | IPLEX GOLD |
| 28 | APOC4p1325del3 | rs79213911 | | 45445863 | | A>C | | 0.024 | | 1.000 | | 98.7 | | 4 | | Intron 1 | | APOC4 | 2 | IPLEX GOLD |
| 29 | APOC4p1430insG | rs763894138 | | 45445968 | | A>G | | 0.034 | | 1.000 | | 87.7 | | 5 | | Intron 1 | | APOC4 | 2 | TQM |
| 30 | APOC4p2099 | rs111339708 | | 45446637 | | G>T | | 0.015 | | 1.000 | | 99.0 | | No Data | | Intron 1 | | APOC4 | 2 | TQM |
| 31 | APOC4p2467 | rs115225947 | | 45447005 | | G>A | | 0.014 | | 1.000 | | 99.1 | | 5 | | Intron 1 | | APOC4 | 2 | TQM |
| 32 | APOC4p2607 | rs5156 | | 45447145 | | G>A | | 0.013 | | 1.000 | | 96.0 | | 4 | | Intron 1 | | APOC4 | 2 | IPLEX GOLD |
| 33 | APOC4p2640 | rs5158 | | 45447178 | | C>T | | 0.021 | | 1.000 | | 98.7 | | 2b | | Intron 1 | | APOC4 | 2 | TQM |
| 34 | APOC4p2678 | rs148564866 | | 45447216 | | G>C | | 0.009 | | 1.000 | | 98.3 | | 2b | | Intron 1 | | APOC4 | 2 | IPLEX GOLD |
| 35 | APOC4p2767 | rs127721107 | | 45447305 | | G>T | | 0.025 | | 1.000 | | 97.8 | | 4 | | Intron 1 | | APOC4 | 2 | IPLEX GOLD |
| 36 | APOC4p3348 | rs769339360 | | 45447886 | | G>A | | 0.001 | | 1.000 | | 98.8 | | 5 | | Intron 1 | | APOC4 | 2 | IPLEX GOLD |
| 37 | APOC2p228 | rs5164 | | 45448070 | | G>A | | 0.007 | | 1.000 | | 98.4 | | 5 | | C4-Exon 2 | | APOC4 | 2 | IPLEX GOLD |
| 38 | APOC2p288APOC4p3592 | rs12691090 | | 45448130 | | C>T | | 0.027 | | 1.000 | | 98.3 | | 5 | | C4-Exon 2 | | APOC4 | 2 | IPLEX GOLD |
| 39 | APOC2p396APOC4p3700 | rs777229733 | | 45448238 | | G>A | | 0.001 | | 1.000 | | 95.8 | | 5 | | C4-Intron 2 | | APOC4 | 2 | IPLEX GOLD |
| 40 | APOC2p488APOC4p3792 | rs5165 | | 45448330 | | G>A | | 0.015 | | 1.000 | | 98.0 | | 5 | | C4-Intron 2 | | APOC4 | 2 | IPLEX GOLD |
| 41 | APOC2p665APOC4p3969 | rs138548797 | | 45448507 | | A>C | | 0.009 | | 1.000 | | 98.7 | | No Data | | C4-Exon 3 | | APOC4 | 2 | IPLEX GOLD |
| 42 | APOC2p708APOC4p4012 | rs757860005 | | 45448550 | | G>A | | 0.001 | | 1.000 | | 98.4 | | 6 | | C4-Exon 3 | | APOC4 | 2 | IPLEX GOLD |
| 43 | APOC2p1042APOC4p4346 | rs12709885 | | 45448884 | | A>T | | 0.018 | | 0.420 | | 99.0 | | 5 | | C4-3'/C2-5' | | APOC2/APOC4 | 2 | IPLEX GOLD |
| 44 | APOC2p1187APOC4p4491 | rs111782345 | | 45449029 | | G>A | | 0.018 | | 1.000 | | 95.3 | | 5 | | C4-3'/C2-5' | | APOC2/APOC4 | 2 | IPLEX GOLD |
| 45 | APOC2p1229APOC4p4533 | rs112698600 | | 45449071 | | C>T | | 0.014 | | 1.000 | | 97.8 | | 2b | | C4-3'/C2-5' | | APOC2/APOC4 | 2 | IPLEX GOLD |
| 46 | APOC2p1275APOC4p4579 | rs111356234 | | 45449117 | | G>A | | 0.035 | | 0.775 | | 98.3 | | 4 | | C4-3'/C2-5' | | APOC2/APOC4 | 2 | IPLEX GOLD |
| 47 | APOC2p2935 | rs11879392 | | 45450777 | | C>G | | 0.014 | | 1.000 | | 96.6 | | 2b | | Intron 1 | | APOC2 | 2 | IPLEX GOLD |
| 48 | APOC2p3692 | rs12721060 | | 45451534 | | T>G | | 0.017 | | 1.000 | | 87.3 | | No Data | | Intron 1 | | APOC2 | 2 | IPLEX GOLD |
| 49 | APOC2p3892 | rs5121 | | 45451734 | | C>T | | 0.036 | | 1.000 | | 96.6 | | 5 | | Exon 2 | | APOC2 | 2 | IPLEX GOLD |
| 50 | APOC2p4086 | rs114780592 | | 45451928 | | G>A | | 0.028 | | 1.000 | | 98.7 | | 4 | | Intron 2 | | APOC2 | 2 | IPLEX GOLD |
| 51 | APOC2p4118 | rs201709243 | | 45451960 | | G>A | | 0.001 | | 1.000 | | 96.2 | | 4 | | Exon 3 | | APOC2 | 2 | IPLEX GOLD |
| 52 | APOC2p4513 | rs180809422 | | 45452355 | | A>C | | 0.014 | | 0.235 | | 90.3 | | 5 | | Intron 3 | | APOC2 | 2 | TQM |
| 53 | APOC2p4973 | rs199828513 | | 45452814 | | A>G | | 0.008 | | 1.000 | | 93.5 | | No Data | | 3'flanking | | APOC2 | 2 | TQM |
| 54 | APOC2p5018 | rs78403558 | | 45452859 | | A>C | | 0.035 | | 1.000 | | 99.3 | | 5 | | 3'flanking | | APOC2 | 2 | TQM |
| 55 | APOC2p5491 | rs190382225 | | 45453332 | | C>T | | 0.001 | | 1.000 | | 98.8 | | 6 | | 3'flanking | | APOC2 | 2 | IPLEX GOLD |
| 56 | APOC2p5512 | rs12721064 | | 45453353 | | C>T | | 0.008 | | 1.000 | | 100.0 | | 6 | | 3'flanking | | APOC2 | 2 | TQM |
| 57 | APOC2p5562 | rs138369841 | | 45453403 | | G>C | | 0.018 | | 1.000 | | 96.7 | | No Data | | 3'flanking | | APOC2 | 2 | IPLEX GOLD |
| 58 | APOC2p5771 | rs750595202 | | 45453612 | | A>C | | 0.005 | | 1.000 | | 97.1 | | 6 | | 3'flanking | | APOC2 | 2 | IPLEX GOLD |
| 59 | APOC2p5965 | rs531834248 | | 45453806 | | G>A | | 0.001 | | 1.000 | | 99.1 | | 5 | | 3'flanking | | APOC2 | 2 | IPLEX GOLD |
| 60 | APOC2p6334 | rs573344137 | | 45454175 | | G>A | | 0.009 | | 1.000 | | 99.5 | | No Data | | 3'flanking | | APOC2 | 2 | TQM |
| HWE-P: Hardy Weinberg equilibrium, MAF: minor allele frequency, Position: chromosomal position corresponding to Chip bioinformatics database ([NC_000019.9](https://www.ncbi.nlm.nih.gov/projects/sviewer/?id=NC_000019.9&search=NC_000019.9:g.45411042G%3EA&v=1:100&content=5" \t )), GT method: Methods used for genotyping, TQM: TaqMan, IPLEX GOLD: Sequenom  RegulomeDB scores were generated by using <http://regulome.stanford.edu/>. Scores represents; 1a- eQTL + TF binding + matched TF motif + matched DNase Footprint + DNase peak; 1b- eQTL + TF binding + any motif + DNase Footprint + DNase peak; 1c- eQTL + TF binding + matched TF motif + DNase peak; 1d- eQTL + TF binding + any motif + DNase peak; 1e- eQTL + TF binding + matched TF motif; 1f- eQTL + TF binding / DNase peak; 2a- TF binding + matched TF motif + matched DNase Footprint + DNase peak; 2b- TF binding + any motif + DNase Footprint + DNase peak; 2c- TF binding + matched TF motif + DNase peak; 3a- TF binding + any motif + DNase peak; 3b- TF binding + matched TF motif; 4- TF binding + DNase peak; 5-TF binding or DNase peak; 6-other.  Selection criteria: 1) Common tagSNPs identified by Tagger analyses of sequencing data (MAF≥0.05, r^2^=0.9); 2) Rare/uncommon variants identified by sequencing (MAF<5%); 3) Additional common SNPs selected from public resources. | | | | | | | | | | | | | | | | | | | | |
