# Supplementary material for: Apolipoprotein E-C1-C4-C2 gene cluster region and inter-individual variation in plasma lipoprotein levels: a comprehensive genetic association study in two ethnic groups
Source: PLoS One. 2019 Mar 26;14(3):e0214060. doi: 10.1371/journal.pone.0214060 (PMC6435132; doi:10.1371/journal.pone.0214060)
Supplement: S12 Table — MAF is the minor allele frequency; GT is genotype; GT count is the number of individuals in each genotype group; GT_SD is standard deviation of the lipid trait in each genotype group;. *Adjusted for relevant covariates, **Adjusted for APOE*2/E*4 SNPs in addition to the covariates. (DOCX) [file pone.0214060.s012.docx]

S12 Table. Single-site association analysis results for LDL-C levels in NHWs (n=623)

| **Variant Name/RefSNP ID** | **Location** | **Genotype** | **GT Count** | **MAF** | **Adjusted Mean of plasma LDL-C*** | **GT_SD*** | **Beta*** | **P*** | **Adj. B**** | **Adj. P**** |
| --- | --- | --- | --- | --- | --- | --- | --- | --- | --- | --- |
| APOE560/rs449647 | 5'flanking | AA/AT/TT | 433/176/12 | 0.1610 | 139.19/132.29/124.15 | 38.8/45.3/36.3 | -7.1 | 0.025 | -0.784 | 0.815 |
| APOE832/rs405509 | 5'flanking | GG/GT/TT | 170/310/142 | 0.4775 | 135.68/138.13/136.32 | 40.3/42.7/37.3 | 0.4 | 0.854 | -5.048 | 0.039 |
| APOE1163/rs440446 | Intron 1 | CC/GC/GG | 76/297/250 | 0.3604 | 130.59/137.86/137.91 | 37.8/40.7/41.7 | -2.6 | 0.281 | -5.901 | 0.024 |
| APOE1575/rs769448 | Intron 1 | CC/CT/TT | 595/24/1 | 0.0210 | 136.96/137.36/142.42 | 41.3/29.6/NA | 0.7 | 0.923 | -0.722 | 0.924 |
| APOE1998/rs769449 | Intron 2 | AA/AG/GG | 6/132/480 | 0.1165 | 160.24/141.21/135.47 | 19.9/41.4/40.7 | 7.0 | 0.055 | 0.088 | 0.990 |
| APOE2440/rs769450 | Intron 2 | AA/GA/GG | 95/307/217 | 0.4015 | 144.97/136.5/133.97 | 40.1/40.9/40.9 | 4.9 | 0.038 | 5.290 | 0.037 |
| APOE2907/rs769451 | Intron 2 | GT/TT | 14/609 | 0.0112 | 137.43/136.98 | 36.6/40.9 | 0.5 | 0.967 | -5.141 | 0.639 |
| APOE3038/rs111833428 | Exon 3 | AG/GG | 2/616 | 0.0016 | 164.05/136.78 | 57.3/40.9 | 27.3 | 0.335 | 25.799 | 0.351 |
| APOE3106/rs769452 | Exon 3 | TC/TT | 1/620 | 0.0008 | 154.92/136.93 | NA/40.9 | 18.0 | 0.652 | 10.142 | 0.795 |
| APOE3937/rs429358 | Exon 4 | CC/CT/TT | 14/159/440 | 0.1525 | 153.24/142.39/134.84 | 23.3/39.4/41.2 | 8.1 | 0.010 | - |  |
| APOE4075/rs7412 | Exon 4 | CC/TC/TT | 523/94/3 | 0.0806 | 140.26/120.22/73.75 | 39.2/44.6/37.0 | -21.8 | 1.84E-07 | - |  |
| APOE4310/rs199768005 | Exon 4 | TA/TT | 5/617 | 0.0040 | 105/137.26 | 35.8/40.8 | -32.6 | 0.071 | -35.139 | 0.045 |
| APOE4528/rs374329439 | 3' UTR | CC/CT | 622/1 | 0.0008 | 137.01/124.75 | 40.8/NA | -12.3 | 0.759 | -14.201 | 0.716 |
| APOE4737/rs117656888 | 3'flanking | CC/GC | 610/10 | 0.0081 | 136.68/150.24 | 40.7/52.3 | 13.6 | 0.287 | 12.512 | 0.316 |
| APOE5361/rs1081106 | 3'flanking | CC/TC/TT | 4/98/520 | 0.0852 | 163.88/138.72/136.47 | 28.8/37.4/41.5 | 4.0 | 0.327 | 3.650 | 0.373 |
| rs439401 | Intergenic | CC/CT/TT | 255/270/84 | 0.3596 | 136.61/139.86/129.57 | 39.4/42.1/39.8 | -1.8 | 0.452 | -5.527 | 0.031 |
| APOC1rs445925 | Intergenic | AA/GA/GG | 7/121/489 | 0.1094 | 100.87/129/139.75 | 55.9/41.5/39.6 | -12.6 | 0.000 | 2.932 | 0.683 |
| APOC1p698/rs72654449 | 5'flanking | CA/CC | 5/613 | 0.0040 | 122.75/136.92 | 21.6/40.9 | -14.3 | 0.428 | -16.002 | 0.363 |
| APOC1p703/rs3207187 | 5'flanking | CC/CT | 619/1 | 0.0008 | 136.94/148.98 | 40.9/NA | 12.1 | 0.763 | 29.171 | 0.457 |
| APOC1p720 | 5'flanking | II/WI/WW | 31/224/367 | 0.2299 | 140.13/132.63/139.44 | 45.0/41.0/40.3 | -3.7 | 0.177 | -20.290 | 0.204 |
| APOC1p1170 | Intron 1 | GA/GG | 1/609 | 0.0008 | 94.93/136.57 | NA/40.7 | -41.7 | 0.296 | -49.167 | 0.207 |
| APOC1p1294 | Intron 2 | AA/AC | 618/1 | 0.0008 | 136.83/130.1 | 40.8/NA | -6.8 | 0.866 | -6.835 | 0.861 |
| APOC1p1317/rs12721048 | Intron 2 | GA/GG | 2/607 | 0.0016 | 150.98/137.55 | 38.9/40.6 | 13.5 | 0.632 | 12.056 | 0.660 |
| APOC1p1422 | Intron 2 | GA/GG | 2/621 | 0.0016 | 85.21/137.16 | 14.7/40.8 | -52.0 | 0.066 | -53.507 | 0.052 |
| APOC1p1566/rs12691088 | Intron 2 | GA/GG | 7/598 | 0.0058 | 139.78/136.62 | 31.2/41.0 | 3.2 | 0.835 | -7.001 | 0.648 |
| APOC1p2041/rs3826688 | Intron 2 | AA/GA/GG | 73/269/264 | 0.3424 | 127.94/139.99/137.23 | 39.9/42.4/39.8 | -2.4 | 0.325 | -5.796 | 0.028 |
| APOC1p2629 | Exon 3 | GA/GG | 1/618 | 0.0008 | 89.64/137.14 | NA/40.9 | -47.6 | 0.235 | -47.986 | 0.219 |
| APOC1p2817 | Intron 3 | CC/CT | 603/4 | 0.0033 | 136.39/130.83 | 40.5/22.8 | -5.6 | 0.779 | -8.417 | 0.664 |
| APOC1p3423/rs389261 | Intron 3 | GA/GG | 3/605 | 0.0025 | 118.03/136.72 | 3.2/40.8 | -18.8 | 0.416 | -20.948 | 0.354 |
| APOC1p3494 | Intron 3 | CC/CT | 619/2 | 0.0016 | 136.81/210.37 | 40.5/98.3 | 73.6 | 0.009 | 72.781 | 0.008 |
| APOC1p4334/rs12721046 | Intron 3 | AA/GA/GG | 13/160/438 | 0.1522 | 156.43/140.12/134.82 | 30.1/41.8/40.3 | 6.9 | 0.031 | 2.150 | 0.645 |
| APOC1p5641/rs1064725 | 3'UTR | GG/GT/TT | 1/46/571 | 0.0388 | 182.23/147.16/136.24 | NA/49.0/40.1 | 12.0 | 0.041 | 10.922 | 0.058 |
| APOC1p5773 | 3'flanking | GA/GG | 1/604 | 0.0008 | 125.4/136.86 | NA/40.9 | -11.5 | 0.774 | -13.867 | 0.722 |
| APOC1p5926/rs56131196 | 3'flanking | AA/GA/GG | 19/195/404 | 0.1885 | 154.08/140.17/134.32 | 29.1/42.7/40.0 | 7.2 | 0.014 | 1.441 | 0.800 |
| APOC1p6026/rs4420638 | 3'flanking | AA/GA/GG | 405/129/22 | 0.1556 | 134.61/137.65/157.81 | 40.1/42.6/35.1 | 7.0 | 0.026 | 4.378 | 0.469 |
| rs4803770 | Intergenic | CC/GC/GG | 229/281/84 | 0.3779 | 132.08/139.47/141.86 | 43.1/39.1/35.6 | 5.5 | 0.020 | 6.159 | 0.016 |
| HCR1p292/rs4803771 | HCR1 | CC/CG/GG | 583/28/1 | 0.0245 | 136.96/140.3/151.37 | 41.2/38.5/NA | 3.9 | 0.596 | 4.175 | 0.557 |
| HCR1p362 | HCR1 | CA/CC | 3/605 | 0.0025 | 122.75/136.98 | 35.9/40.9 | -14.3 | 0.536 | -16.987 | 0.450 |
| HCR1p423 | HCR1 | CC/CG/GG | 589/30/1 | 0.0258 | 137.58/131.7/119.83 | 40.9/32.7/NA | -6.3 | 0.369 | -8.568 | 0.221 |
| HCR1p575/rs157599 | HCR1 | AA/AG | 618/3 | 0.0024 | 137.14/118.01 | 40.9/3.2 | -19.3 | 0.406 | -21.379 | 0.346 |
| HCR1p727/rs149345 | HCR1 | TG/TT | 3/611 | 0.0024 | 117.41/137.05 | 3.2/40.8 | -19.8 | 0.392 | -21.621 | 0.338 |
| rs5112 | Intergenic | CC/GC/GG | 123/284/165 | 0.4633 | 128.41/138.56/138.35 | 39.4/42.5/38.1 | -4.6 | 0.052 | -6.893 | 0.004 |
| rs7259004 | Intergenic | CC/CG/GG | 476/128/8 | 0.1176 | 139.62/127.55/136.46 | 40.9/34.0/48.6 | -9.8 | 0.005 | -3.218 | 0.419 |
| HCR2p188/rs35136575 | HCR2 | CC/GC/GG | 369/203/37 | 0.2274 | 138.79/134.82/134.1 | 41.5/39.4/37.3 | -3.1 | 0.232 | -4.769 | 0.068 |
| HCR2p365 | HCR2 | CA/CC | 5/606 | 0.0041 | 139.15/136.56 | 47.3/40.7 | 2.6 | 0.884 | -7.202 | 0.685 |
| HCR2p523 | HCR2 | CC/CT | 571/27 | 0.0226 | 136.48/139.97 | 40.6/47.5 | 3.5 | 0.658 | 8.319 | 0.292 |
| APOC4p636 | 5’ flanking | CC/CT | 601/1 | 0.0008 | 137.18/191.27 | 40.9/NA | 54.3 | 0.177 | 50.132 | 0.202 |
| APOC4p968/rs76214972 | 5’ UTR | AA/AG | 576/45 | 0.0362 | 137.18/135.34 | 40.7/42.0 | -1.8 | 0.766 | -2.557 | 0.673 |
| APOC4p1150/rs148247675 | Intron 1 | AA/GA | 601/2 | 0.0017 | 136.38/210.21 | 40.5/98.3 | 73.9 | 0.009 | 72.970 | 0.008 |
| APOC4p1229 | Intron 1 | GC/GG | 2/619 | 0.0016 | 155.09/136.99 | 49.6/40.8 | 18.3 | 0.520 | 17.950 | 0.518 |
| APOC4p2557 | Intron 1 | CA/CC | 1/619 | 0.0008 | 101.86/137 | NA/40.8 | -35.3 | 0.378 | -34.748 | 0.374 |
| APOC4p2623/rs5157 | Intron 1 | CC/CT/TT | 155/315/152 | 0.4976 | 138/137.55/134.89 | 40.3/40.2/42.9 | -1.6 | 0.496 | -2.052 | 0.361 |
| APOC4p2640/rs5158 | Intron 1 | CC/CT/TT | 459/149/11 | 0.1381 | 137.39/135.98/133.59 | 40.1/43.6/35.3 | -1.5 | 0.641 | -0.767 | 0.815 |
| APOC4p2683/rs12721109 | Intron 1 | AA/AG/GG | 1/27/584 | 0.0237 | 120.81/117.3/137.95 | NA/48.3/40.4 | -19.1 | 0.009 | -3.627 | 0.640 |
| APOC4p2703/rs12721108 | Intron 1 | GG/GT | 609/10 | 0.0081 | 136.91/138.84 | 41.0/27.6 | 1.9 | 0.880 | 1.377 | 0.916 |
| APOC4p3498/rs1132899 | Exon 2 | CC/CT/TT | 160/317/143 | 0.4863 | 139.55/136.4/135.81 | 40.7/39.8/43.2 | -1.9 | 0.407 | -2.443 | 0.281 |
| APOC4p3546/rs12691089 | Exon 2 | AG/GG | 4/617 | 0.0032 | 139.21/137.04 | 25.8/40.9 | 2.2 | 0.914 | -0.807 | 0.967 |
| APOC4p3847/rs186448850 | Intron 2 | CT/TT | 2/609 | 0.0016 | 155.44/136.92 | 49.6/40.9 | 18.7 | 0.510 | 17.996 | 0.515 |
| APOC4p3927/rs5167 | Exon 3 | GG/TG/TT | 74/300/249 | 0.3596 | 141.08/138.79/133.61 | 36.0/40.9/42.0 | 4.2 | 0.082 | 4.361 | 0.067 |
| APOC4p4661/rs2288912 | C4-3'/C2-5' | CC/CG/GG | 155/316/151 | 0.4968 | 136.3/136.98/137.79 | 42.8/40.1/40.7 | 0.7 | 0.745 | 1.397 | 0.537 |
| APOC2p1591 | Intron 1 | GA/GG | 1/620 | 0.0008 | 190.9/136.96 | NA/40.8 | 54.2 | 0.176 | 49.166 | 0.209 |
| APOC2p1851/rs12709886 | Intron 1 | GA/GG | 46/572 | 0.0372 | 135.89/137.2 | 42.0/40.7 | -1.3 | 0.831 | -2.228 | 0.711 |
| APOC2p2870 | Intron 1 | GG/GT | 616/5 | 0.0040 | 136.62/189.62 | 40.4/62.6 | 53.1 | 0.003 | 49.564 | 0.005 |
| APOC2p3348/rs10420434 | Intron 1 | GA/GG | 46/574 | 0.0371 | 130.8/137.63 | 41.0/40.8 | -6.9 | 0.264 | -7.317 | 0.229 |
| APOC2p3778/rs5120 | Intron 1 | AA/AT/TT | 154/305/157 | 0.4976 | 137.73/137.42/135.27 | 40.2/39.6/43.9 | 1.2 | 0.585 | -1.460 | 0.516 |
| APOC2p4853/rs199828513 | 3'flanking | DD/WD/WW | 316/260/42 | 0.2783 | 134.46/139.37/142.11 | 41.1/41.3/36.1 | 4.4 | 0.094 | 4.794 | 0.062 |
| APOC2p5004/rs10421404 | 3'flanking | CC/CT/TT | 416/177/24 | 0.1823 | 138.15/133.69/139.56 | 40.6/42.0/34.1 | -2.3 | 0.418 | -1.781 | 0.536 |
| APOC2p5310/rs7258345 | 3'flanking | GG/TG/TT | 133/303/176 | 0.4649 | 138.69/138.01/134.9 | 41.0/38.7/43.4 | 2.0 | 0.382 | 2.711 | 0.234 |
| APOC2p5398/rs12709889 | 3'flanking | AA/GA/GG | 41/252/312 | 0.2760 | 141.78/138.36/134.24 | 36.6/40.9/41.1 | 3.9 | 0.132 | 4.514 | 0.081 |
| APOC2p5644 | 3'flanking | AG/GG | 11/585 | 0.0092 | 156.45/136.18 | 41.7/40.8 | 20.5 | 0.095 | 16.584 | 0.167 |
| MAF is the minor allele frequency; GT is genotype; GT count is the number of individuals in each genotype group; GT_SD is standard deviation of the lipid trait in each genotype group;. *Adjusted for relevant covariates, **Adjusted for *APOE*2/E*4* SNPs in addition to the covariates | | | | | | | | | | |
