# Supplementary material for: Apolipoprotein E-C1-C4-C2 gene cluster region and inter-individual variation in plasma lipoprotein levels: a comprehensive genetic association study in two ethnic groups
Source: PLoS One. 2019 Mar 26;14(3):e0214060. doi: 10.1371/journal.pone.0214060 (PMC6435132; doi:10.1371/journal.pone.0214060)
Supplement: S13 Table — MAF is the minor allele frequency; GT is genotype; GT count is the number of individuals in each genotype group; GT_SD is standard deviation of lipid traits mean in each genotype group; *Adjusted for relevant covariates, **Adjusted for APOE*2/E*4 SNPs in addition to the covariates. (DOCX) [file pone.0214060.s013.docx]

S13 Table. Single-site association analysis results for TC in NHWs

| **Variant Name/RefSNP ID** | **Location** | **Genotype** | **GT Count** | **MAF** | **Adjusted Mean of plasma TC *** | **GT_SD*** | **Beta*** | **P*** | **Adj. B.** | **Adj. P.** |
| --- | --- | --- | --- | --- | --- | --- | --- | --- | --- | --- |
| APOE560/rs449647 | 5'flanking | AA/AT/TT | 433/176/12 | 0.1610 | 218.99/212.89/202.5 | 42.5/45.1/46.4 | -6.7 | 0.043 | -1.705 | 0.626 |
| APOE832/rs405509 | 5'flanking | GG/GT/TT | 170/310/142 | 0.4775 | 216.51/218.33/214.93 | 42.7/45.9/39.1 | -0.7 | 0.776 | -6.093 | 0.017 |
| APOE1163/rs440446 | Intron 1 | CC/GC/GG | 76/297/250 | 0.3604 | 209.86/216.67/219.53 | 38.1/43.8/44.7 | -4.3 | 0.089 | -7.470 | 0.006 |
| APOE1575/rs769448 | Intron 1 | CC/CT/TT | 595/24/1 | 0.0210 | 216.84/218.67/220.93 | 43.85/37.5/NA | 1.9 | 0.816 | 0.389 | 0.961 |
| APOE1998/rs769449 | Intron 2 | AA/AG/GG | 6/132/480 | 0.1165 | 242.18/222.31/215.21 | 25.3/44.7/43.2 | 8.3 | 0.029 | 4.090 | 0.574 |
| APOE2440/rs769450 | Intron 2 | AA/GA/GG | 95/307/217 | 0.4015 | 226.44/217.25/211.92 | 42.8/44.6/41.8 | 6.9 | 0.005 | 6.917 | 0.009 |
| APOE2907/rs769451 | Intron 2 | GT/TT | 14/609 | 0.0112 | 215.12/217.03 | 41.9/43.6 | -1.9 | 0.865 | -7.093 | 0.536 |
| APOE3038/rs111833428 | Exon 3 | AG/GG | 2/616 | 0.0016 | 250.2/216.77 | 63.6/43.6 | 33.8 | 0.257 | 32.017 | 0.270 |
| APOE3106/rs769452 | Exon 3 | TC/TT | 1/620 | 0.0008 | 238.11/216.93 | NA/43.6 | 21.3 | 0.611 | 13.867 | 0.735 |
| APOE3937/rs429358 | Exon 4 | CC/CT/TT | 14/159/440 | 0.1525 | 229.3/221.27/214.71 | 26.6/42.9/43.3 | 6.8 | 0.038 | - | - |
| APOE4075/rs7412 | Exon 4 | CC/TC/TT | 523/94/3 | 0.0806 | 219.92/201.81/164.37 | 42.4/46.8/29.1 | -19.5 | 9.51E-06 | - | - |
| APOE4310/rs199768005 | Exon 4 | TA/TT | 5/617 | 0.0040 | 171.03/217.35 | 42.6/43.5 | -46.9 | 0.013 | -48.271 | 0.009 |
| APOE4528/rs374329439 | 3' UTR | CC/CT | 622/1 | 0.0008 | 216.93/252.76 | 43.6/NA | 36.0 | 0.390 | 33.710 | 0.409 |
| APOE4737/rs117656888 | 3'flanking | CC/GC | 610/10 | 0.0081 | 216.53/239.47 | 42.8/79.1 | 23.1 | 0.084 | 22.359 | 0.087 |
| APOE5361/rs1081106 | 3'flanking | CC/TC/TT | 4/98/520 | 0.0852 | 255.84/218.31/216.42 | 31.7/42.3/43.8 | 4.6 | 0.279 | 4.217 | 0.325 |
| rs439401 | Intergenic | CC/CT/TT | 255/270/84 | 0.3596 | 217.84/218.43/209.8 | 43.5/45.4/37.5 | -2.8 | 0.249 | -6.488 | 0.016 |
| APOC1rs445925 | Intergenic | AA/GA/GG | 7/121/489 | 0.1094 | 178.72/207.77/219.45 | 52.9/42.1/42.6 | -13.5 | 3.20E-04 | 0.046 | 0.995 |
| APOC1p698/rs72654449 | 5'flanking | CA/CC | 5/613 | 0.0040 | 193.51/217 | 24.7/43.7 | -23.7 | 0.209 | -25.631 | 0.164 |
| APOC1p703/rs3207187 | 5'flanking | CC/CT | 619/1 | 0.0008 | 216.89/268.82 | 43.5/NA | 52.4 | 0.212 | 71.809 | 0.081 |
| APOC1p720 | 5'flanking | II/WI/WW | 31/224/367 | 0.2299 | 216.21/213.01/219.56 | 44.3/44.1/43.1 | -4.4 | 0.120 | -32.840 | 0.049 |
| APOC1p1170 | Intron 1 | GA/GG | 1/609 | 0.0008 | 187.55/216.79 | NA/43.5 | -29.4 | 0.484 | -36.067 | 0.379 |
| APOC1p1294 | Intron 2 | AA/AC | 618/1 | 0.0008 | 216.82/230.18 | 43.5/NA | 13.4 | 0.749 | 11.468 | 0.779 |
| APOC1p1317/rs12721048 | Intron 2 | GA/GG | 2/607 | 0.0016 | 251.51/217.24 | 11.3/43.3 | 34.5 | 0.244 | 32.181 | 0.266 |
| APOC1p1422 | Intron 2 | GA/GG | 2/621 | 0.0016 | 164.28/217.16 | 31.1/43.5 | -53.3 | 0.072 | -54.868 | 0.058 |
| APOC1p1566/rs12691088 | Intron 2 | GA/GG | 7/598 | 0.0058 | 214.02/216.77 | 36.5/43.7 | -2.8 | 0.862 | -10.774 | 0.501 |
| APOC1p2041/rs3826688 | Intron 2 | AA/GA/GG | 73/269/264 | 0.3424 | 208.35/218.41/218.56 | 37.2/45.6/43.6 | -3.6 | 0.154 | -6.812 | 0.013 |
| APOC1p2629 | Exon 3 | GA/GG | 1/618 | 0.0008 | 192.39/217.2 | NA/43.6 | -24.9 | 0.552 | -26.552 | 0.515 |
| APOC1p2817 | Intron 3 | CC/CT | 603/4 | 0.0033 | 216.34/224.48 | 43.1/28.0 | 8.2 | 0.694 | 5.348 | 0.792 |
| APOC1p3423/rs389261 | Intron 3 | GA/GG | 3/605 | 0.0025 | 185.1/216.81 | 21.2/43.4 | -32.0 | 0.186 | -32.953 | 0.163 |
| APOC1p3494 | Intron 3 | CC/CT | 619/2 | 0.0016 | 216.76/320.11 | 42.8/153.4 | 103.8 | 4.40E-04 | 103.096 | 3.37E-04 |
| APOC1p4334/rs12721046 | Intron 3 | AA/GA/GG | 13/160/438 | 0.1522 | 233.5/219.8/215.22 | 33.9/44.1/43.4 | 5.9 | 0.077 | 3.266 | 0.506 |
| APOC1p5641/rs1064725 | 3'UTR | GG/GT/TT | 1/46/571 | 0.0388 | 259.34/228.98/216.15 | NA/45.3/43.4 | 13.6 | 0.026 | 12.813 | 0.033 |
| APOC1p5773 | 3'flanking | GA/GG | 1/604 | 0.0008 | 254.09/216.74 | NA/43.8 | 37.6 | 0.370 | 34.771 | 0.394 |
| APOC1p5926/rs56131196 | 3'flanking | AA/GA/GG | 19/195/404 | 0.1885 | 229.72/220.53/214.39 | 31.3/44.8/43.1 | 6.7 | 0.030 | 5.863 | 0.325 |
| APOC1p6026/rs4420638 | 3'flanking | AA/GA/GG | 405/129/22 | 0.1556 | 214.64/218.18/235.77 | 43.2/44.6/38.8 | 6.8 | 0.040 | 10.720 | 0.093 |
| rs4803770 | Intergenic | CC/GC/GG | 229/281/84 | 0.3779 | 210.83/219.75/221.05 | 41.2/43.7/43.5 | 6.1 | 0.014 | 6.434 | 0.016 |
| HCR1p292/rs4803771 | HCR1 | CC/CG/GG | 583/28/1 | 0.0245 | 217.05/216.36/248.27 | 44.1/37.6/NA | 1.4 | 0.854 | 2.123 | 0.775 |
| HCR1p362 | HCR1 | CA/CC | 3/605 | 0.0025 | 213.1/216.88 | 19.7/43.7 | -3.8 | 0.875 | -6.109 | 0.795 |
| HCR1p423 | HCR1 | CC/CG/GG | 589/30/1 | 0.0258 | 217.22/209.32/203.05 | 43.1/40.8/NA | -7.8 | 0.281 | -10.024 | 0.171 |
| HCR1p575/rs157599 | HCR1 | AA/AG | 618/3 | 0.0024 | 217.25/185.31 | 436/21.2 | -32.2 | 0.184 | -33.287 | 0.160 |
| HCR1p727/rs149345 | HCR1 | TG/TT | 3/611 | 0.0024 | 184.31/217.22 | 21.2/43.4 | -33.2 | 0.169 | -34.001 | 0.149 |
| rs5112 | *APOC1P1* | CC/GC/GG | 123/284/165 | 0.4633 | 208.18/218.08/218.71 | 38.1/44.7/44.1 | -5.0 | 0.045 | -7.129 | 0.004 |
| rs7259004 | *APOC1P1* | CC/CG/GG | 476/128/8 | 0.1176 | 218.82/207.45/218.26 | 43.5/36.3/54.2 | -9.1 | 0.013 | -1.574 | 0.707 |
| HCR2p188/rs35136575 | HCR2 | CC/GC/GG | 369/203/37 | 0.2274 | 217.48/215.6/212.29 | 44.9/39.3/44.9 | -2.3 | 0.414 | -3.814 | 0.163 |
| HCR2p365 | HCR2 | CA/CC | 5/606 | 0.0041 | 210.22/216.65 | 51.3/43.3 | -6.5 | 0.730 | -14.738 | 0.427 |
| HCR2p523 | HCR2 | CC/CT | 571/27 | 0.0226 | 216.31/224.33 | 43.4/44.7 | 8.1 | 0.327 | 13.449 | 0.103 |
| APOC4p636 | 5’ flanking | CC/CT | 601/1 | 0.0008 | 217.16/280.36 | 43.5/NA | 63.6 | 0.130 | 60.514 | 0.140 |
| APOC4p968/rs76214972 | 5’ UTR | AA/AG | 576/45 | 0.0362 | 217.5/211.93 | 43.7/42.1 | -5.6 | 0.388 | -5.673 | 0.370 |
| APOC4p1150/rs148247675 | Intron 1 | AA/GA | 601/2 | 0.0017 | 216.26/319.6 | 42.6/153.4 | 103.8 | 4.30E-04 | 103.184 | 3.28E-04 |
| APOC4p1229 | Intron 1 | GC/GG | 2/619 | 0.0016 | 233.67/217.04 | 60.1/43.5 | 17.0 | 0.570 | 16.188 | 0.579 |
| APOC4p2557 | Intron 1 | CA/CC | 1/619 | 0.0008 | 160.39/217.07 | NA/43.5 | -57.4 | 0.173 | -57.359 | 0.162 |
| APOC4p2623/rs5157 | Intron 1 | CC/CT/TT | 155/315/152 | 0.4976 | 216.43/217.92/215.73 | 42.9/43.6/44.4 | -0.3 | 0.885 | -0.889 | 0.705 |
| APOC4p2640/rs5158 | Intron 1 | CC/CT/TT | 459/149/11 | 0.1381 | 217.61/215.36/216.86 | 42.0/49.0/33.7 | -1.8 | 0.608 | -2.515 | 0.465 |
| APOC4p2683/rs12721109 | Intron 1 | AA/AG/GG | 1/27/584 | 0.0237 | 204.3/204.19/217.79 | NA/59.9/42.7 | -12.8 | 0.098 | -7.586 | 0.349 |
| APOC4p2703/rs12721108 | Intron 1 | GG/GT | 609/10 | 0.0081 | 217.09/207.6 | 43.8/33.2 | -9.5 | 0.476 | -10.232 | 0.456 |
| APOC4p3498/rs1132899 | Exon 2 | CC/CT/TT | 160/317/143 | 0.4863 | 218.31/216.28/217.16 | 43.3/43.4/44.3 | -0.6 | 0.797 | -1.328 | 0.575 |
| APOC4p3546/rs12691089 | Exon 2 | AG/GG | 4/617 | 0.0032 | 211.98/217.12 | 26.7/43.6 | -5.2 | 0.806 | -7.878 | 0.701 |
| APOC4p3847/rs186448850 | Intron 2 | CT/TT | 2/609 | 0.0016 | 234/216.86 | 60.1/43.7 | 17.5 | 0.558 | 16.131 | 0.579 |
| APOC4p3927/rs5167 | Exon 3 | GG/TG/TT | 74/300/249 | 0.3596 | 220.48/217.95/214.8 | 40.4/42.5/45.8 | 2.9 | 0.244 | 4.243 | 0.088 |
| APOC4p4661/rs2288912 | C4-3'/C2-5' | CC/CG/GG | 155/316/151 | 0.4968 | 217.56/217.08/216.26 | 44.4/43.7/42.6 | -0.7 | 0.786 | 0.207 | 0.930 |
| APOC2p1591 | Intron 1 | GA/GG | 1/620 | 0.0008 | 279.62/216.99 | NA/43.5 | 63.0 | 0.133 | 59.056 | 0.149 |
| APOC2p1851/rs12709886 | Intron 1 | GA/GG | 46/572 | 0.0372 | 213.01/217.48 | 42.5/43.7 | -4.5 | 0.485 | -4.700 | 0.455 |
| APOC2p2870 | Intron 1 | GG/GT | 616/5 | 0.0040 | 216.62/274.94 | 42.7/96.4 | 58.4 | 0.002 | 55.173 | 0.002 |
| APOC2p3348/rs10420434 | Intron 1 | GA/GG | 46/574 | 0.0371 | 214.96/217.38 | 37.0/44.1 | -2.4 | 0.704 | -2.680 | 0.673 |
| APOC2p3778/rs5120 | Intron 1 | AA/AT/TT | 154/305/157 | 0.4976 | 215.87/216.86/218.48 | 42.5/42.7/46.2 | -1.3 | 0.578 | -0.124 | 0.958 |
| APOC2p4853/rs199828513 | 3'flanking | DD/WD/WW | 316/260/42 | 0.2783 | 215.57/218.43/219.64 | 44.6/43.0/40.1 | 2.4 | 0.371 | 3.880 | 0.149 |
| APOC2p5004/rs10421404 | 3'flanking | CC/CT/TT | 416/177/24 | 0.1823 | 218.5/212.98/219.46 | 41.9/47.7/37.0 | -3.1 | 0.309 | -3.472 | 0.250 |
| APOC2p5310/rs7258345 | 3'flanking | GG/TG/TT | 133/303/176 | 0.4649 | 217.14/216.27/217.27 | 43.5/42.7/43.7 | -0.1 | 0.956 | 0.688 | 0.773 |
| APOC2p5398/rs12709889 | 3'flanking | AA/GA/GG | 41/252/312 | 0.2760 | 219.86/217.27/215.5 | 40.6/42.5/44.4 | 2.0 | 0.470 | 3.494 | 0.197 |
| APOC2p5644 | 3'flanking | AG/GG | 11/585 | 0.0092 | 230.97/216.28 | 41.9/43.5 | 14.9 | 0.245 | 11.864 | 0.343 |

MAF is the minor allele frequency; GT is genotype; GT count is the number of individuals in each genotype group; GT_SD is standard deviation of lipid traits mean in each genotype group; *Adjusted for relevant covariates, **Adjusted for *APOE*2/E*4* SNPs in addition to the covariates
