# Supplementary material for: Apolipoprotein E-C1-C4-C2 gene cluster region and inter-individual variation in plasma lipoprotein levels: a comprehensive genetic association study in two ethnic groups
Source: PLoS One. 2019 Mar 26;14(3):e0214060. doi: 10.1371/journal.pone.0214060 (PMC6435132; doi:10.1371/journal.pone.0214060)
Supplement: S14 Table — MAF is the minor allele frequency; GT is genotype; GT count is the number of individuals in each genotype group; GT_SD is standard deviation of lipid traits mean in each genotype group; *Adjusted for relevant covariates, **Adjusted for APOE*2/E*4 SNPs in addition to the covariates. Four rare variants were excluded due to missing data. (DOCX) [file pone.0214060.s014.docx]

S14 Table. Single-site association analysis results for ApoB in NHWs

| **Variant Name/RefSNP ID** | **Location** | **Genotype** | **GT Count** | **MAF** | **Adjusted Mean of plasma of apoB*** | **GT_SD*** | **Beta*** | **P*** | **Adj. B.** | **Adj. P.** |
| --- | --- | --- | --- | --- | --- | --- | --- | --- | --- | --- |
| APOE560/rs449647 | 5'flanking | AA/AT/TT | 306/119/7 | 0.1610 | 88.92/84.42/90.53 | 23.4/24.8/13.2 | -1.0 | 0.12896 | 0.524 | 0.409 |
| APOE832/rs405509 | 5'flanking | GG/GT/TT | 115/217/102 | 0.4775 | 81.67/89.51/91.58 | 26.1/22.6/22.9 | 1.5 | 0.00090 | -0.051 | 0.914 |
| APOE1163/rs440446 | Intron 1 | CC/GC/GG | 51/206/177 | 0.3604 | 88.2/89.54/85.95 | 23.0/23.5/24.8 | 0.6 | 0.23194 | -0.231 | 0.642 |
| APOE1575/rs769448 | Intron 1 | CC/CT/TT | 413/17/1 | 0.0210 | 87.88/90.73/93.77 | 24.3/17.4/NA | 0.9 | 0.52039 | 0.407 | 0.767 |
| APOE1998/rs769449 | Intron 2 | AA/AG/GG | 4/103/324 | 0.1165 | 93.32/93.83/85.99 | 23.2/22.4/24.1 | 2.0 | 0.00297 | -0.190 | 0.886 |
| APOE2440/rs769450 | Intron 2 | AA/GA/GG | 63/217/153 | 0.4015 | 89.59/87.42/87.95 | 24.1/23.9/24.1 | 0.2 | 0.74223 | 0.196 | 0.685 |
| APOE2907/rs769451 | Intron 2 | GT/TT | 9/425 | 0.0112 | 87.57/87.92 | 32.9/23.8 | -0.2 | 0.91960 | -0.608 | 0.781 |
| APOE3038/rs111833428 | Exon 3 | AG/GG | 1/431 | 0.0016 | 140.55/87.71 | NA/23.8 | 13.8 | 0.03418 | 13.502 | 0.027 |
| APOE3106/rs769452 | Exon 3 | TC/TT | 1/432 | 0.0008 | 121.87/87.93 | NA/23.9 | 9.1 | 0.16020 | 6.925 | 0.258 |
| APOE3937/rs429358 | Exon 4 | CC/CT/TT | 9/121/296 | 0.1525 | 90.86/94.72/85.3 | 17.2/22.9/24.1 | 2.1 | 0.00050 | - | - |
| APOE4075/rs7412 | Exon 4 | CC/TC/TT | 365/65/3 | 0.0806 | 90.92/73.55/32.32 | 23.0/21.1/17.1 | -5.6 | 9.65E-13 | - | - |
| APOE4310/rs199768005 | Exon 4 | TA/TT | 2/431 | 0.0040 | 92.47/87.91 | 45.3/23.9 | 1.0 | 0.82199 | -0.364 | 0.933 |
| APOE4737/rs117656888 | 3'flanking | CC/GC | 426/7 | 0.0081 | 87.82/93.42 | 24.0/22.9 | 1.6 | 0.52030 | 1.369 | 0.559 |
| APOE5361/rs1081106 | 3'flanking | CC/TC/TT | 3/59/372 | 0.0852 | 107.81/86.35/88.01 | 28.0/23.3/24.0 | 0.1 | 0.88128 | 0.406 | 0.614 |
| rs439401 | Intergenic | CC/CT/TT | 179/185/59 | 0.3596 | 85.66/91.09/85.24 | 24.8/23.5/22.6 | 0.4 | 0.39749 | -0.433 | 0.374 |
| APOC1rs445925 | Intergenic | AA/GA/GG | 5/86/341 | 0.1094 | 50.38/79.28/90.44 | 27.9/23.8/22.9 | -3.8 | 5.17E-08 | -0.287 | 0.829 |
| APOC1p698/rs72654449 | 5'flanking | CA/CC | 2/429 | 0.0040 | 77.26/88 | 16.2/24.0 | -2.8 | 0.55052 | -2.977 | 0.493 |
| APOC1p703/rs3207187 | 5'flanking | CC/CT | 431/1 | 0.0008 | 87.93/83.81 | 24.0/NA | -0.9 | 0.89283 | 3.644 | 0.555 |
| APOC1p720 | 5'flanking | II/WI/WW | 23/163/247 | 0.2299 | 82.72/86.9/89.18 | 27.6/24.6/23.1 | -0.8 | 0.11683 | -1.283 | 0.675 |
| APOC1p1170 | Intron 1 | GA/GG | 1/424 | 0.0008 | 113.69/87.76 | NA/24.1 | 7.1 | 0.27763 | 4.801 | 0.435 |
| APOC1p1294 | Intron 2 | AA/AC | 432/1 | 0.0008 | 87.88/98.11 | 24.0/NA | 3.1 | 0.63865 | 2.886 | 0.637 |
| APOC1p1317/rs12721048 | Intron 2 | GA/GG | 2/421 | 0.0016 | 130.48/87.94 | 17.4/23.9 | 11.2 | 0.01598 | 10.985 | 0.012 |
| APOC1p1422 | Intron 2 | GA/GG | 1/433 | 0.0016 | 93.79/87.9 | NA/24.0 | 1.9 | 0.77425 | 1.537 | 0.802 |
| APOC1p1566/rs12691088 | Intron 2 | GA/GG | 4/415 | 0.0058 | 69.72/88.14 | 9.3/23.9 | -5.0 | 0.12948 | -7.740 | 0.012 |
| APOC1p2041/rs3826688 | Intron 2 | AA/GA/GG | 55/183/184 | 0.3424 | 83.5/91.49/85.59 | 21.0/23.6/24.8 | 0.3 | 0.51703 | -0.485 | 0.322 |
| APOC1p2629 | Exon 3 | GA/GG | 1/429 | 0.0008 | 69.33/88.11 | NA/24.0 | -5.1 | 0.43402 | -5.381 | 0.379 |
| APOC1p2817 | Intron 3 | CC/CT | 419/3 | 0.0033 | 87.84/71.24 | 23.85/6.9 | -4.5 | 0.23676 | -5.437 | 0.122 |
| APOC1p3423/rs389261 | Intron 3 | GA/GG | 3/421 | 0.0025 | 74.76/87.94 | 10.7/23.8 | -3.5 | 0.35153 | -3.767 | 0.284 |
| APOC1p3494 | Intron 3 | CC/CT | 432/1 | 0.0016 | 87.88/107.03 | 24.0/NA | 5.4 | 0.41256 | 5.272 | 0.391 |
| APOC1p4334/rs12721046 | Intron 3 | AA/GA/GG | 9/123/298 | 0.1522 | 102.6/90.83/86.13 | 25.1/22.7/24.2 | 1.6 | 0.00983 | 0.242 | 0.778 |
| APOC1p5641/rs1064725 | 3'UTR | GG/GT/TT | 1/28/401 | 0.0388 | 53.28/98.46/87.22 | NA/25.9/23.6 | 2.0 | 0.09267 | 1.452 | 0.194 |
| APOC1p5926/rs56131196 | 3'flanking | AA/GA/GG | 13/146/272 | 0.1885 | 97.38/91.68/85.37 | 22.9/23.0/24.2 | 1.7 | 0.00229 | 0.420 | 0.685 |
| APOC1p6026/rs4420638 | 3'flanking | AA/GA/GG | 271/99/16 | 0.1556 | 85.38/89.53/97.75 | 24.3/23.2/21.7 | 1.4 | 0.01775 | 0.408 | 0.712 |
| rs4803770 | Intergenic | CC/GC/GG | 171/187/57 | 0.3779 | 85.8/89.24/88.61 | 23.5/24.6/20.5 | 0.6 | 0.21851 | 0.603 | 0.200 |
| HCR1p292/rs4803771 | HCR1 | CC/CG/GG | 408/17/1 | 0.0245 | 87.78/89.74/101.01 | 23.8/30.3/NA | 0.7 | 0.64404 | 0.943 | 0.492 |
| HCR1p362 | HCR1 | CA/CC | 1/421 | 0.0025 | 94.53/87.84 | NA/24.1 | 2.1 | 0.75319 | 1.636 | 0.791 |
| HCR1p423 | HCR1 | CC/CG/GG | 415/17/1 | 0.0258 | 87.52/98.35/78.55 | 23.7/30.2/NA | 2.1 | 0.14804 | 1.456 | 0.288 |
| HCR1p575/rs157599 | HCR1 | AA/AG | 430/3 | 0.0024 | 88.02/74.88 | 24.0/10.7 | -3.5 | 0.35815 | -3.822 | 0.282 |
| HCR1p727/rs149345 | HCR1 | TG/TT | 3/426 | 0.0024 | 74.72/87.81 | 10.7/23.9 | -3.5 | 0.35832 | -3.798 | 0.282 |
| rs5112 | *APOC1P1* | CC/GC/GG | 87/206/112 | 0.4633 | 86.97/88.52/88.35 | 21.1/25.6/23.3 | -0.1 | 0.75492 | -0.699 | 0.118 |
| rs7259004 | *APOC1P1* | CC/CG/GG | 327/93/6 | 0.1176 | 89.05/82.56/94.13 | 23.3/24.3/35.9 | -1.3 | 0.05153 | 0.889 | 0.236 |
| HCR2p188/rs35136575 | HCR2 | CC/GC/GG | 255/144/25 | 0.2274 | 88.13/88.97/79.55 | 23.5/25.5/20.6 | -0.5 | 0.36635 | -0.878 | 0.078 |
| HCR2p365 | HCR2 | CA/CC | 3/423 | 0.0041 | 99.1/87.77 | 32.0/23.9 | 3.0 | 0.41812 | 1.133 | 0.752 |
| HCR2p523 | HCR2 | CC/CT | 395/21 | 0.0226 | 87.49/91.33 | 23.8/26.3 | 1.0 | 0.47773 | 3.013 | 0.033 |
| APOC4p968/rs76214972 | 5’ UTR | AA/AG | 400/33 | 0.0362 | 87.57/92.24 | 23.8/26.3 | 1.2 | 0.29092 | 0.610 | 0.583 |
| APOC4p1150/rs148247675 | Intron 1 | AA/GA | 418/1 | 0.0017 | 87.74/107.42 | 23.9/NA | 5.5 | 0.39750 | 5.435 | 0.373 |
| APOC4p2557 | Intron 1 | CA/CC | 1/432 | 0.0008 | 68.6/87.95 | NA/24.0 | -5.3 | 0.42411 | -5.365 | 0.382 |
| APOC4p2623/rs5157 | Intron 1 | CC/CT/TT | 112/214/108 | 0.4976 | 89.47/87.43/87.27 | 23.5/24.9/22.5 | -0.3 | 0.49795 | -0.422 | 0.309 |
| APOC4p2640/rs5158 | Intron 1 | CC/CT/TT | 323/101/7 | 0.1381 | 87.34/90.18/81.09 | 23.6/25.2/24.6 | 0.4 | 0.59389 | 0.236 | 0.709 |
| APOC4p2683/rs12721109 | Intron 1 | AA/AG/GG | 1/17/406 | 0.0237 | 53.83/71.26/88.65 | NA/23.5/23.8 | -5.0 | 0.00057 | -1.863 | 0.204 |
| APOC4p2703/rs12721108 | Intron 1 | GG/GT | 423/8 | 0.0081 | 88.02/83.12 | 24.0/16.7 | -1.2 | 0.59732 | -0.163 | 0.944 |
| APOC4p3498/rs1132899 | Exon 2 | CC/CT/TT | 115/216/101 | 0.4863 | 89.22/87.06/88.46 | 23.1/25.1/22.5 | -0.1 | 0.79089 | -0.209 | 0.620 |
| APOC4p3546/rs12691089 | Exon 2 | AG/GG | 2/431 | 0.0032 | 80.57/87.96 | 0.6/24.0 | -1.8 | 0.69724 | -2.033 | 0.639 |
| APOC4p3927/rs5167 | Exon 3 | GG/TG/TT | 51/216/167 | 0.3596 | 88.12/88.12/87.59 | 19.2/25.5/23.3 | 0.1 | 0.81626 | 0.262 | 0.564 |
| APOC4p4661/rs2288912 | C4-3'/C2-5' | CC/CG/GG | 112/213/109 | 0.4968 | 87.57/87.5/89.09 | 22.2/24.9/23.9 | 0.2 | 0.66177 | 0.351 | 0.400 |
| APOC2p1851/rs12709886 | Intron 1 | GA/GG | 36/394 | 0.0372 | 92.79/87.56 | 28.7/23.5 | 1.4 | 0.23570 | 0.625 | 0.560 |
| APOC2p2870 | Intron 1 | GG/GT | 429/4 | 0.0040 | 87.71/110.92 | 23.9/16.9 | 6.3 | 0.05291 | 5.077 | 0.098 |
| APOC2p3348/rs10420434 | Intron 1 | GA/GG | 31/400 | 0.0371 | 84/88.23 | 27.1/23.8 | -1.2 | 0.31010 | -0.642 | 0.582 |
| APOC2p3778/rs5120 | Intron 1 | AA/AT/TT | 110/205/112 | 0.4976 | 88.77/87.16/87.61 | 24.2/24.7/22.2 | 0.1 | 0.74796 | 0.349 | 0.400 |
| APOC2p4853/rs199828513 | 3'flanking | DD/WD/WW | 221/181/29 | 0.2783 | 87.77/87.53/91.29 | 23.6/25.2/19.6 | 0.2 | 0.67570 | 0.465 | 0.338 |
| APOC2p5004/rs10421404 | 3'flanking | CC/CT/TT | 289/125/17 | 0.1823 | 87.5/87.88/91.32 | 23.3/24.6/26.0 | 0.3 | 0.63942 | 0.153 | 0.775 |
| APOC2p5310/rs7258345 | 3'flanking | GG/TG/TT | 95/212/123 | 0.4649 | 89.54/87.89/86.85 | 22.7/25.4/22.5 | 0.4 | 0.41406 | 0.511 | 0.229 |
| APOC2p5398/rs12709889 | 3'flanking | AA/GA/GG | 28/174/219 | 0.2760 | 90.49/87.02/87.9 | 19.5/25.1/23.2 | 0.1 | 0.91243 | 0.341 | 0.485 |
| APOC2p5644 | 3'flanking | AG/GG | 9/405 | 0.0092 | 94.14/87.4 | 30.6/23.7 | 1.7 | 0.42698 | 1.222 | 0.552 |
| MAF is the minor allele frequency; GT is genotype; GT count is the number of individuals in each genotype group; GT_SD is standard deviation of lipid traits mean in each genotype group; *Adjusted for relevant covariates, **Adjusted for *APOE*2/E*4* SNPs in addition to the covariates. Four rare variants were excluded due to missing data. | | | | | | | | | | |
