# Supplementary material for: Apolipoprotein E-C1-C4-C2 gene cluster region and inter-individual variation in plasma lipoprotein levels: a comprehensive genetic association study in two ethnic groups
Source: PLoS One. 2019 Mar 26;14(3):e0214060. doi: 10.1371/journal.pone.0214060 (PMC6435132; doi:10.1371/journal.pone.0214060)
Supplement: S15 Table — MAF is the minor allele frequency; GT is genotype; GT count is the number of individuals in each genotype group; GT_SD is standard deviation of lipid traits mean in each genotype group; *Adjusted for relevant covariates, **Adjusted for APOE*2/E*4 SNPs in addition to the covariates. APOC1p703/rs3207187 was excluded due to missing data. (DOCX) [file pone.0214060.s015.docx]

S15 Table. Single-site association analysis results for HDL-C in NHWs

| **Variant Name/RefSNP ID** | **Location** | **Genotype** | **GT Count** | **MAF** | **Adjusted Mean of plasma of HDL-C*** | **GT_SD*** | **Beta*** | **P*** | **Adj. B.** | **Adj. P** |
| --- | --- | --- | --- | --- | --- | --- | --- | --- | --- | --- |
| APOE560/rs449647 | 5'flanking | AA/AT/TT | 431/176/12 | 0.1610 | 50.95/49.6/50.65 | 14.4/13.4/11.0 | -0.014 | 0.35257 | -0.010 | 0.528 |
| APOE832/rs405509 | 5'flanking | GG/GT/TT | 168/310/142 | 0.4775 | 50.07/50.56/51.17 | 13.9/14.0/14.2 | 0.009 | 0.40306 | 0.012 | 0.292 |
| APOE1163/rs440446 | Intron 1 | CC/GC/GG | 76/297/248 | 0.3604 | 51.8/50.73/50.04 | 13.8/14.3/13.7 | 0.012 | 0.28650 | 0.007 | 0.598 |
| APOE1575/rs769448 | Intron 1 | CC/CT/TT | 593/24/1 | 0.0210 | 50.27/55.4/70.05 | 13.8/17.1/NA | 0.084 | 0.01974 | 0.082 | 0.025 |
| APOE1998/rs769449 | Intron 2 | AA/AG/GG | 6/132/478 | 0.1165 | 48.13/50.11/50.79 | 9.6/14.0/14.1 | -0.009 | 0.58003 | 0.036 | 0.282 |
| APOE2440/rs769450 | Intron 2 | AA/GA/GG | 94/306/217 | 0.4015 | 50.42/50.88/50.12 | 15.4/13.5/14.3 | 0.003 | 0.77245 | -0.005 | 0.688 |
| APOE2907/rs769451 | Intron 2 | GT/TT | 14/607 | 0.0112 | 52.84/50.53 | 11.4/14.1 | 0.048 | 0.33896 | 0.053 | 0.313 |
| APOE3038/rs111833428 | Exon 3 | AG/GG | 2/614 | 0.0016 | 55.59/50.51 | 4.2/14.1 | 0.103 | 0.43657 | 0.094 | 0.484 |
| APOE3106/rs769452 | Exon 3 | TC/TT | 1/618 | 0.0008 | 61.96/50.57 | NA/14.1 | 0.203 | 0.27872 | 0.214 | 0.257 |
| APOE3937/rs429358 | Exon 4 | CC/CT/TT | 14/159/438 | 0.1525 | 47.93/49.89/51.01 | 12.7/14.1/14.1 | -0.018 | 0.22334 | - | - |
| APOE4075/rs7412 | Exon 4 | CC/TC/TT | 522/93/3 | 0.0806 | 50.76/49.16/54.02 | 14.3/12.6/16.1 | -0.015 | 0.45241 | - | - |
| APOE4310/rs199768005 | Exon 4 | TA/TT | 5/615 | 0.0040 | 50.32/50.54 | 10.5/14.0 | 0.011 | 0.89267 | 0.005 | 0.952 |
| APOE4528/rs374329439 | 3' UTR | CC/CT | 620/1 | 0.0008 | 50.56/64.69 | 14.1/NA | 0.244 | 0.19264 | 0.234 | 0.213 |
| APOE4737/rs117656888 | 3'flanking | CC/GC | 608/10 | 0.0081 | 50.4/58.73 | 13.9/20.1 | 0.118 | 0.04872 | 0.109 | 0.072 |
| APOE5361/rs1081106 | 3'flanking | CC/TC/TT | 4/97/519 | 0.0852 | 54.27/52.26/50.19 | 17.2/14.3/13.9 | 0.032 | 0.09145 | 0.028 | 0.157 |
| rs439401 | Intergenic | CC/CT/TT | 253/270/84 | 0.3596 | 50.14/50.35/51.41 | 13.6/14.6/13.6 | 0.007 | 0.54485 | 0.001 | 0.929 |
| APOC1rs445925 | Intergenic | AA/GA/GG | 7/120/488 | 0.1094 | 46.15/49.94/50.91 | 15.2/13.2/14.2 | -0.019 | 0.27231 | -0.015 | 0.664 |
| APOC1p698/rs72654449 | 5'flanking | CA/CC | 5/611 | 0.0040 | 48.45/50.65 | 12.8/14.1 | -0.023 | 0.78922 | -0.032 | 0.703 |
| APOC1p720 | 5'flanking | II/WI/WW | 31/223/366 | 0.2299 | 47.92/49.79/51.31 | 12.8/13.4/14.5 | -0.022 | 0.08599 | -0.103 | 0.179 |
| APOC1p1170 | Intron 1 | GA/GG | 1/607 | 0.0008 | 77.93/50.64 | NA/14.1 | 0.399 | 0.03328 | 0.409 | 0.030 |
| APOC1p1294 | Intron 2 | AA/AC | 616/1 | 0.0008 | 50.57/71.84 | 14.1/NA | 0.268 | 0.15333 | 0.259 | 0.169 |
| APOC1p1317/rs12721048 | Intron 2 | GA/GG | 2/605 | 0.0016 | 74.31/50.58 | 11.3/14.0 | 0.319 | 0.01616 | 0.311 | 0.020 |
| APOC1p1422 | Intron 2 | GA/GG | 2/619 | 0.0016 | 61.07/50.55 | 7.1/14.0 | 0.177 | 0.18304 | 0.167 | 0.211 |
| APOC1p1566/rs12691088 | Intron 2 | GA/GG | 7/596 | 0.0058 | 48.07/50.63 | 21.0/14.0 | -0.073 | 0.30637 | -0.060 | 0.417 |
| APOC1p2041/rs3826688 | Intron 2 | AA/GA/GG | 73/269/262 | 0.3424 | 52.15/50.6/50.26 | 14.5/14.4/13.7 | 0.010 | 0.39851 | 0.004 | 0.776 |
| APOC1p2629 | Exon 3 | GA/GG | 1/616 | 0.0008 | 74.09/50.6 | NA/14.0 | 0.297 | 0.11169 | 0.288 | 0.124 |
| APOC1p2817 | Intron 3 | CC/CT | 602/3 | 0.0033 | 50.49/57.95 | 14.0/13.0 | 0.132 | 0.22460 | 0.130 | 0.233 |
| APOC1p3423/rs389261 | Intron 3 | GA/GG | 3/603 | 0.0025 | 41.42/50.59 | 5/14.0 | -0.180 | 0.09939 | -0.191 | 0.081 |
| APOC1p3494 | Intron 3 | CC/CT | 617/2 | 0.0016 | 50.54/72.29 | 13.9/38.2 | 0.262 | 0.04784 | 0.254 | 0.056 |
| APOC1p4334/rs12721046 | Intron 3 | AA/GA/GG | 13/160/436 | 0.1522 | 45.6/51.2/50.56 | 8.1/14.5/14.1 | -6.21E-05 | 0.99672 | 0.027 | 0.234 |
| APOC1p5641/rs1064725 | 3'UTR | GG/GT/TT | 1/46/569 | 0.0388 | 53.95/51.58/50.49 | NA/12.4/14.2 | 0.027 | 0.32535 | 0.024 | 0.384 |
| APOC1p5773 | 3'flanking | GA/GG | 1/602 | 0.0008 | 64.58/50.52 | NA/14.1 | 0.243 | 0.19556 | 0.234 | 0.214 |
| APOC1p5926/rs56131196 | 3'flanking | AA/GA/GG | 19/194/403 | 0.1885 | 47.37/50.67/50.75 | 11.2/14.1/14.2 | -0.007 | 0.61460 | 0.025 | 0.362 |
| APOC1p6026/rs4420638 | 3'flanking | AA/GA/GG | 404/128/22 | 0.1556 | 50.73/51.06/47.64 | 14.1/15.4/11.2 | -0.009 | 0.56998 | 0.024 | 0.435 |
| rs4803770 | Intergenic | CC/GC/GG | 229/280/83 | 0.3779 | 49.8/51.14/49.72 | 13.6/13.8/14.6 | 0.003 | 0.76528 | -0.003 | 0.828 |
| HCR1p292/rs4803771 | HCR1 | CC/CG/GG | 581/28/1 | 0.0245 | 50.51/50.04/33.57 | 14.1/13.4/NA | -0.021 | 0.54022 | -0.023 | 0.491 |
| HCR1p362 | HCR1 | CA/CC | 3/603 | 0.0025 | 54.67/50.49 | 32.5/14.0 | 0.001 | 0.99148 | -0.007 | 0.950 |
| HCR1p423 | HCR1 | CC/CG/GG | 587/30/1 | 0.0258 | 50.48/53.78/54.15 | 14.1/13.5/NA | 0.055 | 0.09096 | 0.051 | 0.128 |
| HCR1p575/rs157599 | HCR1 | AA/AG | 616/3 | 0.0024 | 50.65/41.6 | 14.0/5 | -0.178 | 0.09959 | -0.190 | 0.082 |
| HCR1p727/rs149345 | HCR1 | TG/TT | 3/609 | 0.0024 | 41.69/50.72 | 5/14.1 | -0.178 | 0.10151 | -0.189 | 0.083 |
| rs5112 | *APOC1P1* | CC/GC/GG | 123/283/164 | 0.4633 | 50.33/50.31/50.79 | 14.1/14.2/14.3 | -0.005 | 0.65328 | -0.008 | 0.490 |
| rs7259004 | *APOC1P1* | CC/CG/GG | 475/127/8 | 0.1176 | 50.51/50.11/56.11 | 14.2/13.9/12.9 | 0.006 | 0.71147 | 0.020 | 0.300 |
| HCR2p188/rs35136575 | HCR2 | CC/GC/GG | 368/202/37 | 0.2274 | 49.76/51.64/51.86 | 14.2/13.9/12.8 | 0.027 | 0.03304 | 0.027 | 0.034 |
| HCR2p365 | HCR2 | CA/CC | 5/604 | 0.0041 | 49.01/50.56 | 12.6/14.0 | -0.019 | 0.81824 | -0.003 | 0.975 |
| HCR2p523 | HCR2 | CC/CT | 569/27 | 0.0226 | 50.56/47.97 | 14.2/11.1 | -0.033 | 0.37507 | -0.043 | 0.258 |
| APOC4p636 | 5’ flanking | CC/CT | 599/1 | 0.0008 | 50.49/31.26 | 14.1/NA | -0.359 | 0.05640 | -0.347 | 0.067 |
| APOC4p968/rs76214972 | 5’ UTR | AA/AG | 574/45 | 0.0362 | 50.73/49.07 | 14.1/13.8 | -0.027 | 0.34336 | -0.030 | 0.307 |
| APOC4p1150/rs148247675 | Intron 1 | AA/GA | 599/2 | 0.0017 | 50.43/72.29 | 13.8/38.2 | 0.263 | 0.04737 | 0.256 | 0.055 |
| APOC4p1229 | Intron 1 | GC/GG | 2/617 | 0.0016 | 34.08/50.66 | 5.7/14.0 | -0.357 | 0.00733 | -0.365 | 0.006 |
| APOC4p2557 | Intron 1 | CA/CC | 1/617 | 0.0008 | 34.71/50.63 | NA/14.1 | -0.274 | 0.14545 | -0.280 | 0.138 |
| APOC4p2623/rs5157 | Intron 1 | CC/CT/TT | 154/315/151 | 0.4976 | 51.28/50.4/50.19 | 14.4/14.2/13.5 | -0.008 | 0.48156 | -0.009 | 0.382 |
| APOC4p2640/rs5158 | Intron 1 | CC/CT/TT | 457/149/11 | 0.1381 | 50.38/50.84/59.86 | 13.9/14.4/15.2 | 0.023 | 0.14018 | 0.025 | 0.109 |
| APOC4p2683/rs12721109 | Intron 1 | AA/AG/GG | 1/27/582 | 0.0237 | 68.58/51.11/50.6 | NA/12.2/14.1 | 0.030 | 0.38586 | 0.056 | 0.135 |
| APOC4p2703/rs12721108 | Intron 1 | GG/GT | 607/10 | 0.0081 | 50.69/47.23 | 14.1/8.4 | -0.037 | 0.53441 | -0.058 | 0.360 |
| APOC4p3498/rs1132899 | Exon 2 | CC/CT/TT | 159/317/142 | 0.4863 | 51.44/50.19/50.27 | 14.5/14.1/13.5 | -0.009 | 0.42951 | -0.011 | 0.320 |
| APOC4p3546/rs12691089 | Exon 2 | AG/GG | 4/615 | 0.0032 | 50.34/50.61 | 13.8/14.1 | -0.010 | 0.91107 | -0.015 | 0.877 |
| APOC4p3847/rs186448850 | Intron 2 | CT/TT | 2/607 | 0.0016 | 33.96/50.54 | 5.7/14.0 | -0.357 | 0.00740 | -0.364 | 0.007 |
| APOC4p3927/rs5167 | Exon 3 | GG/TG/TT | 73/300/248 | 0.3596 | 51.38/50.12/50.9 | 14.9/13.7/14.3 | -0.001 | 0.92518 | 0.001 | 0.956 |
| APOC4p4661/rs2288912 | C4-3'/C2-5' | CC/CG/GG | 154/316/150 | 0.4968 | 50.37/50.37/51.28 | 13.3/14.3/14.3 | 0.006 | 0.58956 | 0.008 | 0.455 |
| APOC2p1591 | Intron 1 | GA/GG | 1/618 | 0.0008 | 31.06/50.64 | NA/14.0 | -0.365 | 0.05115 | -0.356 | 0.059 |
| APOC2p1851/rs12709886 | Intron 1 | GA/GG | 46/570 | 0.0372 | 49.18/50.69 | 13.4/14.1 | -0.024 | 0.41183 | -0.025 | 0.382 |
| APOC2p2870 | Intron 1 | GG/GT | 614/5 | 0.0040 | 50.58/53.91 | 14.0/25.2 | 0.026 | 0.75770 | 0.024 | 0.772 |
| APOC2p3348/rs10420434 | Intron 1 | GA/GG | 46/572 | 0.0371 | 53.83/50.33 | 16.3/13.8 | 0.046 | 0.10518 | 0.045 | 0.122 |
| APOC2p3778/rs5120 | Intron 1 | AA/AT/TT | 153/305/156 | 0.4976 | 51.27/50.52/50.26 | 14.4/14.3/13.5 | 0.007 | 0.51351 | -0.008 | 0.467 |
| APOC2p4853/rs199828513 | 3'flanking | DD/WD/WW | 315/259/42 | 0.2783 | 51.01/49.98/50.19 | 14.1/13.7/15.7 | -0.012 | 0.32077 | -0.011 | 0.364 |
| APOC2p5004/rs10421404 | 3'flanking | CC/CT/TT | 415/176/24 | 0.1823 | 50.38/50.53/54.51 | 14.1/14.1/13.7 | 0.015 | 0.25579 | 0.018 | 0.204 |
| APOC2p5310/rs7258345 | 3'flanking | GG/TG/TT | 132/303/175 | 0.4649 | 50.87/50.35/51.03 | 14.3/13.9/14.2 | -0.002 | 0.84552 | -4.18E-04 | 0.970 |
| APOC2p5398/rs12709889 | 3'flanking | AA/GA/GG | 41/251/311 | 0.2760 | 50.64/49.73/51.18 | 15.9/13.4/14.3 | -0.014 | 0.27402 | -0.013 | 0.311 |
| APOC2p5644 | 3'flanking | AG/GG | 11/583 | 0.0092 | 50.04/50.43 | 10.7/14.2 | 0.008 | 0.89469 | 0.014 | 0.805 |
| MAF is the minor allele frequency; GT is genotype; GT count is the number of individuals in each genotype group; GT_SD is standard deviation of lipid traits mean in each genotype group; *Adjusted for relevant covariates, **Adjusted for *APOE*2/E*4* SNPs in addition to the covariates. APOC1p703/rs3207187 was excluded due to missing data. | | | | | | | | | | |
