# Supplementary material for: Apolipoprotein E-C1-C4-C2 gene cluster region and inter-individual variation in plasma lipoprotein levels: a comprehensive genetic association study in two ethnic groups
Source: PLoS One. 2019 Mar 26;14(3):e0214060. doi: 10.1371/journal.pone.0214060 (PMC6435132; doi:10.1371/journal.pone.0214060)
Supplement: S16 Table — MAF is the minor allele frequency; GT is genotype; GT count is the number of individuals in each genotype group; GT_SD is standard deviation of lipid traits mean in each genotype group; *Adjusted for relevant covariates, **Adjusted for APOE*2/E*4 SNPs in addition to the covariates. Four rare variants were excluded due to missing data. (DOCX) [file pone.0214060.s016.docx]

S16 Table. Single-site association analysis results for ApoA1 in NHWs

| **Variant Name/RefSNP ID** | **Location** | **Genotype** | **GT Count** | **MAF** | **Adjusted Mean of plasma apoA1*** | **GT_SD*** | **Beta*** | **P*** | **Adj. B** | **Adj. P.** |
| --- | --- | --- | --- | --- | --- | --- | --- | --- | --- | --- |
| APOE560/rs449647 | 5'flanking | AA/AT/TT | 307/119/7 | 0.1610 | 150.06/149.21/141.94 | 34.5/29.0/24.3 | -1.6 | 0.61755 | -4.095 | 0.232 |
| APOE832/rs405509 | 5'flanking | GG/GT/TT | 115/217/103 | 0.4775 | 151.84/149.07/148.29 | 34.4/32.5/34.0 | -1.8 | 0.41735 | -0.142 | 0.956 |
| APOE1163/rs440446 | Intron 1 | CC/GC/GG | 51/207/177 | 0.3604 | 153.72/147.42/151.01 | 30.9/32.7/34.6 | -0.2 | 0.93490 | -0.086 | 0.975 |
| APOE1575/rs769448 | Intron 1 | CC/CT/TT | 414/17/1 | 0.0210 | 149.53/144.71/224.9 | 33.2/37.3/NA | 3.4 | 0.63942 | 3.977 | 0.594 |
| APOE1998rs769449 | Intron 2 | AA/AG/GG | 4/104/324 | 0.1165 | 159.38/145.16/151.06 | 32.5/35.0/32.9 | -4.4 | 0.20481 | -3.037 | 0.672 |
| APOE2440/rs769450 | Intron 2 | AA/GA/GG | 63/217/154 | 0.4015 | 152.48/148.5/149.87 | 36.4/31.6/34.5 | 0.7 | 0.76559 | 0.337 | 0.897 |
| APOE2907/rs769451 | Intron 2 | GT/TT | 9/426 | 0.0112 | 153.23/149.54 | 21.6/33.6 | 3.7 | 0.73794 | 2.538 | 0.830 |
| APOE3038/rs111833428 | Exon 3 | AG/GG | 1/432 | 0.0016 | 152.88/149.59 | NA/33.4 | 3.3 | 0.92048 | 3.366 | 0.919 |
| APOE3106/rs769452 | Exon 3 | TC/TT | 1/433 | 0.0008 | 179.31/149.54 | NA/33.4 | 29.8 | 0.36490 | 32.667 | 0.325 |
| APOE3937/rs429358 | Exon 4 | CC/CT/TT | 9/122/296 | 0.1525 | 138.97/147.69/150.56 | 34.4/36.3/32.3 | -3.7 | 0.23988 | - | - |
| APOE4075/rs7412 | Exon 4 | CC/TC/TT | 366/65/3 | 0.0806 | 148.66/154.66/149.98 | 33.4/33.6/14.5 | 5.1 | 0.20813 | - | - |
| APOE4310/rs199768005 | Exon 4 | TA/TT | 2/432 | 0.0040 | 114.13/149.7 | 11.9/33.3 | -35.7 | 0.12454 | -34.553 | 0.141 |
| APOE4737/rs117656888 | 3'flanking | CC/GC | 427/7 | 0.0081 | 149.66/144.12 | 33.2/44.1 | -5.6 | 0.65760 | -5.598 | 0.659 |
| APOE5361/rs1081106 | 3'flanking | CC/TC/TT | 3/59/373 | 0.0852 | 152.9/152.7/149.1 | 10.8/35.9/33.1 | 3.3 | 0.43184 | 2.494 | 0.568 |
| rs439401 | Intergenic | CC/CT/TT | 179/186/59 | 0.3596 | 149.41/147.97/152.86 | 32.7/35.3/28.7 | 0.9 | 0.69151 | 0.978 | 0.710 |
| APOC1rs445925 | Intergenic | AA/GA/GG | 5/86/342 | 0.1094 | 152.11/152.68/148.68 | 15.1/36.2/32.8 | 3.5 | 0.32306 | 1.237 | 0.864 |
| APOC1p698/rs72654449 | 5'flanking | CA/CC | 2/430 | 0.0040 | 145.12/149.76 | 3.7/33.5 | -4.7 | 0.84127 | -4.434 | 0.852 |
| APOC1p703/rs3207187 | 5'flanking | CC/CT | 432/1 | 0.0008 | 149.23/251.77 | 33.0/NA | 103.0 | 0.00165 | 99.082 | 0.003 |
| APOC1p720 | 5'flanking | II/WI/WW | 23/164/247 | 0.2299 | 156.43/147.39/150.62 | 39.6/33.3/32.7 | -0.5 | 0.86119 | -9.380 | 0.571 |
| APOC1p1170 | Intron 1 | GA/GG | 1/425 | 0.0008 | 168.25/149.95 | NA/33.0 | 18.3 | 0.57227 | 20.912 | 0.523 |
| APOC1p1294 | Intron 2 | AA/AC | 433/1 | 0.0008 | 149.49/176.37 | 33.4/NA | 27.0 | 0.41180 | 27.281 | 0.411 |
| APOC1p1317/rs12721048 | Intron 2 | GA/GG | 2/422 | 0.0016 | 216.08/149.57 | 43.8/33.2 | 66.9 | 0.00423 | 67.261 | 0.004 |
| APOC1p1422 | Intron 2 | GA/GG | 1/434 | 0.0016 | 123.27/149.68 | NA/33.4 | -26.5 | 0.42032 | -26.332 | 0.427 |
| APOC1p1566/rs12691088 | Intron 2 | GA/GG | 4/416 | 0.0058 | 123.04/150.35 | 24.9/33.0 | -27.5 | 0.09146 | -25.178 | 0.131 |
| APOC1p2041/rs3826688 | Intron 2 | AA/GA/GG | 55/184/184 | 0.3424 | 151.51/148.58/149.85 | 27.1/34.5/33.7 | 0.2 | 0.91759 | 0.389 | 0.883 |
| APOC1p2629 | Exon 3 | GA/GG | 1/430 | 0.0008 | 152.46/149.93 | NA/33.3 | 2.5 | 0.93833 | 2.465 | 0.941 |
| APOC1p2817 | Intron 3 | CC/CT | 420/3 | 0.0033 | 149.33/170.37 | 33.6/34.2 | 21.2 | 0.27005 | 22.269 | 0.250 |
| APOC1p3423/rs389261 | Intron 3 | GA/GG | 3/422 | 0.0025 | 128.16/149.72 | 12.2/33.6 | -21.7 | 0.25830 | -21.748 | 0.261 |
| APOC1p3494 | Intron 3 | CC/CT | 433/1 | 0.0016 | 149.65/148.26 | 33.4/NA | -1.4 | 0.96622 | -1.209 | 0.971 |
| APOC1p4334/rs12721046 | Intron 3 | AA/GA/GG | 9/124/298 | 0.1522 | 143.94/148.57/150.28 | 27.8/34.4/33.2 | -2.1 | 0.49556 | 0.919 | 0.843 |
| APOC1p5641/rs1064725 | 3'UTR | GG/GT/TT | 1/28/402 | 0.0388 | 151.6/145.08/150.14 | NA/28.1/33.8 | -4.3 | 0.47284 | -3.909 | 0.521 |
| APOC1p5926/rs56131196 | 3'flanking | AA/GA/GG | 13/147/272 | 0.1885 | 138.59/149.26/150.3 | 29.6/35.8/32.2 | -2.6 | 0.36950 | 0.337 | 0.952 |
| APOC1p6026/rs4420638 | 3'flanking | AA/GA/GG | 271/99/16 | 0.1556 | 150.61/151.88/142.57 | 32.2/36.8/30.4 | -1.1 | 0.71599 | 0.153 | 0.980 |
| rs4803770 | Intergenic | CC/GC/GG | 172/187/57 | 0.3779 | 148.59/150.64/147.18 | 34.5/32.1/35.1 | 0.02 | 0.99367 | 0.253 | 0.923 |
| HCR1p292/rs4803771 | HCR1 | CC/CG/GG | 409/17/1 | 0.0245 | 149.65/145.25/126.7 | 33.4/33.6/NA | -5.8 | 0.42728 | -6.578 | 0.373 |
| HCR1p362 | HCR1 | CA/CC | 1/422 | 0.0025 | 146/149.46 | NA/33.5 | -3.5 | 0.91586 | -3.883 | 0.907 |
| HCR1p423 | HCR1 | CC/CG/GG | 416/17/1 | 0.0258 | 149.16/160.49/169.19 | 33.2/38.2/NA | 11.1 | 0.12932 | 11.815 | 0.111 |
| HCR1p575/rs157599 | HCR1 | AA/AG | 431/3 | 0.0024 | 149.8/128.13 | 33.4/12.2 | -21.8 | 0.25308 | -21.967 | 0.254 |
| HCR1p727/rs149345 | HCR1 | TG/TT | 3/427 | 0.0024 | 128.14/149.71 | 12.2/33.4 | -21.7 | 0.25437 | -21.865 | 0.256 |
| rs5112 | *APOC1P1* | CC/GC/GG | 88/206/112 | 0.4633 | 147.66/150.29/149.24 | 32.7/33.8/35.7 | -0.7 | 0.77688 | -0.146 | 0.953 |
| rs7259004 | *APOC1P1* | CC/CG/GG | 328/93/6 | 0.1176 | 147.93/153/173.55 | 32.3/37.7/22.4 | 6.9 | 0.04602 | 7.775 | 0.057 |
| HCR2p188/rs35136575 | HCR2 | CC/GC/GG | 256/144/25 | 0.2274 | 146.45/154.06/151.77 | 32.8/34.8/29.9 | 5.2 | 0.04957 | 5.879 | 0.028 |
| HCR2p365 | HCR2 | CA/CC | 3/424 | 0.0041 | 159.91/149.39 | 31.6/33.5 | 10.6 | 0.58105 | 16.715 | 0.396 |
| HCR2p523 | HCR2 | CC/CT | 396/21 | 0.0226 | 149.23/153.04 | 33.7/31.3 | 3.8 | 0.60612 | 3.865 | 0.618 |
| APOC4p968/rs76214972 | 5’ UTR | AA/AG | 401/33 | 0.0362 | 149.5/151.52 | 33.9/26.9 | 2.0 | 0.73303 | 2.558 | 0.671 |
| APOC4p1150/rs148247675 | Intron 1 | AA/GA | 419/1 | 0.0017 | 149.68/148.08 | 33.3/NA | -1.6 | 0.96085 | -1.370 | 0.967 |
| APOC4p2557 | Intron 1 | CA/CC | 1/433 | 0.0008 | 134.92/149.59 | NA/33.4 | -14.8 | 0.65409 | -14.720 | 0.658 |
| APOC4p2623/rs5157 | Intron 1 | CC/CT/TT | 112/215/108 | 0.4976 | 151.15/147.68/151.88 | 32.5/32.5/35.8 | 0.3 | 0.87974 | 0.419 | 0.852 |
| APOC4p2640/rs5158 | Intron 1 | CC/CT/TT | 323/102/7 | 0.1381 | 150.47/147/160.11 | 33.8/32.3/27.4 | -1.4 | 0.66203 | -1.136 | 0.738 |
| APOC4p2683/rs12721109 | Intron 1 | AA/AG/GG | 1/18/406 | 0.0237 | 202.37/133.19/150.53 | NA/30.4/33.6 | -9.3 | 0.19641 | -12.623 | 0.104 |
| APOC4p2703/rs12721108 | Intron 1 | GG/GT | 424/8 | 0.0081 | 149.85/140.86 | 33.6/20.4 | -9.0 | 0.44345 | -12.439 | 0.325 |
| APOC4p3498/rs1132899 | Exon 2 | CC/CT/TT | 115/217/101 | 0.4863 | 150.64/147.15/153.66 | 32.2/32.5/36.5 | 1.3 | 0.54665 | 1.272 | 0.577 |
| APOC4p3546/rs12691089 | Exon 2 | AG/GG | 2/432 | 0.0032 | 150.3/149.65 | 3.8/33.4 | 0.6 | 0.97779 | 0.851 | 0.971 |
| APOC4p3927/rs5167 | Exon 3 | GG/TG/TT | 51/216/168 | 0.3596 | 150.17/148.48/150.92 | 32.6/32.5/34.7 | -1.0 | 0.67218 | -0.950 | 0.699 |
| APOC4p4661/rs2288912 | C4-3'/C2-5' | CC/CG/GG | 112/214/109 | 0.4968 | 152.58/146.48/152.73 | 35.5/32.5/32.4 | 0.03 | 0.98800 | 0.178 | 0.937 |
| APOC2p1851/rs12709886 | Intron 1 | GA/GG | 36/395 | 0.0372 | 154.75/148.94 | 29.9/33.6 | 5.8 | 0.30785 | 6.404 | 0.269 |
| APOC2p2870 | Intron 1 | GG/GT | 430/4 | 0.0040 | 149.98/113.84 | 33.2/43.7 | -36.2 | 0.02793 | -34.940 | 0.036 |
| APOC2p3348/rs10420434 | Intron 1 | GA/GG | 31/401 | 0.0371 | 156.21/149.31 | 35.8/32.8 | 6.9 | 0.25290 | 5.635 | 0.369 |
| APOC2p3778/rs5120 | Intron 1 | AA/AT/TT | 110/206/112 | 0.4976 | 153.3/146.82/152.67 | 32.1/32.4/35.5 | 0.3 | 0.89544 | 0.427 | 0.849 |
| APOC2p4853/rs199828513 | 3'flanking | DD/WD/WW | 222/181/29 | 0.2783 | 151.65/146.49/150.53 | 35.5/30.1/33.1 | -2.8 | 0.26920 | -2.835 | 0.279 |
| APOC2p5004/rs10421404 | 3'flanking | CC/CT/TT | 289/126/17 | 0.1823 | 149.13/148.48/158.91 | 32.7/34.8/28.8 | 1.6 | 0.56044 | 1.925 | 0.505 |
| APOC2p5310/rs7258345 | 3'flanking | GG/TG/TT | 95/213/123 | 0.4649 | 151.44/146.73/153.03 | 30.9/32.7/35.6 | -1.2 | 0.60410 | -1.134 | 0.619 |
| APOC2p5398/rs12709889 | 3'flanking | AA/GA/GG | 28/174/220 | 0.2760 | 150.8/146.25/151.97 | 33.6/30.5/35.8 | -3.1 | 0.23298 | -3.309 | 0.218 |
| APOC2p5644 | 3'flanking | AG/GG | 9/406 | 0.0092 | 156.78/149.16 | 27.7/33.8 | 7.7 | 0.49290 | 9.445 | 0.404 |

MAF is the minor allele frequency; GT is genotype; GT count is the number of individuals in each genotype group; GT_SD is standard deviation of lipid traits mean in each genotype group; *Adjusted for relevant covariates, **Adjusted for *APOE*2/E*4* SNPs in addition to the covariates. Four rare variants were excluded due to missing data.
