# Supplementary material for: Apolipoprotein E-C1-C4-C2 gene cluster region and inter-individual variation in plasma lipoprotein levels: a comprehensive genetic association study in two ethnic groups
Source: PLoS One. 2019 Mar 26;14(3):e0214060. doi: 10.1371/journal.pone.0214060 (PMC6435132; doi:10.1371/journal.pone.0214060)
Supplement: S17 Table — MAF is the minor allele frequency; GT is genotype; GT count is the number of individuals in each genotype group; GT_SD is standard deviation of lipid traits mean in each genotype group; *Adjusted for relevant covariates, **Adjusted for APOE*2/E*4 SNPs in addition to the covariates. (DOCX) [file pone.0214060.s017.docx]

S17 Table. Single-site association analysis results for TG in NHWs

| **Variant Name/RefSNP ID** | **Location** | **Genotype** | **GT Count** | **MAF** | **Adjusted Mean of plasma TG*** | **GT_SD*** | **Beta*** | **P*** | **Adj. B.** | **Adj. P.** |
| --- | --- | --- | --- | --- | --- | --- | --- | --- | --- | --- |
| APOE560/rs449647 | 5'flanking | AA/AT/TT | 433/172/12 | 0.1610 | 139.61/134.35/133.8 | 68.2/59.3/67.0 | -0.021 | 0.52293 | -0.027 | 0.434 |
| APOE832/rs405509 | 5'flanking | GG/GT/TT | 170/307/141 | 0.4775 | 148.79/135.95/129.7 | 71.4/63.4/62.4 | -0.067 | 0.00333 | -0.077 | 0.002 |
| APOE1163/rs440446 | Intron 1 | CC/GC/GG | 75/295/249 | 0.3604 | 123.14/135.63/145.12 | 56.1/65.4/67.8 | -0.076 | 0.00181 | -0.087 | 0.001 |
| APOE1575/rs769448 | Intron 1 | CC/CT/TT | 591/24/1 | 0.0210 | 138.4/130.89/34.9 | 66.3/48.8/NA | -0.093 | 0.23228 | -0.094 | 0.229 |
| APOE1998/rs769449 | Intron 2 | AA/AG/GG | 6/131/477 | 0.1165 | 179.59/135.92/138 | 80.3/63.7/66.0 | 0.015 | 0.68668 | 0.049 | 0.494 |
| APOE2440/rs769450 | Intron 2 | AA/GA/GG | 95/305/215 | 0.4015 | 148.93/137.7/132.05 | 67.2/64.5/65.2 | 0.063 | 0.00823 | 0.083 | 0.002 |
| APOE2907/rs769451 | Intron 2 | GT/TT | 14/605 | 0.0112 | 124.87/138.24 | 48.8/66.1 | -0.055 | 0.61567 | -0.083 | 0.463 |
| APOE3038/rs111833428 | Exon 3 | AG/GG | 2/612 | 0.0016 | 146.64/138.14 | 10.6/66.0 | 0.160 | 0.57655 | 0.167 | 0.560 |
| APOE3106/rs769452 | Exon 3 | TC/TT | 1/616 | 0.0008 | 101.15/137.96 | NA/65.7 | -0.219 | 0.58650 | -0.228 | 0.572 |
| APOE3937/rs429358 | Exon 4 | CC/CT/TT | 14/159/437 | 0.1525 | 142.88/138.64/137.37 | 74.6/64.4/65.4 | 0.012 | 0.70692 | - | - |
| APOE4075/rs7412 | Exon 4 | CC/TC/TT | 521/92/3 | 0.0806 | 137.8/138.76/179.42 | 65.0/70.1/93.6 | 0.014 | 0.74378 | - | - |
| APOE4310/rs199768005 | Exon 4 | TA/TT | 5/613 | 0.0040 | 78.12/138.52 | 20.5/65.9 | -0.404 | 0.02603 | -0.398 | 0.028 |
| APOE4528 /rs374329439 | 3' UTR | CC/CT | 618/1 | 0.0008 | 137.65/311.89 | 65.5/NA | 0.916 | 0.02316 | 0.930 | 0.021 |
| APOE4737/rs117656888 | 3'flanking | CC/GC | 606/10 | 0.0081 | 137.88/153.84 | 65.6/79.0 | 0.085 | 0.50843 | 0.094 | 0.469 |
| APOE5361/rs1081106 | 3'flanking | CC/TC/TT | 4/98/516 | 0.0852 | 186.18/134.11/138.33 | 76.3/65.6/65.7 | -0.006 | 0.89282 | -0.006 | 0.884 |
| rs439401 | Intergenic | CC/CT/TT | 254/268/83 | 0.3596 | 143.91/135.33/129.53 | 66.6/65.0/63.0 | -0.055 | 0.01936 | -0.072 | 0.006 |
| APOC1rs445925 | Intergenic | AA/GA/GG | 7/120/487 | 0.1094 | 150.57/139.2/137.03 | 62.1/73.1/63.7 | 0.008 | 0.82510 | -0.071 | 0.338 |
| APOC1p698/rs72654449 | 5'flanking | CA/CC | 5/609 | 0.0040 | 109.41/137.85 | 29.6/65.9 | -0.173 | 0.34254 | -0.162 | 0.374 |
| APOC1p703/rs3207187 | 5'flanking | CC/CT | 615/1 | 0.0008 | 138.15/118.38 | 65.9/NA | -0.071 | 0.86073 | -0.082 | 0.840 |
| APOC1p720 | 5'flanking | II/WI/WW | 31/222/365 | 0.2299 | 142.81/138.71/137.05 | 61.0/70.8/63.1 | 0.009 | 0.73507 | -0.136 | 0.411 |
| APOC1p1170 | Intron 1 | GA/GG | 1/605 | 0.0008 | 70.3/138.55 | NA/66.1 | -0.614 | 0.12814 | -0.619 | 0.125 |
| APOC1p1294 | Intron 2 | AA/AC | 614/1 | 0.0008 | 137.94/119.94 | 65.9/NA | -0.101 | 0.80361 | -0.092 | 0.820 |
| APOC1p1317/rs12721048 | Intron 2 | GA/GG | 2/603 | 0.0016 | 138.26/137.77 | 81.3/65.1 | -0.011 | 0.97010 | -0.011 | 0.970 |
| APOC1p1422 | Intron 2 | GA/GG | 2/617 | 0.0016 | 85.16/138.1 | 46.7/65.8 | -0.495 | 0.08380 | -0.489 | 0.087 |
| APOC1p1566/rs12691088 | Intron 2 | GA/GG | 7/594 | 0.0058 | 130.06/138.08 | 78.5/65.9 | -0.077 | 0.61826 | -0.085 | 0.592 |
| APOC1p2041/rs3826688 | Intron 2 | AA/GA/GG | 72/267/263 | 0.3424 | 122.52/133.62/144.45 | 59.9/63.3/66.8 | -0.079 | 0.00113 | -0.094 | 0.001 |
| APOC1p2629 | Exon 3 | GA/GG | 1/614 | 0.0008 | 124.61/138.12 | NA/65.8 | -0.018 | 0.96335 | -0.011 | 0.979 |
| APOC1p2817 | Intron 3 | CC/CT | 599/4 | 0.0033 | 138.22/132.67 | 66.3/26.9 | 0.043 | 0.83338 | 0.049 | 0.809 |
| APOC1p3423/rs389261 | Intron 3 | GA/GG | 3/601 | 0.0025 | 132.55/138.06 | 72.0/66.0 | -0.024 | 0.91687 | -0.010 | 0.965 |
| APOC1p3494 | Intron 3 | CC/CT | 615/2 | 0.0016 | 137.8/197.85 | 65.7/84.9 | 0.398 | 0.16425 | 0.398 | 0.163 |
| APOC1p4334/rs12721046 | Intron 3 | AA/GA/GG | 13/159/435 | 0.1522 | 161.1/137.57/137.8 | 71.8/63.8/66.4 | 0.023 | 0.47873 | 0.033 | 0.494 |
| APOC1p5641/rs1064725 | 3'UTR | GG/GT/TT | 1/45/568 | 0.0388 | 108.18/134.02/138.5 | NA/68.9/65.6 | -0.050 | 0.40638 | -0.049 | 0.417 |
| APOC1p5773 | 3'flanking | GA/GG | 1/600 | 0.0008 | 313.01/137.37 | NA/65.4 | 0.925 | 0.02109 | 0.937 | 0.019 |
| APOC1p5926/rs56131196 | 3'flanking | AA/GA/GG | 19/194/401 | 0.1885 | 144.93/137.9/137.39 | 69.4/66.6/65.4 | 0.009 | 0.76774 | 0.009 | 0.877 |
| APOC1p6026/rs4420638 | 3'flanking | AA/GA/GG | 402/128/22 | 0.1556 | 137.4/139.11/153.86 | 65.2/63.6/77.5 | 0.027 | 0.39236 | 0.048 | 0.456 |
| rs4803770 | Intergenic | CC/GC/GG | 226/281/84 | 0.3779 | 134.33/140.66/144.51 | 66.7/67.0/63.2 | 0.043 | 0.08010 | 0.061 | 0.023 |
| HCR1p292/rs4803771 | HCR1 | CC/CG/GG | 579/28/1 | 0.0245 | 138.16/130.59/300.06 | 65.5/62.2/NA | 0.017 | 0.81882 | 0.018 | 0.804 |
| HR1p362 | HCR1 | CA/CC | 3/601 | 0.0025 | 186.45/137.65 | 81.3/65.5 | 0.296 | 0.20302 | 0.300 | 0.197 |
| HR1p423 | HCR1 | CC/CG/GG | 586/30/1 | 0.0258 | 139.12/115.81/142.97 | 66.3/51.5/NA | -0.124 | 0.07997 | -0.104 | 0.151 |
| HR1p575/rs157599 | HCR1 | AA/AG | 614/3 | 0.0024 | 138.02/132.76 | 65.8/72.0 | -0.022 | 0.92604 | -0.011 | 0.961 |
| HR1p727/rs149345 | HCR1 | TG/TT | 3/607 | 0.0024 | 131.91/137.89 | 72.0/65.9 | -0.028 | 0.90531 | -0.018 | 0.939 |
| rs5112 | *APOC1P1* | CC/GC/GG | 121/282/165 | 0.4633 | 134.65/136.13/139.61 | 68.2/64.3/64.0 | -0.024 | 0.32168 | -0.019 | 0.444 |
| rs7259004 | *APOC1P1* | CC/CG/GG | 473/128/8 | 0.1176 | 135.49/147.31/121.5 | 63.6/74.3/42.6 | 0.045 | 0.21034 | 0.051 | 0.224 |
| HR2p188/rs35136575 | HCR2 | CC/GC/GG | 368/201/37 | 0.2274 | 138.5/136.9/132.18 | 63.1/69.0/61.4 | -0.029 | 0.28305 | -0.029 | 0.283 |
| HR2p365 | HCR2 | CA/CC | 5/602 | 0.0041 | 110.77/138.24 | 42.7/66.1 | -0.202 | 0.26595 | -0.213 | 0.248 |
| HR2p523 | HCR2 | CC/CT | 568/26 | 0.0226 | 138.42/139.96 | 66.2/60.6 | 0.024 | 0.76638 | 0.034 | 0.678 |
| APOC4p636 | 5’ flanking | CC/CT | 597/1 | 0.0008 | 137.96/275.05 | 65.6/NA | 0.725 | 0.07317 | 0.720 | 0.075 |
| APOC4p968/rs76214972 | 5’ UTR | AA/AG | 572/45 | 0.0362 | 138/137.87 | 66.3/59.5 | 0.015 | 0.80852 | 0.018 | 0.780 |
| APOC4p1150/rs148247675 | Intron 1 | AA/GA | 597/2 | 0.0017 | 137.73/196.79 | 65.5/84.9 | 0.391 | 0.16983 | 0.394 | 0.168 |
| APOC4p1229 | Intron 1 | GC/GG | 2/615 | 0.0016 | 245.77/137.64 | 24.0/65.6 | 0.678 | 0.01859 | 0.672 | 0.020 |
| APOC4p2557 | Intron 1 | CA/CC | 1/615 | 0.0008 | 135.71/137.99 | NA/65.9 | 0.120 | 0.76810 | 0.112 | 0.782 |
| APOC4p2623/rs5157 | Intron 1 | CC/CT/TT | 155/311/152 | 0.4976 | 135.81/136.86/142.57 | 65.79/62.7/71.9 | 0.020 | 0.37510 | 0.025 | 0.290 |
| APOC4p2640/rs5158 | Intron 1 | CC/CT/TT | 456/148/11 | 0.1381 | 139.65/133.48/121.82 | 67.4/61.5/58.6 | -0.041 | 0.22194 | -0.046 | 0.177 |
| APOC4p2683/rs12721109 | Intron 1 | AA/AG/GG | 1/26/581 | 0.0237 | 69.01/121.05/139.06 | NA/67.2/65.9 | -0.161 | 0.03289 | -0.207 | 0.010 |
| APOC4p2703/rs12721108 | Intron 1 | GG/GT | 605/10 | 0.0081 | 137.98/111.71 | 65.7/43.4 | -0.151 | 0.23842 | -0.113 | 0.402 |
| APOC4p3498/rs1132899 | Exon 2 | CC/CT/TT | 160/313/143 | 0.4863 | 136.29/135.87/143.8 | 65.2/61.7/72.3 | 0.022 | 0.34646 | 0.022 | 0.336 |
| APOC4p3546/rs12691089 | Exon 2 | AG/GG | 4/613 | 0.0032 | 115.55/138.14 | 63.9/65.8 | -0.155 | 0.44443 | -0.151 | 0.457 |
| APOC4p3847/rs186448850 | Intron 2 | CT/TT | 2/605 | 0.0016 | 246.13/137.59 | 24.0/65.3 | 0.681 | 0.01713 | 0.669 | 0.019 |
| APOC4p3927/rs5167 | Exon 3 | GG/TG/TT | 74/297/248 | 0.3596 | 139.36/136.28/139.49 | 68.8/61.8/69.4 | -0.004 | 0.87590 | 0.001 | 0.974 |
| APOC4p4661/rs2288912 | C4-3'/C2-5' | CC/CG/GG | 155/312/151 | 0.4968 | 143.66/135.77/136.19 | 73.4/61.6/65.7 | -0.020 | 0.38126 | -0.021 | 0.378 |
| APOC2p1591 | Intron 1 | GA/GG | 1/616 | 0.0008 | 275.28/137.77 | NA/65.6 | 0.729 | 0.07120 | 0.725 | 0.073 |
| APOC2p1851/rs12709886 | Intron 1 | GA/GG | 46/568 | 0.0372 | 140.23/137.95 | 59.2/66.5 | 0.034 | 0.58453 | 0.036 | 0.565 |
| APOC2p2870 | Intron 1 | GG/GT | 612/5 | 0.0040 | 137.83/157.24 | 65.7/79.6 | 0.119 | 0.51156 | 0.119 | 0.511 |
| APOC2p3348/rs10420434 | Intron 1 | GA/GG | 45/571 | 0.0371 | 130.78/138.84 | 74.3/65.0 | -0.085 | 0.17358 | -0.093 | 0.141 |
| APOC2p3778/rs5120 | Intron 1 | AA/AT/TT | 154/302/156 | 0.4976 | 134.51/136.63/143.81 | 63.7/62.5/73.2 | -0.025 | 0.28092 | 0.023 | 0.319 |
| APOC2p4853/rs199828513 | 3'flanking | DD/WD/WW | 314/258/42 | 0.2783 | 138.01/138.21/139.6 | 68.6/62.4/67.6 | 0.011 | 0.67415 | 0.016 | 0.540 |
| APOC2p5004/rs10421404 | 3'flanking | CC/CT/TT | 413/176/24 | 0.1823 | 139.84/134.54/131.31 | 68.0/61.5/63.5 | -0.027 | 0.35914 | -0.030 | 0.322 |
| APOC2p5310/rs7258345 | 3'flanking | GG/TG/TT | 133/301/175 | 0.4649 | 137.94/134.99/141.53 | 64.4/61.1/72.9 | -0.005 | 0.81914 | -0.005 | 0.845 |
| APOC2p5398/rs12709889 | 3'flanking | AA/GA/GG | 41/250/310 | 0.2760 | 139.76/138.57/137.76 | 67.7/62.7/68.7 | 0.014 | 0.59738 | 0.018 | 0.508 |
| APOC2p5644 | 3'flanking | AG/GG | 11/581 | 0.0092 | 123.92/138.57 | 53.6/66.2 | -0.101 | 0.41034 | -0.101 | 0.412 |

MAF is the minor allele frequency; GT is genotype; GT count is the number of individuals in each genotype group; GT_SD is standard deviation of lipid traits mean in each genotype group; *Adjusted for relevant covariates, **Adjusted for *APOE*2/E*4* SNPs in addition to the covariates
