# Supplementary material for: Apolipoprotein E-C1-C4-C2 gene cluster region and inter-individual variation in plasma lipoprotein levels: a comprehensive genetic association study in two ethnic groups
Source: PLoS One. 2019 Mar 26;14(3):e0214060. doi: 10.1371/journal.pone.0214060 (PMC6435132; doi:10.1371/journal.pone.0214060)
Supplement: S18 Table — MAF is the minor allele frequency; GT is genotype; GT count is the number of individuals in each genotype group; GT_SD is standard deviation of lipid traits mean in each genotype group; *Adjusted for relevant covariates, **Adjusted for APOE*2/E*4 SNPs in addition to the covariates. APOC2p5771 is excluded due to missing data. (DOCX) [file pone.0214060.s018.docx]

S18 Table. Single-site association analysis results for LDL-C in ABs

| **Variant Name/RefSNP ID** | **Locatİon** | **Genotype** | **GT Count** | **MAF** | **Adjusted Mean of plasma LDL-C*** | **GT_SD*** | **Beta*** | **P*** | **Adj. B.** | **Adj. P.** |
| --- | --- | --- | --- | --- | --- | --- | --- | --- | --- | --- |
| APOE73/rs1081101 | 5'flanking | CC/CT/TT | 660/82/5 | 0.0611 | 109.82/104.38/99.53 | 34.8/33.9/34.1 | -0.62 | 0.111 | -0.683 | 0.083 |
| APOE173 | 5'flanking | AA/GA | 746/3 | 0.0020 | 109.16/99.49 | 34.7/40.5 | -1.14 | 0.598 | -0.932 | 0.663 |
| APOE308/rs769445 | 5'flanking | CC/TC | 739/10 | 0.0072 | 109.16/106.69 | 34.6/42.5 | -0.37 | 0.758 | -0.427 | 0.730 |
| APOE560/rs449647 | 5'flanking | AA/AT/TT | 300/334/105 | 0.3663 | 105.33/110.93/115.09 | 33.1/36.0/33.4 | 0.58 | 0.003 | 0.414 | 0.051 |
| APOE618 | 5'flanking | GC/GG | 1/763 | 0.0006 | 83.39/109.35 | NA/34.5 | -2.85 | 0.443 | -2.892 | 0.431 |
| APOE624/rs769446 | 5'flanking | TC/TT | 9/690 | 0.0077 | 85.79/109.43 | 33.9/34.4 | -2.86 | 0.021 | -1.095 | 0.401 |
| APOE832/rs405509 | 5'flanking | GG/GT/TT | 428/267/61 | 0.2561 | 105.85/114.09/111.31 | 34.1/36.1/30.7 | 0.60 | 0.004 | 0.451 | 0.053 |
| APOE1109/rs9282609 | Splice site | CC/TC/TT | 686/55/4 | 0.0415 | 109.01/109/110.06 | 34.7/33.6/31.7 | 0.05 | 0.918 | 0.004 | 0.993 |
| APOE1163/rs440446 | Intron 1 | CC/CG/GG | 8/126/569 | 0.1004 | 106.24/113.53/108.52 | 22.4/36.0/34.7 | 0.44 | 0.179 | 0.488 | 0.147 |
| APOE1231 | Intron 1 | GA/GG | 19/729 | 0.0125 | 102.61/109.3 | 30.1/34.8 | -0.71 | 0.414 | -1.060 | 0.228 |
| APOE1279/rs877973 | Intron 1 | AA/CA/CC | 3/81/664 | 0.0597 | 75.36/111.5/109.08 | 40.0/39.9/34.0 | -0.15 | 0.721 | -0.329 | 0.430 |
| APOE1539/rs184686013 | Intron 1 | AA/AG/GG | 733/11/1 | 0.0086 | 109.19/102.23/77.29 | 34.6/30.1/NA | -1.03 | 0.286 | -0.854 | 0.389 |
| APOE2072/rs189660912 | Intron 2 | GA/GG | 12/734 | 0.0079 | 103.59/109.27 | 21.4/34.9 | -0.50 | 0.643 | -0.366 | 0.732 |
| APOE2269/rs61357706 | Intron 2 | GA/GG | 25/723 | 0.0169 | 92.91/110.08 | 30.6/34.6 | -2.05 | 0.006 | -2.270 | 0.003 |
| APOE2440/rs769450 | Intron 2 | AA/AG/GG | 107/308/261 | 0.3870 | 112.51/109.87/108.31 | 37.6/35.0/33.7 | 0.20 | 0.341 | 0.203 | 0.408 |
| APOE3673/rs769453 | Intron 3 | CC/GC | 738/9 | 0.0066 | 109.07/106.76 | 34.4/44.5 | -0.37 | 0.765 | -0.444 | 0.719 |
| APOE3937/rs429358 | Exon 4 | CC/CT/TT | 58/285/406 | 0.2656 | 109.67/113.33/106.49 | 31.8/35.5/34.3 | 0.46 | 0.032 | - | - |
| APOE4036/rs769455 | Exon 4 | CC/TC/TT | 708/27/1 | 0.0200 | 109.86/91.6/71.5 | 34.4/28.0/NA | -2.23 | 0.001 | -2.439 | 3.49E-04 |
| APOE4075/rs7412 | Exon 4 | AA/GA/GG | 2/84/670 | 0.0605 | 50.48/95.05/111.16 | 6.8/30.7/34.7 | -2.05 | 5.35E-07 | - | - |
| APOE4569 | 3'UTR | GG/GT | 746/1 | 0.0007 | 109.02/201.25 | 34.6/NA | 8.87 | 0.017 | 8.226 | 0.026 |
| APOE5223 | 3'flanking | CC/CG | 758/8 | 0.0051 | 109.45/90.42 | 34.5/28.8 | -2.25 | 0.087 | -2.410 | 0.064 |
| APOE5231 | 3'flanking | GG/GT/TT | 2/36/708 | 0.0270 | 93.74/109.69/109.12 | 32.2/36.0/34.7 | -0.10 | 0.859 | -0.337 | 0.560 |
| rs439401 | Intergenic | CC/CT/TT | 587/130/15 | 0.1092 | 108.47/112.63/109.57 | 34.9/34.5/23.2 | 0.39 | 0.193 | 0.365 | 0.235 |
| APOC1rs445925 | Intergenic | AA/GA/GG | 68/310/367 | 0.2990 | 108.26/109.76/109.34 | 35.4/33.8/34.7 | -0.01 | 0.951 | 0.667 | 0.101 |
| APOC1p720ins4/rs11568822 | 5'flanking | II/WI/WW | 60/279/396 | 0.2737 | 100.36/107.87/111.26 | 36.7/34.7/34.5 | -0.57 | 0.008 | -0.275 | 0.235 |
| APOC1p894/rs190454394 | 5'flanking | CC/CT | 740/3 | 0.0020 | 108.86/142.2 | 34.6/39.3 | 3.70 | 0.085 | 3.692 | 0.082 |
| APOC1p1166/rs72654452 | Intron 1 | CC/CT/TT | 715/42/2 | 0.0308 | 108.8/116.01/91.06 | 34.3/38.4/11.9 | 0.49 | 0.368 | 0.441 | 0.428 |
| APOC1p1331/rs10408994 | Intron 2 | AG/GG | 98/639 | 0.0666 | 113.82/108.81 | 32.4/34.9 | 0.62 | 0.124 | 0.697 | 0.089 |
| APOC1p1526/rs5114 | Intron 2 | CC/CT/TT | 657/77/3 | 0.0579 | 108.95/112.62/75.24 | 33.9/40.4/40.0 | -0.05 | 0.905 | -0.241 | 0.570 |
| APOC1p1642 | Intron 2 | CC/CT | 742/16 | 0.0103 | 109.44/100.1 | 34.6/31.8 | -1.05 | 0.265 | -1.403 | 0.139 |
| APOC1p1684/rs12709881 | Intron 2 | AA/GA/GG | 8/130/617 | 0.0973 | 85.21/108.63/109.75 | 39.5/37.5/33.9 | -0.51 | 0.118 | -0.682 | 0.037 |
| APOC1p3358 | Intron 3 | AA/GA | 706/3 | 0.0021 | 109.04/105.39 | 35.0/16.6 | -0.25 | 0.907 | 0.434 | 0.840 |
| APOC1p3423/rs389261 | Intron 3 | AA/GA/GG | 79/317/318 | 0.3310 | 110.34/110.1/108.09 | 31.8/34.3/36.0 | 0.19 | 0.356 | 0.004 | 0.986 |
| APOC1p3573/rs10424339 | Intron 3 | AA/GA/GG | 16/174/541 | 0.1396 | 102.24/109.75/109.18 | 27.8/31.9/35.7 | -0.02 | 0.937 | -0.018 | 0.951 |
| APOC1p5006/rs112528434 | Intron 3 | GG/GT/TT | 573/101/7 | 0.0850 | 110.62/110.94/87.02 | 34.3/38.4/39.6 | -0.38 | 0.289 | -0.554 | 0.129 |
| APOC1p5053/rs12721052 | Intron 3 | DD/WD/WW | 42/247/465 | 0.2200 | 117.3/109.24/108.24 | 37.7/35.8/33.3 | 0.30 | 0.190 | 0.277 | 0.243 |
| APOC1p5667/rs12721054 | 3'UTR | AA/GA/GG | 507/163/18 | 0.1446 | 111.06/107.84/87.84 | 34.4/37.1/28.9 | -0.77 | 0.006 | -0.630 | 0.027 |
| APOC1p5926/rs56131196 | 3'flanking | AA/AG/GG | 21/214/494 | 0.1745 | 107.45/108.09/109.77 | 44.0/34.7/34.3 | -0.22 | 0.392 | -0.269 | 0.306 |
| rs4803770 | Intergenic | CC/GC/GG | 393/278/57 | 0.2695 | 108.62/108.21/121.09 | 33.9/34.9/36.8 | 0.38 | 0.081 | 0.289 | 0.202 |
| HCR1p424/rs117664574 | HCR1 | AG/GG | 11/733 | 0.0073 | 104.69/109.12 | 38.8/34.6 | -0.58 | 0.608 | -0.692 | 0.535 |
| HCR1p575/rs157599 | HCR1 | AA/AG/GG | 282/309/92 | 0.3595 | 108.34/108.93/111.46 | 35.5/33.9/32.7 | 0.17 | 0.407 | -0.015 | 0.943 |
| rs5112 | *APOC1P1* | CC/GC/GG | 201/331/166 | 0.4797 | 110.1/109.54/111.98 | 34.7/35.0/33.7 | 0.10 | 0.607 | 0.094 | 0.651 |
| rs7259004 | *APOC1P1* | CC/CG/GG | 74/289/368 | 0.3020 | 111.32/108/109.83 | 33.5/35.4/34.3 | -0.02 | 0.906 | 0.124 | 0.566 |
| HCR2p188/rs35136575 | HCR2 | CC/GC/GG | 530/178/25 | 0.1546 | 109.72/107.36/109.53 | 34.7/34.6/37.3 | -0.16 | 0.533 | -0.106 | 0.683 |
| HCR2p286 | HCR2 | AA/AG/GG | 3/62/676 | 0.0457 | 104.96/117.78/108.53 | 19.3/39.9/34.1 | 0.79 | 0.079 | 0.810 | 0.073 |
| HCR2p523/rs118004808 | HCR2 | CC/TC | 742/4 | 0.0026 | 109.04/133.43 | 34.6/32.2 | 2.74 | 0.142 | 2.681 | 0.146 |
| APOC4p368 | 5’ flanking | TC/TT | 3/749 | 0.0019 | 116.63/109.09 | 25.2/34.7 | 1.04 | 0.628 | 0.882 | 0.679 |
| APOC4p637/rs113814026 | 5’ flanking | GG/GT/TT | 685/68/1 | 0.0452 | 109.16/110.12/33.76 | 34.2/35.9/NA | -0.23 | 0.613 | -0.180 | 0.700 |
| APOC4p757/rs12721105 | 5’ flanking | GG/GT/TT | 708/53/2 | 0.0376 | 109.13/109.85/115.39 | 34.6/34.7/0.9 | 0.14 | 0.778 | 0.062 | 0.901 |
| APOC4p1088 | Intron 1 | GT/TT | 2/736 | 0.0013 | 87.39/109.34 | 41.9/34.6 | -2.69 | 0.305 | -2.710 | 0.295 |
| APOC4p1130 | Intron 1 | CT/TT | 1/744 | 0.0007 | 100.46/109.12 | NA/34.7 | -0.71 | 0.849 | -0.718 | 0.845 |
| APOC4p1192/rs113745034 | Intron 1 | GA/GG | 18/695 | 0.0124 | 104.03/109.18 | 39.1/34.7 | -0.63 | 0.482 | -0.510 | 0.564 |
| APOC4p1325del3 | Intron 1 | WD/WW | 36/706 | 0.0245 | 114.84/108.99 | 34.4/34.6 | 0.70 | 0.271 | 0.695 | 0.275 |
| APOC4p1430ins | Intron 1 | II/WI/WW | 1/45/622 | 0.0341 | 104.28/110.81/108.33 | NA/29.3/34.8 | 0.32 | 0.561 | 0.374 | 0.490 |
| APOC4p2099/rs111339708 | Intron 1 | GG/GT | 735/22 | 0.0141 | 109.41/102.23 | 34.6/36.2 | -0.83 | 0.303 | -0.620 | 0.458 |
| APOC4p2467/rs115225947 | Intron 1 | GA/GG | 21/736 | 0.0141 | 115.18/108.98 | 38.8/34.5 | 0.68 | 0.406 | 0.750 | 0.357 |
| APOC4p2559/rs5155 | Intron 1 | CC/CT/TT | 616/132/7 | 0.0986 | 109.2/108.23/93.93 | 34.2/35.1/23.7 | -0.27 | 0.403 | -0.149 | 0.652 |
| APOC4p2607/rs5156 | Intron 1 | AG/GG | 19/702 | 0.0129 | 100.55/109.48 | 37.2/34.9 | -1.04 | 0.235 | -0.845 | 0.341 |
| APOC4p2623/rs5157 | Intron 1 | CC/CT/TT | 506/220/20 | 0.1723 | 107.96/111.8/106.4 | 34.5/35.4/31.9 | 0.28 | 0.270 | 0.360 | 0.166 |
| APOC4p2640/rs5158 | Intron 1 | CC/CT | 723/31 | 0.0213 | 108.83/113.04 | 34.6/31.4 | 0.55 | 0.414 | 0.568 | 0.406 |
| APOC4p2678/rs148564866 | Intron 1 | GC/GG | 13/726 | 0.0086 | 103.53/109.31 | 32.1/34.7 | -0.61 | 0.555 | -1.079 | 0.296 |
| APOC4p2767/rs127721107 | Intron 1 | GG/GT | 697/38 | 0.0254 | 109.12/112.5 | 34.6/36.8 | 0.35 | 0.579 | 0.320 | 0.606 |
| APOC4p3348 | Intron 1 | AG/GG | 1/742 | 0.0007 | 84.76/109.22 | NA/34.6 | -2.86 | 0.440 | -0.974 | 0.791 |
| APOC2p75APOC4p3380/rs12721104 | C4-Intron 1 | AA/GA/GG | 14/174/562 | 0.1368 | 106.69/109.64/108.85 | 29.7/36.1/34.2 | 0.02 | 0.936 | 0.114 | 0.691 |
| APOC2p194APOC4p3498/rs1132899 | C4-Exon 2 | CC/CT/TT | 434/282/40 | 0.2368 | 107.42/112.24/104.09 | 33.9/35.5/32.8 | 0.21 | 0.361 | 0.243 | 0.288 |
| APOC2p228/rs5164 | C4-Exon 2 | AG/GA/GG | 9/1/730 | 0.0066 | 101.28/110.29/109.33 | 31.4/NA/34.7 | -0.78 | 0.513 | -0.900 | 0.469 |
| APOC2p288APOC4p3592/rs12691090 | C4-Exon 2 | CC/CT | 698/41 | 0.0272 | 109.21/109.85 | 34.5/38.0 | -0.02 | 0.975 | 0.114 | 0.848 |
| APOC2p396APOC4p3700 | C4-Intron 2 | GA/GG | 1/719 | 0.0007 | 68.26/109.69 | NA/34.8 | -4.47 | 0.232 | -4.708 | 0.202 |
| APOC2p488APOC4p3792/rs5165 | C4-Intron 2 | GA/GG | 22/715 | 0.0146 | 109.02/109.11 | 24.8/34.9 | 0.13 | 0.873 | 0.181 | 0.821 |
| APOC2p623APOC4p3927/rs5167 | C4-Exon 3 | GG/GT/TT | 164/367/223 | 0.4594 | 106.02/108.97/112.39 | 33.8/34.6/34.8 | -0.37 | 0.053 | -0.419 | 0.029 |
| APOC2p665APOC4p3969/rs138548797 | C4-Exon 3 | AA/CA | 729/13 | 0.0086 | 108.93/110.64 | 34.5/39.0 | 0.18 | 0.865 | 0.552 | 0.604 |
| APOC2p708APOC4p4012 | C4-Exon 3 | GA/GG | 1/739 | 0.0007 | 96.57/109.29 | NA/34.6 | -1.33 | 0.721 | -1.260 | 0.731 |
| APOC2p853APOC4p4157/rs10425530 | C4-3' UTR | AA/GA/GG | 7/153/587 | 0.1100 | 95/109.14/109.57 | 17.8/35.7/34.4 | -0.20 | 0.531 | -0.253 | 0.418 |
| APOC2p1042APOC4p4346/rs12709885 | C4-3'/C2-5' | AA/TA/TT | 720/23/1 | 0.0178 | 109.64/95.95/116.41 | 34.8/26.9/NA | -1.25 | 0.085 | -1.152 | 0.123 |
| APOC2p1187APOC4p4491/rs111782345 | C4-3'/C2-5' | AG/GG | 26/691 | 0.0178 | 110.91/108.93 | 37.5/34.6 | 0.21 | 0.777 | 0.197 | 0.794 |
| APOC2p1229APOC4p4533/rs112698600 | C4-3'/C2-5' | CC/CT | 714/21 | 0.0140 | 109.09/107.88 | 34.6/38.8 | -0.17 | 0.840 | -0.237 | 0.772 |
| APOC2p1275APOC4p4579/rs111356234 | C4-3'/C2-5' | GA/GG | 52/687 | 0.0352 | 101.79/109.92 | 30.9/34.8 | -0.95 | 0.075 | -0.474 | 0.394 |
| APOC2p1357APOC4p4661/rs2288912 | C4-3'/C2-5' | CC/GC/GG | 50/288/411 | 0.2581 | 113.84/112.38/106.7 | 35.0/35.9/33.4 | 0.51 | 0.020 | 0.473 | 0.033 |
| APOC2p1540APOC4p4844/rs75463753 | C2-Intron 1 | AA/GA/GG | 11/128/558 | 0.1079 | 118.79/114.78/107.23 | 41.9/34.5/34.6 | 0.80 | 0.011 | 0.713 | 0.026 |
| APOC2p2486/rs9304645 | Intron 1 | AA/GA/GG | 89/364/292 | 0.3655 | 104.84/107.27/112.78 | 36.7/33.5/34.4 | -0.54 | 0.008 | -0.389 | 0.064 |
| APOC2p2935/rs11879392 | Intron 1 | CC/GC | 707/19 | 0.0135 | 108.91/116.82 | 34.6/39.3 | 0.87 | 0.317 | 0.820 | 0.338 |
| APOC2p3010/rs10419086 | Intron 1 | AA/AG/GG | 539/150/14 | 0.1253 | 109.73/109.62/109.09 | 35.5/33.3/22.0 | 0.03 | 0.921 | -0.001 | 0.997 |
| APOC2p3692/rs12721060 | Intron 1 | GT/TT | 23/633 | 0.0172 | 107.76/109.43 | 37.3/34.9 | -0.19 | 0.812 | -0.279 | 0.727 |
| APOC2p3778/rs5120 | Intron 1 | AA/AT/TT | 500/232/23 | 0.1845 | 107.58/111.87/120.21 | 34.8/34.2/30.3 | 0.60 | 0.016 | 0.493 | 0.051 |
| APOC2p3805/rs7257095 | Intron 1 | CC/CG/GG | 509/213/16 | 0.1649 | 108.23/110.64/117.89 | 34.0/36.1/35.5 | 0.35 | 0.188 | 0.229 | 0.398 |
| APOC2p3814/rs10422603 | Intron 1 | GG/GT/TT | 63/309/352 | 0.3008 | 107.1/108.14/110.88 | 39.3/34.3/34.3 | -0.30 | 0.172 | -0.242 | 0.267 |
| APOC2p3892/rs5121 | Exon 2 | CC/TC/TT | 675/50/1 | 0.0358 | 109.5/105.9/125.78 | 35.3/28.2/NA | -0.21 | 0.696 | -0.438 | 0.431 |
| APOC2p4086/rs114780592 | Intron 2 | GA/GG | 42/700 | 0.0278 | 109.37/109.04 | 37.5/34.5 | -0.04 | 0.951 | -0.051 | 0.931 |
| APOC2p4118/rs201709243 | Exon 3 | GA/GG | 1/722 | 0.0007 | 62.68/109.3 | NA/34.9 | -6.14 | 0.102 | -6.623 | 0.074 |
| APOC2p4319/rs5123 | Intron 3 | AA/GA/GG | 6/74/649 | 0.0592 | 112.84/110.41/109.17 | 21.9/34.0/34.9 | 0.19 | 0.627 | 0.279 | 0.492 |
| APOC2p4513/rs180809422 | Intron 3 | AA/AC/CC | 666/17/1 | 0.0135 | 108.77/115.46/90.54 | 34.5/35.8/NA | 0.46 | 0.572 | 0.750 | 0.407 |
| APOC2p4587/rs5126 | Exon 4 | AA/CA/CC | 636/70/1 | 0.0499 | 109.47/111.18/34.61 | 34.6/36.8/NA | -0.14 | 0.757 | -0.213 | 0.651 |
| APOC2p4754/rs7253690 | Exon 4 | AA/GA/GG | 6/79/670 | 0.0606 | 113.2/110.92/109.17 | 21.9/32.8/34.8 | 0.26 | 0.509 | 0.451 | 0.257 |
| APOC2p4853/rs150448996 | 3'flanking | DD/WD/WW | 386/284/57 | 0.2736 | 108.19/111.6/106.19 | 33.5/36.4/34.6 | 0.10 | 0.654 | -0.021 | 0.922 |
| APOC2p4973/rs199828513 | 3'flanking | WI/WW | 12/704 | 0.0082 | 96.67/109.4 | 35.9/34.3 | -1.49 | 0.171 | -1.562 | 0.164 |
| APOC2p5004/rs10421404 | 3'flanking | CC/CT/TT | 370/326/56 | 0.2908 | 111.01/107.73/106.6 | 34.1/34.1/38.2 | -0.34 | 0.115 | -0.303 | 0.168 |
| APOC2p5018/rs78403558 | 3'flanking | DD/WD/WW | 1/51/708 | 0.0352 | 125.58/115.27/108.7 | NA/36.5/34.4 | 0.77 | 0.137 | 0.721 | 0.169 |
| APOC2p5310/rs7258345 | 3'flanking | GG/GT/TT | 326/313/57 | 0.3067 | 107.52/108.38/115.79 | 35.7/33.8/27.7 | 0.37 | 0.102 | 0.278 | 0.217 |
| APOC2p5398/rs12709889 | 3'flanking | AA/GA/GG | 50/279/405 | 0.2587 | 106.53/111.31/107.46 | 32.2/36.2/33.6 | 0.19 | 0.389 | 0.098 | 0.658 |
| APOC2p5491 | 3'flanking | CC/TC | 742/1 | 0.0007 | 108.89/184.16 | 34.4/NA | 7.19 | 0.053 | 6.449 | 0.079 |
| APOC2p5512/rs12721064 | 3'flanking | CC/CT | 753/13 | 0.0083 | 109.45/100.18 | 34.5/31.2 | -1.02 | 0.325 | 0.615 | 0.592 |
| APOC2p5562 | 3'flanking | CG/GG | 24/704 | 0.0175 | 105.94/109.08 | 36.0/34.7 | -0.41 | 0.601 | -0.414 | 0.589 |
| APOC2p5586/rs73558127 | 3'flanking | GG/GT/TT | 9/127/589 | 0.1001 | 93.17/107.05/110.17 | 40.0/36.9/34.3 | -0.58 | 0.071 | -0.493 | 0.132 |
| APOC2p5815/rs10423208 | 3'flanking | AA/GA/GG | 340/323/72 | 0.3164 | 108.02/108.74/117.45 | 35.9/34.6/28.4 | 0.42 | 0.047 | 2.029 | 0.179 |
| APOC2p5922/rs10422888 | 3'flanking | AA/AG/GG | 597/99/5 | 0.0784 | 107.54/119.18/97.09 | 34.2/35.4/41.3 | 0.98 | 0.008 | 0.311 | 0.144 |
| APOC2p5965 | 3'flanking | GA/GG | 2/743 | 0.0013 | 138.11/109.1 | 75.9/34.5 | 2.76 | 0.295 | 0.914 | 0.015 |
| APOC2p6334 | 3'flanking | GA/GG | 15/746 | 0.0096 | 123.57/109.11 | 26.6/34.6 | 1.76 | 0.068 | 2.330 | 0.370 |
| MAF is the minor allele frequency; GT is genotype; GT count is the number of individuals in each genotype group; GT_SD is standard deviation of lipid traits mean in each genotype group; *Adjusted for relevant covariates, **Adjusted for *APOE*2/E*4* SNPs in addition to the covariates. APOC2p5771 is excluded due to missing data. | | | | | | | | | | |
