# Supplementary material for: Apolipoprotein E-C1-C4-C2 gene cluster region and inter-individual variation in plasma lipoprotein levels: a comprehensive genetic association study in two ethnic groups
Source: PLoS One. 2019 Mar 26;14(3):e0214060. doi: 10.1371/journal.pone.0214060 (PMC6435132; doi:10.1371/journal.pone.0214060)
Supplement: S19 Table — MAF is the minor allele frequency; GT is genotype; GT count is the number of individuals in each genotype group; GT_SD is standard deviation of lipid traits mean in each genotype group; *Adjusted for relevant covariates, **Adjusted for APOE*2/E*4 SNPs in addition to the covariates. APOC2p5771 is excluded due to missing data. (DOCX) [file pone.0214060.s019.docx]

S19 Table. Single-site association analysis results for TC in ABs

| **Variant Name/RefSNP ID** | **Location** | **Genotype** | **GT Count** | **MAF** | **Adjusted Mean of plasma TC*** | **GT_SD*** | **Beta*** | **P*** | **Adj. B** | **Adj. P** |
| --- | --- | --- | --- | --- | --- | --- | --- | --- | --- | --- |
| APOE73/rs1081101 | 5'flanking | CC/CT/TT | 652/82/5 | 0.0611 | 172.52/168.95/165.88 | 38.8/39.9/34.5 | -0.20 | 0.32826 | -0.221 | 0.292 |
| APOE173 | 5'flanking | AA/GA | 738/3 | 0.0020 | 171.99/177.58 | 39.0/13.7 | 0.41 | 0.71509 | 0.491 | 0.666 |
| APOE308/rs769445 | 5'flanking | CC/TC | 730/11 | 0.0072 | 172.04/170.26 | 39.0/36.3 | -0.06 | 0.92177 | -0.159 | 0.799 |
| APOE560/rs449647 | 5'flanking | AA/AT/TT | 298/331/103 | 0.3663 | 168.65/173.25/177.78 | 37.4/40.2/37.3 | 0.25 | 0.01773 | 0.171 | 0.134 |
| APOE618 | 5'flanking | GC/GG | 1/755 | 0.0006 | 110.73/172.23 | NA/38.7 | -3.62 | 0.06340 | -3.628 | 0.063 |
| APOE624/rs769446 | 5'flanking | TC/TT | 10/681 | 0.0077 | 149.63/172.21 | 36.3/38.7 | -1.25 | 0.04403 | -0.724 | 0.266 |
| APOE832/rs405509 | 5'flanking | GG/GT/TT | 422/267/59 | 0.2561 | 169.18/176.96/170.86 | 38.8/39.5/34.5 | 0.22 | 0.05286 | 0.152 | 0.224 |
| APOE1109/rs9282609 | Splice site | CC/TC/TT | 679/54/4 | 0.0415 | 171.7/173.68/176.55 | 38.8/39.8/31.0 | 0.12 | 0.63205 | 0.089 | 0.717 |
| APOE1163/rs440446 | Intron 1 | CC/CG/GG | 8/126/562 | 0.1004 | 170.43/175.07/172.02 | 34.5/39.6/39.0 | 0.12 | 0.47483 | 0.105 | 0.558 |
| APOE1231 | Intron 1 | GA/GG | 18/722 | 0.0125 | 163.93/172.25 | 38.1/38.9 | -0.46 | 0.33150 | -0.590 | 0.221 |
| APOE1279/rs877973 | Intron 1 | AA/CA/CC | 4/80/656 | 0.0597 | 162.54/174.33/171.91 | 25.5/43.6/38.4 | 0.04 | 0.85443 | -0.040 | 0.854 |
| APOE1539/rs184686013 | Intron 1 | AA/AG/GG | 725/11/1 | 0.0086 | 171.95/169.31/139.83 | 38.8/39.9/NA | -0.33 | 0.51724 | -0.289 | 0.585 |
| APOE2072/rs189660912 | Intron 2 | GA/GG | 12/726 | 0.0079 | 164.23/172.16 | 31.3/39.0 | -0.40 | 0.47964 | -0.344 | 0.546 |
| APOE2269/rs61357706 | Intron 2 | GA/GG | 26/714 | 0.0169 | 160.71/172.86 | 35.5/38.8 | -0.69 | 0.07713 | -0.728 | 0.068 |
| APOE2440/rs769450 | Intron 2 | AA/AG/GG | 105/304/259 | 0.3870 | 175.27/173.72/170.84 | 42.5/39.9/37.2 | 0.11 | 0.30813 | 0.106 | 0.426 |
| APOE3673/rs769453 | Intron 3 | CC/GC | 729/10 | 0.0066 | 171.92/169.91 | 38.9/37.3 | -0.07 | 0.90541 | -0.160 | 0.798 |
| APOE3937/rs429358 | Exon 4 | CC/CT/TT | 57/284/400 | 0.2656 | 171.28/175.76/169.72 | 35.1/39.9/38.7 | 0.17 | 0.13228 |  |  |
| APOE4036/rs769455 | Exon 4 | CC/TC/TT | 699/28/1 | 0.0200 | 172.65/158.44/126.63 | 38.7/34.7/NA | -0.87 | 0.01293 | -0.933 | 0.010 |
| APOE4075/rs7412 | Exon 4 | AA/GA/GG | 3/82/663 | 0.0605 | 132.98/159.61/173.83 | 26.7/35.6/39.0 | -0.82 | 0.00012 |  |  |
| APOE4569 | 3'UTR | GG/GT | 738/1 | 0.0007 | 171.93/285.01 | 38.7/NA | 5.12 | 0.00895 | 4.885 | 0.013 |
| APOE5223 | 3'flanking | CC/CG | 750/8 | 0.0051 | 172.3/153.66 | 38.8/21.6 | -0.94 | 0.17380 | -1.007 | 0.147 |
| APOE5231 | 3'flanking | GG/GT/TT | 1/36/701 | 0.0270 | 177.07/171.06/172.08 | NA/40.3/38.9 | -0.03 | 0.92075 | -0.115 | 0.722 |
| rs439401 | Intergenic | CC/CT/TT | 579/131/14 | 0.1092 | 171.84/173.4/170.9 | 39.4/38.1/29.0 | 0.07 | 0.66212 | 0.036 | 0.828 |
| APOC1rs445925 | Intergenic | AA/GA/GG | 66/309/362 | 0.2990 | 171.23/172.47/172.52 | 39.6/36.7/39.7 | -0.01 | 0.89831 | 0.272 | 0.208 |
| APOC1p720ins4/rs11568822 | 5'flanking | II/WI/WW | 58/277/392 | 0.2737 | 164.47/171.23/173.59 | 38.6/39.4/38.8 | -0.20 | 0.07407 | -0.083 | 0.508 |
| APOC1p894/rs190454394 | 5'flanking | CC/CT | 732/3 | 0.0020 | 171.74/205.36 | 38.8/53.9 | 1.71 | 0.13168 | 1.679 | 0.140 |
| APOC1p1166/rs72654452 | Intron 1 | CC/CT/TT | 708/41/2 | 0.0308 | 171.47/181.29/148.14 | 38.3/43.5/17.7 | 0.31 | 0.27827 | 0.259 | 0.386 |
| APOC1p1331/rs10408994 | Intron 2 | AG/GG | 97/632 | 0.0666 | 177.72/171.39 | 38.9/39.1 | 0.34 | 0.11160 | 0.370 | 0.095 |
| APOC1p1526/rs5114 | Intron 2 | CC/CT/TT | 649/77/4 | 0.0579 | 171.74/176.03/162.64 | 38.4/44.1/25.5 | 0.12 | 0.58756 | 0.036 | 0.871 |
| APOC1p1642 | Intron 2 | CC/CT | 735/15 | 0.0103 | 172.35/161.09 | 38.7/40.2 | -0.64 | 0.21318 | -0.778 | 0.137 |
| APOC1p1684/rs12709881 | Intron 2 | AA/GA/GG | 8/128/611 | 0.0973 | 156.49/171.71/172.5 | 39.8/41.6/38.2 | -0.16 | 0.36396 | -0.235 | 0.181 |
| APOC1p3358 | Intron 3 | AA/GA | 700/3 | 0.0021 | 171.94/176.29 | 39.2/26.1 | 0.34 | 0.76322 | 0.594 | 0.604 |
| APOC1p3423/rs389261 | Intron 3 | AA/GA/GG | 77/318/312 | 0.3310 | 173.68/173.27/170.57 | 35.1/39.5/39.9 | 0.12 | 0.29576 | 0.043 | 0.724 |
| APOC1p3573/rs10424339 | Intron 3 | AA/GA/GG | 16/171/536 | 0.1396 | 164.72/174.52/171.68 | 36.4/36.3/39.9 | 0.07 | 0.63708 | 0.070 | 0.650 |
| APOC1p5006/rs112528434 | Intron 3 | GG/GT/TT | 568/100/6 | 0.0850 | 173.47/174.06/156.23 | 38.6/43.1/39.0 | -0.10 | 0.60525 | -0.177 | 0.371 |
| APOC1p5053/rs12721052 | Intron 3 | DD/WD/WW | 40/246/460 | 0.2200 | 178.43/172.75/171 | 44.3/40.3/37.1 | 0.13 | 0.27278 | 0.121 | 0.344 |
| APOC1p5667/rs12721054 | 3'UTR | AA/GA/GG | 504/161/16 | 0.1446 | 173.87/170.98/153.32 | 38.8/41.5/31.7 | -0.30 | 0.04743 | -0.262 | 0.094 |
| APOC1p5926/rs56131196 | 3'flanking | AA/AG/GG | 21/212/490 | 0.1745 | 170.86/171.58/172.33 | 41.2/39.1/38.7 | -0.05 | 0.72955 | -0.086 | 0.542 |
| rs4803770 | Intergenic | CC/GC/GG | 390/274/56 | 0.2695 | 171.64/170.95/185.27 | 38.0/39.2/42.8 | 0.18 | 0.11016 | 0.152 | 0.211 |
| HCR1p424/rs117664574 | HCR1 | AG/GG | 11/725 | 0.0073 | 174.18/171.92 | 39.3/38.9 | 0.12 | 0.83438 | 0.083 | 0.890 |
| HCR1p575/rs157599 | HCR1 | AA/AG/GG | 277/307/90 | 0.3595 | 171.82/172.01/175.29 | 38.9/39.5/34.8 | 0.08 | 0.46903 | 0.002 | 0.984 |
| rs5112 | *APOC1P1* | CC/GC/GG | 199/330/163 | 0.4797 | 171.13/173.37/177.09 | 39.3/38.4/37.2 | 0.16 | 0.10913 | 0.161 | 0.142 |
| rs7259004 | *APOC1P1* | CC/CG/GG | 73/284/366 | 0.3020 | 176.55/171.72/171.5 | 37.8/39.2/39.1 | 0.09 | 0.39758 | 0.157 | 0.178 |
| HCR2p188/rs35136575 | HCR2 | CC/GC/GG | 526/174/25 | 0.1546 | 171.86/171.73/172.93 | 39.1/38.8/40.7 | 0.01 | 0.91840 | 0.031 | 0.825 |
| HCR2p286 | HCR2 | AA/AG/GG | 3/63/667 | 0.0457 | 153.32/180.43/171.37 | 28.6/41.5/38.7 | 0.31 | 0.18468 | 0.288 | 0.233 |
| HCR2p523/rs118004808 | HCR2 | CC/TC | 734/4 | 0.0026 | 171.93/194.43 | 38.8/44.3 | 1.17 | 0.23621 | 1.104 | 0.263 |
| APOC4p368 | 5’ flanking | TC/TT | 3/741 | 0.0019 | 184.54/171.95 | 37.9/38.9 | 0.71 | 0.52903 | 0.645 | 0.570 |
| APOC4p637/rs113814026 | 5’ flanking | GG/GT/TT | 678/67/1 | 0.0452 | 172.06/172.49/85.95 | 38.6/38.1/NA | -0.13 | 0.58316 | -0.106 | 0.675 |
| APOC4p757/rs12721105 | 5’ flanking | GG/GT/TT | 701/52/2 | 0.0376 | 171.91/173.9/184.62 | 38.6/40.0/43.8 | 0.13 | 0.61917 | 0.059 | 0.824 |
| APOC4p1088 | Intron 1 | GT/TT | 2/728 | 0.0013 | 146.52/172.31 | 48.8/38.9 | -1.51 | 0.27674 | -1.487 | 0.283 |
| APOC4p1130 | Intron 1 | CT/TT | 1/736 | 0.0007 | 198.57/171.99 | NA/38.9 | 1.50 | 0.44346 | 1.457 | 0.457 |
| APOC4p1192/rs113745034 | Intron 1 | GA/GG | 18/690 | 0.0124 | 166.8/172.17 | 44.8/38.9 | -0.31 | 0.50557 | -0.262 | 0.578 |
| APOC4p1325del3 | Intron 1 | WD/WW | 36/698 | 0.0245 | 176.47/171.96 | 35.7/39.0 | 0.28 | 0.40992 | 0.257 | 0.451 |
| APOC4p1430ins | Intron 1 | II/WI/WW | 1/44/616 | 0.0341 | 144.75/178.76/170.96 | NA/35.8/39.2 | 0.35 | 0.22899 | 0.368 | 0.212 |
| APOC4p2099/rs111339708 | Intron 1 | GG/GT | 728/21 | 0.0141 | 172.27/164.72 | 38.8/42.9 | -0.42 | 0.33673 | -0.270 | 0.555 |
| APOC4p2467/rs115225947 | Intron 1 | GA/GG | 20/729 | 0.0141 | 177.8/171.89 | 42.9/38.7 | 0.30 | 0.49226 | 0.330 | 0.457 |
| APOC4p2559/rs5155 | Intron 1 | CC/CT/TT | 608/133/6 | 0.0986 | 172.28/170.33/144.83 | 38.3/39.3/30.3 | -0.22 | 0.20901 | -0.156 | 0.382 |
| APOC4p2607/rs5156 | Intron 1 | AG/GG | 18/697 | 0.0129 | 163.6/172.41 | 43.8/39.0 | -0.49 | 0.29810 | -0.381 | 0.432 |
| APOC4p2623/rs5157 | Intron 1 | CC/CT/TT | 503/215/20 | 0.1723 | 170.66/175.33/164.71 | 39.3/38.4/35.3 | 0.13 | 0.32874 | 0.149 | 0.284 |
| APOC4p2640/rs5158 | Intron 1 | CC/CT | 714/32 | 0.0213 | 171.84/171.36 | 38.8/33.7 | 0.01 | 0.97117 | -0.004 | 0.991 |
| APOC4p2678/rs148564866 | Intron 1 | GC/GG | 12/719 | 0.0086 | 165.62/172.23 | 32.1/39.1 | -0.31 | 0.58397 | -0.501 | 0.383 |
| APOC4p2767/rs127721107 | Intron 1 | GG/GT | 691/37 | 0.0254 | 171.99/175.77 | 38.7/44.1 | 0.16 | 0.62791 | 0.145 | 0.666 |
| APOC4p3348 | Intron 1 | AG/GG | 1/734 | 0.0007 | 183.75/172.05 | NA/39.0 | 0.74 | 0.70666 | 1.567 | 0.427 |
| APOC2p75APOC4p3380/rs12721104 | C4-Intron 1 | AA/GA/GG | 13/174/555 | 0.1368 | 165.15/172.76/171.78 | 39.0/40.5/38.2 | -0.01 | 0.92441 | 0.034 | 0.826 |
| APOC2p194APOC4p3498/rs1132899 | C4-Exon 2 | CC/CT/TT | 429/279/40 | 0.2368 | 170.08/175.69/164.97 | 39.0/38.7/34.4 | 0.11 | 0.35464 | 0.113 | 0.352 |
| APOC2p228/rs5164 | C4-Exon 2 | AG/GA/GG | 9/1/723 | 0.0066 | 164.83/154.22/172.26 | 34.6/NA/39.1 | -0.43 | 0.49532 | -0.428 | 0.520 |
| APOC2p288APOC4p3592/rs12691090 | C4-Exon 2 | CC/CT | 692/40 | 0.0272 | 172.07/173.5 | 38.7/45.2 | 0.02 | 0.94191 | 0.069 | 0.831 |
| APOC2p396APOC4p3700 | C4-Intron 2 | GA/GG | 1/711 | 0.0007 | 144.11/172.63 | NA/39.2 | -1.40 | 0.48018 | -1.491 | 0.450 |
| APOC2p488APOC4p3792/rs5165 | C4-Intron 2 | GA/GG | 22/707 | 0.0146 | 169.39/172.03 | 26.1/39.4 | -0.07 | 0.86634 | -0.056 | 0.895 |
| APOC2p623APOC4p3927/rs5167 | C4-Exon 3 | GG/GT/TT | 164/362/220 | 0.4594 | 168.86/171.92/174.54 | 38.9/39.2/37.7 | -0.16 | 0.10575 | -0.167 | 0.105 |
| APOC2p665APOC4p3969/rs138548797 | C4-Exon 3 | AA/CA | 721/13 | 0.0086 | 171.82/172.47 | 38.8/45.3 | -0.004 | 0.99377 | 0.202 | 0.723 |
| APOC2p708APOC4p4012 | C4-Exon 3 | GA/GG | 1/731 | 0.0007 | 145.97/172.21 | NA/38.9 | -1.34 | 0.49361 | -1.398 | 0.476 |
| APOC2p853APOC4p4157/rs10425530 | C4-3' UTR | AA/GA/GG | 7/150/582 | 0.1100 | 154.39/172.94/172.12 | 19.7/41.6/38.2 | -0.06 | 0.70512 | -0.064 | 0.703 |
| APOC2p1042APOC4p4346/rs12709885 | C4-3'/C2-5' | AA/TA/TT | 712/23/1 | 0.0178 | 172.53/159.97/173.87 | 39.1/31.2/NA | -0.55 | 0.15479 | -0.374 | 0.353 |
| APOC2p1187APOC4p4491/rs111782345 | C4-3'/C2-5' | AG/GG | 25/686 | 0.0178 | 175.33/171.82 | 43.6/38.8 | 0.17 | 0.67391 | 0.184 | 0.655 |
| APOC2p1229APOC4p4533/rs112698600 | C4-3'/C2-5' | CC/CT | 707/20 | 0.0140 | 171.91/173.08 | 38.9/45.0 | 0.04 | 0.93279 | 0.007 | 0.987 |
| APOC2p1275APOC4p4579/rs111356234 | C4-3'/C2-5' | GA/GG | 50/681 | 0.0352 | 163.72/172.87 | 33.9/39.2 | -0.48 | 0.09537 | -0.259 | 0.394 |
| APOC2p1357APOC4p4661/rs2288912 | C4-3'/C2-5' | CC/GC/GG | 49/285/407 | 0.2581 | 175.4/176.37/169.04 | 37.4/39.7/38.2 | 0.28 | 0.01532 | 0.262 | 0.027 |
| APOC2p1540APOC4p4844/rs75463753 | C2-Intron 1 | AA/GA/GG | 11/129/552 | 0.1079 | 185.45/177.57/169.95 | 36.9/38.3/39.1 | 0.42 | 0.01106 | 0.394 | 0.020 |
| APOC2p2486/rs9304645 | Intron 1 | AA/GA/GG | 87/363/287 | 0.3655 | 169.03/170.07/174.92 | 40.7/37.6/39.2 | -0.20 | 0.07368 | -0.122 | 0.276 |
| APOC2p2935/rs11879392 | Intron 1 | CC/GC | 698/20 | 0.0135 | 171.98/177.14 | 39.0/41.7 | 0.27 | 0.53988 | 0.244 | 0.586 |
| APOC2p3010/rs10419086 | Intron 1 | AA/AG/GG | 537/145/13 | 0.1253 | 172.94/171.72/170.36 | 39.8/38.0/28.3 | -0.05 | 0.74391 | -0.068 | 0.674 |
| APOC2p3692/rs12721060 | Intron 1 | GT/TT | 22/627 | 0.0172 | 171.96/172.46 | 43.3/39.3 | -0.04 | 0.92973 | -0.074 | 0.866 |
| APOC2p3778/rs5120 | Intron 1 | AA/AT/TT | 496/227/24 | 0.1845 | 170.12/175.39/183.12 | 39.3/38.1/30.7 | 0.33 | 0.01287 | 0.281 | 0.037 |
| APOC2p3805/rs7257095 | Intron 1 | CC/CG/GG | 507/207/16 | 0.1649 | 171.27/172.82/181.24 | 38.3/40.8/33.6 | 0.13 | 0.35514 | 0.089 | 0.540 |
| APOC2p3814/rs10422603 | Intron 1 | GG/GT/TT | 61/310/345 | 0.3008 | 171.23/171.5/173.16 | 45.9/37.8/38.8 | -0.08 | 0.47136 | -0.056 | 0.635 |
| APOC2p3892/rs5121 | Exon 2 | CC/TC/TT | 669/49/1 | 0.0358 | 172.5/167.84/201.55 | 39.7/28.7/NA | -0.11 | 0.69795 | -0.237 | 0.428 |
| APOC2p4086/rs114780592 | Intron 2 | GA/GG | 41/693 | 0.0278 | 172.27/171.92 | 43.0/38.7 | -0.02 | 0.95061 | -0.038 | 0.905 |
| APOC2p4118/rs201709243 | Exon 3 | GA/GG | 1/716 | 0.0007 | 179.84/172.13 | NA/39.1 | 0.52 | 0.79263 | 0.345 | 0.861 |
| APOC2p4319/rs5123 | Intron 3 | AA/GA/GG | 6/76/639 | 0.0592 | 176.63/174.09/171.69 | 17.9/38.9/39.2 | 0.15 | 0.47956 | 0.218 | 0.311 |
| APOC2p4513/rs180809422 | Intron 3 | AA/AC/CC | 660/16/1 | 0.0135 | 171.48/182.63/147.07 | 38.7/42.8/NA | 0.34 | 0.43800 | 0.556 | 0.264 |
| APOC2p4587/rs5126 | Exon 4 | AA/CA/CC | 630/69/1 | 0.0499 | 172.47/174.05/87.92 | 39.1/39.1/NA | -0.07 | 0.77098 | -0.106 | 0.674 |
| APOC2p4754/rs7253690 | Exon 4 | AA/GA/GG | 6/81/660 | 0.0606 | 176.79/176.46/171.75 | 17.9/37.8/38.9 | 0.25 | 0.22124 | 0.335 | 0.110 |
| APOC2p4853/rs150448996 | 3'flanking | DD/WD/WW | 380/283/56 | 0.2736 | 171.97/173.3/169.32 | 37.4/40.9/41.4 | -0.02 | 0.84447 | -0.078 | 0.506 |
| APOC2p4973/rs199828513 | 3'flanking | WI/WW | 12/695 | 0.0082 | 158.47/172.45 | 42.8/38.5 | -0.79 | 0.16638 | -0.843 | 0.159 |
| APOC2p5004/rs10421404 | 3'flanking | CC/CT/TT | 364/325/54 | 0.2908 | 173.36/170.73/171 | 38.5/37.8/44.3 | -0.11 | 0.33714 | -0.095 | 0.422 |
| APOC2p5018/rs78403558 | 3'flanking | DD/WD/WW | 1/52/699 | 0.0352 | 201.27/176.34/171.67 | NA/39.6/38.7 | 0.30 | 0.27612 | 0.285 | 0.305 |
| APOC2p5310/rs7258345 | 3'flanking | GG/GT/TT | 324/306/56 | 0.3067 | 170.54/172.14/177.55 | 40.3/38.2/29.6 | 0.17 | 0.14969 | 0.127 | 0.294 |
| APOC2p5398/rs12709889 | 3'flanking | AA/GA/GG | 49/278/399 | 0.2587 | 169.1/173.21/171.05 | 37.8/40.8/37.6 | 0.02 | 0.84406 | -0.014 | 0.908 |
| APOC2p5491 | 3'flanking | CC/TC | 734/1 | 0.0007 | 171.71/265.05 | 38.6/NA | 4.21 | 0.03146 | 3.905 | 0.046 |
| APOC2p5512/rs12721064 | 3'flanking | CC/CT | 746/12 | 0.0083 | 172.17/170.04 | 38.7/35.3 | -0.10 | 0.86343 | 0.714 | 0.262 |
| APOC2p5562 | 3'flanking | CG/GG | 26/696 | 0.0175 | 168.35/171.94 | 38.4/39.0 | -0.21 | 0.59488 | -0.200 | 0.612 |
| APOC2p5586/rs73558127 | 3'flanking | GG/GT/TT | 9/126/583 | 0.1001 | 154.43/170.57/172.77 | 45.8/40.7/38.7 | -0.23 | 0.17781 | -0.187 | 0.286 |
| APOC2p5815/rs10423208 | 3'flanking | AA/GA/GG | 341/315/71 | 0.3164 | 170.83/172.31/178 | 40.4/39.2/30.3 | 0.18 | 0.11697 | 0.684 | 0.362 |
| APOC2p5922/rs10422888 | 3'flanking | AA/AG/GG | 590/101/5 | 0.0784 | 170.44/182.24/164.13 | 38.3/40.1/51.6 | 0.48 | 0.01291 | 0.127 | 0.264 |
| APOC2p5965 | 3'flanking | GA/GG | 2/735 | 0.0013 | 208.01/171.97 | 93.3/38.7 | 1.58 | 0.25416 | 0.456 | 0.021 |
| APOC2p6334 | 3'flanking | GA/GG | 15/738 | 0.0096 | 188.71/171.92 | 31.0/38.7 | 0.94 | 0.06281 | 1.430 | 0.303 |
| MAF is the minor allele frequency; GT is genotype; GT count is the number of individuals in each genotype group; GT_SD is standard deviation of lipid traits mean in each genotype group; *Adjusted for relevant covariates, **Adjusted for *APOE*2/E*4* SNPs in addition to the covariates. APOC2p5771 is excluded due to missing data. | | | | | | | | | | |
