# Supplementary material for: Apolipoprotein E-C1-C4-C2 gene cluster region and inter-individual variation in plasma lipoprotein levels: a comprehensive genetic association study in two ethnic groups
Source: PLoS One. 2019 Mar 26;14(3):e0214060. doi: 10.1371/journal.pone.0214060 (PMC6435132; doi:10.1371/journal.pone.0214060)
Supplement: S20 Table — MAF is the minor allele frequency; GT is genotype; GT count is the number of individuals in each genotype group; GT_SD is standard deviation of lipid traits mean in each genotype group; *Adjusted for relevant covariates, **Adjusted for APOE*2/E*4 SNPs in addition to the covariates. APOC2p5771 is excluded due to missing data. (DOCX) [file pone.0214060.s020.docx]

S20 Table. Single-site association analysis results for apoB in ABs

| **Variant Name/RefSNP ID** | **Location** | **Genotype** | **GT Count** |  | **MAF** | **Adjusted Mean of plasma apoB*** | **GT_SD*** | **Beta*** | **P*** | **Adj. B** | **Adj. P** |
| --- | --- | --- | --- | --- | --- | --- | --- | --- | --- | --- | --- |
| APOE73/rs1081101 | 5'flanking | CC/CT/TT | 658/82/5 |  | 0.0611 | 66.45/67.4/73.43 | 21.5/24.7/8.0 | 0.65 | 0.54877 | 0.464 | 0.674 |
| APOE173 | 5'flanking | AA/GA | 744/3 |  | 0.0020 | 66.5/72.76 | 21.8/14.2 | 3.20 | 0.59226 | 3.416 | 0.569 |
| APOE308/rs769445 | 5'flanking | CC/TC | 736/11 |  | 0.0072 | 66.47/70.18 | 21.8/21.5 | 1.87 | 0.55135 | 1.860 | 0.572 |
| APOE560/rs449647 | 5'flanking | AA/AT/TT | 302/335/105 |  | 0.3663 | 65.41/66.78/69.26 | 22.9/21.9/19.0 | 0.92 | 0.09509 | 1.056 | 0.077 |
| APOE618 | 5'flanking | GC/GG | 1/766 |  | 0.0006 | 74.68/66.72 | NA/21.8 | 4.15 | 0.68762 | 3.903 | 0.705 |
| APOE624/rs769446 | 5'flanking | TC/TT | 10/693 |  | 0.0077 | 57.76/66.77 | 13.5/21.9 | -4.19 | 0.20373 | -2.471 | 0.471 |
| APOE832/rs405509 | 5'flanking | GG/GT/TT | 429/269/61 |  | 0.2561 | 65.72/68.69/66.11 | 21.6/22.1/21.5 | 0.66 | 0.26113 | 0.679 | 0.298 |
| APOE1109/rs9282609 | Splice site | CC/TC/TT | 684/55/4 |  | 0.0415 | 66.07/71.5/56.8 | 21.4/24.4/28.8 | 1.38 | 0.27683 | 1.223 | 0.340 |
| APOE1163/rs440446 | Intron 1 | CC/CG/GG | 8/126/568 |  | 0.1004 | 73.38/71.01/66.01 | 15.1/22.5/21.7 | 2.30 | 0.01093 | 1.940 | 0.037 |
| APOE1231 | Intron 1 | GA/GG | 19/727 |  | 0.0125 | 62.87/66.6 | 18.2/21.9 | -1.71 | 0.47484 | -1.630 | 0.507 |
| APOE1279/rs877973 | Intron 1 | AA/CA/CC | 4/82/660 |  | 0.0597 | 51.79/67.05/66.53 | 6.6/24.2/21.6 | -0.45 | 0.68467 | -0.983 | 0.390 |
| APOE1539/rs184686013 | Intron 1 | AA/AG/GG | 732/11/1 |  | 0.0086 | 66.43/70.12/48.6 | 21.8/20.5/NA | 0.23 | 0.93296 | 0.316 | 0.909 |
| APOE2072/rs189660912 | Intron 2 | GA/GG | 12/732 |  | 0.0079 | 67.45/66.55 | 10.5/22.0 | 0.70 | 0.81486 | 0.721 | 0.810 |
| APOE2269/rs61357706 | Intron 2 | GA/GG | 25/726 |  | 0.0169 | 61.69/66.98 | 19.9/21.8 | -2.56 | 0.22005 | -2.822 | 0.186 |
| APOE2440/rs769450 | Intron 2 | AA/AG/GG | 109/302/263 |  | 0.3870 | 65.5/67.83/67.87 | 22.4/21.8/21.0 | -0.50 | 0.37248 | -0.944 | 0.157 |
| APOE3673/rs769453 | Intron 3 | CC/GC | 735/10 |  | 0.0066 | 66.4/70.47 | 21.7/22.5 | 2.02 | 0.53860 | 1.810 | 0.581 |
| APOE3937/rs429358 | Exon 4 | CC/CT/TT | 56/288/408 |  | 0.2656 | 65.25/67.46/66.46 | 20.4/22.6/21.6 | 0.05 | 0.93710 |  |  |
| APOE4036/rs769455 | Exon 4 | CC/TC/TT | 706/27/1 |  | 0.0200 | 66.89/60.39/48.19 | 21.7/22.7/NA | -3.45 | 0.06644 | -3.717 | 0.052 |
| APOE4075/rs7412 | Exon 4 | AA/GA/GG | 3/84/672 |  | 0.0605 | 45.96/63.02/67.16 | 27.7/18.2/22.2 | -2.35 | 0.03564 |  |  |
| APOE4569 | 3'UTR | GG/GT | 744/1 |  | 0.0007 | 66.46/96.65 | 21.8/NA | 14.25 | 0.16840 | 13.769 | 0.182 |
| APOE5223 | 3'flanking | CC/CG | 762/7 |  | 0.0051 | 66.75/61.28 | 21.9/17.7 | -2.54 | 0.51655 | -2.842 | 0.468 |
| APOE5231 | 3'flanking | GG/GT/TT | 2/37/705 |  | 0.0270 | 50.22/66.27/66.55 | 17.5/21.6/21.9 | -0.82 | 0.60359 | -0.857 | 0.593 |
| rs439401 | Intergenic | CC/CT/TT | 584/131/15 |  | 0.1092 | 65.65/69.73/66.92 | 21.8/22.7/17.8 | 1.42 | 0.08620 | 0.995 | 0.253 |
| APOC1rs445925 | Intergenic | AA/GA/GG | 67/311/370 |  | 0.2990 | 67.55/65.84/67.5 | 23.1/21.2/22.3 | -0.31 | 0.59161 | 1.115 | 0.323 |
| APOC1p720ins4/rs11568822 | 5'flanking | II/WI/WW | 62/277/394 |  | 0.2737 | 62.61/65.77/67.67 | 21.4/21.7/22.1 | -1.10 | 0.06438 | -0.798 | 0.216 |
| APOC1p894/rs190454394 | 5'flanking | CC/CT | 738/3 |  | 0.0020 | 66.39/83.3 | 21.8/18.1 | 8.19 | 0.17048 | 7.900 | 0.186 |
| APOC1p1166/rs72654452 | Intron 1 | CC/CT/TT | 717/43/2 |  | 0.0308 | 66.43/69.71/58.03 | 21.8/23.5/6.5 | 0.98 | 0.51091 | 0.552 | 0.722 |
| APOC1p1331/rs10408994 | Intron 2 | AG/GG | 95/640 |  | 0.0666 | 65.22/66.91 | 20.9/22.0 | -0.83 | 0.46727 | -1.057 | 0.368 |
| APOC1p1526/rs5114 | Intron 2 | CC/CT/TT | 653/79/4 |  | 0.0579 | 66.45/67.64/51.76 | 21.6/24.8/6.6 | -0.22 | 0.84865 | -0.732 | 0.528 |
| APOC1p1642 | Intron 2 | CC/CT | 745/16 |  | 0.0103 | 66.75/62.78 | 21.9/19.4 | -1.86 | 0.47702 | -1.832 | 0.491 |
| APOC1p1684/rs12709881 | Intron 2 | AA/GA/GG | 9/132/617 |  | 0.0973 | 49.71/65.76/67 | 9.7/23.1/21.7 | -1.48 | 0.09465 | -1.894 | 0.036 |
| APOC1p3358 | Intron 3 | AA/GA | 703/3 |  | 0.0021 | 66.41/59 | 22.0/31.7 | -3.96 | 0.50955 | -3.705 | 0.539 |
| APOC1p3423/rs389261 | Intron 3 | AA/GA/GG | 77/315/320 |  | 0.3310 | 65.53/67.06/66.54 | 20.1/21.6/22.6 | -0.04 | 0.94719 | -0.037 | 0.954 |
| APOC1p3573/rs10424339 | Intron 3 | AA/GA/GG | 15/171/543 |  | 0.1396 | 63.34/65.58/67.09 | 14.8/19.7/22.7 | -0.71 | 0.36467 | -0.827 | 0.315 |
| APOC1p5006/rs112528434 | Intron 3 | GG/GT/TT | 573/105/7 |  | 0.0850 | 67.71/67.25/49.23 | 21.7/22.7/9.0 | -1.22 | 0.20990 | -1.609 | 0.107 |
| APOC1p5053/rs12721052 | Intron 3 | DD/WD/WW | 43/248/467 |  | 0.2200 | 70.33/66.93/66.24 | 22.6/21.8/21.9 | 0.66 | 0.29256 | 0.639 | 0.337 |
| APOC1p5667/rs12721054 | 3'UTR | AA/GA/GG | 505/163/19 |  | 0.1446 | 67.33/67.34/55.54 | 21.9/21.2/19.3 | -1.01 | 0.18808 | -0.976 | 0.214 |
| APOC1p5926/rs56131196 | 3'flanking | AA/AG/GG | 21/211/494 |  | 0.1745 | 58.05/66.99/66.81 | 26.0/21.8/21.8 | -0.70 | 0.33774 | -0.682 | 0.358 |
| rs4803770 | Intergenic | CC/GC/GG | 392/276/58 |  | 0.2695 | 65.83/66.43/71.57 | 21.7/21.6/24.4 | 0.90 | 0.13554 | 0.717 | 0.263 |
| HCR1p424/rs117664574 | HCR1 | AG/GG | 11/731 |  | 0.0073 | 71.83/66.46 | 37.1/21.6 | 2.07 | 0.51060 | 1.721 | 0.585 |
| HCR1p575/rs157599 | HCR1 | AA/AG/GG | 285/309/92 |  | 0.3595 | 66.91/66.92/68.27 | 22.7/20.7/22.2 | 0.27 | 0.64249 | 0.017 | 0.978 |
| rs5112 | *APOC1P1* | CC/GC/GG | 199/330/171 |  | 0.4797 | 66.7/67.09/68.92 | 22.6/21.7/21.6 | 0.54 | 0.31703 | 0.258 | 0.656 |
| rs7259004 | *APOC1P1* | CC/CG/GG | 76/291/362 |  | 0.3020 | 67.71/66.77/66.22 | 19.5/21.6/22.6 | 0.37 | 0.51449 | 0.161 | 0.791 |
| HCR2p188/rs35136575 | HCR2 | CC/GC/GG | 529/177/25 |  | 0.1546 | 67.35/63.27/73.02 | 22.0/21.2/22.5 | -0.61 | 0.40150 | -0.758 | 0.303 |
| HCR2p286 | HCR2 | AA/AG/GG | 3/63/673 |  | 0.0457 | 39.84/72.35/66.09 | 25.4/23.5/21.6 | 1.37 | 0.27692 | 1.318 | 0.301 |
| HCR2p523/rs118004808 | HCR2 | CC/TC | 740/4 |  | 0.0026 | 66.44/80.18 | 21.9/14.8 | 6.71 | 0.19588 | 6.346 | 0.222 |
| APOC4p368 | 5’ flanking | TC/TT | 3/754 |  | 0.0019 | 63.48/66.95 | 3.8/21.7 | -1.39 | 0.81479 | -1.721 | 0.771 |
| APOC4p637/rs113814026 | 5’ flanking | GG/GT/TT | 689/67/1 |  | 0.0452 | 66.4/69.81/52.26 | 21.5/23.4/NA | 1.34 | 0.29549 | 1.992 | 0.131 |
| APOC4p757/rs12721105 | 5’ flanking | GG/GT/TT | 710/54/2 |  | 0.0376 | 66.65/67.05/74.54 | 21.9/21.2/2.9 | 0.45 | 0.74360 | 0.262 | 0.850 |
| APOC4p1088 | Intron 1 | GT/TT | 2/734 |  | 0.0013 | 61.84/66.56 | 12.6/21.9 | -2.07 | 0.77750 | -2.487 | 0.734 |
| APOC4p1130 | Intron 1 | CT/TT | 1/742 |  | 0.0007 | 25.33/66.55 | NA/21.8 | -21.03 | 0.04207 | -21.470 | 0.038 |
| APOC4p1192/rs113745034 | Intron 1 | GA/GG | 17/694 |  | 0.0124 | 72.18/66.29 | 25.1/21.8 | 2.83 | 0.26681 | 2.949 | 0.248 |
| APOC4p1325del3 | Intron 1 | WD/WW | 36/704 |  | 0.0245 | 69.67/66.4 | 18.1/22.0 | 1.71 | 0.33255 | 1.701 | 0.342 |
| APOC4p1430ins | Intron 1 | II/WI/WW | 1/44/627 |  | 0.0341 | 74.06/68.25/65.56 | NA/21.6/22.1 | 1.43 | 0.35841 | 1.437 | 0.358 |
| APOC4p2099/rs111339708 | Intron 1 | GG/GT | 738/22 |  | 0.0141 | 66.63/69.07 | 21.9/23.8 | 1.19 | 0.59549 | 1.849 | 0.432 |
| APOC4p2467/rs115225947 | Intron 1 | GA/GG | 21/739 |  | 0.0141 | 65.91/66.7 | 24.1/21.9 | -0.42 | 0.85407 | -0.433 | 0.850 |
| APOC4p2559/rs5155 | Intron 1 | CC/CT/TT | 616/135/7 |  | 0.0986 | 66.99/65/61.61 | 22.2/20.6/13.4 | -0.98 | 0.27438 | -0.901 | 0.332 |
| APOC4p2607/rs5156 | Intron 1 | AG/GG | 19/700 |  | 0.0129 | 69.78/66.44 | 24.9/21.9 | 1.60 | 0.50775 | 2.108 | 0.397 |
| APOC4p2623/rs5157 | Intron 1 | CC/CT/TT | 512/216/21 |  | 0.1723 | 65.29/70.07/63.46 | 21.7/22.7/15.2 | 1.43 | 0.04428 | 1.781 | 0.014 |
| APOC4p2640/rs5158 | Intron 1 | CC/CT | 726/31 |  | 0.0213 | 66.45/69.11 | 21.9/17.3 | 1.44 | 0.44596 | 1.505 | 0.433 |
| APOC4p2678/rs148564866 | Intron 1 | GC/GG | 13/724 |  | 0.0086 | 68.5/66.46 | 27.4/21.7 | 0.87 | 0.76308 | 0.572 | 0.844 |
| APOC4p2767/rs127721107 | Intron 1 | GG/GT | 696/37 |  | 0.0254 | 66.3/72.91 | 21.7/25.5 | 3.15 | 0.07284 | 3.004 | 0.088 |
| APOC4p3348 | Intron 1 | AG/GG | 1/740 |  | 0.0007 | 46/66.52 | NA/21.9 | -9.98 | 0.33555 | -8.345 | 0.422 |
| APOC2p75APOC4p3380/rs12721104 | C4-Intron 1 | AA/GA/GG | 14/178/561 |  | 0.1368 | 66.87/66.3/66.63 | 14.1/21.8/22.2 | -0.07 | 0.92847 | -0.189 | 0.814 |
| APOC2p194APOC4p3498/rs1132899 | C4-Exon 2 | CC/CT/TT | 440/279/40 |  | 0.2368 | 65.25/68.96/64.74 | 21.6/22.9/15.7 | 0.95 | 0.13191 | 1.183 | 0.065 |
| APOC2p228/rs5164 | C4-Exon 2 | AG/GA/GG | 9/1/728 |  | 0.0066 | 71.19/78.12/66.48 | 29.3/NA/21.8 | 2.49 | 0.45010 | -0.683 | 0.844 |
| APOC2p288APOC4p3592/rs12691090 | C4-Exon 2 | CC/CT | 697/40 |  | 0.0272 | 66.31/70.55 | 21.7/25.9 | 1.96 | 0.24561 | 1.963 | 0.247 |
| APOC2p396APOC4p3700 | C4-Intron 2 | GA/GG | 1/717 |  | 0.0007 | 47.55/66.81 | NA/21.9 | -9.04 | 0.38386 | -9.555 | 0.357 |
| APOC2p488APOC4p3792/rs5165 | C4-Intron 2 | GA/GG | 22/713 |  | 0.0146 | 64.63/66.62 | 17.4/22.1 | -0.83 | 0.71058 | -0.653 | 0.772 |
| APOC2p623APOC4p3927/rs5167 | C4-Exon 3 | GG/GT/TT | 164/369/224 |  | 0.4594 | 63.91/68.02/66.36 | 22.8/22.5/20.1 | -0.54 | 0.31017 | -0.545 | 0.313 |
| APOC2p665APOC4p3969/rs138548797 | C4-Exon 3 | AA/CA | 727/13 |  | 0.0086 | 66.32/75.01 | 21.8/26.4 | 4.09 | 0.15807 | 4.942 | 0.101 |
| APOC2p708APOC4p4012 | C4-Exon 3 | GA/GG | 1/737 |  | 0.0007 | 79.3/66.53 | NA/21.9 | 6.46 | 0.53306 | 6.181 | 0.550 |
| APOC2p853APOC4p4157/rs10425530 | C4-3' UTR | AA/GA/GG | 7/152/591 |  | 0.1100 | 58.99/66.53/66.84 | 17.7/24.4/21.2 | -0.53 | 0.53990 | -0.746 | 0.393 |
| APOC2p1042APOC4p4346/rs12709885 | C4-3'/C2-5' | AA/TA/TT | 716/25/1 |  | 0.0178 | 66.89/57.22/62.23 | 21.6/26.5/NA | -4.39 | 0.02431 | -5.312 | 0.009 |
| APOC2p1187APOC4p4491/rs111782345 | C4-3'/C2-5' | AG/GG | 25/689 |  | 0.0178 | 72.68/66.23 | 21.6/21.8 | 3.17 | 0.13276 | 3.232 | 0.134 |
| APOC2p1229APOC4p4533/rs112698600 | C4-3'/C2-5' | CC/CT | 713/20 |  | 0.0140 | 66.32/72.07 | 21.8/24.4 | 2.78 | 0.23927 | 2.648 | 0.262 |
| APOC2p1275APOC4p4579/rs111356234 | C4-3'/C2-5' | GA/GG | 52/685 |  | 0.0352 | 66.08/66.63 | 20.4/22.0 | -0.23 | 0.87888 | 0.502 | 0.749 |
| APOC2p1357APOC4p4661/rs2288912 | C4-3'/C2-5' | CC/GC/GG | 51/289/412 |  | 0.2581 | 66.99/69.37/64.75 | 17.8/22.7/21.3 | 1.37 | 0.02344 | 1.422 | 0.020 |
| APOC2p1540APOC4p4844/rs75463753 | C2-Intron 1 | AA/GA/GG | 11/130/555 |  | 0.1079 | 67.06/68.51/65.68 | 14.8/23.3/21.8 | 1.08 | 0.21814 | 0.967 | 0.278 |
| APOC2p2486/rs9304645 | Intron 1 | AA/GA/GG | 89/368/291 |  | 0.3655 | 66.34/66.3/66.87 | 23.7/22.1/21.0 | -0.21 | 0.71760 | -0.010 | 0.987 |
| APOC2p2935/rs11879392 | Intron 1 | CC/GC | 705/19 |  | 0.0135 | 66.34/66.92 | 22.0/16.8 | 0.44 | 0.85516 | 0.221 | 0.927 |
| APOC2p3010/rs10419086 | Intron 1 | AA/AG/GG | 541/150/15 |  | 0.1253 | 67.99/64.76/69.3 | 22.3/20.8/15.5 | -0.91 | 0.25810 | -0.920 | 0.256 |
| APOC2p3692/rs12721060 | Intron 1 | GT/TT | 21/633 |  | 0.0172 | 71.63/66.93 | 23.9/21.4 | 2.28 | 0.31473 | 1.969 | 0.388 |
| APOC2p3778/rs5120 | Intron 1 | AA/AT/TT | 502/232/24 |  | 0.1845 | 66/68.31/69.94 | 22.2/21.8/12.2 | 1.11 | 0.10763 | 1.007 | 0.152 |
| APOC2p3805/rs7257095 | Intron 1 | CC/CG/GG | 510/211/15 |  | 0.1649 | 66.08/67.81/68.2 | 21.3/23.2/23.9 | 0.72 | 0.33716 | 0.832 | 0.279 |
| APOC2p3814/rs10422603 | Intron 1 | GG/GT/TT | 62/309/351 |  | 0.3008 | 67.74/66.13/66.92 | 24.3/22.8/20.7 | -0.10 | 0.87424 | -0.098 | 0.874 |
| APOC2p3892/rs5121 | Exon 2 | CC/TC/TT | 675/48/1 |  | 0.0358 | 66.57/65.72/76.29 | 22.3/16.6/NA | -0.03 | 0.98356 | -0.432 | 0.785 |
| APOC2p4086/rs114780592 | Intron 2 | GA/GG | 41/699 |  | 0.0278 | 72.68/66.2 | 25.8/21.6 | 3.05 | 0.06690 | 2.931 | 0.079 |
| APOC2p4118/rs201709243 | Exon 3 | GA/GG | 1/720 |  | 0.0007 | 60.37/66.56 | NA/22.0 | -2.87 | 0.78260 | -3.165 | 0.761 |
| APOC2p4319/rs5123 | Intron 3 | AA/GA/GG | 6/73/648 |  | 0.0592 | 72.9/64.68/66.64 | 18.3/19.5/22.1 | -0.25 | 0.82293 | -0.201 | 0.861 |
| APOC2p4513/rs180809422 | Intron 3 | AA/AC/CC | 671/16/1 |  | 0.0135 | 65.74/73.83/55.79 | 21.9/24.1/NA | 2.64 | 0.26202 | 3.842 | 0.146 |
| APOC2p4587/rs5126 | Exon 4 | AA/CA/CC | 635/69/1 |  | 0.0499 | 66.24/70.29/52.22 | 21.9/20.8/NA | 1.71 | 0.18011 | 1.918 | 0.142 |
| APOC2p4754/rs7253690 | Exon 4 | AA/GA/GG | 6/79/673 |  | 0.0606 | 72.96/66.14/66.87 | 18.3/18.1/22.3 | 0.19 | 0.85817 | 0.271 | 0.808 |
| APOC2p4853/rs150448996 | 3'flanking | DD/WD/WW | 385/283/56 |  | 0.2736 | 65.87/68.51/61.64 | 20.7/23.7/20.6 | -0.05 | 0.93724 | -0.147 | 0.813 |
| APOC2p4973/rs199828513 | 3'flanking | WI/WW | 12/706 |  | 0.0082 | 74.02/66.82 | 25.3/21.5 | 3.46 | 0.24889 | 3.352 | 0.284 |
| APOC2p5004/rs10421404 | 3'flanking | CC/CT/TT | 370/329/55 |  | 0.2908 | 66.9/66.25/68.08 | 20.7/22.7/23.8 | -0.04 | 0.94186 | -0.111 | 0.856 |
| APOC2p5018/rs78403558 | 3'flanking | DD/WD/WW | 1/51/711 |  | 0.0352 | 81.34/68.78/66.53 | NA/22.5/21.8 | 1.27 | 0.37773 | 0.655 | 0.656 |
| APOC2p5310/rs7258345 | 3'flanking | GG/GT/TT | 327/314/59 |  | 0.3067 | 66.36/67.39/66.36 | 23.1/21.3/16.0 | 0.29 | 0.63627 | 0.208 | 0.737 |
| APOC2p5398/rs12709889 | 3'flanking | AA/GA/GG | 49/279/404 |  | 0.2587 | 61.99/68.59/65.74 | 21.1/23.1/20.9 | 0.16 | 0.79862 | 0.117 | 0.851 |
| APOC2p5491 | 3'flanking | CC/TC | 740/1 |  | 0.0007 | 66.49/49.23 | 21.9/NA | -8.06 | 0.43745 | -8.709 | 0.401 |
| APOC2p5512/rs12721064 | 3'flanking | CC/CT | 756/13 |  | 0.0083 | 66.78/61.92 | 21.9/17.5 | -2.23 | 0.43911 | -1.462 | 0.651 |
| APOC2p5562 | 3'flanking | CG/GG | 24/701 |  | 0.0175 | 64.44/66.45 | 19.8/22.0 | -0.92 | 0.67145 | -1.053 | 0.626 |
| APOC2p5586/rs73558127 | 3'flanking | GG/GT/TT | 9/125/589 |  | 0.1001 | 71.92/66.13/66.69 | 28.0/21.4/22.1 | 0.13 | 0.88663 | 0.575 | 0.536 |
| APOC2p5815/rs10423208 | 3'flanking | AA/GA/GG | 339/321/73 |  | 0.3164 | 66.03/66.9/66.84 | 23.3/21.5/17.8 | 0.34 | 0.55855 | 5.383 | 0.174 |
| APOC2p5922/rs10422888 | 3'flanking | AA/AG/GG | 593/100/5 |  | 0.0784 | 66.04/70.45/60.04 | 21.7/21.5/30.7 | 1.47 | 0.14838 | 0.225 | 0.706 |
| APOC2p5965 | 3'flanking | GA/GG | 2/741 |  | 0.0013 | 44.73/66.55 | 10.8/21.9 | -10.45 | 0.15456 | 0.961 | 0.358 |
| APOC2p6334 | 3'flanking | GA/GG | 15/749 |  | 0.0096 | 72.76/66.62 | 15.3/21.9 | 3.15 | 0.24150 | -10.964 | 0.135 |
| MAF is the minor allele frequency; GT is genotype; GT count is the number of individuals in each genotype group; GT_SD is standard deviation of lipid traits mean in each genotype group; *Adjusted for relevant covariates, **Adjusted for *APOE*2/E*4* SNPs in addition to the covariates. APOC2p5771 is excluded due to missing data. | | | | | | | | | | | |
