# Supplementary material for: Apolipoprotein E-C1-C4-C2 gene cluster region and inter-individual variation in plasma lipoprotein levels: a comprehensive genetic association study in two ethnic groups
Source: PLoS One. 2019 Mar 26;14(3):e0214060. doi: 10.1371/journal.pone.0214060 (PMC6435132; doi:10.1371/journal.pone.0214060)
Supplement: S21 Table — MAF is the minor allele frequency; GT is genotype; GT count is the number of individuals in each genotype group; GT_SD is standard deviation of lipid traits mean in each genotype group; *Adjusted for relevant covariates, **Adjusted for APOE*2/E*4 SNPs in addition to the covariates. APOC2p4118/rs201709243 is excluded due to missing data. (DOCX) [file pone.0214060.s021.docx]

S21 Table. Single-site association analysis results for HDL-C in ABs

| **Variant Name/RefSNP ID** | **Location** | **Genotype** | **GT Count** | **MAF** | **Adjusted Mean of plasma of HDL-C*** | **GT_SD*** | **Beta*** | **P*** | **Adj. B** | **Adj. P** |
| --- | --- | --- | --- | --- | --- | --- | --- | --- | --- | --- |
| APOE73/rs1081101 | 5'flanking | CC/CT/TT | 652/82/5 | 0.0611 | 47.72/49.03/48.5 | 12.6/13.7/5.7 | 0.30 | 0.42732 | 0.321 | 0.415 |
| APOE173 | 5'flanking | AA/GA | 738/3 | 0.0020 | 47.84/40.69 | 12.7/11.3 | -2.10 | 0.32058 | -2.370 | 0.267 |
| APOE308/rs769445 | 5'flanking | CC/TC | 730/11 | 0.0072 | 47.86/44.21 | 12.7/11.0 | -1.06 | 0.34278 | -1.181 | 0.316 |
| APOE560/rs449647 | 5'flanking | AA/AT/TT | 296/333/103 | 0.3663 | 48.06/47.85/46.63 | 12.5/13.0/12.7 | -0.18 | 0.35635 | -0.178 | 0.411 |
| APOE618 | 5'flanking | GC/GG | 1/754 | 0.0006 | 13.45/47.78 | NA/12.6 | -12.25 | 0.00079 | -12.201 | 0.001 |
| APOE624/rs769446 | 5'flanking | TC/TT | 11/681 | 0.0077 | 49.89/47.71 | 15.1/12.9 | 0.65 | 0.56874 | 0.186 | 0.878 |
| APOE832/rs405509 | 5'flanking | GG/GT/TT | 421/268/59 | 0.2561 | 47.91/48.13/45.19 | 12.9/12.7/11.3 | -0.19 | 0.36119 | -0.094 | 0.691 |
| APOE1109/rs9282609 | Splice site | CC/TC/TT | 679/54/4 | 0.0415 | 47.63/50.15/49.36 | 12.6/13.7/6.6 | 0.61 | 0.17876 | 0.607 | 0.189 |
| APOE1163/rs440446 | Intron 1 | CC/CG/GG | 8/126/562 | 0.1004 | 45.27/46.49/48.41 | 14.2/12.5/12.8 | -0.54 | 0.09464 | -0.603 | 0.074 |
| APOE1231 | Intron 1 | GA/GG | 18/722 | 0.0125 | 46.84/47.87 | 11.5/12.7 | -0.27 | 0.75500 | -0.121 | 0.893 |
| APOE1279/rs877973 | Intron 1 | AA/CA/CC | 4/79/657 | 0.0597 | 49.78/49.36/47.61 | 11.2/12.3/12.8 | 0.51 | 0.19812 | 0.567 | 0.167 |
| APOE1539/rs184686013 | Intron 1 | AA/AG/GG | 725/11/1 | 0.0086 | 47.68/54.07/46.37 | 12.7/13.5/NA | 1.35 | 0.15682 | 1.333 | 0.180 |
| APOE2072/rs189660912 | Intron 2 | GA/GG | 12/726 | 0.0079 | 46.66/47.85 | 15.0/12.7 | -0.40 | 0.70658 | -0.498 | 0.643 |
| APOE2269/rs61357706 | Intron 2 | GA/GG | 26/714 | 0.0169 | 50.39/47.78 | 12.6/12.7 | 0.77 | 0.29121 | 0.929 | 0.217 |
| APOE2440/rs769450 | Intron 2 | AA/AG/GG | 106/303/260 | 0.3870 | 46.94/48.89/47.64 | 13.4/13.3/12.3 | -0.02 | 0.93935 | 0.068 | 0.787 |
| APOE3673/rs769453 | Intron 3 | CC/GC | 729/10 | 0.0066 | 47.85/44.06 | 12.7/11.5 | -1.10 | 0.34567 | -1.184 | 0.314 |
| APOE3937/rs429358 | Exon 4 | CC/CT/TT | 57/284/400 | 0.2656 | 46.45/47.78/48 | 12.5/12.0/13.3 | -0.14 | 0.50743 | - | - |
| APOE4036/rs769455 | Exon 4 | CC/TC/TT | 700/28/1 | 0.0200 | 47.95/48.87/15.7 | 12.7/12.5/NA | -0.46 | 0.48274 | -0.381 | 0.575 |
| APOE4075/rs7412 | Exon 4 | AA/GA/GG | 2/84/662 | 0.0605 | 50.84/49.96/47.54 | 12.7/11.5/12.8 | 0.75 | 0.06615 | - | - |
| APOE4569 | 3'UTR | GG/GT | 738/1 | 0.0007 | 47.84/66.63 | 12.7/NA | 5.29 | 0.14968 | 5.322 | 0.150 |
| APOE5223 | 3'flanking | CC/CG | 750/8 | 0.0051 | 47.74/48.97 | 12.7/12.1 | 0.39 | 0.76300 | 0.415 | 0.751 |
| APOE5231 | 3'flanking | GG/GT/TT | 1/35/702 | 0.0270 | 51.97/46.78/47.88 | NA/11.1/12.8 | -0.18 | 0.76449 | -0.081 | 0.896 |
| rs439401 | Intergenic | CC/CT/TT | 580/131/14 | 0.1092 | 48.34/46.18/44.72 | 12.8/12.4/12.1 | -0.60 | 0.04448 | -0.580 | 0.065 |
| APOC1rs445925 | Intergenic | AA/GA/GG | 68/307/362 | 0.2990 | 47.56/48.18/47.55 | 12.3/12.0/13.2 | 0.10 | 0.64004 | 0.200 | 0.627 |
| APOC1p720ins4/rs11568822 | 5'flanking | II/WI/WW | 59/274/394 | 0.2737 | 48.48/48.36/47.27 | 11.6/12.3/13.2 | 0.27 | 0.20872 | 0.144 | 0.543 |
| APOC1p894/rs190454394 | 5'flanking | CC/CT | 732/3 | 0.0020 | 47.81/50.62 | 12.7/21.6 | 0.74 | 0.72955 | 0.744 | 0.729 |
| APOC1p1166/rs72654452 | Intron 1 | CC/CT/TT | 707/42/2 | 0.0308 | 47.84/46.05/44.23 | 12.5/15.1/12.2 | -0.61 | 0.25404 | -0.402 | 0.473 |
| APOC1p1331/rs10408994 | Intron 2 | AG/GG | 99/631 | 0.0666 | 48.89/47.56 | 14.7/12.4 | 0.35 | 0.38621 | 0.322 | 0.437 |
| APOC1p1526/rs5114 | Intron 2 | CC/CT/TT | 650/76/4 | 0.0579 | 47.54/49.45/49.82 | 12.8/12.5/11.2 | 0.55 | 0.17450 | 0.615 | 0.143 |
| APOC1p1642 | Intron 2 | CC/CT | 735/15 | 0.0103 | 47.86/45.82 | 12.7/11.7 | -0.59 | 0.53986 | -0.493 | 0.616 |
| APOC1p1684/rs12709881 | Intron 2 | AA/GA/GG | 8/127/612 | 0.0973 | 47.84/49.16/47.6 | 8.5/12.3/12.7 | 0.40 | 0.20729 | 0.458 | 0.163 |
| APOC1p3358 | Intron 3 | AA/GA | 700/3 | 0.0021 | 47.78/59.16 | 12.8/10.3 | 3.44 | 0.10895 | 3.277 | 0.130 |
| APOC1p3423/rs389261 | Intron 3 | AA/GA/GG | 77/320/310 | 0.3310 | 48.12/48.58/47.01 | 12.8/14.0/11.5 | 0.24 | 0.26136 | 0.336 | 0.144 |
| APOC1p3573/rs10424339 | Intron 3 | AA/GA/GG | 16/173/534 | 0.1396 | 45.5/50.39/47.19 | 13.9/13.8/12.2 | 0.55 | 0.04550 | 0.575 | 0.048 |
| APOC1p5006/rs112528434 | Intron 3 | GG/GT/TT | 569/99/6 | 0.0850 | 47.76/48.84/49.39 | 12.9/13.4/7.8 | 0.32 | 0.38790 | 0.389 | 0.301 |
| APOC1p5053/rs12721052 | Intron 3 | DD/WD/WW | 40/245/461 | 0.2200 | 46.26/47.2/48.25 | 12.4/13.0/12.4 | -0.31 | 0.16174 | -0.327 | 0.173 |
| APOC1p5667/rs12721054 | 3'UTR | AA/GA/GG | 506/159/16 | 0.1446 | 47.56/48.09/49.87 | 13.0/12.9/10.8 | 0.23 | 0.42209 | 0.188 | 0.527 |
| APOC1p5926/rs56131196 | 3'flanking | AA/AG/GG | 21/210/492 | 0.1745 | 49.89/48.35/47.53 | 9.9/12.3/13.1 | 0.31 | 0.22541 | 0.311 | 0.243 |
| rs4803770 | Intergenic | CC/GC/GG | 391/274/56 | 0.2695 | 48.61/47.03/47.95 | 12.4/13.3/11.2 | -0.27 | 0.21475 | -0.211 | 0.359 |
| HCR1p424/rs117664574 | HCR1 | AG/GG | 11/725 | 0.0073 | 54.72/47.7 | 13.2/12.7 | 2.06 | 0.06453 | 2.136 | 0.058 |
| HCR1p575/rs157599 | HCR1 | AA/AG/GG | 275/309/90 | 0.3595 | 47.6/48.7/48.8 | 11.6/14.1/12.6 | 0.19 | 0.36879 | 0.256 | 0.248 |
| rs5112 | *APOC1P1* | CC/GC/GG | 200/328/163 | 0.4797 | 46.88/48.82/48.57 | 11.9/12.3/13.8 | 0.25 | 0.18503 | 0.248 | 0.228 |
| rs7259004 | *APOC1P1* | CC/CG/GG | 73/285/366 | 0.3020 | 48.65/48.54/47.07 | 13.3/13.2/12.2 | 0.29 | 0.15235 | 0.257 | 0.242 |
| HCR2p188/rs35136575 | HCR2 | CC/GC/GG | 528/173/25 | 0.1546 | 47.26/49.37/46.84 | 12.3/13.8/13.7 | 0.30 | 0.23716 | 0.301 | 0.256 |
| HCR2p286 | HCR2 | AA/AG/GG | 3/63/668 | 0.0457 | 40.61/45.9/47.95 | 12.9/12.6/12.8 | -0.70 | 0.11626 | -0.697 | 0.124 |
| HCR2p523/rs118004808 | HCR2 | CC/TC | 734/4 | 0.0026 | 47.78/47.96 | 12.7/18.0 | 0.02 | 0.99167 | 0.014 | 0.994 |
| APOC4p368 | 5’ flanking | TC/TT | 3/741 | 0.0019 | 50.1/47.75 | 29.7/12.7 | 0.33 | 0.87548 | 0.344 | 0.872 |
| APOC4p637/rs113814026 | 5’ flanking | GG/GT/TT | 678/67/1 | 0.0452 | 47.73/48.19/41.63 | 12.7/12.1/NA | 0.10 | 0.82864 | 0.091 | 0.847 |
| APOC4p757/rs12721105 | 5’ flanking | GG/GT/TT | 700/53/2 | 0.0376 | 47.83/46.7/54.95 | 12.6/13.3/37.2 | -0.19 | 0.69377 | -0.344 | 0.489 |
| APOC4p1088 | Intron 1 | GT/TT | 2/728 | 0.0013 | 34.51/47.87 | 19.7/12.7 | -4.26 | 0.10128 | -4.258 | 0.104 |
| APOC4p1130 | Intron 1 | CT/TT | 1/736 | 0.0007 | 82.19/47.76 | NA/12.7 | 9.46 | 0.00979 | 9.473 | 0.010 |
| APOC4p1192/rs113745034 | Intron 1 | GA/GG | 18/690 | 0.0124 | 48.92/47.83 | 12.4/12.9 | 0.35 | 0.69302 | 0.336 | 0.707 |
| APOC4p1325del3 | Intron 1 | WD/WW | 36/699 | 0.0245 | 49.06/47.81 | 16.2/12.5 | 0.30 | 0.63292 | 0.257 | 0.688 |
| APOC4p1430ins | Intron 1 | II/WI/WW | 1/43/617 | 0.0341 | 28.01/51.13/47.62 | NA/11.9/13.2 | 0.69 | 0.22691 | 0.671 | 0.247 |
| APOC4p2099/rs111339708 | Intron 1 | GG/GT | 728/21 | 0.0141 | 47.75/48.85 | 12.7/12.7 | 0.35 | 0.66780 | 0.394 | 0.647 |
| APOC4p2467/rs115225947 | Intron 1 | GA/GG | 21/728 | 0.0141 | 48.04/47.79 | 14.5/12.7 | 0.03 | 0.96646 | -0.026 | 0.975 |
| APOC4p2559/rs5155 | Intron 1 | CC/CT/TT | 607/134/6 | 0.0986 | 47.79/47.95/37.85 | 12.7/12.4/12.5 | -0.20 | 0.53148 | -0.197 | 0.559 |
| APOC4p2607/rs5156 | Intron 1 | AG/GG | 18/697 | 0.0129 | 49.11/47.76 | 12.5/12.8 | 0.43 | 0.62963 | 0.330 | 0.719 |
| APOC4p2623/rs5157 | Intron 1 | CC/CT/TT | 502/215/20 | 0.1723 | 47.74/48.15/42.77 | 12.9/12.5/8.5 | -0.14 | 0.59553 | -0.135 | 0.608 |
| APOC4p2640/rs5158 | Intron 1 | CC/CT | 714/32 | 0.0213 | 47.8/46.98 | 12.6/15.3 | -0.29 | 0.66191 | -0.390 | 0.565 |
| APOC4p2678/rs148564866 | Intron 1 | GC/GG | 12/719 | 0.0086 | 47.93/47.81 | 12.0/12.8 | 0.07 | 0.94518 | 0.143 | 0.895 |
| APOC4p2767/rs127721107 | Intron 1 | GG/GT | 691/37 | 0.0254 | 47.63/49.33 | 12.8/11.6 | 0.56 | 0.37292 | 0.588 | 0.353 |
| APOC4p3348 | Intron 1 | AG/GG | 1/735 | 0.0007 | 90.23/47.79 | NA/12.6 | 11.56 | 0.00153 | 10.916 | 0.003 |
| APOC2p75APOC4p3380/rs12721104 | C4-Intron 1 | AA/GA/GG | 13/176/553 | 0.1368 | 42.19/48.31/47.79 | 16.0/12.8/12.6 | -0.11 | 0.69346 | -0.097 | 0.740 |
| APOC2p194APOC4p3498/rs1132899 | C4-Exon 2 | CC/CT/TT | 429/279/40 | 0.2368 | 47.94/47.82/46.02 | 12.9/12.4/12.2 | -0.14 | 0.53341 | -0.133 | 0.564 |
| APOC2p228/rs5164 | C4-Exon 2 | AG/GA/GG | 9/1/723 | 0.0066 | 48.31/21.33/47.81 | 11.9/NA/12.7 | -0.72 | 0.54187 | -0.270 | 0.830 |
| APOC2p288APOC4p3592/rs12691090 | C4-Exon 2 | CC/CT | 692/40 | 0.0272 | 47.64/49.98 | 12.8/12.0 | 0.75 | 0.21469 | 0.716 | 0.240 |
| APOC2p396APOC4p3700 | C4-Intron 2 | GA/GG | 1/711 | 0.0007 | 68.22/47.77 | NA/12.8 | 5.90 | 0.11176 | 5.994 | 0.109 |
| APOC2p488APOC4p3792/rs5165 | C4-Intron 2 | GA/GG | 22/707 | 0.0146 | 47.63/47.84 | 12.3/12.8 | -0.04 | 0.96019 | -0.098 | 0.903 |
| APOC2p623APOC4p3927/rs5167 | C4-Exon 3 | GG/GT/TT | 164/360/222 | 0.4594 | 47.74/47.89/47.38 | 13.0/12.5/12.9 | 0.05 | 0.77107 | 0.063 | 0.743 |
| APOC2p665APOC4p3969/rs138548797 | C4-Exon 3 | AA/CA | 721/13 | 0.0086 | 47.81/47.51 | 12.8/11.9 | -0.06 | 0.94985 | 0.042 | 0.969 |
| APOC2p708APOC4p4012 | C4-Exon 3 | GA/GG | 1/731 | 0.0007 | 24.53/47.82 | NA/12.7 | -7.15 | 0.05187 | -7.178 | 0.053 |
| APOC2p853APOC4p4157/rs10425530 | C4-3' UTR | AA/GA/GG | 7/148/584 | 0.1100 | 46.9/48.65/47.54 | 14.3/12.8/12.7 | 0.24 | 0.43314 | 0.307 | 0.333 |
| APOC2p1042APOC4p4346/rs12709885 | C4-3’/C2-5’ | AA/TA/TT | 713/22/1 | 0.0178 | 47.7/51.29/53.52 | 12.7/11.9/NA | 1.10 | 0.13505 | 1.345 | 0.080 |
| APOC2p1187APOC4p4491/rs111782345 | C4-3’/C2-5’ | AG/GG | 25/686 | 0.0178 | 49.54/47.8 | 10.1/12.9 | 0.58 | 0.44007 | 0.558 | 0.470 |
| APOC2p1229APOC4p4533/rs112698600 | C4-3’/C2-5’ | CC/CT | 707/20 | 0.0140 | 47.74/50.43 | 12.7/12.0 | 0.83 | 0.31821 | 0.871 | 0.301 |
| APOC2p1275APOC4p4579/rs111356234 | C4-3’/C2-5’ | GA/GG | 51/680 | 0.0352 | 47.4/47.82 | 14.6/12.6 | -0.18 | 0.73230 | -0.276 | 0.625 |
| APOC2p1357APOC4p4661/rs2288912 | C4-3’/C2-5’ | CC/GC/GG | 49/284/407 | 0.2581 | 46.71/48.36/47.62 | 11.4/12.6/13.0 | 0.05 | 0.80869 | 0.121 | 0.587 |
| APOC2p1540APOC4p4844/rs75463753 | C2-Intron 1 | AA/GA/GG | 11/127/552 | 0.1079 | 47.48/47.99/47.65 | 14.1/11.8/13.2 | 0.09 | 0.77127 | 0.140 | 0.666 |
| APOC2p2486/rs9304645 | Intron 1 | AA/GA/GG | 86/365/286 | 0.3655 | 47.83/47.89/47.32 | 14.5/12.4/12.5 | 0.09 | 0.65299 | 0.043 | 0.841 |
| APOC2p2935/rs11879392 | Intron 1 | CC/GC | 698/20 | 0.0135 | 47.79/49.99 | 12.6/17.5 | 0.57 | 0.49871 | 0.569 | 0.500 |
| APOC2p3010/rs10419086 | Intron 1 | AA/AG/GG | 536/146/13 | 0.1253 | 47.78/48.88/46.09 | 12.7/13.8/11.1 | 0.15 | 0.62186 | 0.093 | 0.758 |
| APOC2p3692/rs12721060 | Intron 1 | GT/TT | 22/629 | 0.0172 | 50.36/47.98 | 11.5/13.0 | 0.76 | 0.35360 | 0.843 | 0.307 |
| APOC2p3778/rs5120 | Intron 1 | AA/AT/TT | 496/227/24 | 0.1845 | 47.62/48.02/47.5 | 13.0/11.9/14.4 | 0.08 | 0.74123 | 0.164 | 0.518 |
| APOC2p3805/rs7257095 | Intron 1 | CC/CG/GG | 508/207/16 | 0.1649 | 47.93/47.64/45.13 | 13.0/12.1/12.5 | -0.16 | 0.53621 | -0.144 | 0.599 |
| APOC2p3814/rs10422603 | Intron 1 | GG/GT/TT | 62/310/345 | 0.3008 | 48.94/48.34/47.37 | 14.7/12.4/12.7 | 0.25 | 0.23836 | 0.215 | 0.332 |
| APOC2p3892/rs5121 | Exon 2 | CC/TC/TT | 669/50/1 | 0.0358 | 47.88/47.21/57.02 | 12.9/10.9/NA | -0.03 | 0.95269 | -0.175 | 0.752 |
| APOC2p4086/rs114780592 | Intron 2 | GA/GG | 41/693 | 0.0278 | 49.23/47.69 | 12.5/12.8 | 0.49 | 0.40801 | 0.486 | 0.418 |
| APOC2p4319/rs5123 | Intron 3 | AA/GA/GG | 6/75/642 | 0.0592 | 48.08/47.3/47.88 | 8.9/13.2/12.7 | -0.14 | 0.71873 | -0.091 | 0.823 |
| APOC2p4513/rs180809422 | Intron 3 | AA/AC/CC | 660/16/1 | 0.0135 | 47.73/50.5/52.34 | 12.9/12.7/NA | 0.82 | 0.32552 | 0.916 | 0.333 |
| APOC2p4587/rs5126 | Exon 4 | AA/CA/CC | 631/69/1 | 0.0499 | 47.83/48.33/42.04 | 12.8/12.0/NA | 0.12 | 0.80049 | 0.057 | 0.904 |
| APOC2p4754/rs7253690 | Exon 4 | AA/GA/GG | 6/79/662 | 0.0606 | 47.92/47.81/47.77 | 8.9/13.4/12.7 | -0.01 | 0.98010 | 0.007 | 0.986 |
| APOC2p4853/rs150448996 | 3'flanking | DD/WD/WW | 381/283/56 | 0.2736 | 48.34/47.18/47.93 | 13.1/12.1/13.7 | -0.18 | 0.40916 | -0.197 | 0.372 |
| APOC2p4973/rs199828513 | 3'flanking | WI/WW | 12/695 | 0.0082 | 45.89/47.98 | 11.4/12.9 | -0.60 | 0.58323 | -0.592 | 0.605 |
| APOC2p5004/rs10421404 | 3'flanking | CC/CT/TT | 364/324/55 | 0.2908 | 47.43/47.85/48.84 | 12.6/12.4/15.5 | 0.16 | 0.46461 | 0.126 | 0.571 |
| APOC2p5018/rs78403558 | 3'flanking | DD/WD/WW | 1/51/700 | 0.0352 | 63.14/47.35/47.77 | NA/15.3/12.5 | -0.004 | 0.99390 | 0.071 | 0.893 |
| APOC2p5310/rs7258345 | 3'flanking | GG/GT/TT | 323/307/56 | 0.3067 | 47.88/48.51/48.08 | 13.3/12.6/12.4 | 0.11 | 0.61363 | 0.133 | 0.565 |
| APOC2p5398/rs12709889 | 3'flanking | AA/GA/GG | 49/278/399 | 0.2587 | 47.86/47.3/48.09 | 13.7/12.0/13.2 | -0.12 | 0.59063 | -0.113 | 0.616 |
| APOC2p5491 | 3'flanking | CC/TC | 734/1 | 0.0007 | 47.76/70.37 | 12.7/NA | 6.53 | 0.07620 | 6.635 | 0.074 |
| APOC2p5512/rs12721064 | 3'flanking | CC/CT | 746/12 | 0.0083 | 47.75/47.73 | 12.7/10.4 | 0.05 | 0.96332 | -0.580 | 0.628 |
| APOC2p5562 | 3'flanking | CG/GG | 25/697 | 0.0175 | 45.55/47.83 | 11.8/12.8 | -0.67 | 0.37221 | -0.776 | 0.308 |
| APOC2p5586/rs73558127 | 3'flanking | GG/GT/TT | 10/126/582 | 0.1001 | 47.55/49.36/47.38 | 13.2/11.9/12.9 | 0.47 | 0.13813 | 0.424 | 0.195 |
| APOC2p5771 | 3'flanking | WD/WW | 7/716 | 0.0047 | 45.84/47.8 | 13.9/12.6 | -0.58 | 0.67563 | -0.796 | 0.570 |
| APOC2p5815/rs10423208 | 3'flanking | AA/GA/GG | 340/316/71 | 0.3164 | 47.59/48.15/47.14 | 13.0/12.6/12.3 | 0.03 | 0.87590 | 0.084 | 0.695 |
| APOC2p5922/rs10422888 | 3'flanking | AA/AG/GG | 590/101/5 | 0.0784 | 47.93/47.55/52.87 | 12.6/12.9/30.7 | -0.01 | 0.98595 | 0.077 | 0.838 |
| APOC2p5965 | 3'flanking | GA/GG | 2/735 | 0.0013 | 57.22/47.78 | 19.5/12.7 | 2.75 | 0.29064 | 2.840 | 0.279 |
| APOC2p6334 | 3'flanking | GA/GG | 15/738 | 0.0096 | 49.99/47.73 | 16.1/12.5 | 0.60 | 0.52729 | 0.579 | 0.546 |
| MAF is the minor allele frequency; GT is genotype; GT count is the number of individuals in each genotype group; GT_SD is standard deviation of lipid traits mean in each genotype group; *Adjusted for relevant covariates, **Adjusted for *APOE*2/E*4* SNPs in addition to the covariates. APOC2p4118/rs201709243 is excluded due to missing data. | | | | | | | | | | |
