# Supplementary material for: Apolipoprotein E-C1-C4-C2 gene cluster region and inter-individual variation in plasma lipoprotein levels: a comprehensive genetic association study in two ethnic groups
Source: PLoS One. 2019 Mar 26;14(3):e0214060. doi: 10.1371/journal.pone.0214060 (PMC6435132; doi:10.1371/journal.pone.0214060)
Supplement: S22 Table — MAF is the minor allele frequency; GT is genotype; GT count is the number of individuals in each genotype group; GT_SD is standard deviation of lipid traits mean in each genotype group; *Adjusted for relevant covariates, **Adjusted for APOE*2/E*4 SNPs in addition to the covariates. APOC2p4118/rs201709243 is excluded due to missing data. (DOCX) [file pone.0214060.s022.docx]

S22 Table. Single-site association analysis results for ApoA1 in ABs

|  |  |  | **ApoA1^a^** |  |  | |  |  |  |  |  |
| --- | --- | --- | --- | --- | --- | --- | --- | --- | --- | --- | --- |
| **Variant Name/RefSNP ID** | **Location** | **Genotype** | **GT Count** | **MAF** | **Adjusted Mean of plasma ApoA1 *** | | **GT_SD*** | **Beta*** | **P*** | **Adj. B.** | **Adj. P** |
| APOE73/rs1081101 | 5'flanking | CC/CT/TT | 658/82/5 | 0.0611 | 136.86/138.93/121.99 | | 27.3/30.5/23.6 | -0.003 | 0.99719 | 0.077 | 0.937 |
| APOE173 | 5'flanking | AA/GA | 744/3 | 0.0020 | 136.65/157.72 | | 27.6/32.9 | 6.860 | 0.19127 | 6.024 | 0.248 |
| APOE308/rs769445 | 5'flanking | CC/TC | 736/11 | 0.0072 | 136.6/146.23 | | 27.6/28.1 | 3.205 | 0.24610 | 3.901 | 0.175 |
| APOE560/rs449647 | 5'flanking | AA/AT/TT | 304/337/101 | 0.3663 | 137.15/136.83/133.49 | | 28.1/27.1/30.5 | -0.491 | 0.31886 | -0.207 | 0.695 |
| APOE618 | 5'flanking | GC/GG | 1/765 | 0.0006 | 83.01/136.66 | | NA/27.7 | -18.420 | 0.04342 | -18.137 | 0.045 |
| APOE624/rs769446 | 5'flanking | TC/TT | 11/693 | 0.0077 | 146.85/136.47 | | 18.7/27.9 | 3.550 | 0.20204 | 0.333 | 0.908 |
| APOE832/rs405509 | 5'flanking | GG/GT/TT | 431/269/59 | 0.2561 | 137.58/136.56/132.03 | | 28.2/27.3/26.8 | -0.668 | 0.19928 | -0.213 | 0.713 |
| APOE1109/rs9282609 | Splice site | CC/TC/TT | 685/54/4 | 0.0415 | 136.58/139.25/126.95 | | 27.4/32.1/27.3 | 0.237 | 0.83418 | 0.267 | 0.815 |
| APOE1163/rs440446 | Intron 1 | CC/CG/GG | 8/127/568 | 0.1004 | 131.72/136.2/137.15 | | 25.8/27.4/27.9 | -0.443 | 0.58046 | -0.759 | 0.356 |
| APOE1231 | Intron 1 | GA/GG | 19/727 | 0.0125 | 133.54/136.87 | | 28.3/27.6 | -1.120 | 0.59594 | 0.044 | 0.984 |
| APOE1279/rs877973 | Intron 1 | AA/CA/CC | 4/82/660 | 0.0597 | 129.68/140.9/136.39 | | 33.5/25.8/27.8 | 1.060 | 0.27597 | 1.348 | 0.174 |
| APOE1539/rs184686013 | Intron 1 | AA/AG/GG | 732/11/1 | 0.0086 | 136.35/156.58/122.97 | | 27.6/31.3/NA | 4.237 | 0.07380 | 3.905 | 0.109 |
| APOE2072/rs189660912 | Intron 2 | GA/GG | 12/732 | 0.0079 | 133.01/136.88 | | 31.9/27.7 | -1.347 | 0.61188 | -1.697 | 0.520 |
| APOE2269/rs61357706 | Intron 2 | GA/GG | 26/726 | 0.0169 | 140/136.7 | | 29.2/27.8 | 1.095 | 0.55027 | 1.536 | 0.409 |
| APOE2440/rs769450 | Intron 2 | AA/AG/GG | 109/303/262 | 0.3870 | 134.99/138.74/137.19 | | 26.9/28.3/28.4 | -0.168 | 0.73856 | -0.089 | 0.883 |
| APOE3673/rs769453 | Intron 3 | CC/GC | 735/10 | 0.0066 | 136.5/148.37 | | 27.7/28.4 | 3.935 | 0.17574 | 3.901 | 0.176 |
| APOE3937/rs429358 | Exon 4 | CC/CT/TT | 55/289/408 | 0.2656 | 128.85/136.59/137.88 | | 25.0/27.5/28.2 | -1.000 | 0.05924 | - | - |
| APOE4036/rs769455 | Exon 4 | CC/TC/TT | 705/28/1 | 0.0200 | 136.77/138.61/79.6 | | 27.4/27.5/NA | -0.708 | 0.66412 | -0.408 | 0.805 |
| APOE4075/rs7412 | Exon 4 | AA/GA/GG | 3/85/671 | 0.0605 | 172.7/145.92/135.3 | | 47.7/26.7/27.4 | 3.854 | 8.47E-05 | - | - |
| APOE4569 | 3'UTR | GG/GT | 744/1 | 0.0007 | 136.83/185.08 | | 27.7/NA | 15.528 | 0.08835 | 15.943 | 0.078 |
| APOE5223 | 3'flanking | CC/CG | 762/7 | 0.0051 | 136.65/140.31 | | 27.7/22.0 | 1.293 | 0.70886 | 1.531 | 0.656 |
| APOE5231 | 3'flanking | GG/GT/TT | 2/37/705 | 0.0270 | 125.57/138.13/136.85 | | 88.2/22.9/27.8 | -0.026 | 0.98493 | 0.735 | 0.602 |
| rs439401 | Intergenic | CC/CT/TT | 584/132/14 | 0.1092 | 137.09/135.48/134.62 | | 27.8/27.8/25.6 | -0.496 | 0.50161 | -0.535 | 0.486 |
| APOC1rs445925 | Intergenic | AA/GA/GG | 67/311/370 | 0.2990 | 137.99/137.53/136.18 | | 27.3/27.7/28.1 | 0.365 | 0.48026 | -0.019 | 0.984 |
| APOC1p720ins4/rs11568822 | 5'flanking | II/WI/WW | 63/277/393 | 0.2737 | 135.77/140.58/134.24 | | 24.5/27.4/28.3 | 1.033 | 0.04836 | 0.493 | 0.383 |
| APOC1p894/rs190454394 | 5'flanking | CC/CT | 738/3 | 0.0020 | 136.85/137.78 | | 27.7/28.4 | 0.403 | 0.93927 | 0.680 | 0.897 |
| APOC1p1166/rs72654452 | Intron 1 | CC/CT/TT | 717/43/2 | 0.0308 | 136.77/133.56/128.31 | | 27.8/27.3/12.3 | -1.093 | 0.40589 | -0.402 | 0.767 |
| APOC1p1331/rs10408994 | Intron 2 | AG/GG | 97/638 | 0.0666 | 134.89/136.82 | | 29.1/27.5 | -0.661 | 0.50656 | -0.961 | 0.345 |
| APOC1p1526/rs5114 | Intron 2 | CC/CT/TT | 653/79/4 | 0.0579 | 136.21/141.93/129.69 | | 28.0/26.1/33.5 | 1.380 | 0.16500 | 1.677 | 0.098 |
| APOC1p1642 | Intron 2 | CC/CT | 745/16 | 0.0103 | 136.9/131.29 | | 277/29.6 | -1.899 | 0.41040 | -0.926 | 0.692 |
| APOC1p1684/rs12709881 | Intron 2 | AA/GA/GG | 9/132/617 | 0.0973 | 128.67/140.21/136.26 | | 23.6/26.2/28.0 | 0.731 | 0.34454 | 1.114 | 0.157 |
| APOC1p3358 | Intron 3 | AA/GA | 704/3 | 0.0021 | 136.79/129.19 | | 28.0/16.1 | -2.340 | 0.66010 | -3.315 | 0.530 |
| APOC1p3423/rs389261 | Intron 3 | AA/GA/GG | 76/317/319 | 0.3310 | 133.68/137.66/137.23 | | 29.8/28.0/26.9 | -0.359 | 0.48713 | -0.031 | 0.956 |
| APOC1p3573/rs10424339 | Intron 3 | AA/GA/GG | 15/173/541 | 0.1396 | 128.38/139.71/136.32 | | 19.1/29.5/27.3 | 0.401 | 0.55931 | 0.302 | 0.674 |
| APOC1p5006/rs112528434 | Intron 3 | GG/GT/TT | 574/105/7 | 0.0850 | 136.73/139.26/137.09 | | 27.9/28.5/15.1 | 0.668 | 0.44123 | 1.049 | 0.233 |
| APOC1p5053/rs12721052 | Intron 3 | DD/WD/WW | 42/248/467 | 0.2200 | 133.78/136.4/137.22 | | 24.3/28.9/27.0 | -0.415 | 0.45060 | -0.388 | 0.504 |
| APOC1p5667/rs12721054 | 3'UTR | AA/GA/GG | 506/162/19 | 0.1446 | 135.85/140.83/134.9 | | 28.3/26.6/29.6 | 1.013 | 0.14096 | 0.894 | 0.201 |
| APOC1p5926/rs56131196 | 3'flanking | AA/AG/GG | 21/211/494 | 0.1745 | 141.08/138.62/136.05 | | 25.0/27.1/28.2 | 0.865 | 0.17334 | 1.047 | 0.105 |
| rs4803770 | Intergenic | CC/GC/GG | 393/276/57 | 0.2695 | 137.95/135.57/136.07 | | 26.5/30.1/24.9 | -0.529 | 0.32036 | -0.303 | 0.589 |
| HCR1p424/rs117664574 | HCR1 | AG/GG | 10/732 | 0.0073 | 154.41/136.55 | | 36.0/27.5 | 5.738 | 0.04801 | 6.024 | 0.037 |
| HCR1p575/rs157599 | HCR1 | AA/AG/GG | 285/311/91 | 0.3595 | 139.3/136.05/136.6 | | 27.1/28.9/26.5 | -0.627 | 0.22016 | -0.281 | 0.596 |
| rs5112 | *APOC1P1* | CC/GC/GG | 196/333/171 | 0.4797 | 133.36/139.51/137.98 | | 26.1/29.0/27.0 | 0.805 | 0.09240 | 0.818 | 0.109 |
| rs7259004 | *APOC1P1* | CC/CG/GG | 76/293/360 | 0.3020 | 138.36/138.41/134.76 | | 30.2/25.5/28.9 | 0.829 | 0.10102 | 0.548 | 0.304 |
| HCR2p188/rs35136575 | HCR2 | CC/GC/GG | 533/174/24 | 0.1546 | 135.93/138.83/139.95 | | 27.5/28.4/22.1 | 0.852 | 0.18117 | 0.644 | 0.319 |
| HCR2p286 | HCR2 | AA/AG/GG | 3/63/673 | 0.0457 | 99.8/138.38/136.61 | | 39.6/28.1/27.5 | -0.586 | 0.59403 | -0.662 | 0.550 |
| HCR2p523/rs118004808 | HCR2 | CC/TC | 740/4 | 0.0026 | 136.79/135.78 | | 27.8/23.2 | -0.211 | 0.96328 | 0.060 | 0.990 |
| APOC4p368 | 5’ flanking | TC/TT | 3/754 | 0.0019 | 135.24/136.61 | | 47.2/27.7 | -0.687 | 0.89670 | -0.440 | 0.933 |
| APOC4p637/rs113814026 | 5’ flanking | GG/GT/TT | 690/66/1 | 0.0452 | 136.63/138.36/93.9 | | 27.8/26.0/NA | 0.140 | 0.90262 | 0.070 | 0.952 |
| APOC4p757/rs12721105 | 5’ flanking | GG/GT/TT | 709/55/2 | 0.0376 | 137.13/132.16/137.02 | | 27.9/24.5/52.0 | -1.400 | 0.23935 | -1.529 | 0.204 |
| APOC4p1088 | Intron 1 | GT/TT | 2/734 | 0.0013 | 122.5/136.86 | | 33.6/27.8 | -4.805 | 0.45794 | -4.721 | 0.463 |
| APOC4p1130 | Intron 1 | CT/TT | 1/742 | 0.0007 | | 181.19/136.78 | NA/27.7 | 14.377 | 0.11483 | 14.715 | 0.104 |
| APOC4p1192/rs113745034 | Intron 1 | GA/GG | 17/696 | 0.0124 | | 146.73/136.51 | 24.1/28.1 | 3.445 | 0.12841 | 3.505 | 0.119 |
| APOC4p1325del3 | Intron 1 | WD/WW | 36/704 | 0.0245 | | 136.28/136.88 | 34.2/27.3 | -0.314 | 0.84012 | -0.205 | 0.896 |
| APOC4p1430ins | Intron 1 | II/WI/WW | 1/43/627 | 0.0341 | | 92.72/146.32/135.42 | NA/24.5/28.2 | 2.681 | 0.05543 | 2.775 | 0.047 |
| APOC4p2099/rs111339708 | Intron 1 | GG/GT | 739/21 | 0.0141 | | 136.41/145.3 | 27.9/22.1 | 3.040 | 0.13403 | 3.314 | 0.117 |
| APOC4p2467/rs115225947 | Intron 1 | GA/GG | 21/739 | 0.0141 | | 129.33/136.92 | 28.4/27.8 | -2.522 | 0.21250 | -2.625 | 0.191 |
| APOC4p2559/rs5155 | Intron 1 | CC/CT/TT | 618/134/6 | 0.0986 | | 137.4/134.05/118.2 | 27.6/28.4/21.4 | -1.471 | 0.06609 | -1.541 | 0.062 |
| APOC4p2607/rs5156 | Intron 1 | AG/GG | 18/702 | 0.0129 | | 147.1/136.41 | 23.5/28.1 | 3.604 | 0.10172 | 3.493 | 0.119 |
| APOC4p2623/rs5157 | Intron 1 | CC/CT/TT | 512/215/21 | 0.1723 | | 135.23/140.47/127.95 | 27.9/27.0/25.3 | 0.778 | 0.21466 | 0.728 | 0.250 |
| APOC4p2640/rs5158 | Intron 1 | CC/CT | 725/32 | 0.0213 | | 136.85/133.75 | 27.5/32.3 | -1.115 | 0.49970 | -1.157 | 0.487 |
| APOC4p2678/rs148564866 | Intron 1 | GC/GG | 13/724 | 0.0086 | | 145.03/136.63 | 35.1/27.6 | 2.671 | 0.29564 | 3.279 | 0.198 |
| APOC4p2767/rs127721107 | Intron 1 | GG/GT | 696/37 | 0.0254 | | 136.45/143.13 | 27.6/30.5 | 2.168 | 0.16010 | 2.428 | 0.114 |
| APOC4p3348 | Intron 1 | AG/GG | 1/740 | 0.0007 | | 181.29/136.74 | NA/27.6 | 14.520 | 0.11008 | 11.158 | 0.219 |
| APOC2p75APOC4p3380/rs12721104 | C4-Intron 1 | AA/GA/GG | 13/178/562 | 0.1368 | | 119.74/135.5/137.55 | 23.1/27.2/27.9 | -1.254 | 0.06839 | -1.334 | 0.059 |
| APOC2p194APOC4p3498/rs1132899 | C4-Exon 2 | CC/CT/TT | 440/279/40 | 0.2368 | | 134.91/140.58/133.11 | 27.4/27.5/29.4 | 0.884 | 0.11098 | 0.910 | 0.104 |
| APOC2p228/rs5164 | C4-Exon 2 | AG/GA/GG | 9/1/728 | 0.0066 | | 151.28/103.03/136.67 | 18.4/NA/27.8 | 3.311 | 0.25501 | 2.654 | 0.385 |
| APOC2p288APOC4p3592/rs12691090 | C4-Exon 2 | CC/CT | 697/40 | 0.0272 | | 136.44/142.55 | 27.6/29.2 | 2.001 | 0.17721 | 1.977 | 0.181 |
| APOC2p396APOC4p3700 | C4-Intron 2 | GA/GG | 1/717 | 0.0007 | | 172.07/136.63 | NA/28.0 | 11.509 | 0.21156 | 11.743 | 0.199 |
| APOC2p488APOC4p3792/rs5165 | C4-Intron 2 | GA/GG | 22/713 | 0.0146 | | 132.2/136.93 | 25.4/27.7 | -1.543 | 0.43321 | -1.474 | 0.454 |
| APOC2p623APOC4p3927/rs5167 | C4-Exon 3 | GG/GT/TT | 166/367/224 | 0.4594 | | 134.53/138.92/134.05 | 27.8/27.4/27.8 | 0.192 | 0.67940 | 0.185 | 0.694 |
| APOC2p665APOC4p3969/rs138548797 | C4-Exon 3 | AA/CA | 727/13 | 0.0086 | | 136.97/130.35 | 27.8/18.6 | -2.069 | 0.41707 | -2.366 | 0.368 |
| APOC2p708APOC4p4012 | C4-Exon 3 | GA/GG | 1/737 | 0.0007 | | 108.23/136.83 | NA/27.7 | -9.422 | 0.30174 | -9.299 | 0.304 |
| APOC2p853APOC4p4157/rs10425530 | C4-3' UTR | AA/GA/GG | 7/151/592 | 0.1100 | | 123.03/141.36/135.63 | 37.6/28.3/27.4 | 1.166 | 0.12757 | 1.190 | 0.122 |
| APOC2p1042APOC4p4346/rs12709885 | C4-3’/C2-5’ | AA/TA/TT | 717/24/1 | 0.0178 | | 136.16/156.91/140.36 | 27.5/29.9/NA | 5.947 | 0.00071 | 5.480 | 0.003 |
| APOC2p1187APOC4p4491/rs111782345 | C4-3’/C2-5’ | AG/GG | 25/690 | 0.0178 | | 146.19/136.63 | 22.5/28.0 | 3.226 | 0.08379 | 3.359 | 0.076 |
| APOC2p1229APOC4p4533/rs112698600 | C4-3’/C2-5’ | CC/CT | 713/20 | 0.0140 | | 136.53/145.91 | 27.9/20.9 | 3.197 | 0.12314 | 3.578 | 0.082 |
| APOC2p1275APOC4p4579/rs111356234 | C4-3’/C2-5’ | GA/GG | 51/686 | 0.0352 | | 137.8/136.61 | 30.2/27.6 | 0.349 | 0.79182 | -0.177 | 0.898 |
| APOC2p1357APOC4p4661/rs2288912 | C4-3’/C2-5’ | CC/GC/GG | 51/287/413 | 0.2581 | | 130.59/141.26/134.21 | 27.7/27.0/27.6 | 0.803 | 0.13323 | 1.034 | 0.054 |
| APOC2p1540APOC4p4844/rs75463753 | C2-Intron 1 | AA/GA/GG | 11/130/556 | 0.1079 | | 132.61/138.59/135.96 | 29.5/27.3/28.4 | 0.481 | 0.53840 | 0.937 | 0.236 |
| APOC2p2486/rs9304645 | Intron 1 | AA/GA/GG | 90/368/290 | 0.3655 | | 142.16/135.72/135.49 | 34.5/26.2/26.9 | 0.751 | 0.13555 | 0.401 | 0.437 |
| APOC2p2935/rs11879392 | Intron 1 | CC/GC | 704/20 | 0.0135 | | 136.85/137.68 | 27.6/32.7 | 0.181 | 0.93035 | 0.335 | 0.871 |
| APOC2p3010/rs10419086 | Intron 1 | AA/AG/GG | 542/149/15 | 0.1253 | | 138.46/132.88/136.43 | 28.7/24.9/26.7 | -1.321 | 0.06568 | -1.410 | 0.049 |
| APOC2p3692/rs12721060 | Intron 1 | GT/TT | 21/634 | 0.0172 | | 145.71/136.94 | 20.4/28.2 | 3.013 | 0.14108 | 3.366 | 0.097 |
| APOC2p3778/rs5120 | Intron 1 | AA/AT/TT | 502/232/24 | 0.1845 | | 135.48/138.99/135.74 | 28.3/26.9/28.6 | 0.786 | 0.19964 | 1.064 | 0.085 |
| APOC2p3805/rs7257095 | Intron 1 | CC/CG/GG | 509/212/15 | 0.1649 | | 137.15/135.93/131.19 | 28.0/26.8/28.2 | -0.545 | 0.40568 | -0.236 | 0.725 |
| APOC2p3814/rs10422603 | Intron 1 | GG/GT/TT | 64/307/351 | 0.3008 | | 141.98/137.67/135.22 | 32.6/27.0/27.5 | 0.971 | 0.06515 | 0.744 | 0.164 |
| APOC2p3892/rs5121 | Exon 2 | CC/TC/TT | 675/49/1 | 0.0358 | | 136.66/136.88/142.71 | 28.1/24.5/NA | 0.201 | 0.87741 | 0.196 | 0.886 |
| APOC2p4086/rs114780592 | Intron 2 | GA/GG | 41/699 | 0.0278 | | 141.16/136.52 | 28.7/27.6 | 1.525 | 0.29745 | 1.615 | 0.268 |
| APOC2p4319/rs5123 | Intron 3 | AA/GA/GG | 6/73/649 | 0.0592 | | 138.85/138.09/136.47 | 27.8/29.2/27.6 | 0.492 | 0.61561 | 22.750 | 0.012 |
| APOC2p4513/rs180809422 | Intron 3 | AA/AC/CC | 670/16/1 | 0.0135 | | 136.12/146.66/154.65 | 27.8/22.4/NA | 3.470 | 0.09379 | 0.731 | 0.467 |
| APOC2p4587/rs5126 | Exon 4 | AA/CA/CC | 637/69/1 | 0.0499 | | 136.48/139.92/94.63 | 27.9/27.1/NA | 0.678 | 0.54818 | 4.137 | 0.074 |
| APOC2p4754/rs7253690 | Exon 4 | AA/GA/GG | 6/79/673 | 0.0606 | | 138.53/140.71/136.3 | 27.8/30.2/27.5 | 1.168 | 0.22087 | 0.648 | 0.572 |
| APOC2p4853/rs150448996 | 3'flanking | DD/WD/WW | 386/282/56 | 0.2736 | | 138.01/136.14/131.65 | 28.1/27.0/29.2 | -0.861 | 0.10718 | 1.119 | 0.254 |
| APOC2p4973/rs199828513 | 3'flanking | WI/WW | 12/706 | 0.0082 | | 137.16/137.23 | 11.9/28.1 | 0.158 | 0.95293 | -0.745 | 0.168 |
| APOC2p5004/rs10421404 | 3'flanking | CC/CT/TT | 370/327/57 | 0.2908 | | 135.82/136.31/141.93 | 27.8/26.7/32.4 | 0.611 | 0.24821 | 0.296 | 0.915 |
| APOC2p5018/rs78403558 | 3'flanking | DD/WD/WW | 1/52/710 | 0.0352 | | 137.17/138.07/136.62 | NA/34.0/27.3 | 0.357 | 0.77793 | 0.393 | 0.466 |
| APOC2p5310/rs7258345 | 3'flanking | GG/GT/TT | 327/315/58 | 0.3067 | | 138.01/137.46/134.06 | 29.1/27.2/24.4 | -0.419 | 0.44209 | 0.373 | 0.771 |
| APOC2p5398/rs12709889 | 3'flanking | AA/GA/GG | 49/278/405 | 0.2587 | | 132.9/136/137.54 | 28.8/26.3/28.3 | -0.632 | 0.24337 | -0.327 | 0.553 |
| APOC2p5491 | 3'flanking | CC/TC | 740/1 | 0.0007 | | 136.83/119.12 | 27.8/NA | -5.709 | 0.53224 | -0.445 | 0.415 |
| APOC2p5512/rs12721064 | 3'flanking | CC/CT | 756/13 | 0.0083 | | 136.65/136.54 | 27.9/17.3 | 0.100 | 0.96886 | -5.295 | 0.559 |
| APOC2p5562 | 3'flanking | CG/GG | 24/702 | 0.0175 | | 139.29/136.71 | 39.5/27.4 | 0.647 | 0.73492 | -3.445 | 0.225 |
| APOC2p5586/rs73558127 | 3'flanking | GG/GT/TT | 10/124/589 | 0.1001 | | 146/138.61/136.4 | 29.2/25.2/28.3 | 0.996 | 0.20571 | 0.402 | 0.832 |
| APOC2p5771 | 3'flanking | WD/WW | 7/721 | 0.0047 | | 133.2/136.85 | 20.8/27.7 | -1.103 | 0.74972 | 0.805 | 0.315 |
| APOC2p5815/rs10423208 | 3'flanking | AA/GA/GG | 339/322/72 | 0.3164 | | 137.38/136.77/133.59 | 29.5/27.0/23.8 | -0.426 | 0.41044 | -1.818 | 0.597 |
| APOC2p5922/rs10422888 | 3'flanking | AA/AG/GG | 592/102/5 | 0.0784 | | 136.82/137.93/129.97 | 27.3/30.3/38.3 | 0.052 | 0.95405 | -0.231 | 0.658 |
| APOC2p5965 | 3'flanking | GA/GG | 2/741 | 0.0013 | | 127.8/136.82 | 7.9/27.8 | -2.791 | 0.66578 | 0.293 | 0.749 |
| APOC2p6334 | 3'flanking | GA/GG | 15/749 | 0.0096 | | 130.12/136.79 | 30.5/27.5 | -2.296 | 0.33277 | -2.484 | 0.698 |
| MAF is the minor allele frequency; GT is genotype; GT count is the number of individuals in each genotype group; GT_SD is standard deviation of lipid traits mean in each genotype group; *Adjusted for relevant covariates, **Adjusted for *APOE*2/E*4* SNPs in addition to the covariates. APOC2p4118/rs201709243 is excluded due to missing data. | | | | | | | | | | | |
