# Supplementary material for: Apolipoprotein E-C1-C4-C2 gene cluster region and inter-individual variation in plasma lipoprotein levels: a comprehensive genetic association study in two ethnic groups
Source: PLoS One. 2019 Mar 26;14(3):e0214060. doi: 10.1371/journal.pone.0214060 (PMC6435132; doi:10.1371/journal.pone.0214060)
Supplement: S23 Table — MAF is the minor allele frequency; GT is genotype; GT count is the number of individuals in each genotype group; GT_SD is standard deviation of lipid traits mean in each genotype group; *Adjusted for relevant covariates, **Adjusted for APOE*2/E*4 SNPs in addition to the covariates. APOC2p4118/rs201709243 is excluded due to missing data. (DOCX) [file pone.0214060.s023.docx]

S23 Table. Single-site association analysis results for TG in ABs

| **Variant Name/RefSNP ID** | **Location** | **Genotype** | **GT Count** | **MAF** | **Adjusted Mean of plasma TG*** | **GT_SD*** | **Beta*** | **P*** | **Adj. B.** | **Adj. P** |
| --- | --- | --- | --- | --- | --- | --- | --- | --- | --- | --- |
| APOE73/rs1081101 | 5'flanking | CC/CT/TT | 659/82/5 | 0.0611 | 69.19/76.65/92.6 | 29.8/34.0/55.5 | 0.038 | 0.00927 | 0.037 | 0.013 |
| APOE173 | 5'flanking | AA/GA | 745/2 | 0.0020 | 70.13/73.71 | 30.6/30.4 | 0.037 | 0.70282 | 0.042 | 0.668 |
| APOE308/rs769445 | 5'flanking | CC/TC | 737/10 | 0.0072 | 70.03/77.84 | 30.7/23.3 | 0.056 | 0.20649 | 0.036 | 0.432 |
| APOE560/rs449647 | 5'flanking | AA/AT/TT | 301/335/102 | 0.3663 | 70.73/69.69/70.08 | 31.6/28.9/31.7 | -0.001 | 0.84824 | 0.001 | 0.934 |
| APOE618 | 5'flanking | GC/GG | 1/760 | 0.0006 | 68.05/70.26 | NA/30.5 | 0.025 | 0.85598 | 0.020 | 0.884 |
| APOE624/rs769446 | 5'flanking | TC/TT | 11/687 | 0.0077 | 69.92/70.44 | 27.6/30.6 | 0.005 | 0.90959 | 0.017 | 0.688 |
| APOE832/rs405509 | 5'flanking | GG/GT/TT | 427/268/59 | 0.2561 | 69.98/71.39/68.15 | 31.2/30.0/30.0 | 0.003 | 0.74568 | 0.005 | 0.553 |
| APOE1109/rs9282609 | Splice site | CC/TC/TT | 685/55/4 | 0.0415 | 69.83/73.46/88.67 | 30.5/28.6/66.8 | 0.023 | 0.18141 | 0.020 | 0.237 |
| APOE1163/rs440446 | Intron 1 | CC/CG/GG | 8/126/569 | 0.1004 | 92.59/72.71/69.62 | 59.4/28.8/30.5 | 0.023 | 0.05369 | 0.020 | 0.100 |
| APOE1231 | Intron 1 | GA/GG | 19/727 | 0.0125 | 67.34/70.14 | 38.4/30.4 | -0.030 | 0.34414 | -0.025 | 0.449 |
| APOE1279/rs877973 | Intron 1 | AA/CA/CC | 3/82/661 | 0.0597 | 60.01/64.63/70.92 | 23.2/26.1/31.1 | -0.029 | 0.05132 | -0.038 | 0.013 |
| APOE1539/rs184686013 | Intron 1 | AA/AG/GG | 732/11/1 | 0.0086 | 70.28/66.57/80.4 | 30.7/27.1/NA | 0.002 | 0.95463 | -0.010 | 0.786 |
| APOE2072/rs189660912 | Intron 2 | GA/GG | 12/733 | 0.0079 | 68.98/70.12 | 25.0/30.7 | 0.009 | 0.82286 | 0.009 | 0.829 |
| APOE2269/rs61357706 | Intron 2 | GA/GG | 25/722 | 0.0169 | 79.21/69.84 | 37.5/30.0 | 0.040 | 0.14890 | 0.044 | 0.121 |
| APOE2440/rs769450 | Intron 2 | AA/AG/GG | 107/309/261 | 0.3870 | 69.13/70.46/70.84 | 29.1/30.7/31.3 | -0.003 | 0.73960 | -0.013 | 0.143 |
| APOE3673/rs769453 | Intron 3 | CC/GC | 737/9 | 0.0066 | 70.23/74.25 | 30.7/23.4 | 0.037 | 0.42419 | 0.036 | 0.444 |
| APOE3937/rs429358 | Exon 4 | CC/CT/TT | 56/287/405 | 0.2656 | 67.14/69.52/70.98 | 27.5/30.9/30.7 | -0.008 | 0.30755 |  |  |
| APOE4036/rs769455 | Exon 4 | CC/TC | 709/27 | 0.0200 | 69.45/83.67 | 29.9/42.8 | 0.056 | 0.03718 | 0.059 | 0.035 |
| APOE4075/rs7412 | Exon 4 | AA/GA/GG | 3/85/666 | 0.0605 | 49.67/68.01/70.51 | 16.9/29.1/30.8 | -0.018 | 0.23759 |  |  |
| APOE4569 | 3'UTR | GG/GT | 744/1 | 0.0007 | 70.08/77.54 | 30.6/NA | 0.073 | 0.59764 | 0.076 | 0.585 |
| APOE5223 | 3'flanking | CC/CG | 756/8 | 0.0051 | 70.38/70.64 | 30.7/29.6 | 0.006 | 0.90777 | 0.003 | 0.949 |
| APOE5231 | 3'flanking | GG/GT/TT | 2/35/707 | 0.0270 | 68.24/66.1/70.39 | 17.7/32.2/30.6 | -0.023 | 0.28357 | -0.021 | 0.342 |
| rs439401 | Intergenic | CC/CT/TT | 586/131/14 | 0.1092 | 69.35/71.64/77.45 | 30.2/29.1/48.4 | 0.011 | 0.34010 | 0.011 | 0.357 |
| APOC1rs445925 | Intergenic | AA/GA/GG | 68/309/366 | 0.2990 | 69.27/69.27/71.63 | 30.9/28.9/31.9 | -0.007 | 0.37485 | 0.011 | 0.467 |
| APOC1p720ins4/rs11568822 | 5'flanking | II/WI/WW | 60/281/392 | 0.2737 | 66.67/68.98/71.51 | 27.9/29.7/31.4 | -0.013 | 0.11371 | -0.012 | 0.183 |
| APOC1p894/rs190454394 | 5'flanking | CC/CT | 738/3 | 0.0020 | 70.01/68.41 | 30.5/15.2 | 0.021 | 0.79072 | 0.015 | 0.854 |
| APOC1p1166/rs72654452 | Intron 1 | CC/CT/TT | 714/41/2 | 0.0308 | 69.6/78.54/69.87 | 30.0/39.4/31.8 | 0.028 | 0.16303 | 0.028 | 0.187 |
| APOC1p1331/rs10408994 | Intron 2 | AG/GG | 99/638 | 0.0666 | 73.13/69.81 | 32.7/30.4 | 0.016 | 0.28616 | 0.011 | 0.473 |
| APOC1p1526/rs5114 | Intron 2 | CC/CT/TT | 654/78/3 | 0.0579 | 70.84/65.76/59.97 | 31.1/26.7/23.2 | -0.025 | 0.11175 | -0.033 | 0.036 |
| APOC1p1642 | Intron 2 | CC/CT | 740/16 | 0.0103 | 70.09/69.91 | 30.2/40.5 | -0.019 | 0.58314 | -0.014 | 0.702 |
| APOC1p1684/rs12709881 | Intron 2 | AA/GA/GG | 8/131/614 | 0.0973 | 70.29/66.84/70.55 | 42.3/28.2/30.4 | -0.017 | 0.15409 | -0.022 | 0.067 |
| APOC1p3358 | Intron 3 | AA/GA | 705/3 | 0.0021 | 70.07/64.64 | 30.7/4.6 | 0.003 | 0.97274 | 3.10E-04 | 0.997 |
| APOC1p3423/rs389261 | Intron 3 | AA/GA/GG | 77/319/317 | 0.3310 | 70.17/69.28/71.2 | 29.1/29.3/32.2 | -0.002 | 0.81164 | -0.003 | 0.700 |
| APOC1p3573/rs10424339 | Intron 3 | AA/GA/GG | 16/173/540 | 0.1396 | 83.79/67.92/70.5 | 31.9/30.3/30.7 | 0.001 | 0.90959 | -0.003 | 0.760 |
| APOC1p5006/rs112528434 | Intron 3 | GG/GT/TT | 571/103/6 | 0.0850 | 70.4/66.8/75.44 | 30.7/29.8/48.7 | -0.018 | 0.17321 | -0.025 | 0.067 |
| APOC1p5053/rs12721052 | Intron 3 | DD/WD/WW | 41/248/463 | 0.2200 | 69.77/71.12/69.87 | 29.5/31.2/30.3 | 0.004 | 0.65241 | 0.001 | 0.890 |
| APOC1p5667/rs12721054 | 3'UTR | AA/GA/GG | 506/163/17 | 0.1446 | 72.28/66.04/65.85 | 31.3/27.9/31.8 | -0.028 | 0.00699 | -0.029 | 0.006 |
| APOC1p5926/rs56131196 | 3'flanking | AA/AG/GG | 20/214/493 | 0.1745 | 60.05/68.66/71.08 | 25.7/30.4/30.9 | -0.019 | 0.04423 | -0.021 | 0.036 |
| rs4803770 | Intergenic | CC/GC/GG | 394/276/57 | 0.2695 | 69.84/69.66/74.13 | 30.4/29.8/36.4 | 0.005 | 0.57320 | 0.003 | 0.759 |
| HCR1p424/rs117664574 | HCR1 | AG/GG | 11/731 | 0.0073 | 72.62/70.09 | 24.5/30.7 | 0.025 | 0.55251 | 0.021 | 0.615 |
| HCR1p575/rs157599 | HCR1 | AA/AG/GG | 280/310/90 | 0.3595 | 71.87/68.48/70.03 | 33.0/28.6/29.2 | -0.004 | 0.61322 | -0.004 | 0.666 |
| rs5112 | *APOC1P1* | CC/GC/GG | 199/332/165 | 0.4797 | 68.98/70.65/72.02 | 31.9/29.7/32.1 | 0.010 | 0.18177 | 0.006 | 0.455 |
| rs7259004 | *APOC1P1* | CC/CG/GG | 75/289/366 | 0.3020 | 70.51/70.37/69.79 | 29.6/30.0/31.5 | 0.006 | 0.43279 | 0.004 | 0.597 |
| HCR2p188/rs35136575 | HCR2 | CC/GC/GG | 532/175/25 | 0.1546 | 69.43/71.86/64.97 | 30.2/31.6/26.4 | 0.005 | 0.60400 | 0.001 | 0.932 |
| HCR2p286 | HCR2 | AA/AG/GG | 3/61/676 | 0.0457 | 45.01/77.9/69.6 | 8.3/34.1/30.2 | 0.026 | 0.12920 | 0.027 | 0.113 |
| HCR2p523/rs118004808 | HCR2 | CC/TC | 740/4 | 0.0026 | 70.19/71.25 | 30.7/13.4 | 0.034 | 0.62894 | 0.026 | 0.710 |
| APOC4p368 | 5’ flanking | TC/TT | 3/747 | 0.0019 | 88.72/70.33 | 37.2/30.6 | 0.088 | 0.26413 | 0.085 | 0.281 |
| APOC4p637/rs113814026 | 5’ flanking | GG/GT/TT | 684/68/1 | 0.0452 | 70.59/69.02/44.6 | 30.7/30.9/NA | -0.013 | 0.44570 | -0.015 | 0.403 |
| APOC4p757/rs12721105 | 5’ flanking | GG/GT/TT | 705/54/2 | 0.0376 | 69.34/84.01/72.01 | 30.0/36.9/29.0 | 0.063 | 0.00052 | 0.057 | 0.002 |
| APOC4p1088 | Intron 1 | GT/TT | 2/734 | 0.0013 | 113.92/70.15 | 63.6/30.5 | 0.182 | 0.06327 | 0.178 | 0.070 |
| APOC4p1130 | Intron 1 | CT/TT | 1/742 | 0.0007 | 83.99/70.21 | NA/30.6 | 0.097 | 0.48004 | 0.090 | 0.512 |
| APOC4p1192/rs113745034 | Intron 1 | GA/GG | 18/695 | 0.0124 | 70.29/70.26 | 39.1/30.5 | -0.008 | 0.80150 | -0.008 | 0.801 |
| APOC4p1325del3 | Intron 1 | WD/WW | 36/705 | 0.0245 | 61.96/70.6 | 22.0/30.9 | -0.040 | 0.09089 | -0.038 | 0.114 |
| APOC4p1430ins | Intron 1 | II/WI/WW | 1/44/622 | 0.0341 | 63.94/67.93/70.05 | NA/28.8/30.7 | -0.009 | 0.64588 | -0.010 | 0.634 |
| APOC4p2099/rs111339708 | Intron 1 | GG/GT | 734/21 | 0.0141 | 70.28/66.38 | 30.0/38.0 | -0.033 | 0.27451 | -0.016 | 0.625 |
| APOC4p2467/rs115225947 | Intron 1 | GA/GG | 21/734 | 0.0141 | 62.56/70.49 | 20.4/30.8 | -0.027 | 0.37943 | -0.027 | 0.377 |
| APOC4p2559/rs5155 | Intron 1 | CC/CT/TT | 613/134/6 | 0.0986 | 70.33/69.56/74.26 | 29.8/32.4/41.2 | -0.008 | 0.49082 | -0.008 | 0.549 |
| APOC4p2607/rs5156 | Intron 1 | AG/GG | 18/702 | 0.0129 | 68.08/70.39 | 40.6/30.6 | -0.029 | 0.37912 | -0.013 | 0.704 |
| APOC4p2623/rs5157 | Intron 1 | CC/CT/TT | 507/215/21 | 0.1723 | 70.08/69.51/75.22 | 30.0/29.5/41.4 | 0.001 | 0.94809 | 0.004 | 0.701 |
| APOC4p2640/rs5158 | Intron 1 | CC/CT | 720/32 | 0.0213 | 70.51/59.46 | 30.5/19.6 | -0.052 | 0.03532 | -0.049 | 0.050 |
| APOC4p2678/rs148564866 | Intron 1 | GC/GG | 13/724 | 0.0086 | 77.27/70 | 31.2/30.4 | 0.041 | 0.28796 | 0.043 | 0.268 |
| APOC4p2767/rs127721107 | Intron 1 | GG/GT | 696/37 | 0.0254 | 70.58/68.95 | 30.7/29.5 | -0.007 | 0.75986 | -0.009 | 0.712 |
| APOC4p3348 | Intron 1 | AG/GG | 1/741 | 0.0007 | 35.82/70.21 | NA/30.6 | -0.230 | 0.09471 | -0.222 | 0.110 |
| APOC2p75APOC4p3380/rs12721104 | C4-Intron 1 | AA/GA/GG | 13/177/558 | 0.1368 | 81.44/72.36/69.17 | 46.0/32.2/29.2 | 0.015 | 0.16334 | 0.015 | 0.176 |
| APOC2p194APOC4p3498/rs1132899 | C4-Exon 2 | CC/CT/TT | 435/279/40 | 0.2368 | 70.04/70.4/68.5 | 30.2/30.0/33.0 | -0.001 | 0.87833 | 0.001 | 0.953 |
| APOC2p228/rs5164 | C4-Exon 2 | AG/GA/GG | 9/1/728 | 0.0066 | 78.63/113.47/70.16 | 42.0/NA/30.4 | 0.036 | 0.41379 | 0.024 | 0.601 |
| APOC2p288APOC4p3592/rs12691090 | C4-Exon 2 | CC/CT | 697/40 | 0.0272 | 70.45/68.6 | 30.7/28.8 | -0.008 | 0.72615 | -0.009 | 0.677 |
| APOC2p396APOC4p3700 | C4-Intron 2 | GA/GG | 1/717 | 0.0007 | 40.57/70.3 | NA/30.6 | -0.139 | 0.31613 | -0.143 | 0.303 |
| APOC2p488APOC4p3792/rs5165 | C4-Intron 2 | GA/GG | 22/714 | 0.0146 | 63.29/70.23 | 20.8/30.9 | -0.025 | 0.40589 | -0.021 | 0.482 |
| APOC2p623APOC4p3927/rs5167 | C4-Exon 3 | GG/GT/TT | 164/366/223 | 0.4594 | 70.36/70.44/68.91 | 28.6/30.5/31.1 | 0.007 | 0.34212 | 0.009 | 0.216 |
| APOC2p665APOC4p3969/rs138548797 | C4-Exon 3 | AA/CA | 727/13 | 0.0086 | 70/73.28 | 30.5/41.2 | 0.017 | 0.66857 | 0.019 | 0.630 |
| APOC2p708APOC4p4012 | C4-Exon 3 | GA/GG | 1/737 | 0.0007 | 136.39/70.13 | NA/30.6 | 0.287 | 0.03810 | 0.278 | 0.045 |
| APOC2p853APOC4p4157/rs10425530 | C4-3' UTR | AA/GA/GG | 7/152/587 | 0.1100 | 68.14/70.48/70.05 | 43.0/31.0/29.9 | -0.004 | 0.75183 | -0.002 | 0.882 |
| APOC2p1042APOC4p4346/rs12709885 | C4-3’/C2-5’ | AA/TA/TT | 717/24/1 | 0.0178 | 70.31/66.75/39.45 | 30.5/32.9/NA | -0.035 | 0.18665 | -0.036 | 0.194 |
| APOC2p1187APOC4p4491/rs111782345 | C4-3’/C2-5’ | AG/GG | 25/691 | 0.0178 | 69.46/70.07 | 36.6/30.3 | -0.013 | 0.63757 | -0.004 | 0.887 |
| APOC2p1229APOC4p4533/rs112698600 | C4-3’/C2-5’ | CC/CT | 713/20 | 0.0140 | 70.05/69.98 | 30.2/37.8 | -0.007 | 0.81539 | -0.009 | 0.781 |
| APOC2p1275APOC4p4579/rs111356234 | C4-3’/C2-5’ | GA/GG | 50/687 | 0.0352 | 72.04/70.06 | 33.8/30.4 | 0.006 | 0.74933 | 0.006 | 0.783 |
| APOC2p1357APOC4p4661/rs2288912 | C4-3’/C2-5’ | CC/GC/GG | 50/285/411 | 0.2581 | 65.22/70.65/70.21 | 30.3/31.5/30.0 | -0.007 | 0.36144 | -0.006 | 0.467 |
| APOC2p1540APOC4p4844/rs75463753 | C2-Intron 1 | AA/GA/GG | 10/131/555 | 0.1079 | 75.36/68.12/70.53 | 26.4/30.9/30.3 | -0.008 | 0.50004 | -0.008 | 0.491 |
| APOC2p2486/rs9304645 | Intron 1 | AA/GA/GG | 87/368/289 | 0.3655 | 74.7/70.34/68.75 | 32.0/31.3/28.9 | 0.011 | 0.14059 | 0.011 | 0.149 |
| APOC2p2935/rs11879392 | Intron 1 | CC/GC | 704/20 | 0.0135 | 70.68/56.3 | 31.0/15.3 | -0.069 | 0.02851 | -0.071 | 0.024 |
| APOC2p3010/rs10419086 | Intron 1 | AA/AG/GG | 539/149/14 | 0.1253 | 70.9/64.67/80.16 | 30.9/24.1/47.0 | -0.017 | 0.12716 | -0.015 | 0.179 |
| APOC2p3692/rs12721060 | Intron 1 | GT/TT | 22/634 | 0.0172 | 67.99/70.4 | 36.3/30.8 | -0.015 | 0.62291 | -0.018 | 0.563 |
| APOC2p3778/rs5120 | Intron 1 | AA/AT/TT | 500/230/23 | 0.1845 | 70.55/69.54/73.52 | 30.5/29.8/37.5 | 7.31E-05 | 0.99372 | 0.002 | 0.806 |
| APOC2p3805/rs7257095 | Intron 1 | CC/CG/GG | 510/213/14 | 0.1649 | 70.52/69.28/62.61 | 32.0/27.7/21.0 | -0.003 | 0.74650 | -0.002 | 0.824 |
| APOC2p3814/rs10422603 | Intron 1 | GG/GT/TT | 63/311/349 | 0.3008 | 73.55/70.31/68.57 | 31.9/30.5/29.8 | 0.012 | 0.14544 | 0.011 | 0.167 |
| APOC2p3892/rs5121 | Exon 2 | CC/TC/TT | 675/50/1 | 0.0358 | 70.57/68.38/86.62 | 31.2/26.6/NA | 0.004 | 0.84160 | -0.002 | 0.928 |
| APOC2p4086/rs114780592 | Intron 2 | GA/GG | 41/699 | 0.0278 | 68.73/70.31 | 28.2/30.8 | -0.004 | 0.84856 | -0.006 | 0.787 |
| APOC2p4319/rs5123 | Intron 3 | AA/GA/GG | 6/74/649 | 0.0592 | 79.96/66.14/70.1 | 30.0/28.4/30.6 | -0.008 | 0.60254 | -0.008 | 0.589 |
| APOC2p4513/rs180809422 | Intron 3 | AA/AC/CC | 667/16/1 | 0.0135 | 69.7/74.06/39.14 | 30.2/40.3/NA | -0.021 | 0.50114 | 0.010 | 0.777 |
| APOC2p4587/rs5126 | Exon 4 | AA/CA/CC | 636/70/1 | 0.0499 | 70.44/69.87/43.72 | 30.8/32.5/NA | -0.011 | 0.51928 | -0.014 | 0.419 |
| APOC2p4754/rs7253690 | Exon 4 | AA/GA/GG | 6/78/669 | 0.0606 | 80.33/67.52/70.67 | 30.0/28.0/30.9 | -0.004 | 0.78701 | -0.004 | 0.812 |
| APOC2p4853/rs150448996 | 3'flanking | DD/WD/WW | 383/288/55 | 0.2736 | 70.45/70.09/69.45 | 32.4/28.1/31.5 | 0.001 | 0.90826 | -0.001 | 0.924 |
| APOC2p4973/rs199828513 | 3'flanking | WI/WW | 12/701 | 0.0082 | 76.49/70.11 | 46.3/30.5 | 0.010 | 0.80530 | -0.006 | 0.892 |
| APOC2p5004/rs10421404 | 3'flanking | CC/CT/TT | 368/326/56 | 0.2908 | 69.07/71.11/74.63 | 29.8/31.4/32.8 | 0.011 | 0.15992 | 0.011 | 0.173 |
| APOC2p5018/rs78403558 | 3'flanking | DD/WD/WW | 1/51/706 | 0.0352 | 63.63/64.03/70.79 | NA/21.8/31.1 | -0.024 | 0.21115 | -0.025 | 0.200 |
| APOC2p5310/rs7258345 | 3'flanking | GG/GT/TT | 324/312/56 | 0.3067 | 70.87/69.31/68.65 | 29.8/31.4/33.4 | -0.010 | 0.21601 | -0.008 | 0.342 |
| APOC2p5398/rs12709889 | 3'flanking | AA/GA/GG | 48/283/401 | 0.2587 | 66.62/70.44/70.46 | 28.7/28.3/32.4 | -0.001 | 0.92941 | -0.001 | 0.908 |
| APOC2p5491 | 3'flanking | CC/TC | 740/1 | 0.0007 | 70.01/55.11 | 30.5/NA | -0.040 | 0.77499 | -0.037 | 0.790 |
| APOC2p5512/rs12721064 | 3'flanking | CC/CT | 752/12 | 0.0083 | 70.02/87.21 | 30.5/33.9 | 0.087 | 0.02969 | 0.114 | 0.011 |
| APOC2p5562 | 3'flanking | CG/GG | 24/703 | 0.0175 | 67.09/70.25 | 31.2/30.5 | -0.015 | 0.61087 | -0.016 | 0.572 |
| APOC2p5586/rs73558127 | 3'flanking | GG/GT/TT | 10/125/589 | 0.1001 | 80.41/67.13/70.25 | 39.2/30.0/29.9 | -0.008 | 0.48499 | -0.007 | 0.549 |
| APOC2p5771 | 3'flanking | WD/WW | 7/721 | 0.0047 | 78.25/70.26 | 39.4/30.4 | 0.031 | 0.55408 | 0.033 | 0.529 |
| APOC2p5815/rs10423208 | 3'flanking | AA/GA/GG | 340/322/71 | 0.3164 | 70.84/70.33/67.09 | 29.8/31.1/32.8 | -0.010 | 0.20297 | -0.008 | 0.314 |
| APOC2p5922/rs10422888 | 3'flanking | AA/AG/GG | 594/101/5 | 0.0784 | 69.37/72.64/72.93 | 30.1/31.3/31.5 | 0.015 | 0.25815 | 0.012 | 0.398 |
| APOC2p5965 | 3'flanking | GA/GG | 2/741 | 0.0013 | 61.69/70.14 | 10.6/30.6 | -0.006 | 0.95121 | -0.006 | 0.949 |
| APOC2p6334 | 3'flanking | GA/GG | 15/744 | 0.0096 | 73.45/70.12 | 28.7/30.6 | 0.014 | 0.68863 | 0.011 | 0.752 |
| MAF is the minor allele frequency; GT is genotype; GT count is the number of individuals in each genotype group; GT_SD is standard deviation of lipid traits mean in each genotype group; *Adjusted for relevant covariates, **Adjusted for *APOE*2/E*4* SNPs in addition to the covariates. APOC2p4118/rs201709243 is excluded due to missing data | | | | | | | | | | |
