# Supplementary material for: Apolipoprotein E-C1-C4-C2 gene cluster region and inter-individual variation in plasma lipoprotein levels: a comprehensive genetic association study in two ethnic groups
Source: PLoS One. 2019 Mar 26;14(3):e0214060. doi: 10.1371/journal.pone.0214060 (PMC6435132; doi:10.1371/journal.pone.0214060)
Supplement: S24 Table — hap.freq: haplotype frequency; coef: coefficient; se: standard error; t.stat: test statistic; p-val: haplotype p-value. (DOCX) [file pone.0214060.s024.docx]

S24 Table. Haplotype summary of significant association with LDL-C in NHWs

| **LDL-C** | | | | | | | | | | |
| --- | --- | --- | --- | --- | --- | --- | --- | --- | --- | --- |
|  | **Window** | **loc.1** | **loc.2** | **loc.3** | **loc.4** | **hap.freq** | **coef** | **se** | **t.stat** | **pval** |
| Geno.3 | 1 | A | T | C | C | 0.2591 | -6.76 | 3.01 | -2.25 | 0.02486 |
| Geno.4 | 1 | A | T | C | T | 0.0211 | -1.35 | 7.63 | -0.18 | 0.85966 |
| Geno.5 | 1 | A | T | G | C | 0.1182 | 3.48 | 3.70 | 0.94 | 0.34709 |
| Geno.7 | 1 | T | G | G | C | 0.0814 | -18.23 | 4.88 | -3.74 | 0.00020 |
| Geno.8 | 1 | T | T | C | C | 0.0783 | 0.97 | 4.57 | 0.21 | 0.83201 |
| Geno.rare | 1 | * | * | * | * | 0.0027 | -18.70 | 0.03 | -615.99 | <10E-06 |
| haplo.base | 1 | A | G | G | C | 0.4391 | NA | NA | NA | NA |
| Geno.2 | 3 | C | C | G | G | 0.3374 | -4.93 | 2.65 | -1.86 | 0.06304 |
| Geno.31 | 3 | C | T | G | G | 0.0211 | -1.67 | 7.67 | -0.22 | 0.82803 |
| Geno.52 | 3 | G | C | A | G | 0.1150 | 2.98 | 3.79 | 0.79 | 0.43109 |
| Geno.72 | 3 | G | C | G | G | 0.1221 | -13.46 | 3.69 | -3.65 | 0.00029 |
| Geno.rare2 | 3 | * | * | * | * | 0.0019 | -10.87 | 0.09 | -124.49 | <10E-06 |
| haplo.base2 | 3 | G | C | G | A | 0.4026 | NA | NA | NA | NA |
| Geno.32 | 4 | C | A | G | T | 0.1150 | 10.18 | 3.78 | 2.69 | 0.00724 |
| Geno.53 | 4 | C | G | A | T | 0.4019 | 7.14 | 2.48 | 2.87 | 0.00419 |
| Geno.9 | 4 | T | G | G | T | 0.0210 | 5.04 | 7.73 | 0.65 | 0.51461 |
| Geno.rare3 | 4 | * | * | * | * | 0.0112 | 3.41 | 10.84 | 0.31 | 0.75341 |
| haplo.base3 | 4 | C | G | G | T | 0.4508 | NA | NA | NA | NA |
| Geno.33 | 5 | A | G | T | G | 0.1147 | 9.92 | 3.76 | 2.64 | 0.00850 |
| Geno.61 | 5 | G | A | T | G | 0.4002 | 6.75 | 2.44 | 2.76 | 0.00586 |
| Geno.rare4 | 5 | * | * | * | * | 0.0129 | 6.44 | 0.24 | 26.92 | <10E-06 |
| haplo.base4 | 5 | G | G | T | G | 0.4722 | NA | NA | NA | NA |
| Geno.62 | 7 | T | G | T | C | 0.1489 | 8.17 | 3.18 | 2.57 | 0.01050 |
| Geno.rare6 | 7 | * | * | * | * | 0.0137 | 6.30 | 0.11 | 55.65 | <10E-06 |
| haplo.base6 | 7 | T | G | T | T | 0.8375 | NA | NA | NA | NA |
| Geno.35 | 8 | G | T | C | C | 0.1490 | 5.03 | 3.20 | 1.57 | 0.11629 |
| Geno.63 | 8 | G | T | T | T | 0.0781 | -22.79 | 4.35 | -5.24 | 2.26E-07 |
| Geno.rare7 | 8 | * | * | * | * | 0.0048 | 40.70 | 0.05 | 853.88 | <10E-06 |
| haplo.base7 | 8 | G | T | T | C | 0.7680 | NA | NA | NA | NA |
| Geno.36 | 9 | T | C | C | T | 0.1514 | 5.97 | 3.12 | 1.92 | 0.05594 |
| Geno.73 | 9 | T | T | T | T | 0.0805 | -20.88 | 4.13 | -5.06 | 5.62E-07 |
| Geno.rare8 | 9 | * | * | * | * | 0.0048 | -26.44 | 0.06 | -427.62 | <10E-06 |
| haplo.base8 | 9 | T | T | C | T | 0.7632 | NA | NA | NA | NA |
| Geno.21 | 10 | C | C | T | C | 0.1522 | 6.01 | 3.11 | 1.93 | 0.05361 |
| Geno.74 | 10 | T | T | T | C | 0.0805 | -20.91 | 4.12 | -5.07 | 5.27E-07 |
| Geno.rare9 | 10 | * | * | * | * | 0.0048 | -31.49 | 0.06 | -507.97 | <10E-06 |
| haplo.base9 | 10 | T | C | T | C | 0.7624 | NA | NA | NA | NA |
| Geno.54 | 11 | T | T | C | C | 0.0805 | -21.80 | 4.12 | -5.29 | 1.68E-07 |
| Geno.rare10 | 11 | * | * | * | * | 0.0129 | -6.08 | 10.20 | -0.60 | 0.55144 |
| haplo.base10 | 11 | C | T | C | C | 0.9066 | NA | NA | NA | NA |
| Geno.22 | 14 | C | C | C | G | 0.0810 | 0.41 | 4.31 | 0.09 | 0.92476 |
| Geno.43 | 14 | C | T | C | A | 0.1101 | -15.73 | 3.90 | -4.03 | 6.17E-05 |
| Geno.64 | 14 | C | T | T | G | 0.3559 | -5.13 | 2.56 | -2.01 | 0.04537 |
| Geno.rare13 | 14 | * | * | * | * | 0.0095 | 8.41 | 0.33 | 25.81 | <10E-06 |
| haplo.base13 | 14 | C | T | C | G | 0.4435 | NA | NA | NA | NA |
| Geno.23 | 15 | C | C | G | C | 0.0838 | 0.06 | 4.25 | 0.01 | 0.98920 |
| Geno.44 | 15 | T | C | A | C | 0.1101 | -16.16 | 3.91 | -4.13 | 4.10E-05 |
| Geno.81 | 15 | T | T | G | C | 0.3579 | -5.12 | 2.54 | -2.01 | 0.04453 |
| Geno.rare14 | 15 | * | * | * | * | 0.0055 | -14.66 | 0.16 | -90.88 | <10E-06 |
| haplo.base14 | 15 | T | C | G | C | 0.4428 | NA | NA | NA | NA |
| Geno.11 | 16 | C | A | C | C | 0.1094 | -16.12 | 3.86 | -4.17 | 3.45E-05 |
| Geno.65 | 16 | T | G | C | C | 0.3602 | -5.19 | 2.44 | -2.13 | 0.03336 |
| Geno.rare15 | 16 | * | * | * | * | 0.0048 | -14.78 | 0.09 | -172.37 | <10E-06 |
| haplo.base15 | 16 | C | G | C | C | 0.5255 | NA | NA | NA | NA |
| Geno.12 | 17 | A | C | C | I | 0.1086 | -13.12 | 3.66 | -3.58 | 0.00037 |
| Geno.66 | 17 | G | C | C | I | 0.1210 | 4.10 | 3.46 | 1.18 | 0.23656 |
| Geno.rare16 | 17 | * | * | * | * | 0.0058 | -12.94 | 0.08 | -161.31 | <10E-06 |
| haplo.base16 | 17 | G | C | C | W | 0.7646 | NA | NA | NA | NA |
| Geno.46 | 29 | G | C | A | T | 0.1483 | 8.80 | 3.25 | 2.71 | 0.00700 |
| Geno.56 | 29 | G | C | G | G | 0.0354 | 18.27 | 6.44 | 2.84 | 0.00471 |
| Geno.rare28 | 29 | * | * | * | * | 0.0074 | -4.63 | 0.36 | -12.75 | <10E-06 |
| haplo.base28 | 29 | G | C | G | T | 0.8088 | NA | NA | NA | NA |
| Geno.26 | 30 | C | A | T | G | 0.1522 | 7.41 | 3.17 | 2.34 | 0.01968 |
| Geno.311 | 30 | C | G | G | G | 0.0388 | 12.99 | 5.81 | 2.24 | 0.02557 |
| Geno.rare29 | 30 | * | * | * | * | 0.0024 | 48.33 | 0.03 | 1478.96 | <10E-06 |
| haplo.base29 | 30 | C | G | T | G | 0.8066 | NA | NA | NA | NA |
| Geno.27 | 31 | A | T | G | A | 0.1519 | 9.03 | 3.17 | 2.85 | 0.00457 |
| Geno.47 | 31 | G | G | G | G | 0.0363 | 17.96 | 6.13 | 2.93 | 0.00351 |
| Geno.68 | 31 | G | T | G | A | 0.0349 | 6.75 | 6.29 | 1.07 | 0.28422 |
| Geno.rare30 | 31 | * | * | * | * | 0.0033 | -44.40 | 0.24 | -184.85 | <10E-06 |
| haplo.base30 | 31 | G | T | G | G | 0.7737 | NA | NA | NA | NA |
| Geno.28 | 32 | G | G | G | A | 0.0376 | 12.34 | 6.17 | 2.00 | 0.04586 |
| Geno.48 | 32 | T | G | A | G | 0.1883 | 7.13 | 2.93 | 2.43 | 0.01529 |
| Geno.rare31 | 32 | * | * | * | * | 0.0037 | 36.13 | 0.31 | 116.64 | <10E-06 |
| haplo.base31 | 32 | T | G | G | A | 0.7704 | NA | NA | NA | NA |
| Geno.312 | 33 | G | A | G | C | 0.1850 | 11.78 | 3.20 | 3.68 | 0.00025 |
| Geno.69 | 33 | G | G | A | G | 0.3723 | 9.14 | 2.61 | 3.50 | 0.00050 |
| Geno.rare32 | 33 | * | * | * | * | 0.0070 | 13.80 | 0.03 | 487.26 | <10E-06 |
| haplo.base32 | 33 | G | G | A | C | 0.4358 | NA | NA | NA | NA |
| Geno.13 | 34 | A | G | C | C | 0.1851 | 11.71 | 3.20 | 3.66 | 0.00028 |
| Geno.610 | 34 | G | A | G | C | 0.3480 | 9.11 | 2.68 | 3.40 | 0.00072 |
| Geno.75 | 34 | G | A | G | G | 0.0246 | 8.66 | 7.15 | 1.21 | 0.22644 |
| Geno.rare33 | 34 | * | * | * | * | 0.0061 | 16.42 | 0.05 | 303.71 | <10E-06 |
| haplo.base33 | 34 | G | A | C | C | 0.4363 | NA | NA | NA | NA |
| Geno.49 | 35 | A | G | C | C | 0.3457 | 8.75 | 2.66 | 3.29 | 0.00107 |
| Geno.611 | 35 | A | G | G | C | 0.0239 | 7.87 | 7.26 | 1.08 | 0.27866 |
| Geno.76 | 35 | G | C | C | C | 0.1550 | 10.92 | 3.39 | 3.22 | 0.00134 |
| Geno.rare34 | 35 | * | * | * | * | 0.0081 | 9.03 | 0.16 | 55.45 | <10E-06 |
| haplo.base34 | 35 | A | C | C | C | 0.4674 | NA | NA | NA | NA |
| Geno.314 | 40 | A | T | G | C | 0.4225 | 8.26 | 2.54 | 3.25 | 0.00120 |
| Geno.412 | 40 | A | T | G | G | 0.1152 | -6.88 | 3.71 | -1.85 | 0.06432 |
| Geno.rare39 | 40 | * | * | * | * | 0.0038 | -8.13 | 0.07 | -114.06 | <10E-06 |
| haplo.base39 | 40 | A | T | C | C | 0.4584 | NA | NA | NA | NA |
| Geno.315 | 41 | T | C | C | C | 0.3218 | -5.88 | 3.17 | -1.86 | 0.06353 |
| Geno.413 | 41 | T | C | C | G | 0.1359 | -11.56 | 3.80 | -3.04 | 0.00249 |
| Geno.83 | 41 | T | G | C | G | 0.0645 | 3.46 | 5.80 | 0.60 | 0.55073 |
| Geno.91 | 41 | T | G | G | C | 0.0894 | -14.42 | 4.54 | -3.17 | 0.00158 |
| Geno.10 | 41 | T | G | G | G | 0.0259 | -15.84 | 8.35 | -1.90 | 0.05835 |
| Geno.rare40 | 41 | * | * | * | * | 0.0037 | -15.60 | 0.17 | -93.81 | <10E-06 |
| haplo.base40 | 41 | T | G | C | C | 0.3587 | NA | NA | NA | NA |
| Geno.15 | 42 | C | C | C | C | 0.3241 | -6.11 | 3.16 | -1.93 | 0.05371 |
| Geno.316 | 42 | C | C | G | C | 0.1304 | -12.23 | 3.91 | -3.13 | 0.00184 |
| Geno.77 | 42 | G | C | G | C | 0.0661 | 3.75 | 5.75 | 0.65 | 0.51499 |
| Geno.84 | 42 | G | G | C | C | 0.0912 | -14.70 | 4.51 | -3.26 | 0.00118 |
| Geno.92 | 42 | G | G | G | C | 0.0256 | -16.28 | 8.48 | -1.92 | 0.05521 |
| Geno.rare41 | 42 | * | * | * | * | 0.0055 | 1.95 | 0.28 | 7.05 | 4.95E-12 |
| haplo.base41 | 42 | G | C | C | C | 0.3572 | NA | NA | NA | NA |
| Geno.29 | 43 | C | C | C | T | 0.0130 | 17.22 | 11.08 | 1.55 | 0.12054 |
| Geno.414 | 43 | C | G | C | C | 0.1930 | -2.97 | 3.02 | -0.98 | 0.32675 |
| Geno.613 | 43 | G | C | C | C | 0.0886 | -10.16 | 4.43 | -2.29 | 0.02213 |
| Geno.85 | 43 | G | G | C | C | 0.0249 | -10.90 | 8.38 | -1.30 | 0.19376 |
| Geno.rare42 | 43 | * | * | * | * | 0.0137 | -13.67 | 10.84 | -1.26 | 0.20771 |
| haplo.base42 | 43 | C | C | C | C | 0.6668 | NA | NA | NA | NA |
| Geno.317 | 46 | C | C | G | A | 0.0362 | -1.31 | 6.13 | -0.21 | 0.83047 |
| Geno.59 | 46 | T | C | A | A | 0.0226 | 3.78 | 7.79 | 0.49 | 0.62766 |
| Geno.rare45 | 46 | * | * | * | * | 0.0025 | 67.19 | 0.04 | 1511.14 | <10E-06 |
| haplo.base45 | 46 | C | C | A | A | 0.9387 | NA | NA | NA | NA |
| Geno.417 | 47 | C | G | A | G | 0.0362 | -1.43 | 6.12 | -0.23 | 0.81513 |
| Geno.rare46 | 47 | * | * | * | * | 0.0041 | 47.71 | 0.05 | 901.97 | <10E-06 |
| haplo.base46 | 47 | C | A | A | G | 0.9597 | NA | NA | NA | NA |
| Geno.16 | 54 | A | G | C | G | 0.0224 | -20.88 | 7.53 | -2.77 | 0.00576 |
| Geno.620 | 54 | G | G | T | G | 0.4791 | -3.14 | 2.28 | -1.38 | 0.16831 |
| Geno.rare53 | 54 | * | * | * | * | 0.0113 | -0.45 | 0.51 | -0.87 | 0.38446 |
| haplo.base53 | 54 | G | G | C | G | 0.4872 | NA | NA | NA | NA |
| Geno.422 | 60 | G | G | A | G | 0.0371 | -0.47 | 6.16 | -0.08 | 0.93870 |
| Geno.517 | 60 | G | G | G | G | 0.4548 | 0.45 | 2.29 | 0.20 | 0.84486 |
| Geno.rare58 | 60 | * | * | * | * | 0.0048 | 53.51 | 0.16 | 343.07 | <10E-06 |
| haplo.base59 | 60 | C | G | G | G | 0.5033 | NA | NA | NA | NA |
| Geno.320 | 61 | G | A | G | G | 0.0372 | -1.28 | 6.06 | -0.21 | 0.83302 |
| Geno.423 | 61 | G | G | G | A | 0.0363 | -7.79 | 6.12 | -1.27 | 0.20308 |
| Geno.rare59 | 61 | * | * | * | * | 0.0048 | 52.71 | 0.09 | 598.53 | <10E-06 |
| haplo.base60 | 61 | G | G | G | G | 0.9217 | NA | NA | NA | NA |
| Geno.18 | 62 | A | G | G | A | 0.0372 | -0.52 | 6.16 | -0.08 | 0.93272 |
| Geno.212 | 62 | G | G | A | A | 0.0372 | -5.86 | 6.15 | -0.95 | 0.34095 |
| Geno.321 | 62 | G | G | G | A | 0.4196 | 1.50 | 2.33 | 0.65 | 0.51914 |
| Geno.rare60 | 62 | * | * | * | * | 0.0040 | 53.39 | 0.19 | 286.58 | <10E-06 |
| haplo.base61 | 62 | G | G | G | T | 0.5020 | NA | NA | NA | NA |
| Geno.19 | 63 | G | A | A | D | 0.0374 | -5.71 | 6.12 | -0.93 | 0.35098 |
| Geno.213 | 63 | G | G | A | D | 0.1823 | -1.76 | 2.98 | -0.59 | 0.55596 |
| Geno.322 | 63 | G | G | A | W | 0.2760 | 3.62 | 2.72 | 1.33 | 0.18266 |
| Geno.rare61 | 63 | * | * | * | * | 0.0061 | 40.97 | 0.20 | 209.25 | <10E-06 |
| haplo.base62 | 63 | G | G | T | D | 0.4983 | NA | NA | NA | NA |

hap.freq: haplotype frequency; coef: coefficient; se: standard error; t.stat: test statistic; p-val: haplotype p-value
