# Supplementary material for: Apolipoprotein E-C1-C4-C2 gene cluster region and inter-individual variation in plasma lipoprotein levels: a comprehensive genetic association study in two ethnic groups
Source: PLoS One. 2019 Mar 26;14(3):e0214060. doi: 10.1371/journal.pone.0214060 (PMC6435132; doi:10.1371/journal.pone.0214060)
Supplement: S25 Table — hap.freq: haplotype frequency; coef: coefficient; se: standard error; t.stat: test statistic; p-val: haplotype p-value. (DOCX) [file pone.0214060.s025.docx]

S25 Table. Haplotype summary of significant association with TC in NHWs

| TC | | | | | | | | | | |
| --- | --- | --- | --- | --- | --- | --- | --- | --- | --- | --- |
|  | Window | loc.1 | loc.2 | loc.3 | loc.4 | hap.freq | coef | se | t.stat | pval |
| Geno.3 | 1 | A | T | C | C | 0.25629 | -10.8 | 3.0 | -3.6 | 0.00033 |
| Geno.4 | 1 | A | T | C | T | 0.02109 | 0.0 | 7.7 | 0.0 | 0.99575 |
| Geno.5 | 1 | A | T | G | C | 0.11946 | 4.0 | 3.7 | 1.1 | 0.27585 |
| Geno.7 | 1 | T | G | G | C | 0.08039 | -22.5 | 4.8 | -4.7 | 3.62E-06 |
| Geno.8 | 1 | T | T | C | C | 0.07957 | 1.5 | 4.5 | 0.3 | 0.74659 |
| Geno.rare | 1 | * | * | * | * | 0.00460 | 95.8 | 0.2 | 605.0 | <10E-06 |
| haplo.base | 1 | A | G | G | C | 0.43861 | NA | NA | NA | NA |
| Geno.2 | 3 | C | C | G | G | 0.33737 | -7.4 | 2.7 | -2.7 | 0.00695 |
| Geno.31 | 3 | C | T | G | G | 0.02111 | -1.6 | 8.0 | -0.2 | 0.84270 |
| Geno.52 | 3 | G | C | A | G | 0.11487 | 2.8 | 3.9 | 0.7 | 0.48050 |
| Geno.72 | 3 | G | C | G | G | 0.12204 | -16.6 | 3.8 | -4.3 | 1.67E-05 |
| Geno.rare2 | 3 | * | * | * | * | 0.00187 | -8.9 | 0.1 | -99.9 | <10E-06 |
| haplo.base2 | 3 | G | C | G | A | 0.40274 | NA | NA | NA | NA |
| Geno.32 | 4 | C | A | G | T | 0.11494 | 12.7 | 3.9 | 3.2 | 0.00123 |
| Geno.53 | 4 | C | G | A | T | 0.40207 | 9.7 | 2.6 | 3.8 | 0.00018 |
| Geno.9 | 4 | T | G | G | T | 0.02106 | 7.7 | 8.0 | 1.0 | 0.34050 |
| Geno.rare3 | 4 | * | * | * | * | 0.01124 | 2.1 | 11.3 | 0.2 | 0.85094 |
| haplo.base3 | 4 | C | G | G | T | 0.45069 | NA | NA | NA | NA |
| Geno.33 | 5 | A | G | T | G | 0.11466 | 12.4 | 3.9 | 3.2 | 0.00163 |
| Geno.61 | 5 | G | A | T | G | 0.40036 | 9.2 | 2.5 | 3.6 | 0.00032 |
| Geno.rare4 | 5 | * | * | * | * | 0.01286 | 6.1 | 0.2 | 24.4 | <10E-06 |
| haplo.base4 | 5 | G | G | T | G | 0.47213 | NA | NA | NA | NA |
| Geno.34 | 6 | A | T | G | T | 0.39996 | 6.8 | 2.4 | 2.8 | 0.00595 |
| Geno.41 | 6 | G | G | G | T | 0.01124 | 0.8 | 0.1 | 5.5 | 5.31E-08 |
| Geno.rare5 | 6 | * | * | * | * | 0.00243 | 30.2 | 0.004 | 8221.1 | <10E-06 |
| haplo.base5 | 6 | G | T | G | T | 0.58638 | NA | NA | NA | NA |
| Geno.35 | 8 | G | T | C | C | 0.14929 | 3.8 | 3.3 | 1.2 | 0.24448 |
| Geno.63 | 8 | G | T | T | T | 0.07762 | -22.1 | 4.5 | -4.9 | 9.68E-07 |
| Geno.rare7 | 8 | * | * | * | * | 0.00539 | 59.9 | 0.1 | 705.3 | <10E-06 |
| haplo.base7 | 8 | G | T | T | C | 0.76771 | NA | NA | NA | NA |
| Geno.36 | 9 | T | C | C | T | 0.15153 | 4.6 | 3.3 | 1.4 | 0.16872 |
| Geno.73 | 9 | T | T | T | T | 0.08059 | -18.9 | 4.3 | -4.4 | 1.54E-05 |
| Geno.rare8 | 9 | * | * | * | * | 0.00482 | -37.6 | 0.1 | -576.2 | <10E-06 |
| haplo.base8 | 9 | T | T | C | T | 0.76306 | NA | NA | NA | NA |
| Geno.21 | 10 | C | C | T | C | 0.15235 | 4.7 | 3.3 | 1.4 | 0.15737 |
| Geno.74 | 10 | T | T | T | C | 0.08059 | -18.8 | 4.3 | -4.3 | 1.66E-05 |
| Geno.rare9 | 10 | * | * | * | * | 0.00482 | -35.0 | 0.1 | -534.4 | <10E-06 |
| haplo.base9 | 10 | T | C | T | C | 0.76224 | NA | NA | NA | NA |
| Geno.54 | 11 | T | T | C | C | 0.08057 | -19.3 | 4.3 | -4.5 | 9.95E-06 |
| Geno.rare10 | 11 | * | * | * | * | 0.01289 | -1.1 | 10.7 | -0.1 | 0.91464 |
| haplo.base10 | 11 | C | T | C | C | 0.90654 | NA | NA | NA | NA |
| Geno.22 | 14 | C | C | C | G | 0.08107 | 0.2 | 4.5 | 0.1 | 0.95933 |
| Geno.43 | 14 | C | T | C | A | 0.10936 | -16.6 | 4.0 | -4.1 | 3.94E-05 |
| Geno.64 | 14 | C | T | T | G | 0.35562 | -6.5 | 2.7 | -2.4 | 0.01504 |
| Geno.rare13 | 14 | * | * | * | * | 0.00951 | 15.2 | 0.3 | 46.3 | <10E-06 |
| haplo.base13 | 14 | C | T | C | G | 0.44444 | NA | NA | NA | NA |
| Geno.23 | 15 | C | C | G | C | 0.08378 | 0.1 | 4.4 | 0.0 | 0.97417 |
| Geno.44 | 15 | T | C | A | C | 0.10937 | -17.2 | 4.0 | -4.3 | 2.18E-05 |
| Geno.81 | 15 | T | T | G | C | 0.35785 | -6.4 | 2.6 | -2.4 | 0.01636 |
| Geno.rare14 | 15 | * | * | * | * | 0.00547 | -24.2 | 0.2 | -142.4 | <10E-06 |
| haplo.base14 | 15 | T | C | G | C | 0.44353 | NA | NA | NA | NA |
| Geno.11 | 16 | C | A | C | C | 0.10871 | -17.5 | 4.0 | -4.4 | 1.17E-05 |
| Geno.65 | 16 | T | G | C | C | 0.36018 | -6.5 | 2.5 | -2.6 | 0.01054 |
| Geno.rare15 | 16 | * | * | * | * | 0.00485 | -16.4 | 0.1 | -192.5 | <10E-06 |
| haplo.base15 | 16 | C | G | C | C | 0.52626 | NA | NA | NA | NA |
| Geno.12 | 17 | A | C | C | I | 0.10807 | -14.2 | 3.8 | -3.7 | 0.00022 |
| Geno.66 | 17 | G | C | C | I | 0.12157 | 3.3 | 3.6 | 0.9 | 0.35675 |
| Geno.rare16 | 17 | * | * | * | * | 0.00577 | -11.1 | 0.1 | -132.4 | <10E-06 |
| haplo.base16 | 17 | G | C | C | W | 0.76459 | NA | NA | NA | NA |
| Geno.46 | 29 | G | C | A | T | 0.15147 | 6.5 | 3.3 | 1.9 | 0.05198 |
| Geno.56 | 29 | G | C | G | G | 0.03866 | 14.6 | 6.1 | 2.4 | 0.01690 |
| Geno.rare28 | 29 | * | * | * | * | 0.00419 | 24.6 | 0.1 | 345.5 | <10E-06 |
| haplo.base28 | 29 | G | C | G | T | 0.80569 | NA | NA | NA | NA |
| Geno.26 | 30 | C | A | T | G | 0.15210 | 6.7 | 3.3 | 2.0 | 0.04235 |
| Geno.311 | 30 | C | G | G | G | 0.03876 | 14.8 | 6.0 | 2.5 | 0.01430 |
| Geno.rare29 | 30 | * | * | * | * | 0.00243 | 84.1 | 0.03 | 2478.6 | <10E-06 |
| haplo.base29 | 30 | C | G | T | G | 0.80671 | NA | NA | NA | NA |
| Geno.27 | 31 | A | T | G | A | 0.15411 | 6.8 | 3.3 | 2.0 | 0.04109 |
| Geno.47 | 31 | G | G | G | G | 0.03785 | 13.7 | 6.2 | 2.2 | 0.02751 |
| Geno.68 | 31 | G | T | G | A | 0.03416 | 8.1 | 6.7 | 1.2 | 0.22501 |
| Geno.rare30 | 31 | * | * | * | * | 0.00172 | 49.8 | NA | NA | NA |
| haplo.base30 | 31 | G | T | G | G | 0.77215 | NA | NA | NA | NA |
| Geno.28 | 32 | G | G | G | A | 0.03870 | 14.7 | 6.1 | 2.4 | 0.01550 |
| Geno.48 | 32 | T | G | A | G | 0.18935 | 6.5 | 3.0 | 2.2 | 0.03130 |
| Geno.rare31 | 32 | * | * | * | * | 0.00259 | 65.1 | 0.03 | 2546.5 | <10E-06 |
| haplo.base31 | 32 | T | G | G | A | 0.76936 | NA | NA | NA | NA |
| Geno.312 | 33 | G | A | G | C | 0.18490 | 10.7 | 3.3 | 3.2 | 0.00144 |
| Geno.69 | 33 | G | G | A | G | 0.37287 | 9.5 | 2.7 | 3.5 | 0.00056 |
| Geno.rare32 | 33 | * | * | * | * | 0.00706 | 37.4 | 0.02 | 2008.4 | <10E-06 |
| haplo.base32 | 33 | G | G | A | C | 0.43518 | NA | NA | NA | NA |
| Geno.13 | 34 | A | G | C | C | 0.18498 | 10.7 | 3.3 | 3.2 | 0.00142 |
| Geno.610 | 34 | G | A | G | C | 0.34857 | 9.7 | 2.8 | 3.5 | 0.00056 |
| Geno.75 | 34 | G | A | G | G | 0.02458 | 6.6 | 7.4 | 0.9 | 0.37435 |
| Geno.rare33 | 34 | * | * | * | * | 0.00614 | 35.5 | 0.1 | 593.9 | <10E-06 |
| haplo.base33 | 34 | G | A | C | C | 0.43573 | NA | NA | NA | NA |
| Geno.49 | 35 | A | G | C | C | 0.34618 | 9.2 | 2.8 | 3.3 | 0.00106 |
| Geno.611 | 35 | A | G | G | C | 0.02388 | 5.6 | 7.6 | 0.7 | 0.46084 |
| Geno.76 | 35 | G | C | C | C | 0.15457 | 9.8 | 3.5 | 2.8 | 0.00547 |
| Geno.rare34 | 35 | * | * | * | * | 0.00818 | 20.9 | 0.1 | 146.7 | <10E-06 |
| haplo.base34 | 35 | A | C | C | C | 0.46719 | NA | NA | NA | NA |
| Geno.314 | 40 | A | T | G | C | 0.42316 | 8.2 | 2.7 | 3.1 | 0.00220 |
| Geno.412 | 40 | A | T | G | G | 0.11478 | -5.8 | 3.9 | -1.5 | 0.13185 |
| Geno.rare39 | 40 | * | * | * | * | 0.00378 | -21.0 | 0.1 | -274.3 | <10E-06 |
| haplo.base39 | 40 | A | T | C | C | 0.45827 | NA | NA | NA | NA |
| Geno.315 | 41 | T | C | C | C | 0.32107 | -7.6 | 3.3 | -2.3 | 0.02337 |
| Geno.413 | 41 | T | C | C | G | 0.13659 | -9.7 | 4.0 | -2.4 | 0.01497 |
| Geno.83 | 41 | T | G | C | G | 0.06375 | -0.6 | 6.3 | -0.1 | 0.92727 |
| Geno.91 | 41 | T | G | G | C | 0.08905 | -14.5 | 4.8 | -3.0 | 0.00252 |
| Geno.10 | 41 | T | G | G | G | 0.02590 | -12.7 | 8.9 | -1.4 | 0.15319 |
| Geno.rare40 | 41 | * | * | * | * | 0.00363 | -29.0 | 0.2 | -154.3 | <10E-06 |
| haplo.base40 | 41 | T | G | C | C | 0.36000 | NA | NA | NA | NA |
| Geno.15 | 42 | C | C | C | C | 0.32319 | -7.9 | 3.3 | -2.4 | 0.01861 |
| Geno.316 | 42 | C | C | G | C | 0.13119 | -9.8 | 4.1 | -2.4 | 0.01697 |
| Geno.77 | 42 | G | C | G | C | 0.06517 | -0.5 | 6.3 | -0.1 | 0.93707 |
| Geno.84 | 42 | G | G | C | C | 0.09072 | -14.9 | 4.8 | -3.1 | 0.00190 |
| Geno.92 | 42 | G | G | G | C | 0.02564 | -13.0 | 9.0 | -1.5 | 0.14753 |
| Geno.rare41 | 42 | * | * | * | * | 0.00539 | -8.6 | 0.3 | -28.8 | <10E-06 |
| haplo.base41 | 42 | G | C | C | C | 0.35870 | NA | NA | NA | NA |
| Geno.317 | 46 | C | C | G | A | 0.03624 | -4.7 | 6.4 | -0.7 | 0.45918 |
| Geno.59 | 46 | T | C | A | A | 0.02261 | 8.4 | 8.1 | 1.0 | 0.30381 |
| Geno.rare45 | 46 | * | * | * | * | 0.00248 | 89.6 | 0.05 | 1945.7 | <10E-06 |
| haplo.base45 | 46 | C | C | A | A | 0.93866 | NA | NA | NA | NA |
| Geno.417 | 47 | C | G | A | G | 0.03624 | -5.1 | 6.4 | -0.8 | 0.42772 |
| Geno.rare46 | 47 | * | * | * | * | 0.00410 | 60.5 | 0.1 | 1100.7 | <10E-06 |
| haplo.base46 | 47 | C | A | A | G | 0.95966 | NA | NA | NA | NA |
| Geno.422 | 60 | G | G | A | G | 0.03710 | -4.2 | 6.4 | -0.7 | 0.51117 |
| Geno.517 | 60 | G | G | G | G | 0.45483 | -0.8 | 2.4 | -0.3 | 0.73305 |
| Geno.rare58 | 60 | * | * | * | * | 0.00482 | 58.5 | 0.2 | 359.7 | <10E-06 |
| haplo.base59 | 60 | C | G | G | G | 0.50325 | NA | NA | NA | NA |
| Geno.320 | 61 | G | A | G | G | 0.03721 | -4.2 | 6.3 | -0.7 | 0.51082 |
| Geno.423 | 61 | G | G | G | A | 0.03628 | -3.6 | 6.4 | -0.6 | 0.57191 |
| Geno.rare59 | 61 | * | * | * | * | 0.00482 | 58.7 | 0.1 | 635.4 | <10E-06 |
| haplo.base60 | 61 | G | G | G | G | 0.92169 | NA | NA | NA | NA |
| Geno.18 | 62 | A | G | G | A | 0.03718 | -4.8 | 6.4 | -0.7 | 0.45765 |
| Geno.212 | 62 | G | G | A | A | 0.03722 | -3.0 | 6.4 | -0.5 | 0.64363 |
| Geno.321 | 62 | G | G | G | A | 0.41954 | -1.5 | 2.4 | -0.6 | 0.54721 |
| Geno.rare60 | 62 | * | * | * | * | 0.00402 | 57.0 | 0.2 | 292.4 | <10E-06 |
| haplo.base61 | 62 | G | G | G | T | 0.50204 | NA | NA | NA | NA |
| Geno.19 | 63 | G | A | A | D | 0.03736 | -2.8 | 6.4 | -0.4 | 0.66380 |
| Geno.213 | 63 | G | G | A | D | 0.18233 | -5.2 | 3.1 | -1.7 | 0.09413 |
| Geno.322 | 63 | G | G | A | W | 0.27595 | 0.9 | 2.8 | 0.3 | 0.74682 |
| Geno.rare61 | 63 | * | * | * | * | 0.00606 | 45.7 | 0.2 | 223.1 | <10E-06 |
| haplo.base62 | 63 | G | G | T | D | 0.49830 | NA | NA | NA | NA |

hap.freq: haplotype frequency; coef: coefficient; se: standard error; t.stat: test statistic; p-val: haplotype p-value
