# Supplementary material for: Apolipoprotein E-C1-C4-C2 gene cluster region and inter-individual variation in plasma lipoprotein levels: a comprehensive genetic association study in two ethnic groups
Source: PLoS One. 2019 Mar 26;14(3):e0214060. doi: 10.1371/journal.pone.0214060 (PMC6435132; doi:10.1371/journal.pone.0214060)
Supplement: S26 Table — hap.freq: haplotype frequency; coef: coefficient; se: standard error; t.stat: test statistic; p-val: haplotype p-value. (DOCX) [file pone.0214060.s026.docx]

S26 Table. Haplotype summary of significant association with apoB in NHWs

| **ApoB^a^** | | | | | | | | | | |
| --- | --- | --- | --- | --- | --- | --- | --- | --- | --- | --- |
|  | **Window** | **loc.1** | **loc.2** | **loc.3** | **loc.4** | **hap.freq** | **coef** | **se** | **t.stat** | **pval** |
| Geno.2 | 1 | A | T | C | C | 0.25342 | 0.24 | 0.58 | 0.41 | 0.67873 |
| Geno.3 | 1 | A | T | C | T | 0.02214 | 0.92 | 1.41 | 0.65 | 0.51564 |
| Geno.4 | 1 | A | T | G | C | 0.13135 | 2.00 | 0.69 | 2.91 | 0.00382 |
| Geno.6 | 1 | T | G | G | C | 0.07481 | -2.62 | 0.95 | -2.75 | 0.00616 |
| Geno.7 | 1 | T | T | C | C | 0.07812 | 1.36 | 0.86 | 1.58 | 0.11430 |
| Geno.rare | 1 | * | * | * | * | 0.00116 | -20.89 | 6.37 | -3.28 | 0.00113 |
| haplo.base | 1 | A | G | G | C | 0.43901 | NA | NA | NA | NA |
| Geno.5 | 2 | T | C | C | G | 0.32882 | 1.04 | 0.49 | 2.13 | 0.03342 |
| Geno.61 | 2 | T | C | T | G | 0.02138 | 1.56 | 1.45 | 1.07 | 0.28307 |
| Geno.71 | 2 | T | G | C | A | 0.12575 | 2.47 | 0.70 | 3.55 | 0.00042 |
| Geno.rare1 | 2 | * | * | * | * | 0.01043 | -4.18 | 2.26 | -1.85 | 0.06480 |
| haplo.base1 | 2 | G | G | C | G | 0.51361 | NA | NA | NA | NA |
| Geno.21 | 3 | C | C | G | G | 0.33007 | 0.18 | 0.51 | 0.36 | 0.72077 |
| Geno.31 | 3 | C | T | G | G | 0.02224 | 0.46 | 1.42 | 0.32 | 0.74598 |
| Geno.51 | 3 | G | C | A | G | 0.12699 | 1.65 | 0.70 | 2.34 | 0.01960 |
| Geno.72 | 3 | G | C | G | G | 0.12121 | -3.20 | 0.70 | -4.53 | 7.49E-06 |
| Geno.rare2 | 3 | * | * | * | * | 0.00253 | -6.15 | 4.42 | -1.39 | 0.16502 |
| haplo.base2 | 3 | G | C | G | A | 0.39695 | NA | NA | NA | NA |
| Geno.32 | 4 | C | A | G | T | 0.12743 | 2.36 | 0.73 | 3.26 | 0.00122 |
| Geno.52 | 4 | C | G | A | T | 0.39624 | 0.71 | 0.49 | 1.46 | 0.14637 |
| Geno.10 | 4 | T | G | G | T | 0.02213 | 1.39 | 1.45 | 0.96 | 0.33866 |
| Geno.rare3 | 4 | * | * | * | * | 0.01037 | 0.06 | 2.23 | 0.03 | 0.97919 |
| haplo.base3 | 4 | C | G | G | T | 0.44384 | NA | NA | NA | NA |
| Geno.33 | 5 | A | G | T | G | 0.12687 | 2.26 | 0.72 | 3.13 | 0.00185 |
| Geno.62 | 5 | G | A | T | G | 0.39505 | 0.59 | 0.49 | 1.22 | 0.22353 |
| Geno.rare4 | 5 | * | * | * | * | 0.01153 | 1.49 | 2.10 | 0.71 | 0.47789 |
| haplo.base4 | 5 | G | G | T | G | 0.46655 | NA | NA | NA | NA |
| Geno.63 | 7 | T | G | T | C | 0.15823 | 2.04 | 0.61 | 3.31 | 0.00101 |
| Geno.rare6 | 7 | * | * | * | * | 0.01268 | 2.37 | 1.97 | 1.20 | 0.22976 |
| haplo.base6 | 7 | T | G | T | T | 0.82909 | NA | NA | NA | NA |
| Geno.35 | 8 | G | T | C | C | 0.16061 | 1.52 | 0.58 | 2.63 | 0.00888 |
| Geno.64 | 8 | G | T | T | T | 0.08101 | -5.37 | 0.76 | -7.03 | 8.41E-12 |
| Geno.rare7 | 8 | * | * | * | * | 0.00321 | 10.05 | 4.26 | 2.36 | 0.01883 |
| haplo.base7 | 8 | G | T | T | C | 0.75517 | NA | NA | NA | NA |
| Geno.36 | 9 | T | C | C | T | 0.15861 | 1.37 | 0.60 | 2.28 | 0.02327 |
| Geno.73 | 9 | T | T | T | T | 0.07972 | -5.53 | 0.81 | -6.79 | 3.76E-11 |
| Geno.rare8 | 9 | * | * | * | * | 0.00565 | 3.67 | 3.38 | 1.09 | 0.27814 |
| haplo.base8 | 9 | T | T | C | T | 0.75602 | NA | NA | NA | NA |
| Geno.22 | 10 | C | C | T | C | 0.16058 | 1.50 | 0.61 | 2.46 | 0.01427 |
| Geno.74 | 10 | T | T | T | C | 0.08045 | -5.41 | 0.84 | -6.47 | 2.67E-10 |
| Geno.rare9 | 10 | * | * | * | * | 0.01185 | 1.31 | 2.18 | 0.60 | 0.54871 |
| haplo.base9 | 10 | T | C | T | C | 0.74712 | NA | NA | NA | NA |
| Geno.37 | 11 | C | T | C | C | 0.07016 | -0.15 | 0.81 | -0.18 | 0.85709 |
| Geno.8 | 11 | T | T | C | T | 0.08133 | -5.59 | 0.76 | -7.32 | 1.21E-12 |
| Geno.rare10 | 11 | * | * | * | * | 0.01103 | 0.37 | 2.07 | 0.18 | 0.85654 |
| haplo.base10 | 11 | C | T | C | T | 0.83749 | NA | NA | NA | NA |
| Geno.1 | 13 | C | C | C | G | 0.07172 | -0.59 | 0.85 | -0.70 | 0.48383 |
| Geno.23 | 13 | C | T | C | A | 0.11088 | -4.07 | 0.73 | -5.57 | 4.54E-08 |
| Geno.42 | 13 | C | T | T | G | 0.35676 | -0.49 | 0.48 | -1.03 | 0.30417 |
| Geno.rare12 | 13 | * | * | * | * | 0.00809 | 0.58 | 2.52 | 0.23 | 0.81910 |
| haplo.base12 | 13 | C | T | C | G | 0.45256 | NA | NA | NA | NA |
| Geno.24 | 14 | C | C | G | C | 0.07488 | -0.45 | 0.83 | -0.54 | 0.59038 |
| Geno.38 | 14 | T | C | A | C | 0.11088 | -4.09 | 0.73 | -5.61 | 3.67E-08 |
| Geno.75 | 14 | T | T | G | C | 0.35721 | -0.49 | 0.48 | -1.02 | 0.30781 |
| Geno.rare13 | 14 | * | * | * | * | 0.00233 | -3.82 | 4.44 | -0.86 | 0.38954 |
| haplo.base13 | 14 | T | C | G | C | 0.45470 | NA | NA | NA | NA |
| Geno.11 | 15 | C | A | C | C | 0.10979 | -4.05 | 0.72 | -5.59 | 4.00E-08 |
| Geno.65 | 15 | T | G | C | C | 0.35846 | -0.45 | 0.46 | -0.98 | 0.32812 |
| Geno.rare14 | 15 | * | * | * | * | 0.00348 | -3.19 | 3.64 | -0.88 | 0.38004 |
| haplo.base14 | 15 | C | G | C | C | 0.52827 | NA | NA | NA | NA |
| Geno.12 | 16 | A | C | C | I | 0.10844 | -3.63 | 0.69 | -5.24 | 2.49E-07 |
| Geno.66 | 16 | G | C | C | I | 0.13248 | 1.42 | 0.64 | 2.22 | 0.02712 |
| Geno.rare15 | 16 | * | * | * | * | 0.00483 | -2.00 | 3.16 | -0.63 | 0.52723 |
| haplo.base15 | 16 | G | C | C | W | 0.75425 | NA | NA | NA | NA |
| Geno.26 | 19 | I | G | A | G | 0.24023 | -0.77 | 0.52 | -1.48 | 0.13858 |
| Geno.rare18 | 19 | * | * | * | * | 0.00465 | 7.68 | 3.24 | 2.37 | 0.01838 |
| haplo.base18 | 19 | W | G | A | G | 0.75512 | NA | NA | NA | NA |
| Geno.rare19 | 20 | * | * | * | * | 0.00581 | 6.84 | 3.24 | 2.11 | 0.03538 |
| haplo.base19 | 20 | G | A | G | G | 0.99419 | NA | NA | NA | NA |
| Geno.311 | 27 | C | G | C | A | 0.16262 | 1.61 | 0.61 | 2.66 | 0.00813 |
| Geno.rare26 | 27 | * | * | * | * | 0.00825 | -2.29 | 2.80 | -0.82 | 0.41387 |
| haplo.base26 | 27 | C | G | C | G | 0.82914 | NA | NA | NA | NA |
| Geno.44 | 28 | G | C | A | T | 0.16292 | 1.67 | 0.61 | 2.75 | 0.00623 |
| Geno.55 | 28 | G | C | G | G | 0.03488 | 2.17 | 1.18 | 1.85 | 0.06524 |
| Geno.rare27 | 28 | * | * | * | * | 0.00469 | -0.72 | 3.76 | -0.19 | 0.84788 |
| haplo.base27 | 28 | G | C | G | T | 0.79751 | NA | NA | NA | NA |
| Geno.27 | 29 | C | A | T | A | 0.16259 | 1.71 | 0.61 | 2.81 | 0.00519 |
| Geno.45 | 29 | C | G | G | G | 0.03107 | 1.80 | 1.32 | 1.36 | 0.17327 |
| Geno.56 | 29 | C | G | T | A | 0.03393 | 1.74 | 1.28 | 1.36 | 0.17368 |
| Geno.rare28 | 29 | * | * | * | * | 0.00499 | 6.58 | 4.47 | 1.47 | 0.14222 |
| haplo.base28 | 29 | C | G | T | G | 0.76742 | NA | NA | NA | NA |
| Geno.46 | 30 | A | T | A | G | 0.16377 | 1.63 | 0.61 | 2.67 | 0.00791 |
| Geno.68 | 30 | G | G | G | A | 0.03111 | 1.97 | 1.35 | 1.46 | 0.14504 |
| Geno.82 | 30 | G | T | A | G | 0.03403 | 1.83 | 1.29 | 1.42 | 0.15664 |
| Geno.rare29 | 30 | * | * | * | * | 0.00631 | 4.40 | 3.90 | 1.13 | 0.26068 |
| haplo.base29 | 30 | G | T | G | A | 0.76478 | NA | NA | NA | NA |
| Geno.28 | 31 | G | G | A | C | 0.03480 | 2.94 | 1.19 | 2.47 | 0.01378 |
| Geno.312 | 31 | T | A | G | C | 0.19780 | 2.67 | 0.62 | 4.31 | 2.06E-05 |
| Geno.69 | 31 | T | G | A | G | 0.35656 | 1.64 | 0.52 | 3.18 | 0.00155 |
| Geno.rare30 | 31 | * | * | * | * | 0.00629 | 0.48 | 3.06 | 0.16 | 0.87473 |
| haplo.base30 | 31 | T | G | A | C | 0.40454 | NA | NA | NA | NA |
| Geno.29 | 32 | A | G | C | C | 0.19761 | 2.53 | 0.62 | 4.07 | 5.68E-05 |
| Geno.76 | 32 | G | A | G | C | 0.33425 | 1.43 | 0.53 | 2.72 | 0.00672 |
| Geno.83 | 32 | G | A | G | G | 0.02235 | 1.43 | 1.43 | 1.00 | 0.31820 |
| Geno.rare31 | 32 | * | * | * | * | 0.00649 | 0.27 | 3.08 | 0.09 | 0.93039 |
| haplo.base31 | 32 | G | A | C | C | 0.43929 | NA | NA | NA | NA |
| Geno.47 | 33 | A | G | C | C | 0.33253 | 1.25 | 0.53 | 2.35 | 0.01942 |
| Geno.57 | 33 | A | G | G | C | 0.02235 | 1.20 | 1.44 | 0.83 | 0.40541 |
| Geno.77 | 33 | G | C | C | C | 0.16958 | 2.08 | 0.65 | 3.19 | 0.00154 |
| Geno.rare32 | 33 | * | * | * | * | 0.00710 | 1.35 | 3.06 | 0.44 | 0.66038 |
| haplo.base32 | 33 | A | C | C | C | 0.46843 | NA | NA | NA | NA |
| Geno.415 | 47 | C | C | C | G | 0.36666 | 0.31 | 0.47 | 0.65 | 0.51801 |
| Geno.515 | 47 | C | C | T | A | 0.01929 | -5.00 | 1.52 | -3.28 | 0.00114 |
| Geno.612 | 47 | C | C | T | G | 0.11485 | 1.44 | 0.71 | 2.02 | 0.04446 |
| Geno.rare46 | 47 | * | * | * | * | 0.00381 | -3.04 | 3.69 | -0.82 | 0.41088 |
| haplo.base46 | 47 | C | T | C | G | 0.49539 | NA | NA | NA | NA |
| Geno.212 | 48 | C | C | G | G | 0.36783 | 0.29 | 0.47 | 0.61 | 0.54354 |
| Geno.416 | 48 | C | T | A | G | 0.01851 | -5.01 | 1.55 | -3.23 | 0.00135 |
| Geno.613 | 48 | C | T | G | G | 0.11483 | 1.45 | 0.71 | 2.02 | 0.04349 |
| Geno.rare47 | 48 | * | * | * | * | 0.01195 | -1.28 | 2.12 | -0.60 | 0.54826 |
| haplo.base47 | 48 | T | C | G | G | 0.48689 | NA | NA | NA | NA |
| Geno.213 | 49 | C | G | G | C | 0.37976 | 0.09 | 0.48 | 0.18 | 0.85784 |
| Geno.614 | 49 | T | A | G | C | 0.01851 | -5.09 | 1.55 | -3.27 | 0.00115 |
| Geno.93 | 49 | T | G | G | C | 0.11481 | 1.37 | 0.72 | 1.90 | 0.05776 |
| Geno.rare48 | 49 | * | * | * | * | 0.01194 | -1.31 | 2.12 | -0.62 | 0.53790 |
| haplo.base48 | 49 | C | G | G | T | 0.47498 | NA | NA | NA | NA |
| Geno.14 | 50 | A | G | C | G | 0.02144 | -5.20 | 1.47 | -3.54 | 0.00045 |
| Geno.615 | 50 | G | G | T | G | 0.47399 | -0.41 | 0.45 | -0.92 | 0.35904 |
| Geno.rare49 | 50 | * | * | * | * | 0.01159 | -1.74 | 2.16 | -0.81 | 0.41994 |
| haplo.base49 | 50 | G | G | C | G | 0.49298 | NA | NA | NA | NA |

hap.freq: haplotype frequency; coef: coefficient; se: standard error; t.stat: test statistic; p-val: haplotype p-value
