# Supplementary material for: Apolipoprotein E-C1-C4-C2 gene cluster region and inter-individual variation in plasma lipoprotein levels: a comprehensive genetic association study in two ethnic groups
Source: PLoS One. 2019 Mar 26;14(3):e0214060. doi: 10.1371/journal.pone.0214060 (PMC6435132; doi:10.1371/journal.pone.0214060)
Supplement: S27 Table — hap.freq: haplotype frequency; coef: coefficient; se: standard error; t.stat: test statistic; p-val: haplotype p-value. (DOCX) [file pone.0214060.s027.docx]

S27 Table. Haplotype summary of significant windows with HDL-C in NHWs

| **HDL-C^a^** | | | | | | | | | | |
| --- | --- | --- | --- | --- | --- | --- | --- | --- | --- | --- |
|  | **Window** | **loc.1** | **loc.2** | **loc.3** | **loc.4** | **hap.freq** | **coef** | **se** | **t.stat** | **pval** |
| Geno.37 | 12 | T | C | C | C | 0.08136 | 0.03 | 0.02 | 1.77 | 0.07798 |
| Geno.rare11 | 12 | * | * | * | * | 0.01296 | 0.10 | 0.05 | 1.93 | 0.05354 |
| haplo.base11 | 12 | T | C | C | T | 0.90568 | NA | NA | NA | NA |
| Geno.1 | 13 | C | C | C | C | 0.08002 | 0.04 | 0.02 | 1.74 | 0.08184 |
| Geno.42 | 13 | C | C | T | T | 0.35455 | 0.01 | 0.01 | 0.75 | 0.45065 |
| Geno.rare12 | 13 | * | * | * | * | 0.01078 | 0.13 | 0.06 | 2.38 | 0.01768 |
| haplo.base12 | 13 | C | C | T | C | 0.55465 | NA | NA | NA | NA |
| Geno.24 | 19 | I | G | A | G | 0.22903 | -0.02 | 0.01 | -1.67 | 0.09583 |
| Geno.rare18 | 19 | * | * | * | * | 0.00324 | 0.32 | 0.13 | 2.44 | 0.01497 |
| haplo.base18 | 19 | W | G | A | G | 0.76773 | NA | NA | NA | NA |
| Geno.rare19 | 20 | * | * | * | * | 0.00486 | 0.28 | 0.09 | 3.01 | 0.00268 |
| haplo.base19 | 20 | G | A | G | G | 0.99514 | NA | NA | NA | NA |
| Geno.315 | 40 | T | C | C | C | 0.32054 | -0.02 | 0.02 | -1.44 | 0.15061 |
| Geno.414 | 40 | T | C | C | G | 0.13846 | 0.03 | 0.02 | 1.98 | 0.04796 |
| Geno.83 | 40 | T | G | C | G | 0.06261 | 0.01 | 0.03 | 0.43 | 0.67006 |
| Geno.91 | 40 | T | G | G | C | 0.08989 | 0.02 | 0.02 | 1.00 | 0.31985 |
| Geno.10 | 40 | T | G | G | G | 0.02512 | -0.04 | 0.04 | -0.89 | 0.37283 |
| Geno.rare39 | 40 | * | * | * | * | 0.00358 | -0.16 | 0.11 | -1.43 | 0.15249 |
| haplo.base39 | 40 | T | G | C | C | 0.35980 | NA | NA | NA | NA |
| Geno.416 | 43 | C | C | T | C | 0.01751 | 0.01 | 0.04 | 0.23 | 0.81592 |
| Geno.612 | 43 | G | C | C | C | 0.21727 | 0.03 | 0.01 | 2.59 | 0.00980 |
| Geno.rare42 | 43 | * | * | * | * | 0.01007 | -0.11 | 0.06 | -1.70 | 0.08927 |
| haplo.base42 | 43 | C | C | C | C | 0.75515 | NA | NA | NA | NA |
| Geno.513 | 49 | G | C | C | C | 0.36252 | 0.001 | 0.01 | 0.05 | 0.96388 |
| Geno.614 | 49 | G | C | C | T | 0.13896 | 0.02 | 0.02 | 1.28 | 0.20088 |
| Geno.rare48 | 49 | * | * | * | * | 0.00242 | -0.32 | 0.13 | -2.42 | 0.01563 |
| haplo.base48 | 49 | G | C | T | C | 0.49609 | NA | NA | NA | NA |
| Geno.318 | 57 | T | G | C | G | 0.02125 | 0.07 | 0.04 | 1.81 | 0.07062 |
| Geno.517 | 57 | T | G | G | G | 0.33704 | 0.001 | 0.01 | 0.10 | 0.91679 |
| Geno.79 | 57 | T | T | G | G | 0.15965 | 0.02 | 0.02 | 1.53 | 0.12595 |
| Geno.rare56 | 57 | * | * | * | * | 0.00242 | -0.34 | 0.13 | -2.62 | 0.00910 |
| haplo.base56 | 57 | T | T | C | G | 0.47963 | NA | NA | NA | NA |

hap.freq: haplotype frequency; coef: coefficient; se: standard error; t.stat: test statistic; p-val: haplotype p-value
