# Supplementary material for: Apolipoprotein E-C1-C4-C2 gene cluster region and inter-individual variation in plasma lipoprotein levels: a comprehensive genetic association study in two ethnic groups
Source: PLoS One. 2019 Mar 26;14(3):e0214060. doi: 10.1371/journal.pone.0214060 (PMC6435132; doi:10.1371/journal.pone.0214060)
Supplement: S28 Table — hap.freq: haplotype frequency; coef: coefficient; se: standard error; t.stat: test statistic; p-val: haplotype p-value. (DOCX) [file pone.0214060.s028.docx]

S28 Table. Haplotype summary of significant windows with ApoA1 in NHWs

| **ApoA1** | | | | | | | | | | |
| --- | --- | --- | --- | --- | --- | --- | --- | --- | --- | --- |
|  | **Window** | **loc.1** | **loc.2** | **loc.3** | **loc.4** | **hap.freq** | **coef** | **se** | **t.stat** | **pval** |
| Geno.25 | 18 | C | I | G | A | 0.23963 | -0.79 | 2.62 | -0.30 | 0.76340 |
| Geno.rare17 | 18 | * | * | * | * | 0.00347 | 49.06 | 0.04 | 1119.99 | <10E-06 |
| haplo.base17 | 18 | C | W | G | A | 0.75690 | NA | NA | NA | NA |
| Geno.26 | 19 | I | G | A | G | 0.24078 | -0.02 | 2.62 | -0.01 | 0.99278 |
| Geno.rare18 | 19 | * | * | * | * | 0.00463 | 44.78 | 0.06 | 761.17 | <10E-06 |
| haplo.base18 | 19 | W | G | A | G | 0.75459 | NA | NA | NA | NA |
| Geno.rare19 | 20 | * | * | * | * | 0.00581 | 30.69 | 0.01 | 2087.61 | <10E-06 |
| haplo.base19 | 20 | G | A | G | G | 0.99419 | NA | NA | NA | NA |
| Geno.321 | 52 | C | G | G | C | 0.02074 | -11.21 | 7.92 | -1.42 | 0.15749 |
| Geno.418 | 52 | C | G | G | G | 0.34125 | -0.77 | 2.52 | -0.31 | 0.76016 |
| Geno.617 | 52 | C | G | T | G | 0.15064 | -1.13 | 3.27 | -0.34 | 0.73060 |
| Geno.rare51 | 52 | * | * | * | * | 0.00609 | 41.27 | 0.22 | 184.54 | <10E-06 |
| haplo.base51 | 52 | T | G | T | C | 0.48127 | NA | NA | NA | NA |

hap.freq: haplotype frequency; coef: coefficient; se: standard error; t.stat: test statistic; p-val: haplotype p-value
