# Supplementary material for: Apolipoprotein E-C1-C4-C2 gene cluster region and inter-individual variation in plasma lipoprotein levels: a comprehensive genetic association study in two ethnic groups
Source: PLoS One. 2019 Mar 26;14(3):e0214060. doi: 10.1371/journal.pone.0214060 (PMC6435132; doi:10.1371/journal.pone.0214060)
Supplement: S29 Table — hap.freq: haplotype frequency; coef: coefficient; se: standard error; t.stat: test statistic; p-val: haplotype p-value. (DOCX) [file pone.0214060.s029.docx]

S29 Table. Haplotype summary of significant windows with TG in NHWs

| **TG** | | | | | | | | | | |
| --- | --- | --- | --- | --- | --- | --- | --- | --- | --- | --- |
|  | Window | loc.1 | loc.2 | loc.3 | loc.4 | hap.freq | coef | se | t.stat | pval |
| Geno.3 | 1 | A | T | C | C | 0.25969 | -0.09 | 0.03 | -3.06 | 0.00229 |
| Geno.4 | 1 | A | T | C | T | 0.02118 | -0.12 | 0.08 | -1.61 | 0.10696 |
| Geno.5 | 1 | A | T | G | C | 0.11909 | -0.02 | 0.04 | -0.56 | 0.57379 |
| Geno.7 | 1 | T | G | G | C | 0.08150 | -0.05 | 0.05 | -0.98 | 0.32783 |
| Geno.8 | 1 | T | T | C | C | 0.07674 | -0.07 | 0.05 | -1.60 | 0.10978 |
| Geno.rare | 1 | * | * | * | * | 0.00185 | 0.68 | 0.39 | 1.76 | 0.07969 |
| haplo.base | 1 | A | G | G | C | 0.43997 | NA | NA | NA | NA |
| Geno.51 | 2 | T | C | C | G | 0.33508 | -0.08 | 0.03 | -3.11 | 0.00198 |
| Geno.6 | 2 | T | C | T | G | 0.02076 | -0.12 | 0.08 | -1.52 | 0.12829 |
| Geno.71 | 2 | T | G | C | A | 0.11339 | -0.01 | 0.04 | -0.20 | 0.84085 |
| Geno.rare1 | 2 | * | * | * | * | 0.01063 | -0.09 | 0.11 | -0.80 | 0.42648 |
| haplo.base1 | 2 | G | G | C | G | 0.52013 | NA | NA | NA | NA |
| Geno.2 | 3 | C | C | G | G | 0.33636 | -0.08 | 0.03 | -3.08 | 0.00215 |
| Geno.31 | 3 | C | T | G | G | 0.02124 | -0.13 | 0.08 | -1.67 | 0.09597 |
| Geno.52 | 3 | G | C | A | G | 0.11483 | -0.02 | 0.04 | -0.49 | 0.62712 |
| Geno.72 | 3 | G | C | G | G | 0.12204 | -0.05 | 0.04 | -1.26 | 0.20779 |
| Geno.rare2 | 3 | * | * | * | * | 0.00185 | -0.44 | 0.27 | -1.60 | 0.11047 |
| haplo.base2 | 3 | G | C | G | A | 0.40368 | NA | NA | NA | NA |
| Geno.33 | 5 | A | G | T | G | 0.11458 | 0.05 | 0.04 | 1.26 | 0.20924 |
| Geno.61 | 5 | G | A | T | G | 0.40146 | 0.07 | 0.02 | 2.90 | 0.00384 |
| Geno.rare4 | 5 | * | * | * | * | 0.01295 | -0.003 | 0.10 | -0.03 | 0.97936 |
| haplo.base4 | 5 | G | G | T | G | 0.47101 | NA | NA | NA | NA |
| Geno.67 | 23 | G | G | G | A | 0.33711 | -0.08 | 0.02 | -3.12 | 0.00187 |
| Geno.rare22 | 23 | * | * | * | * | 0.00909 | -0.16 | 0.12 | -1.35 | 0.17626 |
| haplo.base22 | 23 | G | G | G | G | 0.65380 | NA | NA | NA | NA |
| Geno.55 | 24 | G | G | A | G | 0.33622 | -0.08 | 0.02 | -3.12 | 0.00191 |
| Geno.rare23 | 24 | * | * | * | * | 0.00825 | -0.18 | 0.13 | -1.30 | 0.19293 |
| haplo.base23 | 24 | G | G | G | G | 0.65554 | NA | NA | NA | NA |
| Geno.45 | 25 | G | A | G | C | 0.33819 | -0.08 | 0.02 | -3.22 | 0.00134 |
| Geno.rare24 | 25 | * | * | * | * | 0.00997 | -0.06 | 0.12 | -0.47 | 0.63557 |
| haplo.base24 | 25 | G | G | G | C | 0.65185 | NA | NA | NA | NA |
| Geno.39 | 26 | A | G | C | G | 0.33966 | -0.08 | 0.02 | -3.23 | 0.00130 |
| Geno.rare25 | 26 | * | * | * | * | 0.00663 | -0.01 | 0.16 | -0.07 | 0.94310 |
| haplo.base25 | 26 | G | G | C | G | 0.65372 | NA | NA | NA | NA |
| Geno.312 | 33 | G | A | G | C | 0.18546 | 0.03 | 0.03 | 0.84 | 0.40320 |
| Geno.69 | 33 | G | G | A | G | 0.37369 | 0.05 | 0.03 | 1.77 | 0.07645 |
| Geno.rare32 | 33 | * | * | * | * | 0.00692 | 0.39 | 0.22 | 1.80 | 0.07293 |
| haplo.base32 | 33 | G | G | A | C | 0.43394 | NA | NA | NA | NA |
| Geno.417 | 47 | C | G | A | G | 0.03647 | 0.02 | 0.06 | 0.32 | 0.74844 |
| Geno.rare46 | 47 | * | * | * | * | 0.00412 | 0.57 | 0.20 | 2.88 | 0.00414 |
| haplo.base46 | 47 | C | A | A | G | 0.95941 | NA | NA | NA | NA |
| Geno.510 | 48 | G | A | G | C | 0.03647 | 0.02 | 0.06 | 0.30 | 0.76284 |
| Geno.rare47 | 48 | * | * | * | * | 0.00410 | 0.45 | 0.20 | 2.26 | 0.02417 |
| haplo.base47 | 48 | A | A | G | C | 0.95944 | NA | NA | NA | NA |
| Geno.511 | 49 | A | G | C | T | 0.49347 | 0.02 | 0.02 | 0.83 | 0.40944 |
| Geno.rare48 | 49 | * | * | * | * | 0.00410 | 0.47 | 0.20 | 2.32 | 0.02069 |
| haplo.base48 | 49 | A | G | C | C | 0.50244 | NA | NA | NA | NA |
| Geno.318 | 58 | T | G | C | G | 0.02133 | -0.07 | 0.09 | -0.76 | 0.44821 |
| Geno.516 | 58 | T | G | G | G | 0.33732 | -0.01 | 0.03 | -0.48 | 0.63124 |
| Geno.78 | 58 | T | T | G | G | 0.15936 | -0.04 | 0.03 | -1.24 | 0.21543 |
| Geno.rare57 | 58 | * | * | * | * | 0.00249 | 0.67 | 0.28 | 2.41 | 0.01610 |
| haplo.base57 | 58 | T | T | C | G | 0.47950 | NA | NA | NA | NA |

hap.freq: haplotype frequency; coef: coefficient; se: standard error; t.stat: test statistic; p-val: haplotype p-value
