# Supplementary material for: Apolipoprotein E-C1-C4-C2 gene cluster region and inter-individual variation in plasma lipoprotein levels: a comprehensive genetic association study in two ethnic groups
Source: PLoS One. 2019 Mar 26;14(3):e0214060. doi: 10.1371/journal.pone.0214060 (PMC6435132; doi:10.1371/journal.pone.0214060)
Supplement: S30 Table — hap.freq: haplotype frequency; coef: coefficient; se: standard error; t.stat: test statistic; p-val: haplotype p-value; aBox-Cox transformed data. (DOCX) [file pone.0214060.s030.docx]

S30 Table. Haplotype summary of significant windows with LDL-C in ABs

| **LDL-C^a^** | | | | | | | | | | |
| --- | --- | --- | --- | --- | --- | --- | --- | --- | --- | --- |
|  | **Window** | **loc.1** | **loc.2** | **loc.3** | **loc.4** | **hap.freq** | **coef** | **se** | **t.stat** | **pval** |
| Geno.2 | 1 | C | A | C | T | 0.36494 | 0.53 | 0.20 | 2.65 | 0.00815 |
| Geno.6 | 1 | T | A | C | A | 0.06140 | -0.36 | 0.40 | -0.91 | 0.36339 |
| Geno.rare | 1 | * | * | * | * | 0.00666 | -0.25 | 1.29 | -0.20 | 0.84412 |
| haplo.base | 1 | C | A | C | A | 0.56700 | NA | NA | NA | NA |
| Geno.4 | 2 | A | C | T | G | 0.36464 | 0.59 | 0.19 | 3.01 | 0.00266 |
| Geno.rare1 | 2 | * | * | * | * | 0.00731 | -0.40 | 1.33 | -0.30 | 0.76355 |
| haplo.base1 | 2 | A | C | A | G | 0.62805 | NA | NA | NA | NA |
| Geno.61 | 3 | C | T | G | T | 0.36310 | 0.54 | 0.20 | 2.79 | 0.00547 |
| Geno.rare2 | 3 | * | * | * | * | 0.01303 | -1.01 | 0.92 | -1.10 | 0.27269 |
| haplo.base2 | 3 | C | A | G | T | 0.62387 | NA | NA | NA | NA |
| Geno.3 | 4 | A | G | T | T | 0.04221 | 0.50 | 0.50 | 1.01 | 0.31120 |
| Geno.62 | 4 | T | G | T | G | 0.15663 | 0.43 | 0.28 | 1.54 | 0.12379 |
| Geno.7 | 4 | T | G | T | T | 0.21424 | 0.67 | 0.23 | 2.88 | 0.00406 |
| Geno.rare3 | 4 | * | * | * | * | 0.00716 | -2.53 | 1.26 | -2.01 | 0.04529 |
| haplo.base3 | 4 | A | G | T | G | 0.57976 | NA | NA | NA | NA |
| Geno.5 | 5 | G | T | G | T | 0.04142 | 0.24 | 0.46 | 0.53 | 0.59901 |
| Geno.63 | 5 | G | T | T | C | 0.25707 | 0.58 | 0.21 | 2.76 | 0.00592 |
| Geno.rare4 | 5 | * | * | * | * | 0.00715 | -2.71 | 1.27 | -2.13 | 0.03354 |
| haplo.base4 | 5 | G | T | G | C | 0.69436 | NA | NA | NA | NA |
| Geno.51 | 6 | T | G | T | G | 0.04209 | 0.19 | 0.46 | 0.41 | 0.67890 |
| Geno.64 | 6 | T | T | C | C | 0.09836 | 0.65 | 0.32 | 2.00 | 0.04579 |
| Geno.71 | 6 | T | T | C | G | 0.15818 | 0.53 | 0.26 | 2.01 | 0.04437 |
| Geno.rare5 | 6 | * | * | * | * | 0.01387 | -1.83 | 0.89 | -2.05 | 0.04074 |
| haplo.base5 | 6 | T | G | C | G | 0.68751 | NA | NA | NA | NA |
| Geno.25 | 11 | A | A | G | G | 0.05634 | -0.19 | 0.41 | -0.47 | 0.64066 |
| Geno.54 | 11 | C | A | G | A | 0.01593 | -2.06 | 0.79 | -2.59 | 0.00971 |
| Geno.rare10 | 11 | * | * | * | * | 0.01631 | -0.93 | 0.77 | -1.21 | 0.22486 |
| haplo.base10 | 11 | C | A | G | G | 0.91142 | NA | NA | NA | NA |
| Geno.32 | 12 | A | G | A | G | 0.01681 | -2.00 | 0.75 | -2.69 | 0.00738 |
| Geno.43 | 12 | A | G | G | A | 0.38633 | 0.09 | 0.20 | 0.42 | 0.67194 |
| Geno.rare11 | 12 | * | * | * | * | 0.01549 | -0.81 | 0.74 | -1.09 | 0.27669 |
| haplo.base11 | 12 | A | G | G | G | 0.58137 | NA | NA | NA | NA |
| Geno.33 | 13 | G | A | G | C | 0.01681 | -1.98 | 0.75 | -2.66 | 0.00802 |
| Geno.44 | 13 | G | G | A | C | 0.38655 | 0.10 | 0.20 | 0.49 | 0.62637 |
| Geno.rare12 | 13 | * | * | * | * | 0.01405 | -0.44 | 0.82 | -0.54 | 0.59146 |
| haplo.base12 | 13 | G | G | G | C | 0.58259 | NA | NA | NA | NA |
| Geno.26 | 14 | A | G | C | T | 0.01682 | -2.12 | 0.74 | -2.84 | 0.00462 |
| Geno.55 | 14 | G | G | C | C | 0.26690 | 0.23 | 0.23 | 0.98 | 0.32691 |
| Geno.66 | 14 | G | G | C | T | 0.32732 | -0.43 | 0.23 | -1.85 | 0.06475 |
| Geno.rare13 | 14 | * | * | * | * | 0.00712 | -0.43 | 1.17 | -0.37 | 0.71146 |
| haplo.base13 | 14 | G | A | C | T | 0.38185 | NA | NA | NA | NA |
| Geno.34 | 15 | G | C | C | C | 0.26692 | 0.22 | 0.23 | 0.94 | 0.34618 |
| Geno.56 | 15 | G | C | T | C | 0.32485 | -0.41 | 0.23 | -1.78 | 0.07500 |
| Geno.67 | 15 | G | C | T | T | 0.01952 | -2.28 | 0.68 | -3.37 | 0.00080 |
| Geno.rare14 | 15 | * | * | * | * | 0.00712 | -0.45 | 1.17 | -0.38 | 0.70263 |
| haplo.base14 | 15 | A | C | T | C | 0.38160 | NA | NA | NA | NA |
| Geno.11 | 16 | C | C | C | G | 0.26743 | 0.22 | 0.21 | 1.04 | 0.29819 |
| Geno.35 | 16 | C | T | C | A | 0.05813 | -2.05 | 0.40 | -5.08 | 4.65E-07 |
| Geno.57 | 16 | C | T | T | G | 0.01984 | -2.47 | 0.67 | -3.69 | 0.00024 |
| Geno.rare15 | 16 | * | * | * | * | 0.00601 | -0.43 | 1.24 | -0.35 | 0.72988 |
| haplo.base15 | 16 | C | T | C | G | 0.64859 | NA | NA | NA | NA |
| Geno.12 | 17 | C | C | G | G | 0.26686 | 0.19 | 0.21 | 0.92 | 0.35766 |
| Geno.45 | 17 | T | C | A | G | 0.05813 | -2.06 | 0.40 | -5.11 | 4.14E-07 |
| Geno.68 | 17 | T | T | G | G | 0.01983 | -2.47 | 0.67 | -3.69 | 0.00024 |
| haplo.base16 | 17 | T | C | G | G | 0.65451 | NA | NA | NA | NA |
| Geno.13 | 18 | C | A | G | C | 0.05810 | -2.14 | 0.40 | -5.39 | 9.62E-08 |
| Geno.69 | 18 | T | G | G | C | 0.01979 | -2.51 | 0.67 | -3.78 | 0.00017 |
| Geno.rare16 | 18 | * | * | * | * | 0.00589 | -1.35 | 1.28 | -1.06 | 0.28994 |
| haplo.base17 | 18 | C | G | G | C | 0.91623 | NA | NA | NA | NA |
| Geno.27 | 19 | A | G | C | T | 0.05817 | -2.04 | 0.40 | -5.09 | 4.44E-07 |
| Geno.36 | 19 | G | G | C | G | 0.02629 | -0.16 | 0.56 | -0.28 | 0.77761 |
| Geno.rare17 | 19 | * | * | * | * | 0.00589 | -1.25 | 1.37 | -0.91 | 0.36338 |
| haplo.base18 | 19 | G | G | C | T | 0.90966 | NA | NA | NA | NA |
| Geno.16 | 24 | A | I | C | C | 0.13261 | -0.32 | 0.30 | -1.08 | 0.28098 |
| Geno.38 | 24 | A | W | C | C | 0.16401 | 0.27 | 0.31 | 0.90 | 0.36953 |
| Geno.59 | 24 | G | I | C | C | 0.13751 | -0.61 | 0.32 | -1.89 | 0.05977 |
| Geno.82 | 24 | G | W | C | T | 0.02495 | 1.66 | 0.70 | 2.39 | 0.01713 |
| Geno.rare22 | 24 | * | * | * | * | 0.00746 | -2.54 | 1.33 | -1.91 | 0.05649 |
| haplo.base23 | 24 | G | W | C | C | 0.53345 | NA | NA | NA | NA |
| Geno.17 | 25 | I | C | C | G | 0.27035 | -0.49 | 0.22 | -2.27 | 0.02351 |
| Geno.39 | 25 | W | C | C | A | 0.06631 | 0.40 | 0.41 | 0.99 | 0.32384 |
| Geno.611 | 25 | W | C | T | G | 0.02898 | 0.34 | 0.57 | 0.60 | 0.54773 |
| Geno.rare23 | 25 | * | * | * | * | 0.00335 | 2.08 | 1.98 | 1.05 | 0.29342 |
| haplo.base24 | 25 | W | C | C | G | 0.63100 | NA | NA | NA | NA |
| Geno.113 | 35 | G | D | A | G | 0.21977 | 0.27 | 0.23 | 1.14 | 0.25378 |
| Geno.315 | 35 | G | W | A | A | 0.06231 | 0.78 | 0.41 | 1.88 | 0.06102 |
| Geno.514 | 35 | G | W | G | A | 0.03311 | -1.36 | 0.60 | -2.28 | 0.02300 |
| Geno.615 | 35 | G | W | G | G | 0.02413 | -0.23 | 0.68 | -0.34 | 0.73547 |
| Geno.78 | 35 | T | W | G | A | 0.08798 | -0.44 | 0.34 | -1.30 | 0.19301 |
| haplo.base34 | 35 | G | W | A | G | 0.57271 | NA | NA | NA | NA |
| Geno.215 | 36 | D | A | G | G | 0.21908 | 0.29 | 0.23 | 1.23 | 0.21764 |
| Geno.414 | 36 | W | A | A | C | 0.06120 | 0.85 | 0.42 | 2.01 | 0.04461 |
| Geno.616 | 36 | W | A | G | G | 0.05080 | 0.30 | 0.42 | 0.69 | 0.48774 |
| Geno.79 | 36 | W | G | A | C | 0.12309 | -0.69 | 0.31 | -2.24 | 0.02534 |
| Geno.9 | 36 | W | G | G | C | 0.02412 | -0.13 | 0.71 | -0.18 | 0.85506 |
| haplo.base35 | 36 | W | A | G | C | 0.52097 | NA | NA | NA | NA |
| Geno.114 | 37 | A | A | C | G | 0.06082 | 0.86 | 0.42 | 2.03 | 0.04284 |
| Geno.515 | 37 | A | G | G | G | 0.26924 | 0.34 | 0.22 | 1.54 | 0.12471 |
| Geno.710 | 37 | G | A | C | G | 0.12049 | -0.69 | 0.31 | -2.23 | 0.02617 |
| Geno.91 | 37 | G | G | C | G | 0.02338 | -0.03 | 0.71 | -0.04 | 0.96558 |
| Geno.rare31 | 37 | * | * | * | * | 0.00738 | -0.48 | 1.17 | -0.41 | 0.68376 |
| haplo.base36 | 37 | A | G | C | G | 0.51869 | NA | NA | NA | NA |
| Geno.96 | 75 | G | C | A | G | 0.02667 | -1.52 | 0.64 | -2.35 | 0.01879 |
| Geno.105 | 75 | G | C | G | C | 0.24608 | 0.40 | 0.23 | 1.79 | 0.07334 |
| Geno.rare67 | 75 | * | * | * | * | 0.02585 | 0.31 | 0.67 | 0.45 | 0.64964 |
| haplo.base74 | 75 | G | C | G | G | 0.70140 | NA | NA | NA | NA |
| Geno.428 | 76 | C | A | G | G | 0.02498 | -1.46 | 0.67 | -2.18 | 0.02972 |
| Geno.534 | 76 | C | G | C | A | 0.09245 | 0.67 | 0.35 | 1.93 | 0.05382 |
| Geno.633 | 76 | C | G | C | G | 0.15584 | 0.22 | 0.28 | 0.80 | 0.42320 |
| Geno.124 | 76 | T | G | G | G | 0.01110 | -0.39 | 0.98 | -0.40 | 0.69145 |
| Geno.rare68 | 76 | * | * | * | * | 0.01745 | 0.98 | 0.87 | 1.13 | 0.25729 |
| haplo.base75 | 76 | C | G | G | G | 0.69817 | NA | NA | NA | NA |
| Geno.120 | 78 | C | A | G | C | 0.10388 | 0.56 | 0.34 | 1.67 | 0.09628 |
| Geno.229 | 78 | C | G | A | C | 0.05838 | -0.17 | 0.49 | -0.35 | 0.72687 |
| Geno.430 | 78 | C | G | G | C | 0.09877 | 0.30 | 0.41 | 0.75 | 0.45603 |
| Geno.720 | 78 | G | G | A | C | 0.28994 | -0.47 | 0.26 | -1.84 | 0.06630 |
| Geno.818 | 78 | G | G | A | G | 0.01323 | 0.91 | 0.85 | 1.07 | 0.28551 |
| Geno.rare70 | 78 | * | * | * | * | 0.00422 | 1.74 | 1.62 | 1.07 | 0.28505 |
| haplo.base77 | 78 | G | G | G | C | 0.43159 | NA | NA | NA | NA |
| Geno.330 | 79 | A | G | C | A | 0.10305 | 0.57 | 0.35 | 1.64 | 0.10223 |
| Geno.634 | 79 | G | A | C | A | 0.33439 | -0.47 | 0.24 | -1.99 | 0.04692 |
| Geno.721 | 79 | G | A | C | G | 0.01326 | -0.83 | 1.00 | -0.83 | 0.40439 |
| Geno.819 | 79 | G | A | G | A | 0.01314 | 0.85 | 0.85 | 1.00 | 0.31762 |
| Geno.106 | 79 | G | G | C | G | 0.11232 | 0.01 | 0.35 | 0.04 | 0.96931 |
| Geno.rare71 | 79 | * | * | * | * | 0.00495 | 0.83 | 1.78 | 0.47 | 0.64025 |
| haplo.base78 | 79 | G | G | C | A | 0.41889 | NA | NA | NA | NA |
| Geno.126 | 80 | A | C | A | G | 0.01486 | 0.26 | 0.86 | 0.31 | 0.75976 |
| Geno.230 | 80 | A | C | A | T | 0.31966 | -0.62 | 0.22 | -2.76 | 0.00593 |
| Geno.331 | 80 | A | C | G | T | 0.01588 | -0.92 | 0.91 | -1.01 | 0.31257 |
| Geno.535 | 80 | A | G | A | T | 0.01202 | 1.12 | 0.90 | 1.24 | 0.21690 |
| Geno.98 | 80 | G | C | G | T | 0.10905 | 0.02 | 0.34 | 0.07 | 0.94184 |
| Geno.rare72 | 80 | * | * | * | * | 0.00261 | -4.64 | 2.06 | -2.25 | 0.02474 |
| haplo.base79 | 80 | G | C | A | T | 0.52593 | NA | NA | NA | NA |
| Geno.130 | 84 | A | C | G | C | 0.30079 | -0.07 | 0.23 | -0.28 | 0.78159 |
| Geno.537 | 84 | A | G | T | C | 0.13216 | 0.52 | 0.31 | 1.69 | 0.09062 |
| Geno.638 | 84 | A | G | T | T | 0.03586 | -0.01 | 0.52 | -0.02 | 0.98253 |
| Geno.820 | 84 | T | C | T | C | 0.18380 | 0.65 | 0.27 | 2.41 | 0.01630 |
| haplo.base83 | 84 | A | C | T | C | 0.34740 | NA | NA | NA | NA |
| Geno.436 | 95 | W | C | W | T | 0.30785 | 0.31 | 0.24 | 1.27 | 0.20455 |
| Geno.543 | 95 | W | T | D | G | 0.03499 | 0.77 | 0.52 | 1.48 | 0.13834 |
| Geno.640 | 95 | W | T | W | G | 0.24705 | -0.30 | 0.25 | -1.18 | 0.23870 |
| Geno.rare80 | 95 | * | * | * | * | 0.00896 | -1.53 | 1.02 | -1.50 | 0.13373 |
| haplo.base94 | 95 | W | C | W | G | 0.40115 | NA | NA | NA | NA |
| Geno.234 | 96 | C | W | G | A | 0.25779 | -0.22 | 0.26 | -0.86 | 0.39099 |
| Geno.334 | 96 | C | W | G | G | 0.13634 | -0.73 | 0.33 | -2.24 | 0.02561 |
| Geno.641 | 96 | T | D | G | G | 0.03499 | 0.38 | 0.53 | 0.72 | 0.47241 |
| Geno.823 | 96 | T | W | G | G | 0.25475 | -0.70 | 0.26 | -2.69 | 0.00733 |
| Geno.rare81 | 96 | * | * | * | * | 0.00332 | -0.67 | 1.63 | -0.41 | 0.68042 |
| haplo.base95 | 96 | C | W | T | G | 0.31282 | NA | NA | NA | NA |
| Geno.139 | 97 | D | G | G | C | 0.03491 | 1.05 | 0.52 | 2.01 | 0.04463 |
| Geno.335 | 97 | W | G | A | C | 0.25752 | 0.46 | 0.24 | 1.88 | 0.06011 |
| Geno.824 | 97 | W | T | G | C | 0.30988 | 0.62 | 0.24 | 2.57 | 0.01042 |
| haplo.base96 | 97 | W | G | G | C | 0.39702 | NA | NA | NA | NA |
| Geno.143 | 102 | C | T | A | A | 0.01626 | -0.21 | 0.78 | -0.27 | 0.78644 |
| Geno.338 | 102 | G | G | A | A | 0.10007 | -0.30 | 0.33 | -0.91 | 0.36180 |
| Geno.545 | 102 | G | T | A | G | 0.07827 | 1.11 | 0.38 | 2.95 | 0.00323 |
| Geno.643 | 102 | G | T | G | A | 0.31767 | 0.49 | 0.22 | 2.23 | 0.02630 |
| haplo.base101 | 102 | G | T | A | A | 0.48750 | NA | NA | NA | NA |
| Geno.144 | 103 | G | A | A | G | 0.10014 | -0.27 | 0.33 | -0.84 | 0.40117 |
| Geno.728 | 103 | T | A | G | G | 0.07769 | 1.15 | 0.37 | 3.08 | 0.00218 |
| Geno.826 | 103 | T | G | A | G | 0.31685 | 0.52 | 0.22 | 2.41 | 0.01626 |
| Geno.rare86 | 103 | * | * | * | * | 0.00134 | 3.22 | 0.00 | 3.01E+16 | <10E-06 |
| haplo.base102 | 103 | T | A | A | G | 0.50398 | NA | NA | NA | NA |
| Geno.644 | 104 | A | G | G | G | 0.07743 | 1.23 | 0.37 | 3.33 | 0.00090 |
| Geno.827 | 104 | G | A | G | G | 0.31638 | 0.60 | 0.21 | 2.86 | 0.00430 |
| Geno.rare87 | 104 | * | * | * | * | 0.01120 | 2.19 | 0.95 | 2.30 | 0.02184 |
| haplo.base103 | 104 | A | A | G | G | 0.59500 | NA | NA | NA | NA |

hap.freq: haplotype frequency; coef: coefficient; se: standard error; t.stat: test statistic; p-val: haplotype p-value; ^a^Box-Cox transformed data.
