# Supplementary material for: Apolipoprotein E-C1-C4-C2 gene cluster region and inter-individual variation in plasma lipoprotein levels: a comprehensive genetic association study in two ethnic groups
Source: PLoS One. 2019 Mar 26;14(3):e0214060. doi: 10.1371/journal.pone.0214060 (PMC6435132; doi:10.1371/journal.pone.0214060)
Supplement: S31 Table — hap.freq: haplotype frequency; coef: coefficient; se: standard error; t.stat: test statistic; p-val: haplotype p-value; aBox-Cox transformed data. (DOCX) [file pone.0214060.s031.docx]

S31 Table. Haplotype summary of significant windows with TC in ABs

| **TC** | | | | | | | | | | |
| --- | --- | --- | --- | --- | --- | --- | --- | --- | --- | --- |
|  | **Window** | **loc.1** | **loc.2** | **loc.3** | **loc.4** | **hap.freq** | **coef** | **se** | **t.stat** | **pval** |
| Geno.4 | 2 | A | C | T | G | 0.36241 | 0.24 | 0.10 | 2.38 | 0.01764 |
| Geno.rare1 | 2 | * | * | * | * | 0.00805 | -0.26 | 0.59 | -0.45 | 0.65610 |
| haplo.base1 | 2 | A | C | A | G | 0.62954 | NA | NA | NA | NA |
| Geno.41 | 3 | C | T | G | T | 0.36188 | 0.23 | 0.10 | 2.23 | 0.02597 |
| Geno.rare2 | 3 | * | * | * | * | 0.01388 | -0.42 | 0.45 | -0.95 | 0.34462 |
| haplo.base2 | 3 | C | A | G | T | 0.62424 | NA | NA | NA | NA |
| Geno.3 | 4 | A | G | T | T | 0.04244 | 0.01 | 0.26 | 0.02 | 0.98317 |
| Geno.61 | 4 | T | G | T | G | 0.15540 | 0.17 | 0.15 | 1.15 | 0.25134 |
| Geno.7 | 4 | T | G | T | T | 0.21408 | 0.28 | 0.12 | 2.30 | 0.02149 |
| Geno.rare3 | 4 | * | * | * | * | 0.00792 | -1.33 | 0.61 | -2.17 | 0.03002 |
| haplo.base3 | 4 | A | G | T | G | 0.58017 | NA | NA | NA | NA |
| Geno.5 | 5 | G | T | G | T | 0.04119 | 0.22 | 0.24 | 0.90 | 0.36837 |
| Geno.62 | 5 | G | T | T | C | 0.25711 | 0.22 | 0.11 | 1.94 | 0.05312 |
| Geno.rare4 | 5 | * | * | * | * | 0.00790 | -1.39 | 0.62 | -2.24 | 0.02508 |
| haplo.base4 | 5 | G | T | G | C | 0.69380 | NA | NA | NA | NA |
| Geno.34 | 15 | G | C | C | C | 0.26783 | 0.06 | 0.12 | 0.49 | 0.62327 |
| Geno.46 | 15 | G | C | T | C | 0.32360 | -0.19 | 0.12 | -1.55 | 0.12041 |
| Geno.55 | 15 | G | C | T | T | 0.02047 | -0.91 | 0.35 | -2.58 | 0.00995 |
| Geno.rare13 | 15 | * | * | * | * | 0.00787 | -0.10 | 0.59 | -0.16 | 0.87005 |
| haplo.base14 | 15 | A | C | T | C | 0.38023 | NA | NA | NA | NA |
| Geno.11 | 16 | C | C | C | G | 0.26736 | 0.07 | 0.11 | 0.64 | 0.52426 |
| Geno.35 | 16 | C | T | C | A | 0.05863 | -0.82 | 0.21 | -3.89 | 0.00011 |
| Geno.56 | 16 | C | T | T | G | 0.02069 | -0.96 | 0.35 | -2.77 | 0.00573 |
| Geno.rare14 | 16 | * | * | * | * | 0.00676 | -0.13 | 0.62 | -0.21 | 0.83225 |
| haplo.base15 | 16 | C | T | C | G | 0.64657 | NA | NA | NA | NA |
| Geno.12 | 17 | C | C | G | G | 0.26775 | 0.06 | 0.11 | 0.53 | 0.59942 |
| Geno.47 | 17 | T | C | A | G | 0.05881 | -0.83 | 0.21 | -3.91 | 0.00010 |
| Geno.66 | 17 | T | T | G | G | 0.02068 | -0.96 | 0.35 | -2.78 | 0.00562 |
| haplo.base16 | 17 | T | C | G | G | 0.65208 | NA | NA | NA | NA |
| Geno.13 | 18 | C | A | G | C | 0.05876 | -0.85 | 0.21 | -4.08 | 4.99E-05 |
| Geno.67 | 18 | T | G | G | C | 0.02063 | -0.97 | 0.34 | -2.81 | 0.00515 |
| Geno.rare15 | 18 | * | * | * | * | 0.00595 | -0.40 | 0.68 | -0.59 | 0.55242 |
| haplo.base17 | 18 | C | G | G | C | 0.91465 | NA | NA | NA | NA |
| Geno.27 | 19 | A | G | C | T | 0.05883 | -0.81 | 0.21 | -3.88 | 0.00011 |
| Geno.36 | 19 | G | G | C | G | 0.02521 | -0.05 | 0.31 | -0.16 | 0.87518 |
| Geno.rare16 | 19 | * | * | * | * | 0.00595 | -0.36 | 0.72 | -0.50 | 0.61882 |
| haplo.base18 | 19 | G | G | C | T | 0.91000 | NA | NA | NA | NA |
| Geno.95 | 75 | G | C | A | G | 0.02577 | -0.78 | 0.34 | -2.30 | 0.02153 |
| Geno.105 | 75 | G | C | G | C | 0.24511 | 0.23 | 0.12 | 1.94 | 0.05331 |
| Geno.rare65 | 75 | * | * | * | * | 0.02514 | 0.25 | 0.36 | 0.70 | 0.48596 |
| haplo.base74 | 75 | G | C | G | G | 0.70398 | NA | NA | NA | NA |
| Geno.431 | 76 | C | A | G | G | 0.02411 | -0.72 | 0.35 | -2.07 | 0.03884 |
| Geno.534 | 76 | C | G | C | A | 0.09378 | 0.37 | 0.18 | 2.01 | 0.04514 |
| Geno.629 | 76 | C | G | C | G | 0.15381 | 0.14 | 0.15 | 0.92 | 0.35750 |
| Geno.125 | 76 | T | G | G | G | 0.01014 | -0.05 | 0.56 | -0.09 | 0.93140 |
| Geno.rare66 | 76 | * | * | * | * | 0.01766 | 0.50 | 0.47 | 1.07 | 0.28583 |
| haplo.base75 | 76 | C | G | G | G | 0.70051 | NA | NA | NA | NA |
| Geno.145 | 102 | C | T | A | A | 0.01803 | -0.12 | 0.39 | -0.30 | 0.76200 |
| Geno.337 | 102 | G | G | A | A | 0.10047 | -0.10 | 0.17 | -0.57 | 0.56761 |
| Geno.545 | 102 | G | T | A | G | 0.08052 | 0.54 | 0.20 | 2.75 | 0.00608 |
| Geno.639 | 102 | G | T | G | A | 0.31430 | 0.22 | 0.12 | 1.87 | 0.06166 |
| haplo.base101 | 102 | G | T | A | A | 0.48668 | NA | NA | NA | NA |
| Geno.146 | 103 | G | A | A | G | 0.10052 | -0.08 | 0.17 | -0.47 | 0.64169 |
| Geno.731 | 103 | T | A | G | G | 0.07968 | 0.57 | 0.20 | 2.90 | 0.00382 |
| Geno.825 | 103 | T | G | A | G | 0.31348 | 0.24 | 0.12 | 2.08 | 0.03791 |
| Geno.rare85 | 103 | * | * | * | * | 0.00136 | 1.80 | NA | NA | NA |
| haplo.base102 | 103 | T | A | A | G | 0.50497 | NA | NA | NA | NA |
| Geno.640 | 104 | A | G | G | G | 0.07934 | 0.60 | 0.19 | 3.10 | 0.00199 |
| Geno.826 | 104 | G | A | G | G | 0.31300 | 0.27 | 0.11 | 2.41 | 0.01617 |
| Geno.rare86 | 104 | * | * | * | * | 0.01132 | 1.16 | 0.50 | 2.31 | 0.02114 |
| haplo.base103 | 104 | A | A | G | G | 0.59634 | NA | NA | NA | NA |

hap.freq: haplotype frequency; coef: coefficient; se: standard error; t.stat: test statistic; p-val: haplotype p-value; ^a^Box-Cox transformed data.
