# Supplementary material for: Apolipoprotein E-C1-C4-C2 gene cluster region and inter-individual variation in plasma lipoprotein levels: a comprehensive genetic association study in two ethnic groups
Source: PLoS One. 2019 Mar 26;14(3):e0214060. doi: 10.1371/journal.pone.0214060 (PMC6435132; doi:10.1371/journal.pone.0214060)
Supplement: S32 Table — hap.freq: haplotype frequency; coef: coefficient; se: standard error; t.stat: test statistic; p-val: haplotype p-value; aBox-Cox transformed data. (DOCX) [file pone.0214060.s032.docx]

S32 Table. Haplotype summary of significant windows with ApoB in Blacks

| **ApoB** | | | | | | | | | | |
| --- | --- | --- | --- | --- | --- | --- | --- | --- | --- | --- |
|  | **Window** | **loc.1** | **loc.2** | **loc.3** | **loc.4** | **hap.freq** | **coef** | **se** | **t.stat** | **pval** |
| Geno.12 | 17 | C | C | G | G | 0.26517 | -0.30 | 0.60 | -0.50 | 0.61601 |
| Geno.47 | 17 | T | C | A | G | 0.05926 | -2.59 | 1.13 | -2.29 | 0.02206 |
| Geno.67 | 17 | T | T | G | G | 0.01984 | -3.83 | 1.90 | -2.01 | 0.04480 |
| haplo.base16 | 17 | T | C | G | G | 0.65506 | NA | NA | NA | NA |
| Geno.13 | 18 | C | A | G | C | 0.05921 | -2.51 | 1.11 | -2.25 | 0.02478 |
| Geno.56 | 18 | T | G | G | C | 0.01981 | -3.80 | 1.91 | -1.99 | 0.04669 |
| Geno.rare16 | 18 | * | * | * | * | 0.00522 | -0.89 | 3.90 | -0.23 | 0.81963 |
| haplo.base17 | 18 | C | G | G | C | 0.91576 | NA | NA | NA | NA |

hap.freq: haplotype frequency; coef: coefficient; se: standard error; t.stat: test statistic; p-val: haplotype p-value; ^a^Box-Cox transformed data
