# Supplementary material for: Apolipoprotein E-C1-C4-C2 gene cluster region and inter-individual variation in plasma lipoprotein levels: a comprehensive genetic association study in two ethnic groups
Source: PLoS One. 2019 Mar 26;14(3):e0214060. doi: 10.1371/journal.pone.0214060 (PMC6435132; doi:10.1371/journal.pone.0214060)
Supplement: S33 Table — hap.freq: haplotype frequency; coef: coefficient; se: standard error; t.stat: test statistic; p-val: haplotype p-value; aBox-Cox transformed data. (DOCX) [file pone.0214060.s033.docx]

S33 Table. Haplotype summary of significant windows with HDL-C in Blacks

| **HDL-C^a^** | | | | | | | | | | |
| --- | --- | --- | --- | --- | --- | --- | --- | --- | --- | --- |
|  | **Window** | **loc.1** | **loc.2** | **loc.3** | **loc.4** | **hap.freq** | **coef** | **se** | **t.stat** | **pval** |
| Geno.419 | 41 | A | C | G | G | 0.02611 | 1.08 | 0.80 | 1.35 | 0.17657 |
| Geno.514 | 41 | A | G | C | C | 0.18718 | 1.07 | 0.32 | 3.35 | 0.00085 |
| Geno.616 | 41 | A | G | C | G | 0.02454 | -1.57 | 0.85 | -1.84 | 0.06554 |
| Geno.714 | 41 | A | G | G | C | 0.09375 | -0.05 | 0.40 | -0.12 | 0.90227 |
| Geno.88 | 41 | A | G | G | G | 0.03745 | 2.07 | 0.59 | 3.49 | 0.00051 |
| Geno.102 | 41 | G | C | G | C | 0.18672 | 0.50 | 0.34 | 1.46 | 0.14443 |
| Geno.115 | 41 | G | C | G | G | 0.03822 | 1.67 | 0.68 | 2.45 | 0.01450 |
| Geno.122 | 41 | G | G | C | C | 0.05652 | 0.76 | 0.55 | 1.37 | 0.16992 |
| Geno.132 | 41 | G | G | C | G | 0.01845 | 0.84 | 1.13 | 0.75 | 0.45618 |
| Geno.141 | 41 | G | G | G | C | 0.05064 | 0.65 | 0.53 | 1.22 | 0.22153 |
| Geno.151 | 41 | G | G | G | G | 0.01026 | -3.70 | 1.65 | -2.24 | 0.02544 |
| Geno.rare34 | 41 | * | * | * | * | 0.01185 | -1.66 | 0.98 | -1.69 | 0.09063 |
| haplo.base40 | 41 | A | C | G | C | 0.25833 | NA | NA | NA | NA |
| Geno.116 | 42 | C | C | C | G | 0.01090 | -2.01 | 1.03 | -1.96 | 0.05006 |
| Geno.315 | 42 | C | G | C | A | 0.01966 | -1.15 | 0.81 | -1.42 | 0.15565 |
| Geno.617 | 42 | C | G | G | G | 0.05760 | 1.38 | 0.53 | 2.62 | 0.00893 |
| Geno.715 | 42 | G | C | C | A | 0.01106 | 1.19 | 1.25 | 0.96 | 0.33898 |
| Geno.89 | 42 | G | C | C | G | 0.23549 | 0.76 | 0.25 | 3.11 | 0.00195 |
| Geno.103 | 42 | G | C | G | G | 0.04107 | -1.41 | 0.57 | -2.47 | 0.01386 |
| Geno.123 | 42 | G | G | C | G | 0.13707 | -0.13 | 0.32 | -0.41 | 0.68023 |
| Geno.142 | 42 | G | G | G | G | 0.04596 | 1.09 | 0.59 | 1.84 | 0.06579 |
| Geno.rare35 | 42 | * | * | * | * | 0.01643 | -0.71 | 1.09 | -0.65 | 0.51705 |
| haplo.base41 | 42 | C | G | C | G | 0.42477 | NA | NA | NA | NA |
| Geno.117 | 43 | C | C | A | C | 0.01232 | 1.01 | 1.12 | 0.90 | 0.36770 |
| Geno.218 | 43 | C | C | G | C | 0.24347 | 0.67 | 0.23 | 2.98 | 0.00300 |
| Geno.515 | 43 | C | G | G | C | 0.04040 | -1.41 | 0.57 | -2.46 | 0.01431 |
| Geno.618 | 43 | G | C | A | C | 0.02501 | -1.13 | 0.64 | -1.76 | 0.07895 |
| Geno.104 | 43 | G | G | G | C | 0.10411 | 1.25 | 0.34 | 3.68 | 0.00025 |
| Geno.rare36 | 43 | * | * | * | * | 0.01264 | -0.05 | 1.00 | -0.05 | 0.96371 |
| haplo.base42 | 43 | G | C | G | C | 0.56205 | NA | NA | NA | NA |

hap.freq: haplotype frequency; coef: coefficient; se: standard error; t.stat: test statistic; p-val: haplotype p-value; ^a^Box-Cox transformed data.
