# Supplementary material for: Apolipoprotein E-C1-C4-C2 gene cluster region and inter-individual variation in plasma lipoprotein levels: a comprehensive genetic association study in two ethnic groups
Source: PLoS One. 2019 Mar 26;14(3):e0214060. doi: 10.1371/journal.pone.0214060 (PMC6435132; doi:10.1371/journal.pone.0214060)
Supplement: S34 Table — hap.freq: haplotype frequency; coef: coefficient; se: standard error; t.stat: test statistic; p-val: haplotype p-value; aBox-Cox transformed data. (DOCX) [file pone.0214060.s034.docx]

S34 Table. Haplotype summary of significant windows with apoA1 in Blacks

| **ApoA1^a^** | | | | | | | | | | |
| --- | --- | --- | --- | --- | --- | --- | --- | --- | --- | --- |
|  | **Window** | **loc.1** | **loc.2** | **loc.3** | **loc.4** | **hap.freq** | **coef** | **se** | **t.stat** | **pval** |
| Geno.11 | 16 | C | C | C | G | 0.26407 | -0.69 | 0.53 | -1.30 | 0.19546 |
| Geno.35 | 16 | C | T | C | A | 0.06005 | 3.63 | 0.98 | 3.69 | 0.00024 |
| Geno.56 | 16 | C | T | T | G | 0.02054 | -0.56 | 1.65 | -0.34 | 0.73524 |
| Geno.rare15 | 16 | * | * | * | * | 0.00672 | 3.80 | 2.88 | 1.32 | 0.18695 |
| haplo.base15 | 16 | C | T | C | G | 0.64862 | NA | NA | NA | NA |
| Geno.12 | 17 | C | C | G | G | 0.26448 | -0.75 | 0.53 | -1.41 | 0.16024 |
| Geno.47 | 17 | T | C | A | G | 0.06004 | 3.60 | 0.98 | 3.66 | 0.00027 |
| Geno.66 | 17 | T | T | G | G | 0.02054 | -0.63 | 1.65 | -0.38 | 0.70465 |
| haplo.base16 | 17 | T | C | G | G | 0.65427 | NA | NA | NA | NA |
| Geno.13 | 18 | C | A | G | C | 0.05996 | 3.87 | 0.97 | 3.98 | 7.50E-05 |
| Geno.57 | 18 | T | G | G | C | 0.02049 | -0.29 | 1.65 | -0.18 | 0.85797 |
| Geno.rare16 | 18 | * | * | * | * | 0.00522 | 3.55 | 3.42 | 1.04 | 0.29928 |
| haplo.base17 | 18 | C | G | G | C | 0.91433 | NA | NA | NA | NA |
| Geno.27 | 19 | A | G | C | T | 0.06000 | 3.89 | 0.97 | 4.01 | 6.81E-05 |
| Geno.36 | 19 | G | G | C | G | 0.02702 | 0.20 | 1.39 | 0.14 | 0.88674 |
| Geno.rare17 | 19 | * | * | * | * | 0.00522 | 3.57 | 3.64 | 0.98 | 0.32651 |
| haplo.base18 | 19 | G | G | C | T | 0.90775 | NA | NA | NA | NA |
| Geno.418 | 41 | A | C | G | G | 0.02893 | 3.56 | 2.18 | 1.64 | 0.10196 |
| Geno.516 | 41 | A | G | C | C | 0.19772 | 2.48 | 0.78 | 3.17 | 0.00160 |
| Geno.616 | 41 | A | G | C | G | 0.02261 | -1.02 | 2.11 | -0.48 | 0.62939 |
| Geno.712 | 41 | A | G | G | C | 0.09638 | -0.07 | 1.08 | -0.06 | 0.94838 |
| Geno.89 | 41 | A | G | G | G | 0.03512 | 2.90 | 1.70 | 1.71 | 0.08848 |
| Geno.102 | 41 | G | C | G | C | 0.18825 | 0.34 | 0.92 | 0.37 | 0.71262 |
| Geno.116 | 41 | G | C | G | G | 0.03502 | 1.79 | 1.85 | 0.97 | 0.33243 |
| Geno.122 | 41 | G | G | C | C | 0.05623 | -1.20 | 1.32 | -0.91 | 0.36309 |
| Geno.132 | 41 | G | G | C | G | 0.02027 | 1.27 | 2.58 | 0.49 | 0.62174 |
| Geno.141 | 41 | G | G | G | C | 0.04830 | 2.02 | 1.46 | 1.38 | 0.16697 |
| Geno.151 | 41 | G | G | G | G | 0.01045 | -4.83 | 4.01 | -1.20 | 0.22894 |
| Geno.rare35 | 41 | * | * | * | * | 0.01112 | -3.08 | 2.43 | -1.27 | 0.20511 |
| haplo.base40 | 41 | A | C | G | C | 0.24961 | NA | NA | NA | NA |
| Geno.118 | 43 | C | C | A | C | 0.01193 | 5.27 | 2.82 | 1.87 | 0.06164 |
| Geno.217 | 43 | C | C | G | C | 0.25124 | 1.08 | 0.56 | 1.92 | 0.05535 |
| Geno.517 | 43 | C | G | G | C | 0.04096 | -0.97 | 1.42 | -0.68 | 0.49511 |
| Geno.618 | 43 | G | C | A | C | 0.02438 | -3.38 | 1.61 | -2.10 | 0.03593 |
| Geno.104 | 43 | G | G | G | C | 0.10014 | 1.85 | 0.89 | 2.08 | 0.03812 |
| Geno.rare37 | 43 | * | * | * | * | 0.01309 | 2.12 | 2.64 | 0.80 | 0.42278 |
| haplo.base42 | 43 | G | C | G | C | 0.55826 | NA | NA | NA | NA |
| Geno.119 | 54 | I | G | G | C | 0.03369 | 2.50 | 1.36 | 1.84 | 0.06673 |
| Geno.319 | 54 | W | G | A | C | 0.01382 | -2.46 | 2.00 | -1.23 | 0.21953 |
| Geno.526 | 54 | W | G | G | T | 0.09637 | -1.33 | 0.80 | -1.66 | 0.09749 |
| Geno.623 | 54 | W | T | G | C | 0.01381 | 2.85 | 2.01 | 1.42 | 0.15609 |
| haplo.base53 | 54 | W | G | G | C | 0.84231 | NA | NA | NA | NA |
| Geno.321 | 63 | G | A | C | G | 0.13381 | -1.15 | 0.71 | -1.62 | 0.10520 |
| Geno.717 | 63 | G | G | T | G | 0.23641 | 0.73 | 0.57 | 1.28 | 0.20162 |
| Geno.rare53 | 63 | * | * | * | * | 0.00745 | 4.40 | 3.12 | 1.41 | 0.15918 |
| haplo.base62 | 63 | G | G | C | G | 0.62233 | NA | NA | NA | NA |
| Geno.225 | 71 | A | G | A | A | 0.09162 | 0.18 | 0.83 | 0.22 | 0.82698 |
| Geno.326 | 71 | A | G | A | T | 0.01742 | 5.81 | 1.74 | 3.34 | 0.00088 |
| Geno.rare61 | 71 | * | * | * | * | 0.00948 | -2.37 | 2.69 | -0.88 | 0.37862 |
| haplo.base70 | 71 | A | G | G | A | 0.88148 | NA | NA | NA | NA |
| Geno.226 | 72 | G | A | A | A | 0.01423 | 2.53 | 2.03 | 1.25 | 0.21301 |
| Geno.327 | 72 | G | A | A | G | 0.07773 | -0.21 | 0.88 | -0.24 | 0.81144 |
| Geno.430 | 72 | G | A | T | G | 0.01742 | 5.91 | 1.74 | 3.40 | 0.00071 |
| Geno.rare62 | 72 | * | * | * | * | 0.00373 | 3.97 | 4.41 | 0.90 | 0.36868 |
| haplo.base71 | 72 | G | G | A | G | 0.88689 | NA | NA | NA | NA |
| Geno.227 | 73 | A | A | A | T | 0.01333 | 3.16 | 2.05 | 1.54 | 0.12390 |
| Geno.328 | 73 | A | A | G | C | 0.07750 | -0.33 | 0.88 | -0.38 | 0.70749 |
| Geno.533 | 73 | A | T | G | C | 0.01742 | 5.93 | 1.74 | 3.41 | 0.00068 |
| Geno.rare63 | 73 | * | * | * | * | 0.00418 | 5.37 | 4.50 | 1.19 | 0.23390 |
| haplo.base72 | 73 | G | A | G | C | 0.88757 | NA | NA | NA | NA |
| Geno.431 | 74 | A | A | T | G | 0.01295 | 2.87 | 2.11 | 1.36 | 0.17354 |
| Geno.534 | 74 | A | G | C | A | 0.03365 | 0.18 | 1.34 | 0.14 | 0.89176 |
| Geno.816 | 74 | T | G | C | G | 0.01749 | 5.98 | 1.73 | 3.45 | 0.00060 |
| Geno.rare64 | 74 | * | * | * | * | 0.00466 | 6.17 | 4.42 | 1.39 | 0.16367 |
| haplo.base73 | 74 | A | G | C | G | 0.93125 | NA | NA | NA | NA |

hap.freq: haplotype frequency; coef: coefficient; se: standard error; t.stat: test statistic; p-val: haplotype p-value; ^a^Box-Cox transformed data.
