# Supplementary material for: Apolipoprotein E-C1-C4-C2 gene cluster region and inter-individual variation in plasma lipoprotein levels: a comprehensive genetic association study in two ethnic groups
Source: PLoS One. 2019 Mar 26;14(3):e0214060. doi: 10.1371/journal.pone.0214060 (PMC6435132; doi:10.1371/journal.pone.0214060)
Supplement: S35 Table — hap.freq: haplotype frequency; coef: coefficient; se: standard error; t.stat: test statistic; p-val: haplotype p-value; aBox-Cox transformed data. (DOCX) [file pone.0214060.s035.docx]

S35 Table. Haplotype summary of significant windows with TG in Blacks

| **TG^a^** | | | | | | | | | | |
| --- | --- | --- | --- | --- | --- | --- | --- | --- | --- | --- |
|  | **Window** | **loc.1** | **loc.2** | **loc.3** | **loc.4** | **hap.freq** | **coef** | **se** | **t.stat** | **pval** |
| Geno.2 | 1 | C | A | C | T | 0.36137 | 0.003 | 0.008 | 0.424 | 0.67171 |
| Geno.6 | 1 | T | A | C | A | 0.06186 | 0.039 | 0.015 | 2.641 | 0.00843 |
| Geno.rare | 1 | * | * | * | * | 0.00668 | 0.060 | 0.045 | 1.313 | 0.18941 |
| haplo.base | 1 | C | A | C | A | 0.57009 | NA | NA | NA | NA |
| Geno.419 | 46 | C | T | G | T | 0.03746 | 0.061 | 0.018 | 3.375 | 0.00078 |
| Geno.516 | 46 | C | T | T | G | 0.04651 | -0.009 | 0.017 | -0.556 | 0.57807 |
| Geno.rare40 | 46 | * | * | * | * | 0.00470 | 0.060 | 0.056 | 1.084 | 0.27891 |
| haplo.base45 | 46 | C | T | G | G | 0.91133 | NA | NA | NA | NA |
| Geno.517 | 47 | T | G | T | T | 0.03746 | 0.061 | 0.018 | 3.388 | 0.00074 |
| Geno.622 | 47 | T | T | G | T | 0.04651 | -0.009 | 0.017 | -0.537 | 0.59118 |
| Geno.rare41 | 47 | * | * | * | * | 0.00337 | 0.131 | 0.068 | 1.933 | 0.05362 |
| haplo.base46 | 47 | T | G | G | T | 0.91266 | NA | NA | NA | NA |
| Geno.420 | 48 | G | T | T | T | 0.03808 | 0.063 | 0.018 | 3.506 | 0.00048 |
| Geno.518 | 48 | T | G | T | T | 0.04650 | -0.009 | 0.017 | -0.543 | 0.58745 |
| Geno.rare42 | 48 | * | * | * | * | 0.00205 | 0.159 | 0.096 | 1.653 | 0.09882 |
| haplo.base47 | 48 | G | G | T | T | 0.91336 | NA | NA | NA | NA |
| Geno.317 | 49 | G | T | T | A | 0.01264 | -0.003 | 0.032 | -0.104 | 0.91684 |
| Geno.519 | 49 | T | T | T | G | 0.03809 | 0.063 | 0.018 | 3.527 | 0.00044 |
| Geno.rare43 | 49 | * | * | * | * | 0.00205 | 0.160 | 0.096 | 1.662 | 0.09696 |
| haplo.base48 | 49 | G | T | T | G | 0.94723 | NA | NA | NA | NA |
| Geno.332 | 79 | A | G | C | A | 0.10358 | -0.009 | 0.013 | -0.683 | 0.49486 |
| Geno.632 | 79 | G | A | C | A | 0.33332 | 0.006 | 0.009 | 0.621 | 0.53458 |
| Geno.721 | 79 | G | A | C | G | 0.01407 | 0.079 | 0.037 | 2.124 | 0.03400 |
| Geno.818 | 79 | G | A | G | A | 0.01382 | -0.071 | 0.031 | -2.310 | 0.02116 |
| Geno.106 | 79 | G | G | C | G | 0.11102 | -0.034 | 0.013 | -2.540 | 0.01129 |
| Geno.rare69 | 79 | * | * | * | * | 0.00482 | 0.031 | 0.077 | 0.404 | 0.68667 |
| haplo.base78 | 79 | G | G | C | A | 0.41936 | NA | NA | NA | NA |
| Geno.125 | 80 | A | C | A | G | 0.01492 | -0.004 | 0.032 | -0.140 | 0.88873 |
| Geno.228 | 80 | A | C | A | T | 0.31847 | 0.007 | 0.009 | 0.804 | 0.42170 |
| Geno.333 | 80 | A | C | G | T | 0.01670 | 0.078 | 0.035 | 2.234 | 0.02579 |
| Geno.535 | 80 | A | G | A | T | 0.01293 | -0.073 | 0.032 | -2.308 | 0.02126 |
| Geno.98 | 80 | G | C | G | T | 0.10829 | -0.033 | 0.013 | -2.493 | 0.01287 |
| Geno.rare70 | 80 | * | * | * | * | 0.00188 | -0.082 | 0.127 | -0.647 | 0.51751 |
| haplo.base79 | 80 | G | C | A | T | 0.52681 | NA | NA | NA | NA |

hap.freq: haplotype frequency; coef: coefficient; se: standard error; t.stat: test statistic; p-val: haplotype p-value; ^a^Box-Cox transformed data.
